# Blockade of NKp46⁻ CCR6⁻ ILC3 autophagy protects against necrotizing enterocolitis by restoring energy metabolism balance in mice

**DOI:** 10.1038/s41467-026-73356-x

**Published:** 2026-05-19

**Authors:** Junyu He, Meiqi Chen, Laiqin Peng, Qiqiong Wang, Yizhuang Lu, Yimin Chen, Xinyao Li, Yanling Mou, Jianjun Wang, Yuxiong Guo, Kai Wu, Yumei He

**Affiliations:** 1 Department of Immunology, School of Basic Medical Sciences, Southern Medical University, Guangzhou, China; 2 Pediatric Intensive Care Unit and Guangdong Provincial Cardiovascular Institute, Guangdong Provincial People’s Hospital (Guangdong Academy of Medical Sciences), Southern Medical University, Guangzhou, China; 3 Department of Pediatric Surgery, Zhujiang Hospital, Southern Medical University, Guangzhou, China; 4 Department of Gynecology and Obstetrics, Huizhou Central People’s Hospital, Huizhou, China; 5 Department of Neonatology, Nanfang Hospital, Southern Medical University, Guangzhou, China; 6 Guangdong Provincial Key Laboratory of Single Cell and Extracellular Vesicles, Southern Medical University, Guangzhou, China

**Keywords:** Innate lymphoid cells, Acute inflammation, Autophagy, Metabolism

## Abstract

Group 3 innate lymphoid cells (ILC3) are crucial in neonatal necrotizing enterocolitis (NEC); however, the underlying mechanisms remain elusive. Here, we identify NKp46⁻CCR6⁻ (double-negative, DN) ILC3s as the dominant pathogenic subset driving NEC via IL-17 A secretion, which disrupts intestinal barrier integrity. Mechanistically, Atg5 activates autophagy in DN ILC3s during NEC. Atg5 conditional knockout in RORγt⁺ cells mitigates NEC, reduces DN ILC3 accumulation and IL-17 A production. Atg5 deficiency also decreases HIF-1α chromatin accessibility and transcriptional activity, shifting DN ILC3 metabolism from glycolysis to fatty acid oxidation. Lipidomics reveals phosphatidylcholine as a key downstream metabolite of Atg5-mediated autophagy. Phosphatidylcholine supplementation suppresses DN ILC3-driven inflammation, restores metabolic homeostasis, elevates *Clostridium* abundance, and ameliorates NEC in mice. Importantly, human NEC tissues exhibit increased ILC3 proportions, autophagic activity, and IL-17 A/IL-22 secretion. Thus, we uncover an Atg5–autophagy–glycolipid metabolic axis in DN ILC3s that drives NEC pathogenesis, providing a promising therapeutic target for neonatal NEC.

## Abstract (web summary)

Group 3 innate lymphoid cells (ILC3s) are key regulator of intestinal immunity. However, whether these cells drive necrotizing enterocolitis (NEC) and through which mechanism remains unexplored. Here, the authors employ a mouse model of neonatal NEC and implicate NKp46⁻CCR6⁻ double negative ILC3 autophagy in pathogenic ILC3 expansion and activation during NEC via HIF1a-modulation of glycolipid metabolism.

## Introduction

Necrotizing enterocolitis (NEC) is a severe gastrointestinal disorder affecting approximately 10% of preterm infants weighing less than 1500 g and represents a major threat to neonatal survival^1^. Pathologically, NEC is characterized by inflammation and necrosis of the small intestine, predominantly the distal ileum^2^. Intestinal dysbiosis triggers Toll-like receptor 4 (TLR4) signaling in intestinal epithelial cells (IEC), which compromises intestinal barrier integrity, facilitating bacterial translocation, and increases susceptibility to sepsis^3–5^. TLR4 activation also directly drives epithelial cell death through apoptosis, autophagy, and necroptosis^6–9^. This epithelial damage is worsened by impaired proliferation and mucosal repair, and further amplified by neutrophil infiltration into the small intestinal mucosa, exacerbating NEC-associated inflammation^10–13^. Current therapeutic strategies for NEC remain limited and have not substantially improved patient survival, underscoring the urgent need for novel therapeutic approaches^14^.

Central to these pathological changes is intestinal immune dysregulation—a key driver of NEC in preterm infants. It features imbalances among CD4⁺ effector T cells, γδ T cells, Th17 cells, and regulatory T (Treg) cells, along with their signature cytokines, fueling excessive inflammation^14–16^. Recent advances in NEC pathoimmunology have further demonstrated that elevated abundance of group 3 innate lymphoid cells (ILC3) and reduced interleukin-37 (IL-37) production are closely linked to disease development^17^. In murine models of NEC, these cells produce markedly increased interleukin-17 A (IL-17 A), which may contribute to the enhanced susceptibility of neonates to intestinal inflammation^18^.

ILC3s are key regulators of intestinal immunity, with distinct subsets exhibiting context-dependent protective or pathogenic functions^19,20^. In inflammatory bowel disease (IBD), ILC3s generate cytokines IL-17A, IL-22, and granulocyte-macrophage colony-stimulating factor (GM-CSF, encoded by *Csf2*), which promote mucosal protection through antimicrobial peptide production^19–21^. However, excessive cytokine secretion by ILC3s can drive neutrophilic inflammation and exacerbate tissue damage^22^. ILC3s encompass highly phenotypically and functionally heterogeneous subsets defined by surface marker expression. NKp46^+^ ILC3s maintain intestinal homeostasis through IL-23-driven, tissue-protective IL-22 production during *Citrobacte* (C.) *rodentium* infection^23,24^. CCR6^+^ ILC3s, encompassing fetal lymphoid tissue inducer cells and adult lymphoid tissue inducer-like cells, contribute to intestinal lymphoid tissue formation and provide an innate source of IL-17A, IL-17F, IL-22, and GM-CSF upon stimulation^25,26^. In addition, the small intestine harbors a poorly characterized “double-negative” subset: CCR6^−^NKp46^−^ ILC3 (DN ILC3)^27,28^. Notably, the functional role of DN ILC3s in intestinal inflammation—especially in neonatal NEC—remains completely unknown. Given their unique phenotype and tissue localization, the precise contribution of DN ILC3s to NEC pathogenesis warrants in-depth investigation.

Metabolic reprogramming of immune cells is a fundamental regulator in intestinal inflammation, with distinct metabolic pathways dictating the functional plasticity of ILC3s in health and disease. In ILC3s, the mTORC1-hypoxia-inducible factor 1α (HIF1α) axis pathway promotes glycolysis and RORγt-dependent cytokine production, whereas LKB1 preserves mitochondrial integrity and energy homeostasis. Disruption of these pathways exacerbates colitis in murine models^29,30^. Dietary lipids also modulate ILC3 function: saturated fats suppress IL-22 production by downregulating RORγt and inducing mitochondrial stress, whereas unsaturated fats support IL-22 secretion through lipid droplet-mediated fatty acid oxidation (FAO)^31^. Notably, these findings are largely derived from studies in adult mice, with limited investigation in neonatal systems, particularly neonatal NEC.

Although direct evidence linking ILC3 metabolism to NEC pathogenesis remains scarce, autophagy has emerged as a key contributor to disease development. In murine NEC models, TLR4 activation induces autophagy in IECs, thereby impairing epithelial migration and promoting disease progression^32^. Additionally, the elevated expression of autophagy-related genes in intestinal tissues from patients with NEC further implicates autophagy as a critical pathogenic mechanism^33,34^. Beyond its pathogenic effects, autophagy functions as a central regulator of cellular metabolism, shaping energy homeostasis by modulating glucose and fatty acid metabolism, and mitochondrial bioenergetic pathways in immune cells^35,36^. For example, autophagy provides free fatty acids through lipophagy, the selective degradation of lipid droplets, facilitating a metabolic shift from glycolysis to FAO and oxidative phosphorylation (OXPHOS) to sustain cellular energy demands^37–40^. While immunometabolic crosstalk in immune cell regulation is well established, the role of an autophagy–energy metabolism axis in controlling DN ILC3 effector function during NEC remains completely unexplored. Clarifying this mechanism will deepen our understanding of how autophagy-driven DN ILC3s contribute to NEC pathogenesis, and holds promise for identifying cell-specific therapeutic targets. Such insights may guide the development of novel strategies for the prevention, early diagnosis, or treatment of NEC in preterm infants.

In this study, we characterize the identity and regulation of pathogenic ILC3 subsets in NEC, define DN ILC3s as key drivers of disease, and establish a causal link between Atg5-mediated autophagy, metabolic reprogramming, and their pathogenic activation. We further validate the clinical relevance of this axis by profiling corresponding molecular signatures in human intestinal tissues from NEC patients. Together, these findings uncover a novel immunometabolic regulatory axis in neonatal intestinal inflammation, providing new insights for the development of targeted preventive and therapeutic strategies for NEC.

## Results

### DN ILC3s are a key ILC3 subset in neonatal mice with NEC

First, to rule out the potential impact of the gavage procedure on the neonatal mouse model of NEC. Littermate pups at postnatal day 8(P8) were randomly divided into a control group (exclusive breastmilk feeding) and an experimental group receiving four daily gavages of sterile phosphate-buffered saline (PBS) with concurrent breast milk feeding (Supplementary Fig. 1a). Oral gavage alone did not induce intestinal injury in neonatal mice, as evidenced by no body weight gain and comparable intestinal pathological scores relative to breastfed controls (Supplementary Fig. 1b, c). Next, we established the NEC mouse model by randomly assigning littermate P8 pups to either the breast-fed control group (CON) or the NEC induction group (NEC) (Supplementary Fig. 1d). Compared with CON mice, NEC mice exhibited significant body weight loss, marked intestinal mucosal shedding and increased intestinal permeability (Supplementary Fig. 1e–g). Flow cytometry analysis revealed that the proportion and absolute number of IECs were significantly reduced in NEC mice, accompanied by increased apoptosis and decreased Ki67^+^ proliferative activity (Supplementary Fig. 1h–j). The mRNA levels of the tight junction proteins *ZO-1*, *Claudin-1*, and *Occludin* were significantly downregulated in NEC mice, indicating impaired intestinal barrier function and confirming the successful establishment of the NEC model (Supplementary Fig. 1k).

**Fig. 1:**
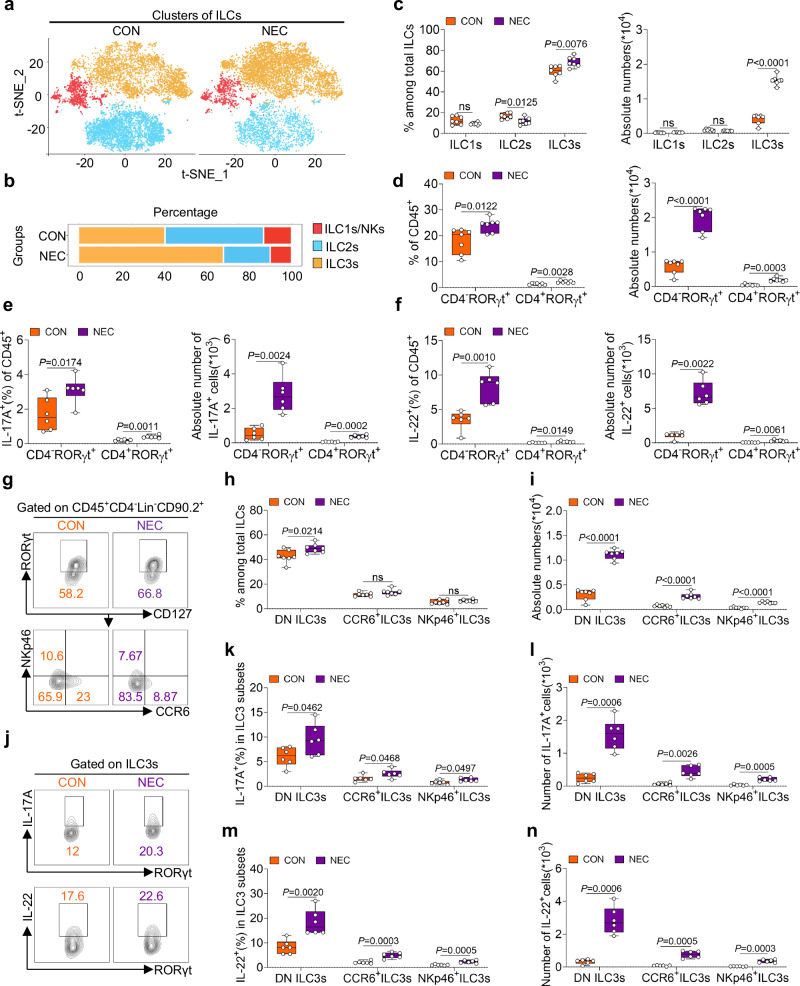
The intestinal DN ILC3 subset is markedly increased in both abundance and function after NEC induction. **a** T-distributed stochastic neighbor embedding (t-SNE) analysis comparing ILC clusters in the intestines of breast-fed control (CON) and NEC model (NEC) neonatal mice. **b** Median percentage of each ILC cluster, color-coded to match the populations in (**a**). Percentage and absolute number of intestinal ILC1s, ILC2s, ILC3s the intestinal lamina propria (*n* = 7 biological replicates per group) (**c**); intestinal CD4^−^RORγt^+^ and CD4^+^RORγt^+^ cells (*n* = 7 biological replicates per group) (**d**); intestinal IL-17A^+^CD4^−^RORγt^+^ and IL-17A^+^CD4^+^RORγt^+^ cells (*n* = 6 biological replicates per group) (**e**); and intestinal IL-22^+^CD4^−^RORγt^+^ and IL-22^+^CD4^+^RORγt^+^ cells (*n* = 6 biological replicates per group) (**f**). **g** Representative flow cytometry profiles of intestinal ILC3 subsets, including double negative (DN) ILC3s, CCR6^+^ ILC3s, and NKp46^+^ ILC3s. Percentage (**h**) and absolute number (**i**) of intestinal DN ILC3s, CCR6^+^ ILC3s, and NKp46^+^ ILC3s (*n* = 7 biological replicates per group). **j** Representative flow cytometry profiles of intestinal IL-17A^+^ ILC3s and IL-22^+^ ILC3s. Percentage (**k**) and absolute number (**l**) of intestinal IL-17A^+^ ILC3s (*n* = 6 biological replicates per group). Percentage (**m**) and absolute number (**n**) of intestinal IL-22^+^ ILC3s (*n* = 6 biological replicates per group). All experiments were performed using C57BL/6 mice of both sexes at P8, with littermates randomly assigned to control and experimental groups. Each data point represents one biologically independent mouse, and results are representative of at least three independent experiments. Box plots show the median (center line, 50th percentile), with the lower and upper bounds of the box representing the 25th and 75th percentiles, respectively. Whiskers extend to the absolute minimum and maximum values (0th and 100th percentiles, respectively) of the dataset. *P*-values were determined by unpaired two tailed Student’s t tests (**c**–**f**,**h**,**i**, and **k**–**n**). ns = not significant. Source data are provided as a Source Data file.

To define the role of ILC subsets in NEC pathogenesis, we isolated ILCs, which were defined as CD45^+^CD90.2^+^Lineage^−^ (CD3, B220, CD11b, Ly6G, TER-119, CD11c, CD5, CD8a, TCRαβ, and TCRγδ), from the intestines of CON and NEC mice for single-cell RNA sequencing (scRNA-seq) (Supplementary Fig. 2a). Among the three identified subsets—ILC1s, ILC2s, and ILC3s—ILC3s were the dominant population (Fig. 1a and Supplementary Fig. 2b). Notably, NEC mice exhibited a significantly higher abundance of ILC3s in the intestine relative to ILC1s and ILC2s. Additionally, their ILC3 levels were significantly increased compared with the CON mice. In contrast, ILC2s were reduced in NEC mice, whereas ILC1s showed no significant changes (Fig. 1b). Consistent with our scRNA-seq data, flow cytometry analysis confirmed that both the proportion and absolute number of ILC3s were significantly increased after NEC induction. The proportion of ILC2s was reduced, but their absolute numbers remained unchanged, and neither the proportion nor absolute number of ILC1s changed significantly (Fig. 1c and Supplementary Fig. 2c–e). Notably, oral gavage alone had no significant effect on the proportion or absolute number of CD45⁺ immune cells, ILC1s, ILC2s, or ILC3s (Supplementary Fig. 3a–d). Given that ILC3s are defined by RORγt expression, we next analyzed RORγt⁺ cell subsets before in steady-state and NEC-induced conditions. Of the two major identified subsets, CD4⁻RORγt⁺ cells were the dominant RORγt⁺ population in NEC mice, whereas CD4⁺RORγt⁺ cells remained negligible (Fig. 1d and Supplementary Fig. 4a). Within the CD4⁻RORγt⁺ subset, neither the proportion nor absolute number of TCRαβ⁺ or TCRγδ⁺ cells changed significantly following NEC induction (Supplementary Fig. 4b, c).

Following previous reports of increased CD4⁺RORγt⁺IL-17A⁺ cells during NEC development, we examined IL-17A and IL-22 production by CD4⁻RORγt⁺ and CD4⁺RORγt⁺ cells. The proportion and absolute number of CD4⁺RORγt⁺IL-17A⁺ cells were increased after NEC induction, but remained three-fold lower than the corresponding values for CD4⁻RORγt⁺IL-17A⁺ cells (Fig. 1e and Supplementary Fig. 4d, e). Similarly, IL-22 production was also predominantly derived from CD4⁻RORγt⁺ cells (Fig. 1f and Supplementary Fig. 4d, e). Considering the observed changes in CD4⁺RORγt⁺ cell abundance (which nevertheless remained low) and functional properties following NEC induction, we investigated the potential role of T cell deficiency in NEC pathogenesis. NEC was induced in Rag2-knockout (KO) mice, which lack mature T and B cells, and wild-type (WT) control mice (Supplementary Fig. 5a). No significant differences were observed in body weight trajectory, hematoxylin–eosin (H&E) staining disease score (Supplementary Fig. 5b, c), or intestinal ILC3 abundance and function (Supplementary Fig. 5d–f) between Rag2 KO and WT mice. These findings suggest a strong link between CD4^−^RORγt^+^ cells, particularly ILC3s, and NEC progression.

To clarify the role of ILC3s in NEC development, we next characterized ILC3 subpopulations and their functional profiles. Following NEC induction, both the proportion and absolute number of ILC3s were significantly increased, with DN ILC3s showing the most pronounced increase. While the proportions of CCR6⁺ ILC3s and NKp46⁺ ILC3s remained unchanged, their absolute numbers were significantly elevated after NEC induction (Fig. 1g–i). Functional analysis revealed marked increases in the proportions and absolute numbers of IL-17A⁺ and IL-22⁺ ILC3s in the NEC mice, particularly within the DN ILC3 subset (Fig. 1j–n). Notably, DN ILC3s maintained consistently high frequencies from the early postnatal period to adulthood. In contrast, NKp46⁺ ILC3s increased only after two weeks of age, whereas CCR6⁺ ILC3s were reduced in adulthood (Supplementary Fig. 6a, b). Collectively, these findings identify DN ILC3s as a key subset involved in NEC progression in neonatal mice.

### DN ILC3s drive NEC progression through IL-17A

To further identify the major pathogenic ILC3 subset driving NEC progression, we performed adoptive transfer experiments using individual ILC3 subsets. We first transferred ILC3 subsets at proportions matching their relative abundance in vivo (Supplementary Fig. 7a). Compared with PBS-treated controls, NEC mice receiving NEC-derived DN ILC3s exhibited exacerbated body weight loss and intestinal inflammation, whereas NEC-derived NKp46⁺ ILC3s and CCR6⁺ ILC3s had no significant effect on either body weight loss or intestinal inflammation (Supplementary Fig. 7b, c). To exclude confounding effects from differing cell number, we performed parallel adoptive transfer using equal numbers of each ILC3 subset (Supplementary Fig. 7d). Consistent with the above results, DN ILC3s aggravated body weight loss and intestinal inflammation, while NKp46⁺ ILC3s and CCR6⁺ ILC3s did not induce obvious pathological changes (Supplementary Fig. 7e, f). Collectively, these data identify DN ILC3s as the key pathogenic ILC3 subset driving NEC onset.

**Fig. 2:**
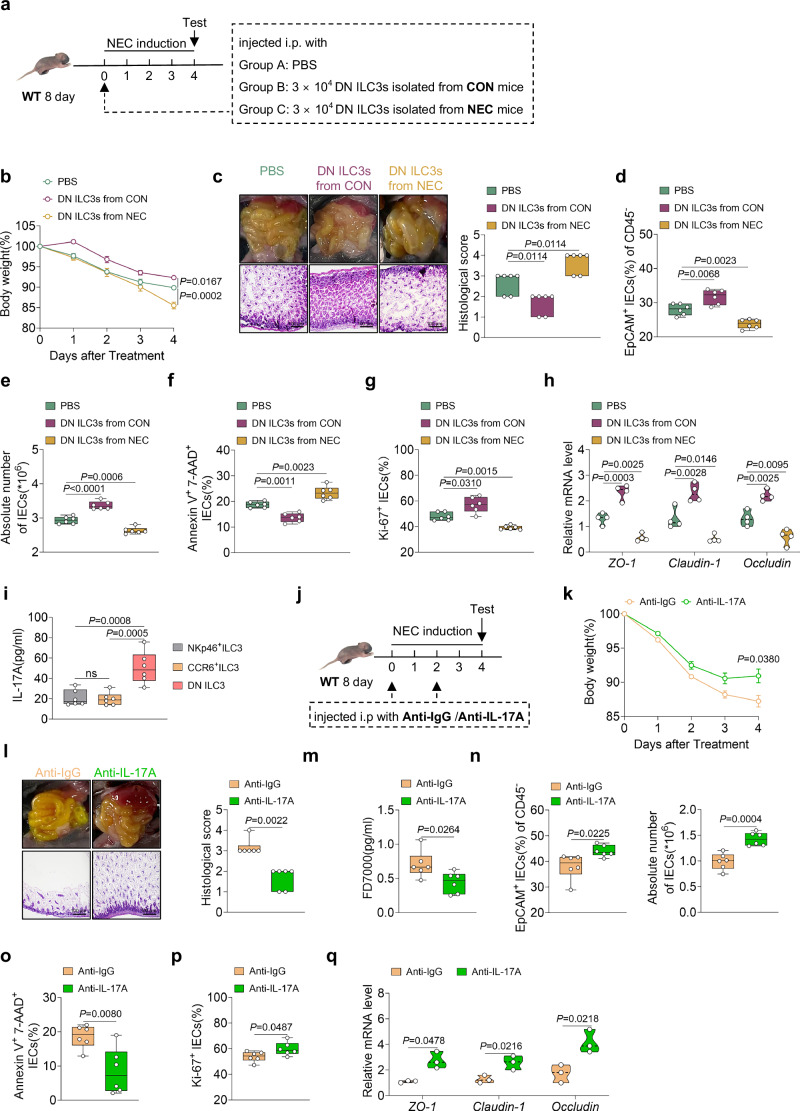
Pathogenic DN ILC3s exacerbate neonatal NEC via IL-17A, which is reversed by treatment with the neutralizing anti-IL-17A antibody. **a** Experimental schema of adoptive transfer during NEC induction. **b** Body weight changes in the indicated groups (*n*  =  6 biological replicates per group). Data are presented as mean ± SEM. **c** Macroscopic image and hematoxylin and eosin (H&E) staining of the intestines (scale bar: 100 μm). Inflammation scores were calculated based on the severity of intestinal inflammation (*n*  =  6 biological replicates per group). Percentage (**d**) and absolute number (**e**) of IEC in CD45^**−**^ cells (*n*  =  6 biological replicates per group). **f** Statistical analysis of Annexin V^+^7-AAD^+^ IECs (*n*  =  6 biological replicates per group). **g** Statistical analysis of Ki67^+^ IECs (*n*  =  6 biological replicates per group). **h** Relative mRNA levels of *ZO-1*, *Claudin-1*, and *Occludin* in the intestines (*n*  =  4 biological replicates per group). **i** IL-17A production by intestinal DN ILC3s, CCR6^+^ ILC3s, and NKp46^+^ ILC3s were measured using enzyme-linked immunosorbent assay (*n*  =  6 biological replicates per group). **j** Experimental schema of neutralizing anti-IL-17A antibody (anti-IL-17A) during NEC induction. **k** Body weight changes in the indicated groups (*n*  =  6 biological replicates per group). Data are presented as mean ± SEM. **l** Macroscopic image and H&E staining of the intestines (scale bar: 100 μm), with corresponding inflammation scores (*n*  =  6 biological replicates per group). **m** Intestinal permeability was assessed by plasma FD7000 concentrations (*n*  =  6 biological replicates per group). **n** Percentage and absolute number of IECs (*n*  =  6 biological replicates per group). **o** Statistical analysis of Annexin V^+^7-AAD^+^ IECs (*n*  =  6 biological replicates per group). **p** Statistical analysis of Ki67^+^ IECs (*n*  =  6 biological replicates per group). **q** Relative mRNA levels of *ZO-1*, *Claudin-1*, and *Occludin* in the intestines (*n*  =  3 biological replicates per group). All experiments were performed using C57BL/6 mice of both sexes at P8, with littermates randomly assigned to control and experimental groups. Each data point represents one biologically independent mouse, and results are representative of at least three independent experiments. Box plots show the median (center line, 50th percentile), with the lower and upper bounds of the box representing the 25th and 75th percentiles, respectively. Whiskers extend to the absolute minimum and maximum values (0th and 100th percentiles, respectively) of the dataset. *P*-values were determined by one-way ANOVA followed by Tukey-Kramer multiple comparisons test (**b**–**i**) or unpaired two tailed Student’s t tests (**k**–**q**). Source data are provided as a Source Data file.

Accordingly, we established an NEC model by injecting equal numbers of steady-state (physiological) or NEC-derived (pathological) DN ILC3s into WT mice once before NEC induction, using PBS as a control (Fig. 2a). Mice receiving physiological DN ILC3s exhibited less severe weight loss and significantly lower intestinal inflammation scores than the PBS group. In contrast, transfer of pathological DN ILC3s accelerated weight loss and exacerbated intestinal inflammation (Fig. 2b, c). Furthermore, transfer of pathological DN ILC3s markedly reduced the proportion and absolute number of IECs, suppressed IEC proliferative capacity, and promoted IEC apoptosis. Conversely, administration of physiological DN ILC3s effectively reversed these detrimental effects, restored the proportion and absolute number of IECs, enhanced IEC proliferation, and reduced IEC apoptosis (Fig. 2d–g and Supplementary Fig. 8a–c). Additionally, transfer of pathological DN ILC3s downregulated the expression of the tight junction proteins *ZO-1*, *Claudin-1*, and *Occludin*, whereas physiological DN ILC3s reversed this effect (Fig. 2h). These results demonstrate that pathological DN ILC3s exacerbate intestinal inflammation and promote NEC development.

To investigate the functional relevance of the reduced ILC2 frequency observed in NEC mice, we performed additional adoptive transfer experiments (Supplementary Fig. 9a). Transfer of steady-state ILC2s from one-week-old conferred significant protection against body weight loss and intestinal inflammation. In contrast, ILC2s isolated from eight-week-old mice exhibited a markedly diminished protective activity, with only a modest protective trend that did not reach statistical significance (Supplementary Fig. 9b, c). These results indicate that intestinal neonatal ILC2s critically protect against NEC. Given that both T cells and DN ILC3s exert pathogenic functions during NEC development, we further evaluated their contributions to disease progression by performing adoptive transfer of CD4⁺ T cells or DN ILC3s into Rag2 KO mice (Supplementary Fig. 10a). Transfer of 3 × 10^4^ DN ILC3s significantly exacerbated NEC severity, whereas transfer of an equal number (3 × 10^4^) of CD4⁺ T cells had no obvious effect on NEC pathology. A substantially higher number (4 × 10^6^) of CD4⁺ T cells was required to achieve a comparable pathogenic effect (pronounced body weight loss and aggravated intestinal inflammation) to that of DN ILC3s (Supplementary Fig. 10b, c). These findings indicate that DN ILC3s are stronger drivers of NEC pathogenesis than CD4⁺ T cells.

To define the critical molecules responsible for DN ILC3‑driven pathogenesis in NEC, we used scRNA‑seq data to profile cytokine expression among ILC3 subsets (Supplementary Fig. 11a). Subset analysis revealed that, compared with controls, *Il17a* and *Csf2* upregulation was significantly more pronounced in DN ILC3s than in NKp46⁺ ILC3s and CCR6⁺ ILC3s. In contrast, *Il22* expression in DN ILC3s tended to increase albeit non-significantly, with a far more modest and statistically insignificant change compared to the robust upregulation observed in NKp46⁺ ILC3s (Supplementary Fig. 11b). To validate these findings at the protein level, equal numbers of sorted ILC3 subsets from NEC mice were cultured in vitro, and their cytokine secretion profiles were assessed (Supplementary Fig. 11c). Consistent with our transcriptomic analysis, IL-17 A production by DN ILC3s was significantly higher than that by other ILC3 subsets (Fig. 2i). In contrast, secretion levels of GM-CSF and IL-22 were comparable among the three subsets (Supplementary Fig. 11d). To determine the functional roles of these cytokines in NEC pathogenesis, we treated NEC mice with neutralizing antibodies against IL‑17 A, IL‑22, and GM‑CSF. Anti-IL-17A treatment significantly attenuated body weight loss and intestinal pathological damage in NEC mice (Fig. 2j–l), and effectively restored intestinal barrier function, as evidenced by reduced intestinal permeability, increased IEC proportion and absolute number, enhanced IEC proliferation and survival, and upregulated expression of tight junction proteins (Fig. 2m–q and Supplementary Fig. 12a–c). In contrast, IL-22 neutralization failed to mitigate body weight loss and intestinal pathological damage in NEC mice, with no significant alterations in all examined parameters including intestinal permeability, the proportion, absolute number, proliferation and survival of IECs, and tight junction protein expression (Supplementary Fig. 13a–h). Consistent with these findings, GM-CSF neutralization also did not alleviate body weight loss, intestinal pathological injury, or alter the above intestinal barrier-related parameters in NEC mice (Supplementary Fig. 13i–p).

### DN ILC3 autophagy plays a crucial role in NEC development

**Fig. 3:**
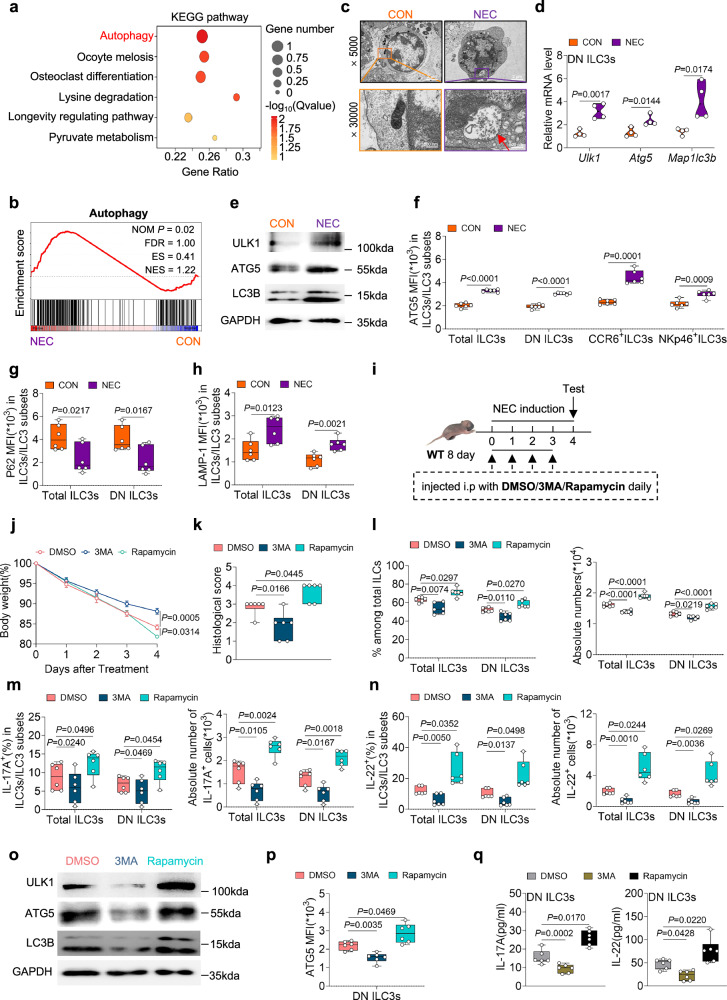
Autophagy activation in intestinal DN ILC3s aggravates NEC severity. **a** Kyoto Encyclopedia of Genes and Genomes (KEGG) analysis of differentially expressed genes in DN ILC3 of CON and NEC neonatal mice. **b** Gene Set Enrichment Analysis (GSEA) showing autophagy upregulation in DN ILC3 from NEC neonates (FDR, false discovery rate; NES, normalized enrichment score). **c** Representative transmission electron microscopy images of intestinal DN ILC3s sorted from CON and NEC neonates. Red arrows indicate autophagosome structures. Scale bars: 2 μm (×5,000 magnification, top panels); 500 nm (×30,000 magnification, bottom panels). **d** Relative mRNA levels of *Ulk1*, *Atg5*, and *Map1lc3b* in intestinal DN ILC3s (*n*  =  4 biological replicates per group). **e** Western blot analysis of ULK1, ATG5, and MAP1LC3b (LC3B) in sorted intestinal DN ILC3s (replicated twice, isolated from 14 neonates per replicated). **f** Protein levels of ATG5 in intestinal total ILC3s and ILC3 subsets (*n*  =  6 biological replicates per group). **g** Protein levels of P62 in intestinal total ILC3s and DN ILC3s (*n*  =  6 biological replicates per group). **h** Protein levels of LAMP-1 in intestinal total ILC3s and DN ILC3s (*n*  =  6 biological replicates per group). **i** Schematic diagram of autophagy interventions during NEC induction. **j** Body weight changes in the indicated groups (*n*  =  6 biological replicates per group). Data are presented as mean ± SEM. **k** Statistical analysis of histological scores (*n*  =  6 biological replicates per group). **l**–**n** Percentage and absolute number of intestinal total ILC3s and DN ILC3s (*n*  =  6 biological replicates per group) (**l**); intestinal IL-17A^+^ total ILC3s and IL-17A^+^ DN ILC3s (*n*  =  6 biological replicates per group) (**m**); and intestinal IL-22^+^ total ILC3s and IL-22^+^ DN ILC3s (*n*  =  6 biological replicates per group) (**n**). **o** Western blot analysis of ULK1, ATG5, and MAP1LC3b (LC3B) in sorted intestinal DN ILC3s (replicated twice, isolated from 14 neonates per replicated). **p** Protein levels of ATG5 in intestinal DN ILC3s (*n*  =  6 biological replicates per group). **q** IL-17A and IL-22 production in sorted intestinal DN ILC3s treated with 3-MA or rapamycin for 48 h (*n*  =  6 biological replicates per group). All experiments were performed using C57BL/6 mice of both sexes at P8, with littermates randomly assigned to control and experimental groups. Each data point represents one biologically independent mouse, and results are representative of at least three independent experiments. Box plots show the median (center line, 50th percentile), with the lower and upper bounds of the box representing the 25th and 75th percentiles, respectively. Whiskers extend to the absolute minimum and maximum values (0th and 100th percentiles, respectively) of the dataset. *P*-values were determined by one-way ANOVA followed by Tukey-Kramer multiple comparisons test (**j**–**n,** **p**, and **q**) or unpaired two tailed Student’s t tests (**d** and **f**–**h**). Source data are provided as a Source Data file.

We next performed Kyoto Encyclopedia of Genes and Genomes (KEGG) and gene set enrichment analysis (GSEA) on the scRNA-seq data. The autophagy pathway was significantly enriched in ILC3s following NEC induction (Fig. 3a, b). Notably, the most significantly enriched pathways across the three major ILC3 subsets were all associated with metabolism. Of these, the mTOR pathway—a canonical metabolic signaling cascade that negatively regulates autophagy—was specifically enriched in DN ILC3s (Supplementary Fig. 14a–c). These observations suggest that autophagy represents a critical biological process in DN ILC3s during NEC pathogenesis.

Based on these findings, we systematically assessed autophagy activation in DN ILC3s at morphological, transcriptional, and protein levels. Transmission electron microscopy revealed the typical double-membrane autophagosomes in DN ILC3s from the NEC group, directly confirming autophagy induction in this subset (Fig. 3c). Both the mRNA and protein levels of key autophagy-related genes were also significantly elevated in DN ILC3s during NEC progression (Fig. 3d, e). Among these, Atg5 expressions were significantly increased in all ILC3s (Fig. 3f). Further analysis of downstream autophagy-related proteins showed that DN ILC3s, but not CCR6⁺ or NKp46⁺ ILC3s, displayed significantly reduced P62 and increased LAMP-1 expression following NEC induction (Fig. 3g, h and Supplementary Fig. 15a, b). These results identify enhanced autophagy as a specific feature of DN ILC3s, implicating this pathway in the pro-inflammatory effector function of DN ILC3s during NEC.

To further investigate the functional role of autophagy in DN ILC3s during NEC, we treated NEC mice with the autophagy inhibitor 3-methyladenine (3-MA) or the agonist rapamycin (RAP) (Fig. 3i)^41^. Intraperitoneal injection of 3-MA significantly attenuated weight loss and intestinal disease scores, whereas RAP treatment worsened these disease parameters (Fig. 3j, k and Supplementary Fig. 16a). Consistent with these phenotypic changes, the frequency and absolute number of total ILC3s were significantly reduced by 3-MA treatment and increased by RAP treatment. These effects were largely driven by changes in the DN ILC3 subset, as neither treatment significantly affected CD4⁺RORγt⁺ cells, NKp46⁺ ILC3s, or CCR6⁺ ILC3s (Fig. 3l and Supplementary Fig. 16b–e).

In both total ILC3s and the DN ILC3s, the proportions of IL-17A⁺ and IL-22⁺ cells were significantly decreased by 3-MA treatment but markedly increased by RAP treatment (Fig. 3m, n and Supplementary Fig. 17a). In contrast, cytokine production by the CD4⁺RORγt⁺, NKp46⁺ ILC3, and CCR6⁺ ILC3 subsets remained unchanged following autophagy modulation (Supplementary Fig. 17b–e). At the molecular level, the expression of key autophagy-related proteins (ULK1, ATG5, and LC3B) in DN ILC3s was reduced by 3-MA treatment but enhanced by RAP treatment (Fig. 3o). Flow cytometry further confirmed that ATG5 expression was specifically modulated in DN ILC3s under these treatment conditions (Fig. 3p and Supplementary Fig. 17f), with no significant changes detected in NKp46⁺ ILC3s or CCR6⁺ ILC3s (Supplementary Fig. 17g, h). Collectively, these results demonstrate that autophagy selectively regulates the expansion and function of pathogenic DN ILC3s during NEC progession.

To verify the direct regulation of autophagy on DN ILC3 function, we isolated intestinal DN ILC3s from NEC mice and cultured them in vitro with 3-MA or RAP for 48 h. Consistent with our in vivo observations, 3-MA treatment significantly reduced IL-17A and IL-22 production by NEC DN ILC3s, whereas RAP markedly enhanced their secretion (Fig. 3q). These findings confirm that autophagy directly regulates the effector functions of DN ILC3s in NEC.

### Atg5 is a key protein driving NEC progression in DN ILC3s

To clarify the role of Atg5 in DN ILC3s during NEC, we selectively deleted *Atg5* from RORγt-positive cells in mice (Rorc^cre^Atg5^fl/fl^). Atg5 expression in CD4^+^RORγt^+^ and ILC3s from lamina propria mononuclear cells was significantly decreased in Rorc^cre^Atg5^fl/fl^ mice, confirming the specific deficiency of *Atg5* in RORγt^+^ cells (Supplementary Fig. 18a, b). Compared with Atg5^fl/fl^ mice, Rorc^cre^Atg5^fl/fl^ mice showed no significant changes in intestinal inflammation scores, cell ratios, or absolute numbers of CD4^+^RORγt^+^, CD4^−^RORγt^+^, total ILC3, or ILC3 subsets (Supplementary Fig. 18c–g), nor in IL-17A or IL-22 secretion from ILC3s (Supplementary Fig. 18h–k). The proportions and absolute numbers of ILC1s and ILC2s among LPMCs were similar between *Atg5*-deficient and control mice (Supplementary Fig. 18l–o).

**Fig. 4:**
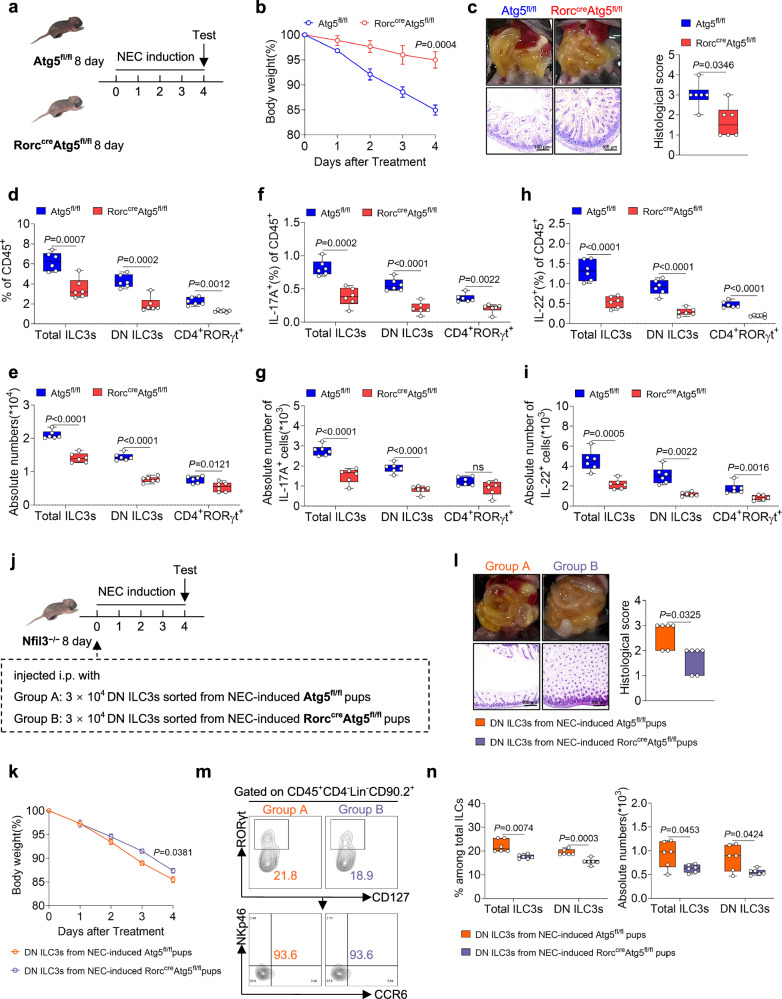
ATG5 plays an essential role in intestinal DN ILC3s during NEC progression. **a** Experimental NEC was induced in Atg5^fl/fl^ and Rorc^cre^Atg5^fl/fl^ neonates. **b** Body weight changes in the indicated groups (*n*  =  6 biological replicates per group). Data are presented as mean ± SEM. **c** Macroscopic image and H&E staining of the intestines (scale bar: 100 μm), with corresponding inflammation scores (*n*  =  6 biological replicates per group). Percentage (**d**) and absolute number (**e**) of intestinal total ILC3s, DN ILC3s, and CD4^+^RORγt^+^ cells (*n*  =  6 biological replicates per group). Percentage (**f**) and absolute number (**g**) of intestinal IL-17A^+^ total ILC3s, IL-17A^+^ DN ILC3s, and IL-17A^+^CD4^+^RORγt^+^ cells (*n*  =  6 biological replicates per group). Percentage (**h**) and absolute number (**i**) of intestinal IL-22^+^ total ILC3s, IL-22^+^ DN ILC3s, and IL-22^+^CD4^+^RORγt^+^ cells (*n*  =  6 biological replicates per group). **j** Experimental schema of adoptive transfer during NEC induction. **k** Body weight changes in the indicated groups (*n*  =  6 biological replicates per group). Data are presented as mean ± SEM. **l** Macroscopic image and H&E staining of the intestines (scale bar: 100 μm), with corresponding inflammation scores (*n*  =  6 biological replicates per group). **m** Representative flow cytometry profiles of ILC3 subsets in the intestinal lamina propria. **n** Percentage and absolute number of intestinal total ILC3s and DN ILC3s (*n*  =  6 biological replicates per group). All experiments were performed using Atg5^fl/fl^ and Rorc^cre^Atg5^fl/fl^ mice of both sexes at P8, with littermates randomly assigned to control and experimental groups. Each data point represents one biologically independent mouse, and results are representative of at least three independent experiments. Box plots show the median (center line, 50th percentile), with the lower and upper bounds of the box representing the 25th and 75th percentiles, respectively. Whiskers extend to the absolute minimum and maximum values (0th and 100th percentiles, respectively) of the dataset. *P*-values were determined by unpaired two tailed Student’s t tests (**b**–**i**, **k**, **l**, and **n**). ns = not significant. Source data are provided as a Source Data file.

We next performed NEC induction in Atg5^fl/fl^ and Rorc^cre^Atg5^fl/fl^ mice to investigate the impact of *Atg5* deletion on DN ILC3-induced intestinal inflammation (Fig. 4a). Rorc^cre^Atg5^fl/fl^ mice exhibited significantly attenuated weight loss and reduced intestinal pathology scores than Atg5^fl/fl^ mice (Fig. 4b, c). Following NEC induction, Rorc^cre^Atg5^fl/fl^ mice had significantly reduced proportions and absolute numbers of CD4^+^RORγt^+^ cells, total ILC3s, and specifically DN ILC3s (Fig. 4d, e and Supplementary Fig. 19a, b). However, no significant changes were noted in NKp46^+^ ILC3s or CCR6^+^ ILC3s (Supplementary Fig. 19c). The proportions and absolute numbers of intestinal ILC1s and ILC2s remained largely unchanged between two groups (Supplementary Fig. 19d–g), indicating that the decrease in ILC3 after NEC induction was not caused by ILC subset conversion. Cytokine secretion assays revealed that *Atg5* deficiency impaired IL-17A and IL-22 expression by CD4^+^RORγt^+^, total ILC3, and DN ILC3 cells (Fig. 4f–i and Supplementary Fig. 19h), with no changes occurred in NKp46^+^ ILC3s or CCR6^+^ ILC3s (Supplementary Fig. 19i, j). Notably, although both CD4^+^RORγt^+^ cells and DN ILC3s showed reductions in the proportions, absolute numbers, and cytokine secretions, the effect was more pronounced in DN ILC3s. These results suggest that NEC disease remission in *Atg5* deficiency is primarily linked to DN ILC3s.

To assess the specificity of DN ILC3s for NEC under autophagy-deficient conditions, we transferred these cells from control or *Atg5*-deficient mice into *Nfil3* KO mice (which lack ILCs) and induced NEC (Fig. 4j). Recipients of *Atg5*-deficient DN ILC3s showed reduced weight loss (Fig. 4k, l) and decreased donor-derived DN ILC3 numbers than the controls (Fig. 4m, n), with no effect on NKp46⁺ ILC3s or CCR6⁺ ILC3s (Supplementary Fig. 20a, b). These findings confirm the essential role of Atg5-dependent autophagy in DN ILC3s is essential for driving NEC pathogenesis.

### Atg5 tunes DN ILC3 glycolipid metabolism during NEC onset

**Fig. 5:**
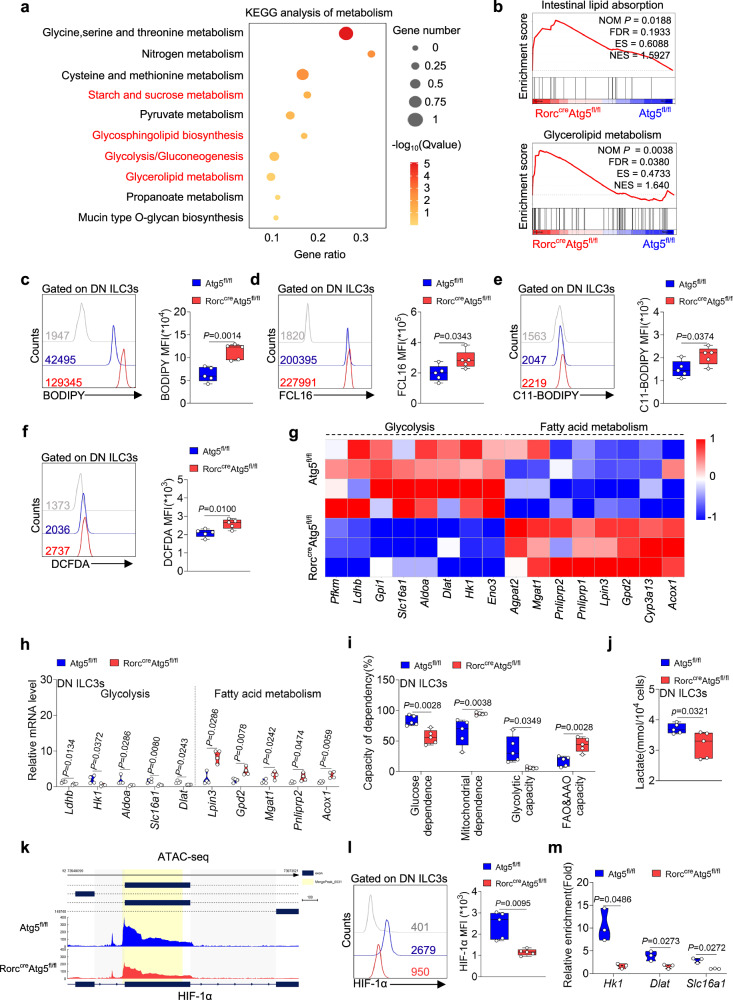
ATG5 regulates glycolipid metabolic homeostasis in intestinal DN ILC3s. **a** KEGG analysis showing metabolic pathway enrichment in intestinal ILC3s from Atg5^fl/fl^ and Rorc^cre^Atg5^fl/fl^ neonates. **b** GSEA displaying upregulation of intestinal lipid absorption and glycerolipid metabolism in intestinal ILC3s from Rorc^cre^Atg5^fl/fl^ mice. GSEA was performed on bulk RNA-seq data using 1000 one-sided phenotype-based permutation tests. Genes were ranked by signal-to-noise ratio, and multiple comparisons were adjusted via Benjamini-Hochberg FDR calculation. Significance was defined as nominal *P* < 0.05 and FDR < 0.25. Representative histogram (left) and statistical analysis (right) of BODIPY (**c**); FCL16 (**d**); C11-BODIPY (**e**); and DCFDA (**f**) staining in intestinal DN ILC3s (*n*  =  5 biological replicates per group). **g** Heatmap showing differentially expressed genes related to glycolysis and fatty acid metabolism in intestinal DN ILC3s from Atg5^fl/fl^ versus Rorc^cre^Atg5^fl/fl^ neonates. **h** Relative mRNA levels of the indicated genes in intestinal DN ILC3s (*n*  =  4 biological replicates per group). **i** Glucose dependence, mitochondrial dependence, glycolytic capacity, and fatty acid oxidation (FAO) and amino acid oxidation (AAO) in intestinal DN ILC3s (*n*  =  5 biological replicates per group). **j** Lactate concentrations in intestinal DN ILC3s (*n*  =  5 biological replicates per group). **k** Assay for Transposase-Accessible Chromatin with high-throughput sequencing (ATAC-seq) tracks at the HIF-1α locus in intestinal DN ILC3s from Atg5^fl/fl^ versus Rorc^cre^Atg5^fl/fl^ neonates. Top track shows gene annotations, with exons in black and merged peaks in yellow. **l** Representative histogram and statistical analysis of HIF-1α expression in intestinal DN ILC3s (*n*  =  5 biological replicates per group). **m** Relative enrichment of HIF-1α binding to *Hk1*, *Dlat*, and *Slc16a1* genes (*n*  =  3 biological replicates per group). All experiments were performed using Atg5^fl/fl^ and Rorc^cre^Atg5^fl/fl^ mice of both sexes at P8, with littermates randomly assigned to control and experimental groups. Each data point represents one biologically independent mouse, and results are representative of at least three independent experiments. Box plots show the median (center line, 50th percentile), with the lower and upper bounds of the box representing the 25th and 75th percentiles, respectively. Whiskers extend to the absolute minimum and maximum values (0th and 100th percentiles, respectively) of the dataset. *P*-values were determined by unpaired two tailed Student’s t tests (**c**–**f**, **h**–**j**, **l** and **m**). Source data are provided as a Source Data file.

To elucidate the role of Atg5 in regulating DN ILC3 function during intestinal inflammation in neonatal mice, we conducted transcriptome analysis of intestinal DN ILC3s from NEC-induced control (Atg5^fl/fl^) and *Atg5*-deficient (Rorc^cre^Atg5^fl/fl^) mice. The top 10 enriched metabolic pathways according to KEGG analysis indicated that *Atg5*-deficient DN ILC3s were significantly associated with glycolipid metabolism (Fig. 5a). GSEA further revealed upregulation of genes related to intestinal lipid uptake and increased glycerolipid metabolism in *Atg5*-deficient DN ILC3s (Fig. 5b). We next used the neutral lipid-specific dye BODIPY to label lipid droplets and quantified using flow cytometry. *Atg5*-deficient DN ILC3s exhibited significantly increase lipid accumulation compared with control DN ILC3s (Fig. 5c). FCL16 data confirmed enhanced lipid uptake capacity in *Atg5*-deficient DN ILC3s (Fig. 5d) and elevated lipid peroxidation levels (Fig. 5e), leading to an overall increase in total intracellular reactive oxygen species (ROS) levels (Fig. 5f). Atg5 deletion significantly reshaped the metabolic profiles of DN ILC3s, resulting in downregulation of glucose metabolism genes and upregulation of lipid metabolism genes (Fig. 5g). Quantitative real-time PCR (qPCR) analysis confirmed the downregulation of glycolysis genes *Hk1*, *Aldoa*, and *Dlat*, as well as the upregulation of lipid metabolism genes *Lpin3*, *Gpd2*, and *Acox1* in *Atg5*-deficient DN ILC3s (Fig. 5h). These observations indicate that Atg5-dependent autophagy maintains DN ILC3 metabolic homeostasis in NEC by promoting glycolysis and suppressing lipid metabolism.

To investigate whether *Atg5* deficiency reshapes the metabolic landscape of DN ILC3s during NEC, we re-analyzed scRNA-seq data from CON and NEC mice. GSEA revealed that glycolytic activity remained largely unchanged in DN ILC3s following NEC induction, whereas both the tricarboxylic acid (TCA) cycle and OXPHOS pathways were significantly downregulated (Supplementary Fig. 21a–c). Heatmap of DN ILC3s showed that glycolysis-related genes (e.g., *Hk1*, *Hk2*, *Glut1*) were upregulated in NEC mice, whereas genes associated with the TCA cycle and OXPHOS (e.g., *Pdk1*, *Slc25a14*, *Sdhc*, *Ndufv1*, *Ndufv2*, *Atp5b*) were downregulated (Supplementary Fig. 21d). To validate these findings, we sorted DN ILC3s and performed transcriptional analysis; the results were highly consistent with the scRNA-seq results. Compared with homeostatic conditions, DN ILC3s under NEC conditions exhibited a pronounced metabolic shift characterized by significant upregulation of glycolytic genes (*Hk1*, *Glut1*, and *Glut3*) and marked downregulation of genes involved in the TCA cycle and OXPHOS-related genes (Supplementary Fig. 21e). Consistently, protein levels of GLUT1 and GLUT3 were also significantly increased in DN ILC3s following NEC induction (Supplementary Fig. 21f, g). Enzyme-linked immunosorbent assay (ELISA) further confirmed elevated intracellular lactate contents in the DN ILC3s of NEC mice, indicating enhanced glycolytic capacity (Supplementary Fig. 21h). Based on these in vivo observations, we next performed in vitro intervention assays to identify the primary energy source driving this glycolytic activity. Our results confirmed that NEC induction drives a glycolytic shift in DN ILC3s, with glucose serving as the core metabolic substrate for DN ILC3 glycolysis, whereas lactate, as a secondary metabolite, exerts only a modest regulatory effect (Supplementary Fig. 21i, j).

To validate the metabolic alterations in *Atg5*-deficient DN ILC3s, we performed single cell energetic metabolism by profiling translation inhibition (SCENITH) analysis. Intestinal DN ILC3s from Rorc^cre^Atg5^fl/fl^ mice showed marked reductions in glucose dependence and glycolytic capacity, but significantly enhanced mitochondrial dependence and FAO (Fig. 5i). GSEA revealed no significant differences in OXPHOS between groups (Supplementary Fig. 22a, b). Consistently, lactate levels were significantly reduced in *Atg5*-deficient DN ILC3s (Fig. 5j). These results confirm that *Atg5* deficiency drives a metabolic switch from glycolysis toward lipid metabolism in DN ILC3s during NEC. Notably, this *Atg5*-dependent metabolic phenotype was tissue-specific: lung DN ILC3s exhibited increased glucose dependence and impaired FAO, whereas DN ILC3s from the liver, kidney, and spleen remained unchanged (Supplementary Fig. 23a). To dissect the regulatory mechanism underlying this glucose–lipid metabolic switch, we performed the Assay for Transposase-Accessible Chromatin Sequencing (ATAC-seq) on intestinal DN ILC3s. *Atg5* deficiency selectively altered chromatin accessibility at the promoter regions of key metabolic transcription factors (Supplementary Fig. 24a). Specifically, chromatin accessibility of the glycolytic regulators HIF-1α and Mlxip was markedly decreased, whereas that of Myc was unaltered (Fig. 5k and Supplementary Fig. 24b). Consistent with these changes, HIF-1α protein levels were significantly reduced in *Atg5*-deficient DN ILC3s, whereas MYC and MLXIP were comparable between the two genotypes (Fig. 5l and Supplementary Fig. 24c, d). Functionally, *Atg5* deficiency significantly decreased HIF-1α binding affinity to the promoter regions of glycolysis-associated genes *Hk1*, *Dlat*, and *Slc16a1* (Fig. 5m). To examine whether this metabolic regulation is specific to NEC, we employed a *C. rodentium*-induced infectious colitis model in adult mice. Compared with controls, *C. rodentium* infection significantly upregulated glycolysis-related genes in intestinal DN ILC3s from Atg5^fl/fl^ mice, but did not affect lipid metabolism-related genes. Moreover, no significant difference in metabolic gene expression were observed in DN ILC3s between *C. rodentium*-infected Rorc^cre^Atg5^fl/fl^ and Atg5^fl/fl^ littermates (Supplementary Fig. 25a). Collectively, these findings demonstrate that Atg5 governs glycolipid metabolism in DN ILC3s specifically in the context of NEC-induced intestinal inflammation.

### Metabolic disruption exacerbates NEC in Atg5-deficient mice

**Fig. 6:**
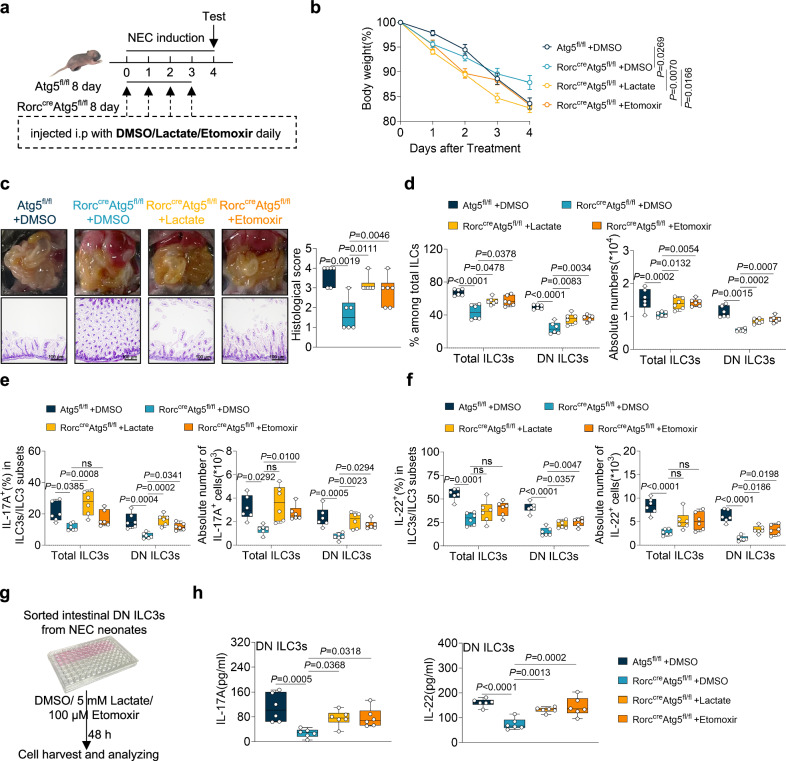
Disruption of intestinal DN ILC3 glycolipid metabolic balance exacerbates NEC in Rorc^cre^Atg5^fl/fl^ mice. **a** Schematic diagram of metabolic interventions during NEC induction. **b** Body weight changes in the indicated groups (*n*  =  6 biological replicates per group). Data are presented as mean ± SEM. **c** Macroscopic image and H&E staining of the intestines (scale bar: 100 μm), with corresponding inflammation scores (*n*  =  6 biological replicates per group). Percentage and absolute number of intestinal total ILC3s and DN ILC3s (*n*  =  6 biological replicates per group) (**d**); intestinal IL-17A^+^ total ILC3s and IL-17A^+^ DN ILC3s (*n*  =  6 biological replicates per group) (**e**); and intestinal total IL-22^+^ ILC3s and IL-22^+^ DN ILC3s (*n*  =  6 biological replicates per group) (**f**). **g** Sorted intestinal DN ILC3s from NEC neonates were treated with dimethyl sulfoxide (DMSO), lactate, or etomoxir for 48 h. **h** Levels of IL-17A and IL-22 in intestinal DN ILC3s following treatments (*n*  =  6 biological replicates per group). All experiments were performed using Atg5^fl/fl^ and Rorc^cre^Atg5^fl/fl^ mice of both sexes at P8, with littermates randomly assigned to control and experimental groups. Each data point represents one biologically independent mouse, and results are representative of at least three independent experiments. Box plots show the median (center line, 50th percentile), with the lower and upper bounds of the box representing the 25th and 75th percentiles, respectively. Whiskers extend to the absolute minimum and maximum values (0th and 100th percentiles, respectively) of the dataset. *P*-values were determined by one-way ANOVA followed by Tukey-Kramer multiple comparisons test (**b**–**f**, **h**). ns = not significant. Source data are provided as a Source Data file.

To investigate whether metabolic reprogramming contributes to the protective phenotype observed in Rorc^cre^Atg5^fl/fl^ mice during NEC, we first performed control experiments in Atg5^fl/fl^ mice treated with DMSO, lactate, or etomoxir (an inhibitor of FAO) (Supplementary Fig. 26a). Neither lactate nor etomoxir alone significantly altered NEC severity, body weight, or ILC3 abundance in Atg5^fl/fl^ mice (Supplementary Fig. 26b–e). We therefore used DMSO-treated Atg5^fl/fl^ mice as the control group for all subsequent comparisons. Following NEC induction, Rorc^cre^Atg5^fl/fl^ mice exhibited a significant protective phenotype with better body weight maintenance compared to Atg5^fl/fl^ mice. We then treated Rorc^cre^Atg5^fl/fl^ mice with either lactate or etomoxir (Fig. 6a), and found that both treatments abrogated this protective effect and exacerbated weight loss in these knockout mice (Fig. 6b, c). This aggravation was accompanied by selective expansion of ILC3s, particularly DN ILC3s, with no significant changes observed in CD4⁺RORγt⁺ cells, CCR6⁺ ILC3s, or NKp46⁺ ILC3s (Fig. 6d and Supplementary Fig. 27a–d). Functionally, lactate or etomoxir treatment significantly increased the proportions and absolute numbers of IL-17A⁺ and IL-22⁺ cells among total ILC3s and DN ILC3s (Fig. 6e, f and Supplementary Fig. 27e), whereas cytokine production by other ILC3 subsets, including the CD4⁺RORγt⁺ cells, remained unchanged (Supplementary Fig. 27f–i). Collectively, these results suggest that disrupting metabolic processes in ILC3s within Rorc^cre^Atg5^fl/fl^ mice aggravates NEC progression.

To verify whether metabolic interventions directly affect *Atg5*-deficient DN ILC3 function, we isolated DN ILC3s from the NEC-induced Rorc^cre^Atg5^fl/fl^ and Atg5^fl/fl^ mice and cultured them ex vivo with lactate or etomoxir for 48 h (Fig. 6g). Lactate or etomoxir treatment significantly increased IL-17A and IL-22 production by *Atg5*-deficient DN ILC3s, although cytokine levels remained significantly lower than those of Atg5^fl/fl^-derived DN ILC3s (Fig. 6h). These findings demonstrate that metabolic interventions can partially restore the function of *Atg5*-deficient DN ILC3s, further underscoring the importance of Atg5-dependent metabolism in NEC.

### PC inhibits DN ILC3 function and reshapes microbiota in NEC

Non-targeted lipidomics analysis was performed to investigate the potential association between intestinal inflammation relief and lipid metabolites in *Atg5*-deficient mice. Among the identified lipid metabolites, phosphatidylcholine (PC) levels were elevated in *Atg5*-deficient DN ILC3s (Supplementary Fig. 28a). RNA sequencing data from and validations of DN ILC3s from Atg5^fl/fl^ and Rorc^cre^Atg5^fl/fl^ mice showed upregulation of glycerophospholipid metabolism-related genes *Gpat3*, *Pcyt1a*, and *Cds1* (Supplementary Fig. 28b, c). In addition, PC levels were significantly increased in DN ILC3s of Rorc^cre^Atg5^fl/fl^ mice after NEC induction (Supplementary Fig. 28d). These results suggest that PC accumulation may contribute to the protective phenotype observed in *Atg5*-deficient mice during NEC pathogenesis.

**Fig. 7:**
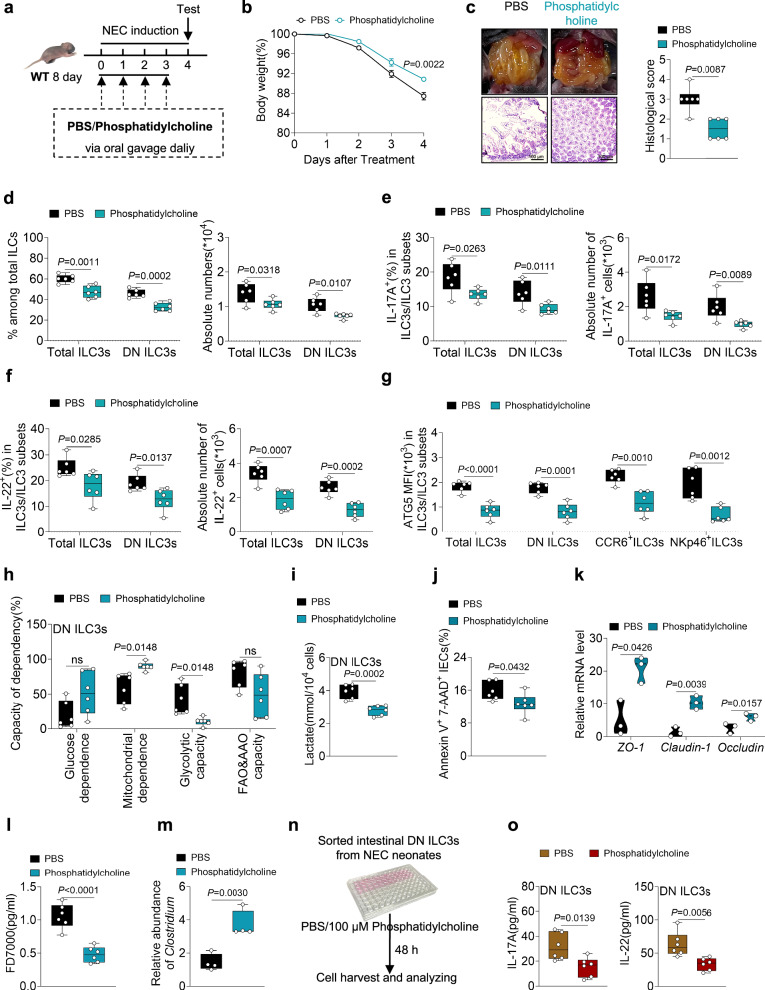
Phosphatidylcholine supplementation alleviates NEC by inhibiting intestinal DN ILC3 glycolysis and pathogenicity. **a** Schematic diagram of phosphatidylcholine (PC) supplementation during NEC induction. **b** Body weight changes in the indicated groups (*n*  =  6 biological replicates per group). Data are presented as mean ± SEM. **c** Macroscopic image and H&E staining of the intestines (scale bar: 100 μm), with corresponding inflammation scores (*n*  =  6 biological replicates per group). Percentage and absolute number of intestinal total ILC3s and DN ILC3s (*n*  =  6 biological replicates per group) (**d**); intestinal IL-17A^+^ total ILC3s and IL-17A^+^ DN ILC3s (*n*  =  6 biological replicates per group) (**e**); and intestinal IL-22^+^ total ILC3s and IL-22^+^ DN ILC3s (*n*  =  6 biological replicates per group) (**f**). **g** ATG5 expression in intestinal total ILC3s and ILC3 subsets (*n*  =  6 biological replicates per group). **h** Glucose dependence, mitochondrial dependence, glycolytic capacity, and FAO and AAO in intestinal DN ILC3s (*n*  =  6 biological replicates per group). **i** Lactate concentrations in intestinal DN ILC3s (*n*  =  6 biological replicates per group). **j** Statistical analysis of Annexin V^+^7-AAD^+^ IECs (*n*  =  6 biological replicates per group). **k** Relative mRNA levels of *ZO-1*, *Claudin-1*, and *Occludin* in the intestines (*n*  =  3 biological replicates per group). **l** Intestine permeability was assessed by plasma FD7000 concentrations (*n*  =  6 biological replicates per group). **m** Relative abundance of *Clostridium* in fecal samples (*n*  =  4 biological replicates per group). **n** Sorted intestinal DN ILC3s from NEC neonates were treated with either phosphate buffered saline (PBS) or PC for 48 h. **o** Levels of IL-17A and IL-22 in intestinal DN ILC3s following treatments (*n*  =  6 biological replicates per group). All experiments were performed using C57BL/6 mice of both sexes at P8, with littermates randomly assigned to control and experimental groups. Each data point represents one biologically independent mouse, and results are representative of at least three independent experiments. Box plots show the median (center line, 50th percentile), with the lower and upper bounds of the box representing the 25th and 75th percentiles, respectively. Whiskers extend to the absolute minimum and maximum values (0th and 100th percentiles, respectively) of the dataset. *P*-values were determined by unpaired two tailed Student’s t tests (**b**–**m** and **o**). ns = not significant. Source data are provided as a Source Data file.

To validate the functional role of PC in NEC progression, 40 mg/kg PC was added to formula milk before NEC induction in mice (Fig. 7a). Compared with PBS‑treated controls, PC supplementation significantly alleviated weight loss and reduced disease severity in NEC mice (Fig. 7b, c). The proportion and absolute number of ILC3s, particularly DN ILC3s, were decreased, whereas those of CD4^+^RORγt^+^, NKp46^+^ ILC3, and CCR6^+^ ILC3 cells showed no significant differences (Fig. 7d and Supplementary Fig. 29a–d). We then assessed ILC3 function and found that IL-17A and IL-22 levels were significantly reduced following PC supplementation, especially in DN ILC3s (Fig. 7e, f and Supplementary Fig. 29e). In contrast, no significant differences were observed in this functionality of CD4^+^RORγt^+^, NKp46^+^ ILC3, or CCR6^+^ ILC3 cells (Supplementary Fig. 29f–i). PC supplementation also significantly inhibited ATG5 expression in ILC3s and their subpopulations (Fig. 7g). Moreover, PC treatment significantly reduced glycolysis dependence of DN ILC3s, while increasing their dependence on mitochondrial respiration (Fig. 7h). Consistent with this metabolic shift, lactate levels in DN ILC3s were significantly lower in the PC‑treated group than in the PBS‑treated control group (Fig. 7i). Although PC intervention had no significant effect on the absolute number or proliferation of IECs, it inhibited IEC apoptosis and upregulated the expression of intestinal barrier-related genes (Fig. 7j, k and Supplementary Fig. 29j–l). Decreased intestinal permeability confirmed these observations (Fig. 7l). Next, we investigated the effects of PC intervention on the gut microbiota of NEC mice. Principal coordinate analysis revealed a clear separation of the overall gut microbiota community structure between PC-treated and control groups (Supplementary Fig. 30a). Specifically, PC treatment substantially reshaped the gut microbiota composition at the phylum and class level, with a particularly marked increase in the relative abundance of *Clostridia* (Fig. 7m and Supplementary Fig. 30b–d).

The impact of PC intervention on the functionality of DN ILC3 derived from neonatal intestines was further examined. We isolated intestinal DN ILC3s from the NEC-induced mice and co-cultured them with PC for 48 h (Fig. 7n). PC treatment significantly inhibited IL-17A and IL-22 secretion by DN ILC3s (Fig. 7o). Collectively, these results imply that PC alleviates NEC-associated intestinal inflammation by modulating the Atg5–DN ILC3 axis, remodeling cellular metabolism and gut microbiota, and protecting the intestinal barrier.

### Human NEC tissues show enhanced ILC3 levels and autophagy

**Fig. 8:**
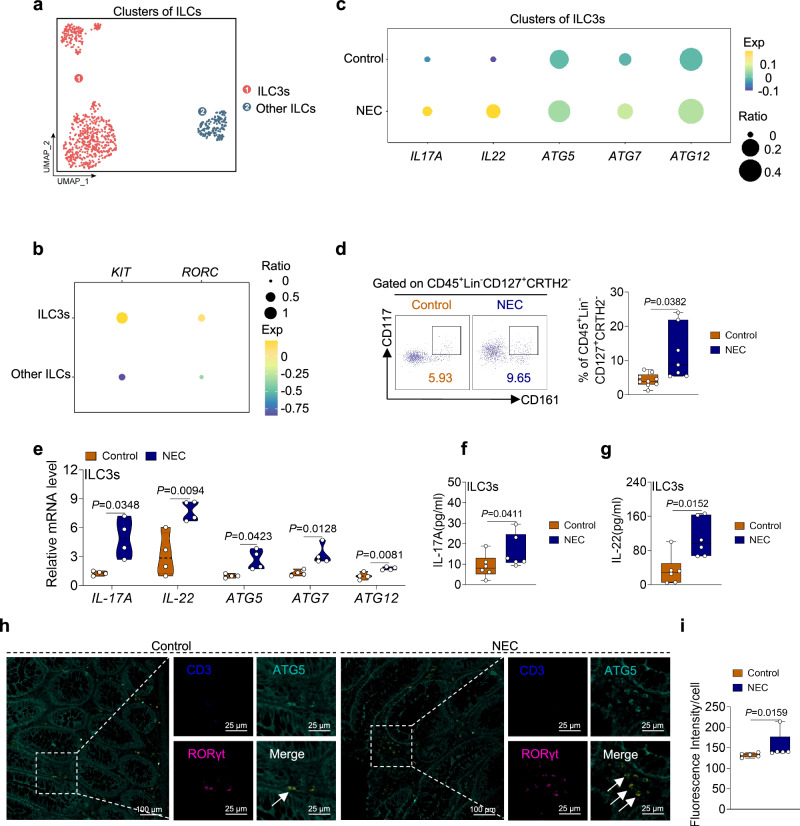
Enhanced intestinal ILC3 function and autophagy in neonatal patients with NEC. **a** Uniform manifold approximation and projection (UMAP) plot showing the ILC3 and other ILC clusters. **b** Bubble plot of *KIT* and *RORC* expression in these clusters. **c** Average expression levels of *IL-17A*, *IL-22*, *ATG5*, *ATG7*, and *ATG12* in the ILC3 cluster. **d** Representative flow cytometry profiles and statistical analysis of intestinal ILC3s in controls and patients with NEC (*n*  =  9 and 7). **e** Relative mRNA levels of *IL-17A*, *IL-22*, *ATG5*, *ATG7*, and *ATG12* in human intestinal ILC3s (*n*  =  4 biological replicates per group). Levels of IL-17A (**f**) and IL-22 (**g**) in human intestinal ILC3s (*n*  =  6 biological replicates per group). **h** Immunofluorescence staining of the intestines from controls and patients with NEC. Scale bars: 100 µm for the low-magnification overview (left panel); 25 µm for the high-magnification insets (right panels). CD3 (blue), RORγt (magenta), and ATG5 (teal). **i** Quantification of fluorescence intensity per cell in (**h**). Each symbol represents an individual, and data represent at least three independent experiments. Box plots show the median (center line, 50th percentile), with the lower and upper bounds of the box representing the 25th and 75th percentiles, respectively. Whiskers extend to the absolute minimum and maximum values (0th and 100th percentiles, respectively) of the dataset. *P*-values were determined by unpaired two tailed Student’s t tests (**d**–**g** and **i**). Source data are provided as a Source Data file.

To verify the clinical relevance of our findings, we first reanalyzed a public single-cell transcriptomic dataset of intestinal tissues from neonatal patients with NEC (Zenodo database, accession number: 5813397). After excluding CD3⁺ T cells, the remaining cells were re-clustered, and ILCs were identified based on the signature gene expression (Supplementary Fig. 31a, b). ILC3s were specifically defined by the co-expression of the core transcription factor *RORC* and the surface marker *KIT* (Fig. 8a, b and Supplementary Fig. 31c). Consistent with our results observed in murine models, the proportion of ILC3s was higher in patients with NEC than in the controls (Supplementary Fig. 31d). ILC3s from patients with NEC also exhibited marked upregulation of both effector cytokines (*IL-17A* and *IL-22*) and autophagy-related genes (*ATG5*, *ATG7*, and *ATG12*) (Fig. 8c).

We next validated these findings using freshly collected intestinal tissue samples from patients with NEC and neonatal controls. Flow cytometry confirmed a significant increase in ILC3 proportions in patients with NEC (Fig. 8d). qPCR analysis revealed that intestinal ILC3s from NEC patients exhibited significantly higher mRNA levels of the effector cytokine genes *IL-17A* and *IL-22*, as well as the core autophagy-related genes *ATG5*, *ATG7*, and *ATG12*, compared with ILC3s from the controls (Fig. 8e). Consistently, ELISA demonstrated higher secretion of IL-17A and IL-22 from ILC3s isolated from NEC tissues relative to those from controls tissues (Fig. 8f, g). Immunofluorescence staining of intestinal tissues further confirmed that ATG5 protein expression was significantly higher in ILC3s from the NEC group compared with control group (Fig. 8h, i). To assess the specificity of autophagy activation in human intestinal ILC3s, we calculated autophagy scores across different cell populations within the NEC intestinal. Although autophagy was broadly enriched in both immune and non-immune cells, ILC3s displayed a significantly higher autophagy score than other ILC subsets and T cells (Supplementary Fig. 32a). Furthermore, subset analysis of human ILC3s based on the expression of CCR6 and NKp46 (*NCR1*) revealed that DN ILC3s (CCR6⁻NKp46⁻) constituted the dominant ILC3 population, and that DN ILC3s was significantly increased in patients with NEC (Supplementary Fig. 33a–c). Together, these data confirm that ILC3 expansion and enhanced autophagy are a defining immune signature of human neonatal NEC.

## Discussion

Emerging evidence links immune cell metabolism and autophagy to intestinal inflammation, however, the crosstalk in DN ILC3s during NEC pathogenesis remains poorly understood. In this study, we identified Atg5-mediated autophagy as a critical regulator of DN ILC3 pathogenicity in NEC. Mechanistically, we found that Atg5 sustains the HIF-1α/RORγt transcriptional program, which drives glycolytic metabolism and subsequent IL-17A production in DN ILC3s. This metabolic shift promotes intestinal epithelial necrosis and exacerbates the severity of NEC. Conversely, Atg5 deficiency attenuates HIF-1α/RORγt activity, suppresses glycolysis, and enhances FAO, leading to reduced IL-17A secretion and alleviated damage (Supplementary Fig. 34). These findings elucidate the role of autophagy in coupling of immune cell metabolism with inflammatory function in the neonatal intestine, and highlight the autophagy–energy metabolism axis as a promising therapeutic target for NEC.

Previous mouse models of NEC are characterized by increased levels of pro-inflammatory NKp46⁻RORγt⁺T-bet⁺ ILC3s and decreased levels of the protective NKp46⁺RORγt⁺ ILC3 subset^24^. Inhibition of RORγt function reduces ILC3 abundance and alleviates NEC severity^25^. In this study, we first assessed the contribution of distinct ILC3 subsets to NEC pathogenesis. Adoptive transfer experiments confirmed that only NEC-derived DN ILC3s significantly exacerbated body weight loss and intestinal inflammation, whereas NKp46⁺ ILC3s and CCR6⁺ ILC3s had no such effects. Notably, this pro-inflammatory phenotype was specific to NEC-derived DN ILC3s, as homeostatic DN ILC3s retained their physiological role in maintaining intestinal barrier stability. Our scRNA-seq profiling also revealed a marked expansion of pro-inflammatory DN ILC3s in NEC, accompanied by a striking reduction in ILC2s. Given the well-established anti-inflammatory roles of ILC2s in mucosal immunity, we further validated their function in our model and demonstrated that neonatal—but not adult—ILC2s significantly alleviated NEC. The detailed cellular and molecular mechanism underlying this age-dependent functional difference will be explored in our future studies.

The role of T cells in NEC pathogenesis has been well documented^15,42,43^. However, when we employed Rag2^–/–^ mice in our experimental system, these mice remained susceptible to NEC. This observation differs from a previous report demonstrating that Rag1^–/–^ mice were resistant to NEC induction, suggesting potential functional differences between the two models. To investigate this discrepancy, we re-examined the most relevant study, which employed a similar induction protocol^16^. The key methodological difference was the use of a bacterial product cocktail derived from the intestinal tracts of NEC patients, which was added to the milk formula. However, critical details—including disease severity of donor patients, as well as the exact composition and dosage of the bacterial products—were not reported, precluding direct comparison. Consistent with previous reports, we observed that CD4⁻RORγt⁺ cells were more than 8-fold more abundant than CD4⁺RORγt⁺ T cells in neonatal NEC^16^. This marked abundance, together with the susceptibility of Rag2^–/–^ mice to NEC, supports a predominant role for DN ILC3s in driving NEC pathogenesis in our model. The divergent outcomes between Rag1^–/–^ and Rag2^–/–^ models highlight the importance of model system considerations when interpreting immune contributions to this disease.

To further assess the functional contribution of T cells versus DN ILC3s to NEC pathogenesis, we performed adoptive transfer experiments using Rag2^–/–^ recipient mice. Transfer of CD4⁺ T cells at a dose equivalent to that used for DN ILC3s (3 × 10^4^ cells) failed to exacerbate NEC pathology. In contrast, a substantially higher number of CD4⁺ T cells (4 × 10^6^ cells) were required to achieve a pathogenic effect comparable to that of the lower dose DN ILC3s. This dose-dependent effect may reflect the opposing roles of CD4⁺ T cell subsets in NEC pathogenesis. Previous studies have shown that the NEC environment suppresses Tregs while expanding pathogenic Th17 cells, suggesting a functional equilibrium between these subsets^16^. At low transfer numbers, these opposing forces may counterbalance, resulting in no net effect. However, at high transfer numbers, this equilibrium may shift, allowing pathogenic Th17 subsets to predominate and promote disease progression. These findings delineate a functional hierarchy in NEC pathogenesis: while T cells can contribute when present in sufficient numbers, DN ILC3s appear to act as key early drivers of NEC, consistent with their marked abundance in neonatal NEC. Elucidating the distinct contributions of these two populations is essential for developing subset-specific therapeutic interventions.

Previous studies have reported elevated IL-17 and IL-22 expression in mouse NEC intestinal tissues, while loss of NKp46⁺ ILC3s has been shown to reduce IL-22 expression^17^. Additionally, intestinal epithelial cell-specific gene knockout has also been implicated in regulating IL-22 levels^44^. To identify the key cytokines mediating the pathogenic role of DN ILC3s, we re-analyzed our scRNA-seq data and validated findings by quantifying cytokine production in vitro. Integrated analysis consistently showed that IL-17A—not IL-22 or GM-CSF—was significantly upregulated in DN ILC3s after NEC induction. Moreover, only IL-17A neutralization—not IL-22 or GM-CSF neutralization—significantly reduced disease severity in murine NEC models, confirming the central role of IL-17A in NEC progression. These observations align with Mihi et al., who found no increase in intestinal IL-22 after NEC induction and no increased NEC susceptibility in *Il-22*-deficient mice^45^. However, our IL-22 neutralization results differ from Zhang et al., who reported worsened inflammation with IL-22 blockade^44^. This discrepancy is likely due to the differences in the frequency of antibody application: they administered anti-IL-22 antibody continuously over four days, whereas we used two injections at the same dose, in line with our published protocol^46^—suggesting sustained neutralization may be required for effective IL-22 suppression, while transient blockade may be insufficient during active inflammation. These findings highlight dosing strategy as a key variable in interpreting cytokine neutralization data, and further implicate IL-17A as a promising therapeutic target for NEC.

We identified enhanced autophagy in DN ILC3s as a key feature of NEC through scRNA-seq data analysis. Initial pharmacological experiments showed that 3-MA attenuated disease while RAP exacerbated it, correlating with reciprocal changes in ILC3 numbers—effects confined to the DN ILC3 subset. However, given the well-documented off-target effects of 3-MA and RAP^47,48^, genetic validation was required. We therefore generated mice with conditional *Atg5* deletion in RORγt⁺ cells (Rorc^cre^Atg5^fl/fl^) and performed adoptive transfer into *Nfil3*^–/–^ recipients, confirming that Atg5-mediated autophagy in DN ILC3s drives NEC pathogenesis. Notably, the non-specificity of 3-MA and RAP limits their translational potential, highlighting the need for small molecules that selectively target autophagy in DN ILC3s as a promising therapeutic avenue for NEC.

Metabolic reprogramming is a central mechanism underlying the autophagy-dependent pathogenicity of DN ILC3s in NEC. Compared with homeostasis, DN ILC3s from NEC mice exhibited upregulated glycolysis-related genes and downregulated TCA cycle and OXPHOS-related genes, indicating a shift toward glycolysis. Notably, Atg5 deletion reversed this shift: glycolysis decreased while lipid metabolism increased. Thus, Atg5-dependent autophagy sustains glycolysis and suppresses lipid metabolism in DN ILC3s during NEC. Importantly, this autophagy–metabolism axis is NEC-specific—it was absent in IBD models—highlighting a critical, context-dependent role for autophagy–energy metabolism crosstalk in regulating DN ILC3 function specifically in NEC. Transcriptomic and metabolomic analyses revealed PC as a key downstream metabolite of Atg5 mediated autophagy, and we further indicated that PC supplementation inhibited DN ILC3 function, restored glycolytic homeostasis, reshaped the gut microbiota and ameliorated NEC pathology in mice. These findings suggest PC might be a key downstream effector of the DN ILC3 autophagy–metabolism axis and a promising therapeutic avenue for NEC. Identifying the precise molecular targets and underlying mechanisms through which PC acts on DN ILC3s will be a central focus of our future research.

Our findings in human sample recapitulated our murine observations, encompassing both reanalysis of published scRNA-seq datasets and de novo analysis of intestinal tissue from NEC patients. These clinical data reveal a significant increase in ILC3 abundance and functional activation in NEC patients, accompanied by upregulated autophagic activity, thereby supporting cross-species conservation of this pathogenic mechanism. A key limitation of our clinical study is the relatively small human cohort, which reflects the well-documented challenge of obtaining high-quality, clinically well-annotated NEC specimens. Larger cohorts are therefore essential for independent validation. Collectively, these cross-species data establish the autophagy–metabolism axis as a central driver of DN ILC3-mediated NEC pathogenesis.

Targeting the autophagy–metabolism axis—via autophagy modulation, metabolic intervention, or PC supplementation—holds significant promise for the prevention of NEC. However, key translational challenges remain, including achieving cell-type-specific therapeutic modulation, defining the optimal therapeutic window in the period, and ensuring safety in neonates. Moreover, the large gap between murine models and human newborns demands rigorous evaluation of efficacy and toxicity in clinically relevant settings. Humanized mouse models and patient-derived intestinal organoids offer powerful platforms to bridge this gap and advance precision therapeutics for NEC.

## Methods

### Human samples

Fresh small intestinal tissues were obtained from surgical resections in neonatal patients at the Department of Pediatric Surgery, Zhujiang Hospital, Southern Medical University (Guangzhou, China). Human intestinal samples were collected from neonates undergoing resection for NEC (at the time of stoma closure) and from non-NEC surgical controls. Control tissues were obtained during stoma closure surgery in the absence of active intestinal disease or history of NEC at the of sampling, whose primary diagnoses including meconium plug/ileus, spontaneous intestinal perforation, Hirschsprung disease (unaffected tissue used), duodenal atresia, and intestinal pseudo-obstruction. The clinical characteristics are summarized in Supplementary Table 1. The study protocol was approved by the Medical Ethics Committee of Zhujiang Hospital, Southern Medical University (Approval Number: 2023-KY-035-01). Written informed consent was obtained from the legal guardians of all participants, including explicit consent for the publication of de-identified clinical information that may potentially contain indirect identifiers of individual participants.

### Mice

C57BL/6 mice (eight-day-old) were obtained from the Experimental Animal Center of Southern Medical University (Guangzhou, China). Atg5^fl/fl^ mice were provided by Professor Zhexiong Lian (Guangdong Academy of Medical Sciences, Guangzhou, China)^49^, with genotyping confirmation. Rorc^cre^ mice were provided by Professor Chen Dong (Tsinghua University)^50^. Rag2^–/–^ mice (stock no.C000115) with a C.B6(Cg)-Ragtm1.1Cgn/J background and *Nfil3*^–/–^ mice (stock no. NM-KO-190125) with a C57BL/6JCya-Nfil3em1/Cya background were purchased from Changzhou Cavens Laboratory Animal Co., Ltd. (Changzhou, China) and Shanghai Model Organisms Center, Inc. (Shanghai, China), respectively. All mice were housed in a specific pathogen-free (SPF) barrier animal facility under a controlled 12-h light/12-h dark cycle, constant temperature (22 ± 2 °C) and relative humidity (50 ± 10%), with ad libitum access to sterile commercial chow and autoclaved drinking water. Age- and sex-matched littermates were used for all experiments. For experiments comparing healthy breastfed control mice and NEC model experimental mice, the two groups were housed separately to avoid interference from breastfeeding dams with the NEC modeling procedure, while maintaining identical environmental parameters between groups. For experiments comparing different intervention groups within the NEC model (control and experimental groups both subjected to NEC induction), mice were randomly assigned to groups after modeling and co-housed to eliminate cage effects. All animal experimental protocols were approved by the Institutional Animal Care and Use Committee of Southern Medical University (Approval Number: L2023083).

### Reagents and antibodies

The reagents and antibodies used in this study are provided in Supplementary Table 2 and 3.

### NEC model establishment

Experimental NEC models were induced in eight-day-old pups via gavage feeding of formula milk (four times daily). The formula milk contains a 2:1 mixture of Similac Advance infant formula (Abbott Nutrition, Columbus, OH, USA) and Esbilac puppy milk replacer (PetAg, Hampshire, IL, USA). Mice were subjected to gentle abdominal massage to facilitate urination prior to each gavage and stressed twice daily by hypoxia (5% oxygen plus 95% nitrogen for 2 min) and cold stress (4 °C for 10 min) for four days. For vivo interventions, the autophagy inhibitor 3-methyladenine (30 mg/kg/day; Selleck, Cat#: S2767) or induced rapamycin (1 mg/kg/day; Selleck, S1039) was administered intraperitoneally once daily during NEC induction. Sodium lactate (1 g/kg/day; Macklin, Cat#: S817836) and the FAO inhibitor etomoxir (30 mg/kg/day; Selleck, Cat#: E4787) were administered intraperitoneally once daily during NEC induction to assess the effect of energy metabolism on intestinal inflammation in Rorc^cre^Atg5^fl/fl^ mice. PC (40 mg/kg; Aladdin, Cat#: 8002-43-5) was supplemented into the formula milk via oral gavage during NEC induction. After a 4-day induction, all mice were first deeply anesthetized by inhalation of 5% isoflurane, followed by cervical dislocation to ensure rapid and painless euthanasia, performed by trained personnel.

### Neutralizing antibodies in vivo intervention

Eight-day-old neonates were treated intraperitoneally with 5 mg/kg anti-IL-17A (Bio X Cell; Clone 17F3; Cat#: BE0173) or mouse IgG1 isotype control antibody (Bio X Cell; Clone MOPC-21; Cat#: BE0083) at day 0 and day 2 before and during NEC induction. Similarly, 5 mg/kg anti-IL-22 (eBioscience; Clone IL22JOP; Cat#:16-7222-85) or rat IgG2a isotype control antibody (eBioscience; Clone eBR2a; Cat#:16-4321-85) was administered intraperitoneally at day 0 and day 2 before and during NEC. Additionally, 5 mg/kg anti-GM-CSF (Bio X cell; Clone MP1-22E9; Cat#: BE0259) or rat IgG2a isotype control antibody (Bio X cell; Clone 3A2; Cat#: BE0089) was injected intraperitoneally at day 0 and day 2 before and during NEC. All mice were euthanized on day 5 for further analysis.

### Histopathological analysis

Intestinal tissues were dissected, perfused with ice-cold phosphate buffered saline (PBS) to reduce blood cell interference, then immediately fixed in 4% phosphate-buffered formaldehyde solution for at least 24 h. Tissues were embedded in paraffin and sliced into 4-μm sections. Paraffin-embedded sections were stained with hematoxylin and eosin and visualized under a light microscope (Nikon Eclipse 80i, Japan) to evaluate immune cell infiltration, intestinal vacuolization, and tissue integrity. Histological scoring was performed as previously described^51^.

### Intestinal permeability

FITC-labeled dextran (FD7000, Sigma-Aldrich, Cat#: FD70-1G) was used to evaluate intestinal permeability in mice. At the end of the NEC model, pups were administered 750 mg/kg FD7000 (molecular mass 73,000 Da) suspended in PBS. Mice were euthanized 4 h later, and plasma was collected for FD7000 quantification using fluorescence spectrophotometry.

### Isolation of lamina propria mononuclear cells

Mouse and human intestinal samples were washed with ice-cold PBS, cut longitudinally, cleaned thoroughly, and mechanically minced into 1-cm pieces with scissors. Intestinal fragments were transferred into Hank’s Balanced Salt Solution (HBSS) buffer containing 10 mM EDTA (XPBiomed, Cat#: C3530-0100) and 1 mM dithiothreitol (DTT, Amresco; Cat#: MS5511) and incubated for 30 min at 37 °C on an orbital shaker to remove epithelial cells and mucus. After vertexing and washing twice with PBS, the epithelial fraction was discarded. The remaining tissues were minced into 1-mm pieces and digested in Roswell Park Memorial Institute (RPMI)-1640 medium (BI, Cat#: 01-100-1ACS) supplemented with 5% fetal bovine serum (FBS; BI, Cat#: 04-001-1ACS), 1 mg/mL collagenase I (Gibco, Cat#: 17104019), 100 μg/mL DNase I (Sangon Biotech, Cat#: B002138-0025), 1 mg/mL dispase (Roche, Cat#: 04942078001), and 10 mM HEPES (Beyotime, Cat#: ST092), with incubation for 45 min at 37 °C on an orbital shaker. Digested tissues were filtered through a 70-μm cell strainer, and the cell suspension was resuspended in 40% Percoll (GE Healthcare, Cat#:17-0891-09). LPMCs were enriched by 40%/80% Percoll gradient centrifugation. After centrifugation at 400 × *g* for 25 min at room temperature, the white intermediate layer was collected and washed with PBS to obtain LPMCs, which were then used for flow cytometry analysis or cell sorting.

### Flow cytometry analysis

The gating strategies for mouse and human samples are provided (Supplementary Fig. 35a–e). Mouse DN ILC3s were defined as CD45^+^CD4^−^Lin^−^CD90.2^+^CD127^+^RORγt^+^CCR6^−^NKp46^−^ cells, NKp46^+^ ILC3s were defined as CD45^+^CD4^−^Lin^−^CD90.2^+^CD127^+^RORγt^+^CCR6^−^NKp46^+^ cells, CCR6^+^ ILC3s were defined as CD45^+^CD4^−^Lin^−^CD90.2^+^CD127^+^RORγt^+^CCR6^+^NKp46^−^ cells; ILC1s were defined as CD45^+^CD4^−^Lin^−^CD90.2^+^CD127^+^T-bet^+^ cells, and ILC2s were defined as CD45^+^CD4^−^Lin^−^CD90.2^+^CD127^+^GATA3^+^ cells. The mouse lineage markers included CD3, B220, CD11b, Ly6G, Ter119, CD11c, CD5, CD8a, TCRαβ, and TCRγδ. Mouse intestinal TCRγδ T cells and TCRαβ T cells were identified as CD45^+^TCRγδ^+^ cells and CD45^+^TCRαβ^+^ cells, respectively. Mouse IECs were identified as CD45^−^EpCAM^+^ cells. Human intestine ILC3s were identified as CD45^+^Lin^−^CD127^+^CD161^+^CRTH2^−^CD117^+^ cells. The human lineage markers included CD11b, TCRγδ, TCRαβ, CD14, CD34, CD19, CD3, CD123, CD8, CD5, CD4, CD11c, and FceR1. Fluorescence-activated cell sorting (FACS) data were acquired using a CytoFLEX S flow cytometer (Beckman Coulter, Brea, CA, USA) and analyzed using FlowJo v10.0.8. Flow cytometry analysis was performed at the Department of Immunology and the Department of Developmental Biology, School of Basic Medical Sciences, Southern Medical University. The antibodies used in the study are reported in Supplementary Table 3

### Cell sorting

The sorting strategies for mouse and human samples are provided (Supplementary Fig. 36a–c). For mouse isolation, FACS was performed using a CytoFLEX SRT flow cytometer (Beckman Coulter, USA) to isolate target cell populations for subsequent experiments (scRNA-seq, RNA-seq, ATAC-seq, RT-qPCR, in vitro culture, untargeted lipidomics, and adoptive transfer). Sorted cell populations were defined as follows: DN ILC3s (CD45^+^CD4^−^CD90.2^hi^Lin^−^KLRG1^−^CCR6^−^ NKp46^−^ cells); NKp46⁺ ILC3s (CD45^+^CD4^−^CD90.2^hi^Lin^−^KLRG1^−^CCR6^−^NKp46^+^ cells); CCR6⁺ ILC3s (CD45^+^CD4^−^CD90.2^hi^Lin^−^KLRG1^−^CCR6^+^NKp46^−^ cells); ILC2s (CD45^+^CD4^−^CD90.2^+^ Lin^−^KLRG1^+^ cells); and CD4⁺ T cells (CD45⁺CD4⁺ cells). Human ILC3s were isolated by FACS using a CytoFLEX SRT flow cytometer (Beckman Coulter, USA), and were defined as CD45⁺Lin⁻CD127⁺CD117⁺CD161^+^ cells^52^. Purified cells were used for qPCR and ELISA analysis.

For immunomagnetic isolation of DN ILC3s, the EasySep Mouse Pan-ILC Enrichment Kit (StemCell, Cat#: 19875) and EasySep Mouse PE Positive Selection Kit II (StemCell, Cat#: 17656) were used according to the manufacturer’s instructions. For mouse DN ILC3 isolation, lineage-negative cells were first enriched (Pan-ILC Enrichment kit), labeled with anti-mouse CD90.2-PE antibody (eBioscience, Cat#: 12-0902-82), and purified (Mouse PE Positive Selection Kit II). After sorting, cell purity was verified using flow cytometry. The lineage markers used for mouse cell sorting included CD3, B220, CD11b, Ly6G, Ter119, CD11c, CD5, CD8a, TCRαβ, TCRγδ, and NK1.1. Purified cells were used for western blotting and transmission electron microscopy.

### Adoptive transfer

For ILC3 subset adoptive transfer, the cells (DN ILC3s, NKp46^+^ ILC3s, and CCR6^+^ ILC3s) were isolated from NEC-induced C57BL/6 mice. Prior to NEC induction (day 0), each ILC3 subset (3 × 10^4^ cells in PBS) were transferred into NEC-induced C57BL/6 mice via intraperitoneal injection. For ILC2 adoptive transfer, the cells were isolated from one-week-old and eight-week-old mice under physiological conditions. Before NEC induction (day 0), ILC2s (3 × 10^4^ cells in PBS) were transferred into NEC-induced C57BL/6 mice via intraperitoneal injection. For DN ILC3 and CD4^+^ T cell comparative transfer, the cells were isolated from NEC-induced C57BL/6 pups. Prior to NEC induction (day 0), DN ILC3s (3 × 10^4^ cells in PBS), low-dose CD4^+^ T cells (3 × 10^4^ cells in PBS), and high-dose CD4^+^ T cells (4 × 10^6^ cells in PBS) were transferred into NEC-induced Rag2^−/−^ mice via intraperitoneal injection. For *Atg5*-deficient DN ILC3 transfer, the cells were isolated from NEC-induced Atg5^fl/fl^ and Rorc^cre^Atg5^fl/fl^ mice. Before NEC induction (day 0), DN ILC3s (3 × 10^4^ cells in PBS) were transferred into NEC-induced *Nfil3*^−/−^ mice via intraperitoneal injection. All recipient mice were euthanized on post-transfer day five for further analysis.

### In vitro culture experiments

1 × 10^4^ DN ILC3s were cultured in 96-well U-bottom plates in 100 μL of complete Dulbecco’s Modified Eagle Medium (DMEM) supplemented with 10% FBS and 1% penicillin/streptomycin solution at 37 °C in a 5% CO_2_ atmosphere. The stimuli were as follows: (1) 3-MA (final concentration 10 μM) (Selleck, Cat#: S2767); (2) Rapamycin (final concentration 10 nM) (Selleck, Cat#: S1039); (3) Sodium lactate (final concentration 5 mM) (Macklin, Cat#: S817836); (4) Etomoxir (final concentration 100 μM) (Selleck, Cat#: E4787); and (5) PC (final concentration 100 μM) (Aladdin, Cat#: 8002-43-5). After 48 h, the cells were harvested for subsequent experiments.

### Carbon source utilization assay

1 ×  10^4^ DN ILC3s were cultured in 96-well U-bottom plates in 100 μL of glucose‑free complete DMEM (Biosharp, Cat#: BL1124A) supplemented with 10% FBS and 1% penicillin/streptomycin solution at 37 °C in a 5% CO_2_ atmosphere. The stimuli were as follows: (1) Glucose (final concentration 5 mM) (Aladdin, Cat#: G640146) and (2) Sodium lactate (final concentration 5 mM) (Macklin, Cat#: S817836). After 24 h, the cells were harvested for subsequent experiments.

### Quantification of lipid metabolism parameters

Briefly, 1 × 10^6^ cells were resuspended in 500 μL of RPMI-1640 medium and incubated with 2 μM C11-BODIPY (581/591; Invitrogen, Cat#: D3861), 2 μM HCS LipidTOX Deep Red neutral lipid stain (Invitrogen, Cat#: H34477), or 5 μM DCFH-DA (cytosolic ROS) (Invitrogen, Cat#: C369) for 30 min at 37 °C. Then, the cells were washed with fresh PBS and analyzed using flow cytometry at a 488 nm laser and collecting emission at 525/540 nm. Data were analyzed using FlowJo v10.0.8.

### Single-cell energetic metabolism by profiling translation inhibition analysis

Intestinal cells were seeded in 96-well plates at a density of 1 × 10^6^ cells/mL then treated with DMSO, 2-deoxy-d-glucose (50 mM, Selleck, Cat#: S4701), oligomycin (Oligo; 1 mM, Selleck, Cat#: S1478), or a combination of both for 30 min at 37 °C, 5% CO_2_. Puromycin (Puro; 10 μg/mL, MedChemExpress, Cat#: HY-B1743A) was then added, and the cells were incubated for an additional 30 min at 37 °C. The cells were then washed with ice-cold PBS and stained with fluorochrome-conjugated antibodies against surface markers for 20 min at 4 °C in staining buffer. After washing, the cells were fixed and permeabilized using Foxp3 fixation and permeabilization buffer following the manufacturer’s instructions. Intracellular staining with Puro was then performed by incubating cells with an anti-puromycin antibody (Sigma-Aldrich, Cat#: 12D10) diluted in permeabilization buffer for 45 min at 4 °C^53,54^. The cells were analyzed using flow cytometry, and data were analyzed using FlowJo v10.0.8.

### Western blot analysis

Cells were washed with cold PBS and lysed using RIPA lysis buffer containing phosphatase inhibitors (1 mM, Beyotime, Cat#: C0013B) and PMSF (1 mM, Beyotime, Cat#: C1005) on ice. The lysate was then centrifuged at 10,000 × *g* for 10 min at 4 °C to remove insoluble debris. The protein concentration of the whole cell lysates was measured using a BCA Protein Assay Kit (Beyotime, Cat#: P0010). Next, proteins were electrophoresed on SDS-PAGE gels and transferred to polyvinylidene difluoride membranes (Millipore, Bedford, MA, USA) using a wet transfer system at 100 V for 80 min. After blocking with 5% non-fat milk for 1 h, the membranes were incubated with the primary antibodies overnight at 4 °C with the following primary antibodies: ATG5 (APG5L) rabbit monoclonal antibody mAb (Zenbio, clone R06-1E5, 1:500, Cat# R381320), ULK1 rabbit mAb (Zenbio, clone R02-8E7, 1:500, Cat# R381887), LC3B rabbit mAb (Zenbio, clone R06-4K9, 1:500, Cat# R381544), and GAPDH rabbit mAb (Zenbio, clone R09-4E-1, 1:500, Cat# R380626). Membranes were then washed and incubated for 1 h at room temperature with the corresponding horseradish peroxidase (HRP)-conjugated secondary antibodies: goat anti-rabbit IgG H&L (HRP, Zenbio, 1:200, Cat# 511203) and goat anti-mouse IgG H&L (HRP, Zenbio, 1:200, Cat# 511103). Protein bands were visualized using enhanced chemiluminescent HRP substrate (Millipore, Billerica, MA, USA) with the ChemiDoc XRS+ System (Bio-Rad, Hercules, CA, USA).

### Immunofluorescence staining

Human intestinal histopathological sections of the control and NEC groups were provided by the Department of Pediatric Surgery, Zhujiang Hospital, Southern Medical University (Guangzhou, China). The paraffin-embedded sections were deparaffinized in xylene and rehydrated through a graded alcohol series. Antigen retrieval was performed via microwave heating, followed by blocking and permeabilization with 5% bovine serum albumin (BSA) containing 0.3% Triton X-100 for 1 h at room temperature. Sections were then incubated overnight at 4 °C with the following primary antibodies: ATG5 (APG5L) rabbit monoclonal antibody mAb (Zenbio, clone R06-1E5, 1:100, Cat# R381320), BV421-conjugated anti-human CD3 (BD Biosciences, clone SK7, 1:50, Cat# 563797), and RORγt mouse (Santa Cruz Biotechnology, clone 27.92, 1:100, Cat# sc-293150). After washing with PBS, sections were incubated for 1 h at room temperature in the dark with the corresponding fluorophore-conjugated secondary antibodies: Alexa Fluor 488 (AF488)-conjugated goat anti-rabbit IgG H&L (Abcam, 1:200, Cat# ab150077) and Alexa Fluor 647 (AF647)-conjugated goat anti-mouse IgG H&L (Abcam, 1:200, Cat# ab150115). Representative images were captured using a Nikon A1R-si confocal microscope, and the percentage of positive cells was quantified using NIS-Elements Viewer software (version 4.50; Nikon, Tokyo, Japan).

### Enzyme-linked immunosorbent assasy

Intestinal DN ILC3s were sorted from NEC mice, and intracellular lactate concentrations were measured after deproteinization using a lactate colorimetric/fluorometric assay kit (Elabscience, Cat#: E-BC-K002-M). PC levels were quantified using a colorimetric PC assay kit (Elabscience, Cat#: E-BC-K796-M). Concentrations of IL-17A and IL-22 were quantified using commercial ELISA kits (Invitrogen, Cat#: 88-7371-22, Cat#: 88-7422-22), whereas GM-CSF was measured using ELISA kits (Dogesce, Cat#: DG30864M). For human samples, IL-17A and IL-22 concentrations were quantified using corresponding ELISA kits (Dogesce, Cat#: DG10431H, Cat#: DG10322H). All assays were performed in accordance with the manufacturer’s instructions. Absorbance was measured using a microplate reader (ThermoFisher Scientific, Varioskan Lux, USA), and final concentrations were calculated against the corresponding standard curves.

### Quantitative real-time PCR

Total RNA was extracted using TRIzol reagent (Invitrogen, Cat#: 15596-026) and reverse transcribed using a StarScript II First-strand cDNA Synthesis Kit (GenStar, Cat#: A214-10) in a ProFlex PCR System (ThermoFisher Scientific, USA). qPCR was then performed using a RealStar Green Power Mixture kit (GenStar, Cat#: A308-10) in a QuantStudio 6 Flex system (Thermo Fisher Scientific, USA). mRNA expression levels were determined using the relative standard curve method and normalized to β-actin expression, with the lowest expression in the control group set to 1. The relative mRNA expression of other genes was measured using the standard 2^−△△Ct^ method. The primer sequences used in this study are listed in Supplementary Table 4.

### Chromatin immunoprecipitation assay

Sorted DN ILC3s were fixed in a 1% formaldehyde solution, cross-linked with 0.125 mol/L glycine, and lysed using ultrasound. Cell lysates were incubated with anti-HIF-1α (CST, Cat#: 36169 T) or anti-IgG antibodies (CST, Cat#: 2729S) for immunoprecipitation. The antibody-chromatin complexes were collected using protein A/G-agarose beads (Invitrogen, Cat#: 10001D, Cat#: 10003D). After de-crosslinking, DNA was purified and quantified by qPCR using specific primers (Supplementary Table 4), with the first 10% of the lysate used as input control. Data were calculated as the percentage of input (% input).

### Transmission electron microscopy of autophagic vesicles

Sorted DN ILC3s were fixed with 4% glutaraldehyde and post-fixed with 1% OsO_4_ in 0.1 M cacodylate buffer containing 0.1% CaCl_2_ for 2 h at 4 °C. The fixed samples were then stained with 1% uranyl acetate, dehydrated in increasing concentrations of ethanol, and embedded in araldite. Thin sections were obtained and stained with uranyl acetate and lead citrate, then analyzed with a transmission electron microscope (Tecnai G2, FEI) at 30,000 × magnification. Electron microscopy imaging was performed by Servicebio Biotechnology Co., Ltd. (Wuhan, China).

### Quality control and single-cell RNA sequencing data preprocessing

The raw sequencing data were converted and preprocessed by Gene Denovo Biotechnology Co., Ltd. (Guangzhou, China) using 10 × Genomics Cell Ranger software V3.1.0 with default parameters. The pre-mRNA reference (Ensemble_release 109, *Mus musculus*) was then compared with the preprocessed data. Droplets with low-quality barcodes or unique molecular identifiers (UMI) counts were excluded. DoubletFinder V2.0.3 was used to identify cells exhibiting aberrant parameters, such as gene counts deviating from the standard range (500–3700 per cell), unique molecular identifier counts ≥ 15,000, or mitochondrial gene percentage ≥ 10%. After eliminating low-quality cells, a data merging process was executed, accompanied by batch effect correction using Harmony with a resolution of 0.1. Gene expression per cell was then normalized via total expression using global scaling normalization to derive normalized counts. The expression matrix was scaled and integrated. Differentially expressed genes were identified using the following criteria: expression in >25% of cells in the target cluster, with *P* < 0.05 and log_2_ fold-change ≥ 0.25. Heatmaps and violin plots were generated using the R package (v.4.2.2). KEGG pathway enrichment was performed on the identified gene set.

For human scRNA-seq analysis, data were obtained from the Zenodo database (10.5281/zenodo.5813397) and analyzed using the R package Seurat (v5.1.0). Gene expression was visualized via uniform manifold approximation and projection (UMAP).

### Assay for transposase-accessible chromatin sequencing

Nuclei suspensions were incubated in Transposition Mix containing transposase at 37 °C for 30 min, where transposase fragmented the open-chromatin DNA and ligated the adapters onto the fragment ends. Transposition products were purified with QIAGEN MinElute Kit, PCR-amplified, and sequenced on NovaSeq X Plus (Gene Denovo). The methods for library construction and analysis of sequencing results were provided by Genedenovo Biotechnology Co., Ltd (Guangzhou, China). Raw reads were filtered to remove adapter-containing reads, as well as those with >10% N bases and >50% low-quality (Q ≤ 20) bases for clean reads. Clean reads were aligned to the reference genome using Bowtie 2 (v2.2.8, -X 2000, --mm), with mitochondrial/chloroplast alignments and duplicates removed. Reads were strand-offset (+4 bp for +strand, −5 bp for -strand), and peaks calling was performed using MACS 2 (v2.1.2, --nomodel --shift -100 --extsize 200 -B --SPMR -q 0.05). Peaks were annotated via ChIPseeker, the hypergeometric test was used for GO/KEGG enrichment (false discovery rate ≤0.05), MEME-ChIP/AME identified the transcription factor motifs, and DiffBind analyzed differential peaks (false discovery rate <0.05) across groups.

### RNA sequencing

For RNA sequencing analysis, ILC3s were sorted from Atg5^fl/fl^ or Rorc^cre^Atg5^fl/fl^ mice (three samples were pooled from 12 mice) for transcriptome analysis. The methods for library construction and analysis of sequencing results were authored by Genedenovo Biotechnology Co., Ltd (Guangzhou, China). Differentially expressed genes identified using DESeq2 (fold-change > 1.5, *P* < 0.05) were highlighted in the GSEA plot and used for KEGG analysis. All heatmaps were generated using an integrative toolkit software.

### 16S rRNA gene sequencing

The HiPure Fecal DNA Kit (Magen, Guangzhou, China) was used to extract total genomic DNA from samples according to the manufacturer’s protocol. After DNA extraction, the bacterial 16S V3–V4 region was amplified and sequenced on the Illumina MiSeq platform at GeneDenovo Biotechnology Co., Ltd (Guangzhou, China). Raw data were processed and analyzed using the HiSeq2500 PE250 platform.

### LC-MS/MS method for lipid analysis

Initially, the samples were separated using a Nexera LC-30A ultra-high performance liquid chromatography system. A C18 chromatographic column was used with a column temperature of 45 °C and flow rate of 300 μL/min. Mobile phase A comprised an acetonitrile–water solution (acetonitrile:water=6:4,v/v) and mobile phase B comprised an acetonitrile–isopropanol solution (acetonitrile:isopropanol=1:9, v/v). The gradient elution program was as follows: 0–2 min, 30% B; 2–25 min, 30% to 100% B; 25–35 min, 30% B. Throughout analysis, the samples were kept in an autosampler at 10 °C to minimize signal variability due to instrument fluctuations. To avoid any systematic errors, the samples were analyzed in a random order.

Mass spectra were acquired using a Q-Exactive Plus instrument in positive and negative modes. The electrospray ionization parameters were optimized and preset for all measurements: source temperature, 300 °C; capillary temperature, 350 °C; ion spray voltage, 3000 V; S-Lens RF Level, 50%; and scan range (mass-to-charge ratio, m/z) 200–1800. The lipid molecules and fragments were detected using the following MS/MS acquisition method. After each full scan, 10 fragment spectra (MS2 scan, HCD) were collected. The resolution of MS1 was 70,000 at m/z 200, whereas the resolution of MS2 was 17,500 at m/z 200. Metabolomic profiling was performed in collaboration with Gene Denovo Biotechnology Co., Ltd. (Guangzhou, China).

### Statistical analysis

All experimental data were analyzed using GraphPad Prism 8.0 (GraphPad Software Inc., San Diego, CA, USA). After testing for homogeneity of variance, a two-tailed unpaired Student’s *t*-test or Wilcox test was used for data with equal variable and normal distribution. When variance was unequal or distribution was not normal, the rank-sum or nonparametric Mann–Whitney U test was used. Correlation analysis was used to assess associations between variables. One-way ANOVA was applied for multigroup comparisons. Box plots show the median (center line, 50th percentile), with the lower and upper bounds of the box representing the 25th and 75th percentiles, respectively. Whiskers extend to the absolute minimum and maximum values (0th and 100th percentiles, respectively) of the dataset. ns = not significant.

### Reporting summary

Further information on research design is available in the Nature Portfolio Reporting Summary linked to this article.

## Supplementary information

Supplementary Information

Reporting Summary

Transparent Peer Review file

## Source data

Source Data

## Data Availability

The single-cell RNA-seq (scRNA-seq) data generated in this study have been deposited in the NCBI Gene Expression Omnibus (GEO) database under accession code GSE306522. The ATAC-seq data generated in this study have been deposited in the NCBI Gene Expression Omnibus (GEO) database under accession code GSE319269. The bulk RNA-seq data generated in this study have been deposited in the NCBI Gene Expression Omnibus (GEO) database under accession code GSE306398. The 16S rRNA gene sequencing data generated in this study have been deposited in the NCBI Sequence Read Archive (SRA) database under BioProject accession code PRJNA1432033. The metabolomic data generated in this study have been deposited in the MetaboLights database under accession code MTBLS12891. The raw flow cytometry data for the core figures in this study have been deposited in Figshare with the following DOIs: 32025558, 32020050, 32020017, 32019897, 32013036, 32011416, 32010489, 32009283, 32008974, 32008173, 32008092, 32006430. All other raw flow cytometry data are available from the corresponding author upon reasonable request. All data are included in the Supplementary Information or available from the authors. The raw numbers for charts and graphs are available in the Source Data file whenever possible. Source data are provided with this paper.
