# Supplementary material for: Blockade of NKp46⁻ CCR6⁻ ILC3 autophagy protects against necrotizing enterocolitis by restoring energy metabolism balance in mice
Source: Nat Commun. 2026 May 19;17:6579. doi: 10.1038/s41467-026-73356-x (PMC13381918; doi:10.1038/s41467-026-73356-x)
Supplement: Supplementary file 1 — Supplementary Information [file 41467_2026_73356_MOESM1_ESM.pdf]

Blockade of NKp46⁻ CCR6⁻ ILC3 autophagy protects against necrotizing enterocolitis by restoring energy metabolism balance in mice

Junyu He[#1,2,3], Meiqi Chen[#1,2,3], Laiqin Peng[#4], Qiqiong Wang[#5], Yizhuang Lu[1], Yimin Chen[1], Xinyao Li[1], Yanling Mou[3], Jianjun Wang[3], Yuxiong Guo[2*], Kai Wu[3*] and Yumei He[1,2,3,6*]

Supplementary Information:

Supplementary Figure 1-36

Supplementary Table 1-4

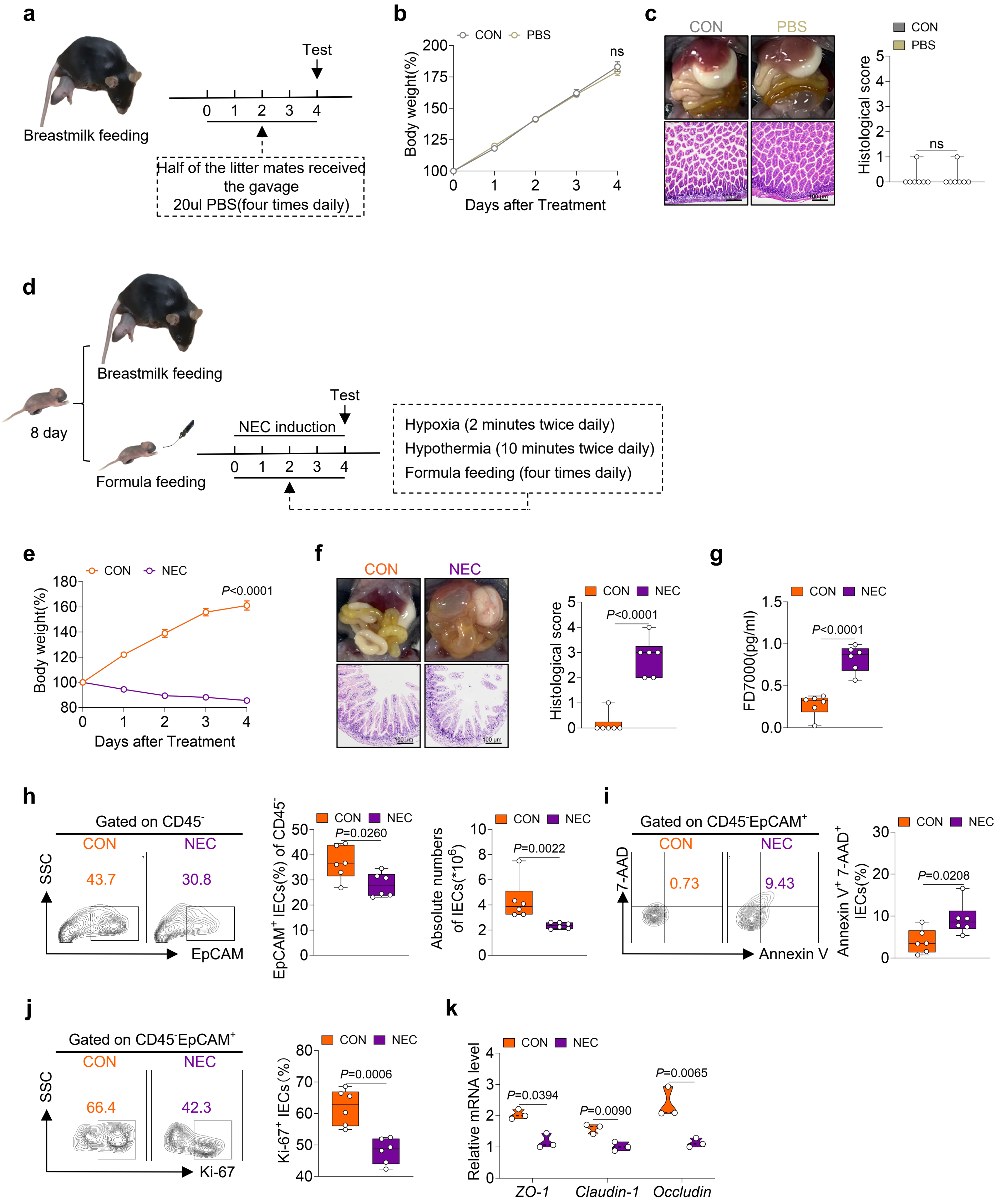

a
Test
Breastmilk feeding
Half of the litter mates received the gavage
20ul PBS(four times daily)
b
CON PBS
ns
Body weight(%)
Days after Treatment
c
CON
PBS
Histological score
ns
d
Breastmilk feeding
8 day
Formula feeding
NEC induction
Test
Hypoxia (2 minutes twice daily)
Hypothermia (10 minutes twice daily)
Formula feeding (four times daily)
e
CON NEC
P<0.0001
Body weight(%)
Days after Treatment
f
CON
NEC
Histological score
P<0.0001
g
CON NEC
FD7000(pg/ml)
P<0.0001
h
Gated on CD45-
CON NEC
43.7
30.8
SSC
EpCAM
EpCAM+ IECs(%) of CD45-
P=0.0260
Absolute numbers of IECs(*10^6)
P=0.0022
i
Gated on CD45-EpCAM+
CON NEC
0.73
9.43
7-AAD
Annexin V
Annexin V+ 7-AAD+ IECs(%)
P=0.0208
j
Gated on CD45-EpCAM+
CON NEC
66.4
42.3
SSC
Ki-67
Ki-67+ IECs(%)
P=0.0006
k
CON NEC
Relative mRNA level
P=0.0394
P=0.0090
P=0.0065
ZO-1
Claudin-1
Occludin

Supplementary Figure 1. Experimental NEC induction causes intestinal injury in mice. **a** Experimental schema of breastmilk feeding and oral gavage procedure. **b** Body weight changes in the indicated groups (n = 7 biological replicates per group). Data are presented as mean ± SEM. **c** Macroscopic image and hematoxylin and eosin (H&E) staining of the intestines (scale bar: 100 μm), with corresponding inflammation scores (n = 7 biological replicates per group). **d** Experimental schema of NEC induction. **e** Body weight changes in the indicated groups (n = 6 biological replicates per group). Data are presented as mean ± SEM. **f** Macroscopic image and H&E staining of the intestines (scale bar: 100 μm), with corresponding inflammation scores (n = 6 biological replicates per group). **g** Intestinal permeability was assessed by plasma FD7000 concentrations (n = 6 biological replicates per group). **h** Representative flow cytometry profiles and statistical analysis of percentages and absolute numbers of IECs (n = 6 biological replicates per group). **i** Representative flow cytometry profiles and statistical analysis of Annexin V^+7-AAD^+ IECs (n = 6 biological replicates per group). **j** Representative flow cytometry profiles and statistical analysis of $Ki67^+$ IECs (n = 6 biological replicates per group). **k** Relative mRNA levels of *ZO-1*, *Claudin-1*, and *Occludin* in the intestines (n = 3 biological replicates per group). All experiments were performed using C57BL/6 mice of both sexes at P8, with littermates randomly assigned to control and experimental groups. Each data point represents one biologically independent mouse, and results are representative of at least three independent experiments. Box plots show the median (centre line, 50th percentile), with the lower and upper bounds of the box representing the 25th and 75th percentiles, respectively. Whiskers extend to the absolute minimum and maximum values (0th and 100th percentiles, respectively) of the dataset. P-values were determined by unpaired two tailed Student's t tests (**b**, **c**, and **e**–**k**). ns = not significant. Source data are provided as a Source Data file.

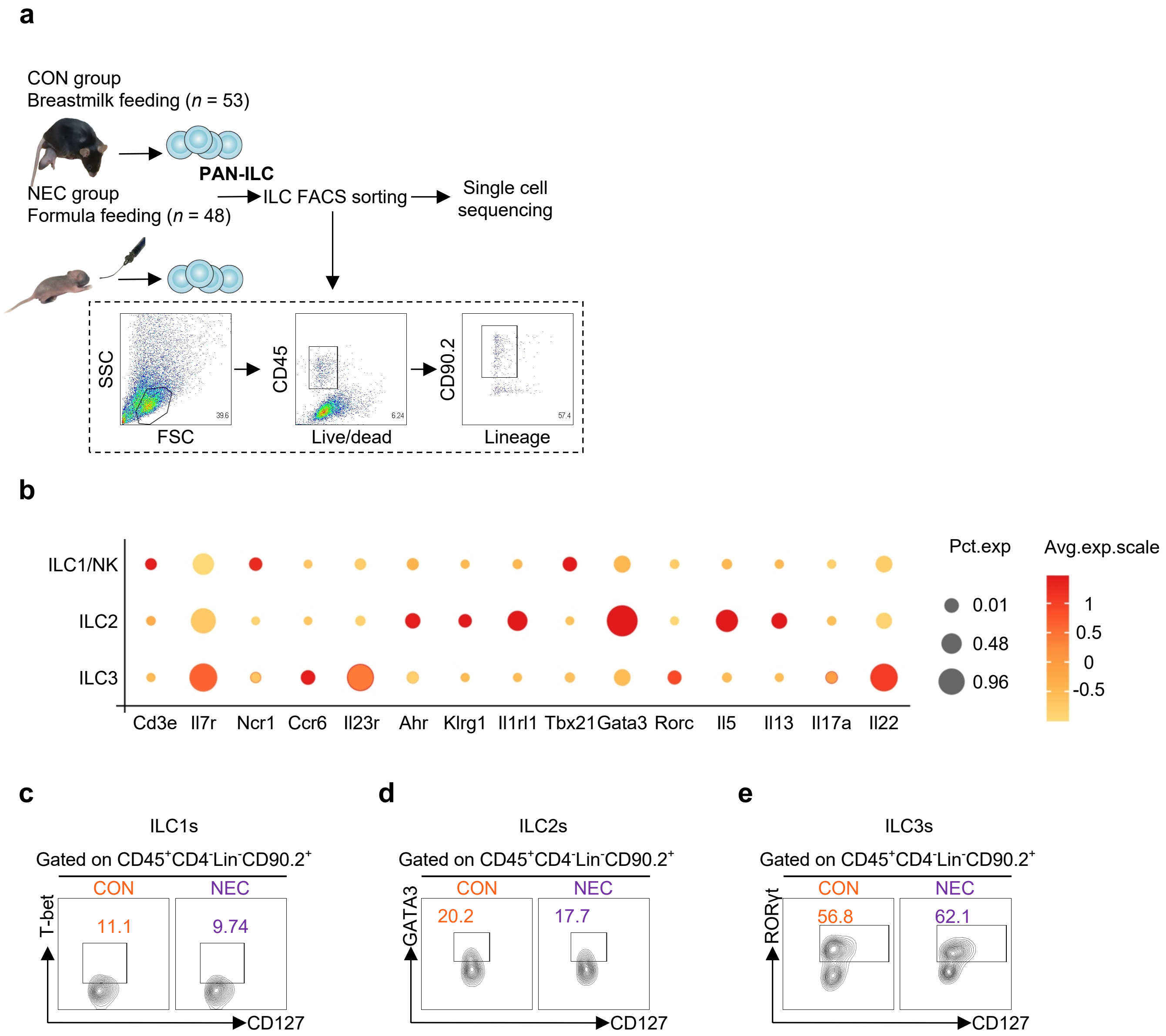

Supplementary Figure 2. Single-cell transcriptome and flow cytometry profiles of intestinal ILCs from control and NEC mice. a Schematic diagram depicting the workflow for ILC sorting from breast-fed control (CON) (*n* = 53) and NEC model (NEC) (*n* = 48) neonatal mice. Single cells were isolated from the intestines, enriched using a Pan-ILC enrichment kit, and then sorted by fluorescence-activated cell sorting (FACS). **b** Bubble plots showing the mean expression of specific subset signature genes across different ILC populations. **c**–**e** Representative flow cytometry profiles of intestinal ILC1s **(c)**; ILC2s **(d)**; and ILC3s **(e)**.

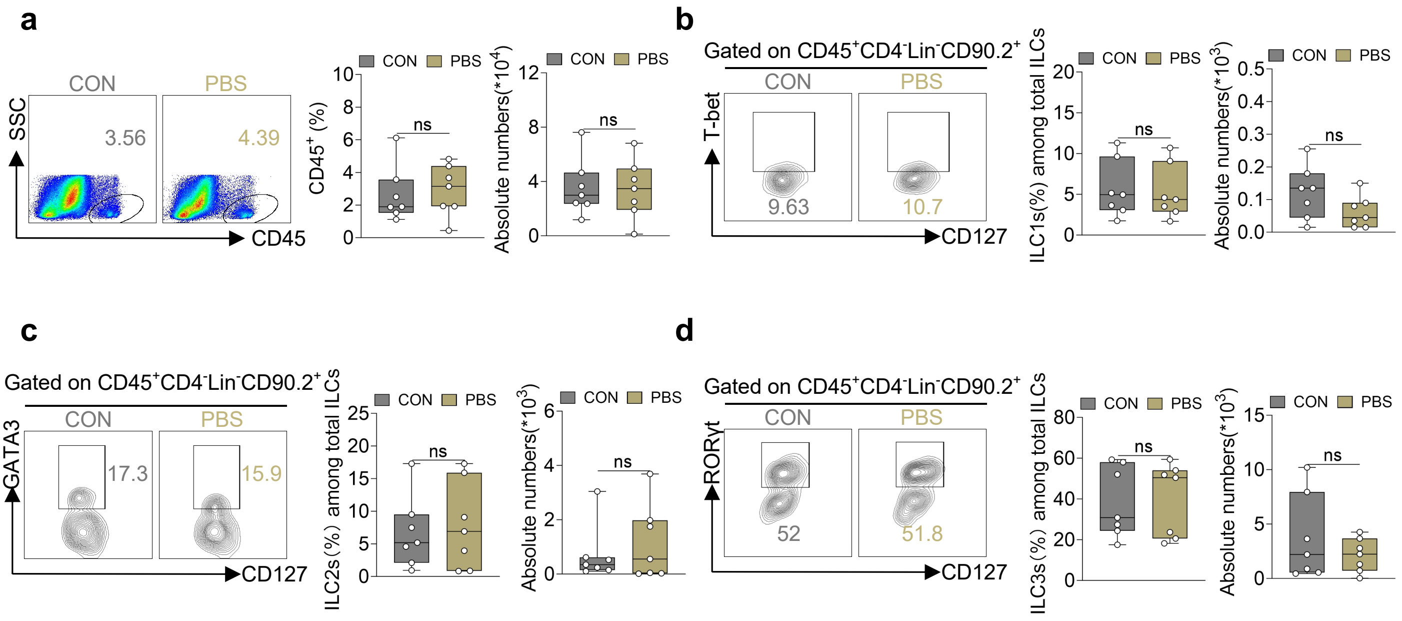

Supplementary Figure 3. Oral gavage procedure does not alter the levels of $CD45^+$ cells, ILC1s, ILC2s, or ILC3s. a Representative flow cytometry profiles and percentages and absolute number of intestinal $CD45^+$ cells (*n* = 7 biological replicates per group). **b** Representative flow cytometry profiles and percentages and absolute number of intestinal ILC1s (*n* = 7 biological replicates per group). **c** Representative flow cytometry profiles and percentages and absolute number of intestinal ILC2s (*n* = 7 biological replicates per group). **d** Representative flow cytometry profiles and percentages and absolute number of intestinal ILC3s (*n* = 7 biological replicates per group). All experiments were performed using C57BL/6 mice of both sexes at P8, with littermates randomly assigned to control and experimental groups. Each data point represents one biologically independent mouse, and results are representative of at least three independent experiments. Box plots show the median (centre line, 50th percentile), with the lower and upper bounds of the box representing the 25th and 75th percentiles, respectively. Whiskers extend to the absolute minimum and maximum values (0th and 100th percentiles, respectively) of the dataset. *P*-values were determined by unpaired two tailed Student's t tests (**a**–**d**). ns = not significant. Source data are provided as a Source Data file.

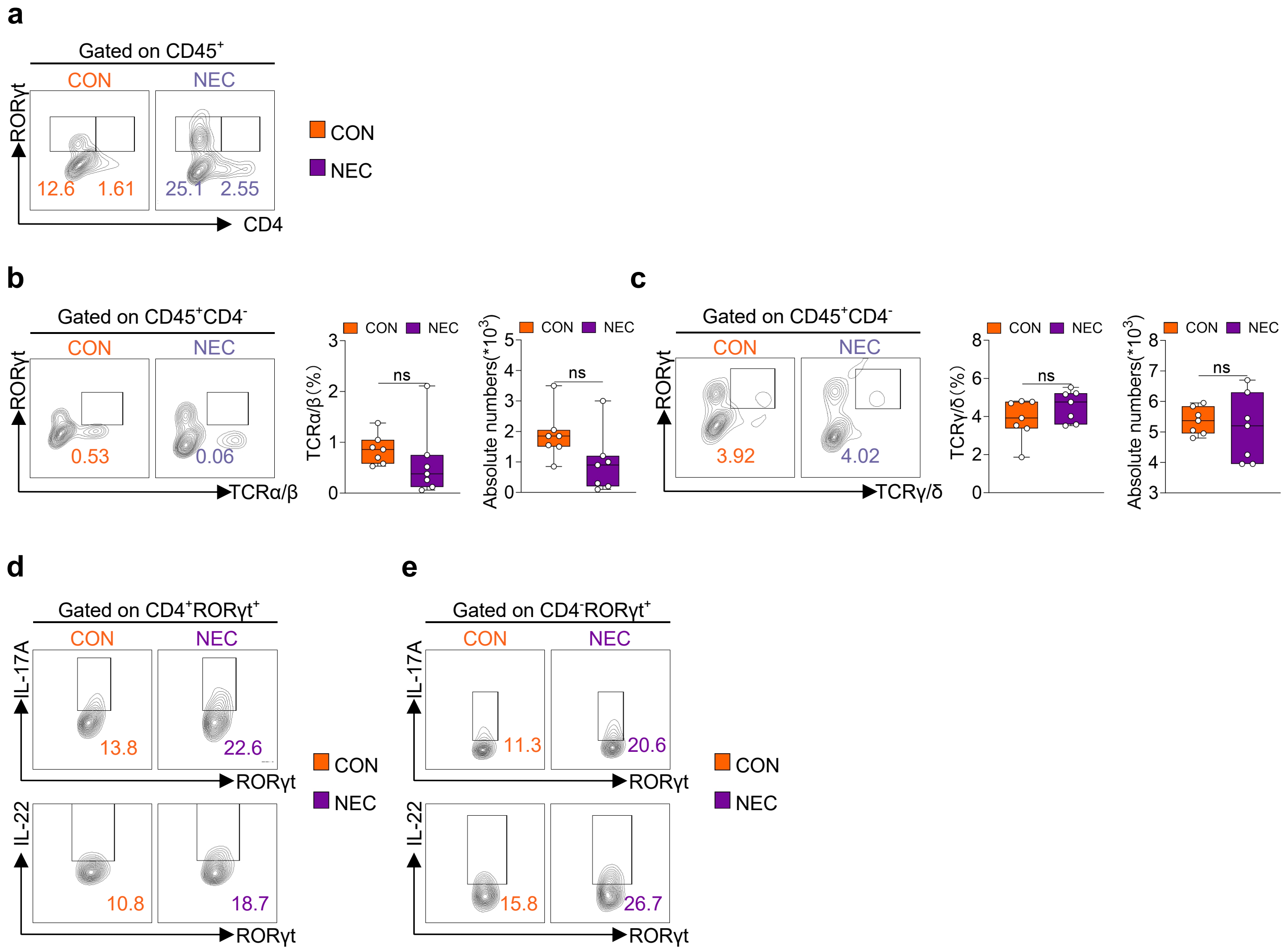

Supplementary Figure 4. Experimental NEC induction in mice leads to the activation of CD4⁺RORγt⁺ cells rather than conventional TCRαβ or TCRγδ T cells. **a** Representative flow cytometry profiles of intestinal CD4⁺RORγt⁺ and CD4⁻RORγt⁺ cells. **b** Representative flow cytometry profiles and percentages and absolute number of intestinal CD4⁻RORγt⁺TCRαβ⁺ cells (*n* = 7 biological replicates per group). **c** Representative flow cytometry profiles and percentages and absolute number of intestinal CD4⁻RORγt⁺TCRγδ⁺ cells (*n* = 7 biological replicates per group). **d** Representative flow cytometry profiles of intestinal IL-17A⁺CD4⁺RORγt⁺ and IL-22⁺CD4⁺RORγt⁺ cells. **e** Representative flow cytometry profiles of intestinal IL-17A⁺CD4⁻RORγt⁺ and IL-22⁺CD4⁻RORγt⁺ cells. All experiments were performed using C57BL/6 mice of both sexes at P8, with littermates randomly assigned to control and experimental groups. Each data point represents one biologically independent mouse, and results are representative of at least three independent experiments. Box plots show the median (centre line, 50th percentile), with the lower and upper bounds of the box representing the 25th and 75th percentiles, respectively. Whiskers extend to the absolute minimum and maximum values (0th and 100th percentiles, respectively) of the dataset. *P*-values were determined by unpaired two tailed Student’s t tests (**b** and **c**). ns = not significant. Source data are provided as a Source Data file.

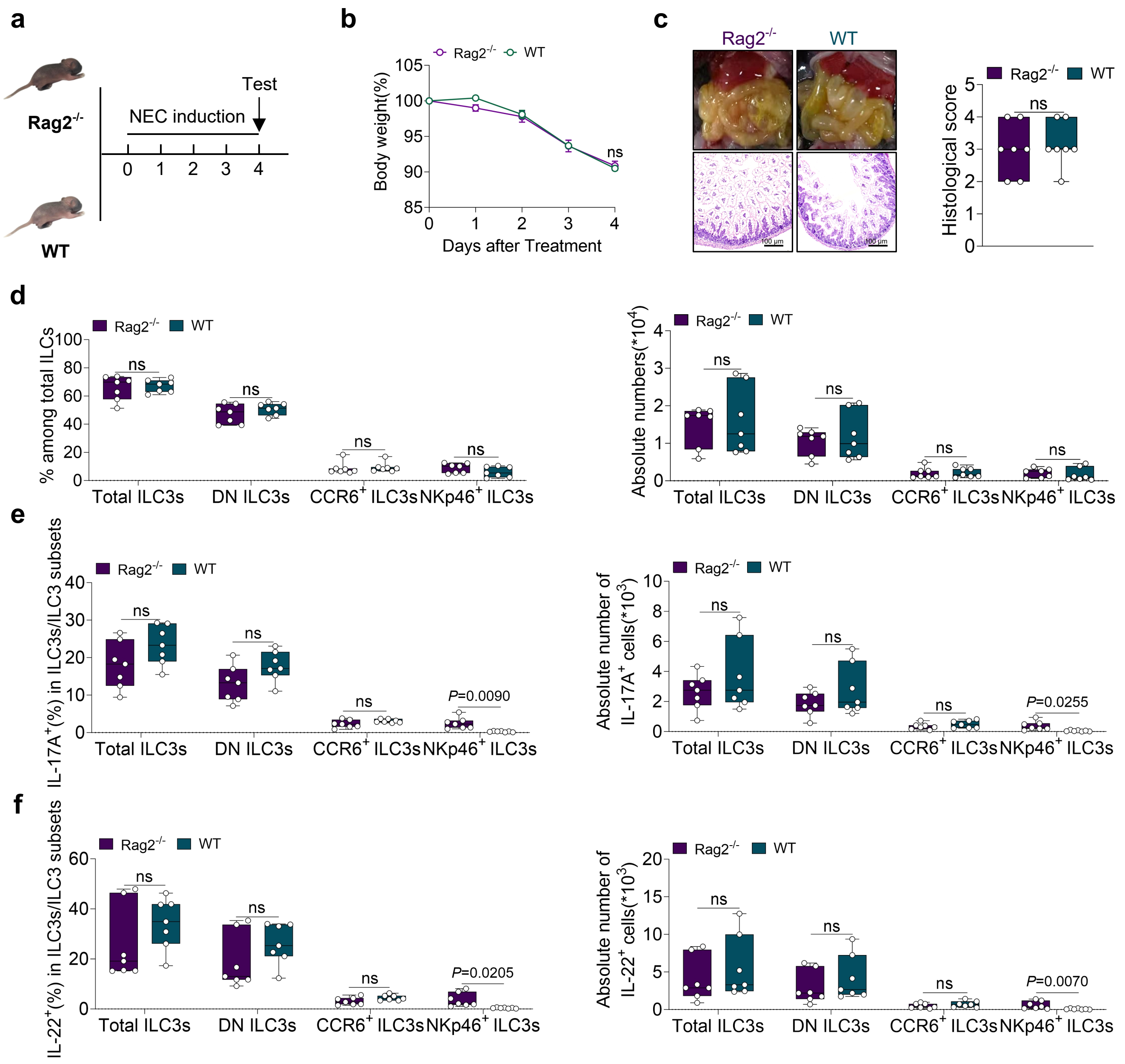

Supplementary Figure 5. Adaptive immune deficiency has no effects on NEC progression. a Experimental schema of NEC induction in Rag2$^{-/-}$ and wild-type littermates. **b** Body weight changes in the indicated groups (*n* = 7 biological replicates per group). Data are presented as mean ± SEM. **c** Macroscopic image and H&E staining of the intestines (scale bar: 100 μm), with corresponding inflammation scores (*n* = 7 biological replicates per group). **d** Percentage and absolute number of intestinal total ILC3s, DN ILC3s, CCR6^{+} ILC3s and NKp46^{+} ILC3s (*n* = 7 biological replicates per group). **e** Percentage and absolute number of intestinal IL-17A^{+} ILC3s and IL-17A^{+} ILC3 subsets (*n* = 7 biological replicates per group). **f** Percentage and absolute number of intestinal IL-22^{+} ILC3s and IL-22^{+} ILC3 subsets (*n* = 7 biological replicates per group). All experiments were performed using C57BL/6 and Rag2$^{-/-}$ mice of both sexes at P8, with littermates randomly assigned to control and experimental groups. Each data point represents one biologically independent mouse, and results are representative of at least three independent experiments. Box plots show the median (centre line, 50th percentile), with the lower and upper bounds of the box representing the 25th and 75th percentiles, respectively. Whiskers extend to the absolute minimum and maximum values (0th and 100th percentiles, respectively) of the dataset. *P*-values were determined by unpaired two tailed Student's t tests (**b**–**f**). ns = not significant. Source data are provided as a Source Data file.

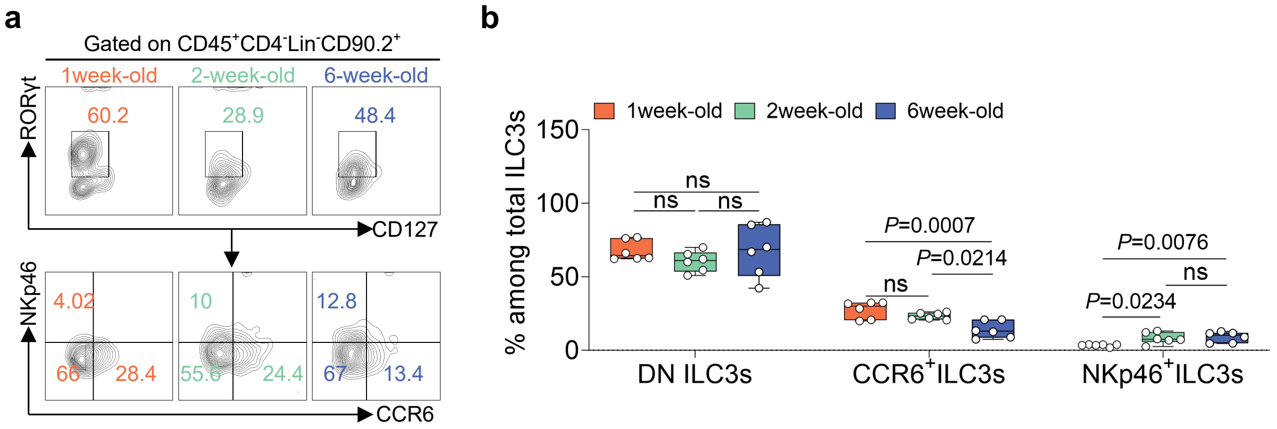

Supplementary Figure 6. Dynamic changes in the proportion of ILC3 subsets during postnatal development. a Representative flow cytometry profiles of intestinal ILC3 subsets. **b** Statistical analysis of intestinal double negative (DN) ILC3s, $CCR6^+$ ILC3s, and $NKp46^+$ ILC3s (n = 6 biological replicates per group). All experiments were performed using C57BL/6 mice of both sexes, with littermates randomly assigned to control and experimental groups. Each data point represents one biologically independent mouse, and results are representative of at least three independent experiments. Box plots show the median (centre line, 50th percentile), with the lower and upper bounds of the box representing the 25th and 75th percentiles, respectively. Whiskers extend to the absolute minimum and maximum values (0th and 100th percentiles, respectively) of the dataset. *P*-values were determined by one-way ANOVA followed by Tukey-Kramer multiple comparisons test (two-sided) (**b**). ns = not significant. Source data are provided as a Source Data file.

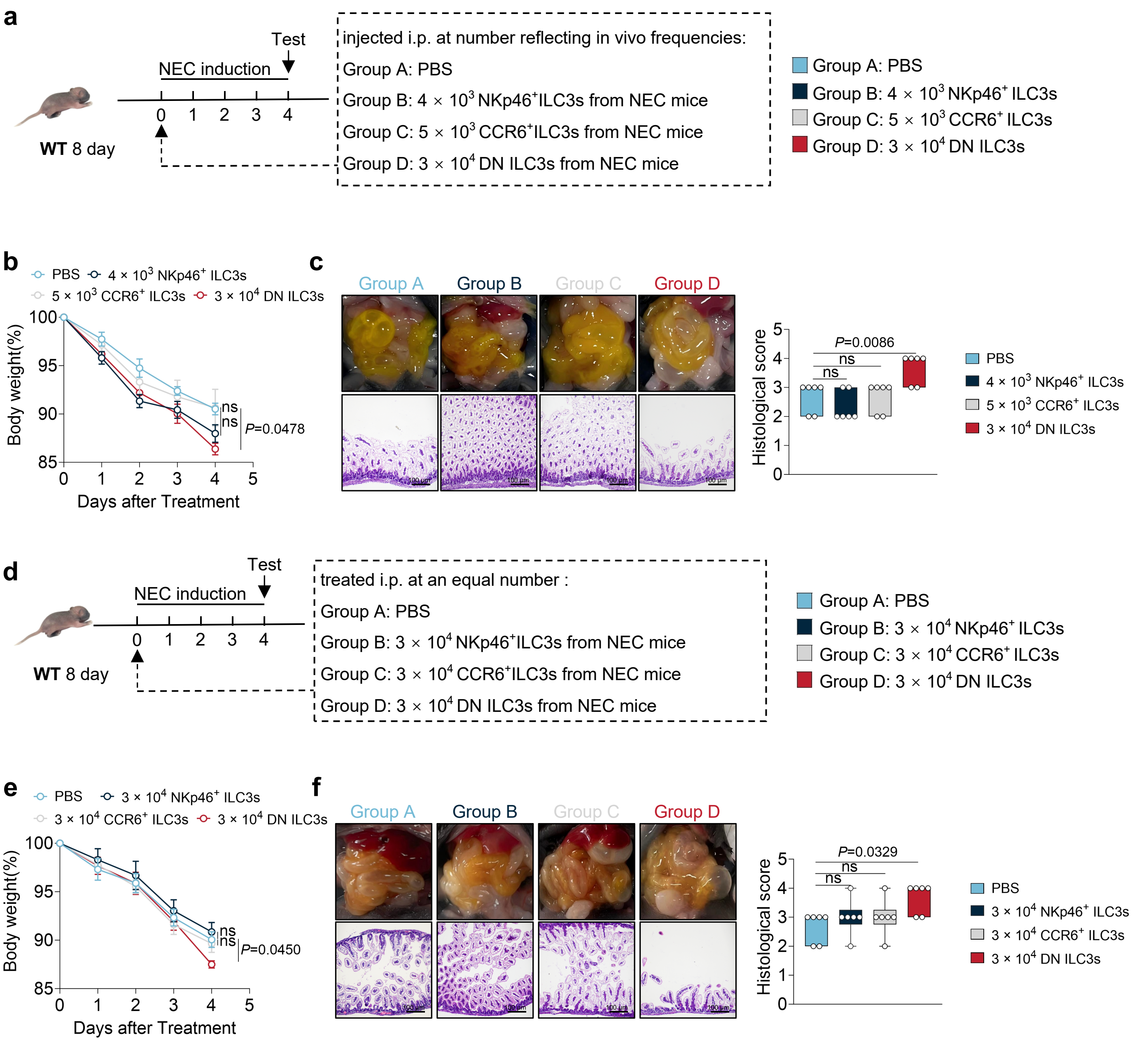

Supplementary Figure 7. Adoptive transfer of DN ILC3s, but not NKp46$^+$ or CCR6$^+$ ILC3s, exacerbates intestinal inflammation in NEC. **a** Schematic diagram of adoptive transfer prior to NEC induction. **b** Body weight changes in the indicated groups (*n* = 6 biological replicates per group). Data are presented as mean ± SEM. **c** Macroscopic image and H&E staining of the intestines (scale bar: 100 μm), with corresponding inflammation scores (*n* = 6 biological replicates per group). **d** Schematic diagram of adoptive transfer at equal numbers. **e** Body weight changes in the indicated groups (*n* = 6 biological replicates per group). Data are presented as mean ± SEM. **f** Macroscopic image and H&E staining of the intestines (scale bar: 100 μm), with corresponding inflammation scores (*n* = 6 biological replicates per group). All experiments were performed using C57BL/6 mice of both sexes at P8, with littermates randomly assigned to control and experimental groups. Each data point represents one biologically independent mouse, and results are representative of at least three independent experiments. Box plots show the median (centre line, 50th percentile), with the lower and upper bounds of the box representing the 25th and 75th percentiles, respectively. Whiskers extend to the absolute minimum and maximum values (0th and 100th percentiles, respectively) of the dataset. *P*-values were determined by one-way ANOVA followed by Tukey-Kramer multiple comparisons test (two-sided) (**b**, **c**, **e**, and **f**). ns = not significant. Source data are provided as a Source Data file.

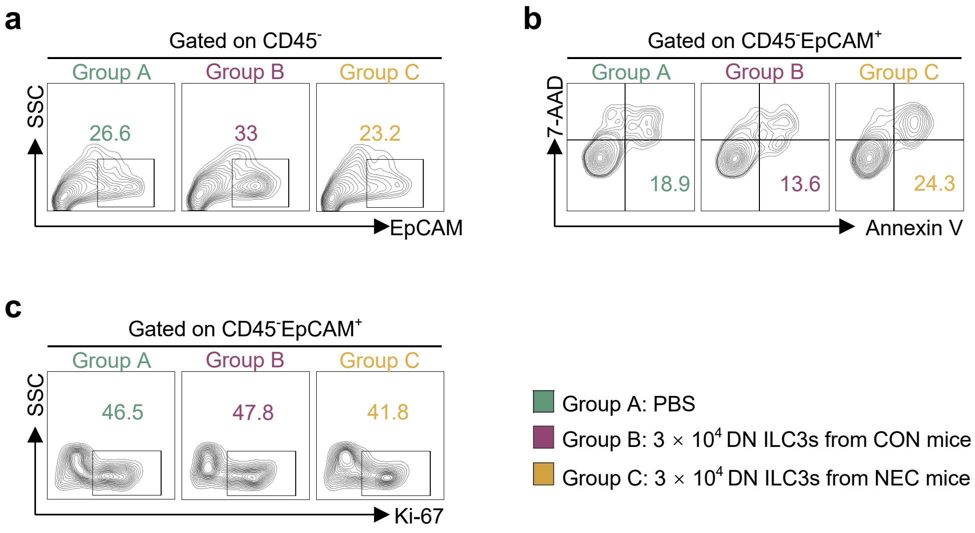

Supplementary Figure 8. Adoptive transfer of pathogenic DN ILC3s causes IEC reduction and induces cell death. a Representative flow cytometry profiles of IECs from different groups after adoptive transfer. **b** Representative flow cytometry profiles of Annexin V$^+$7-AAD$^+$ IECs. **c** Representative flow cytometry profiles of Ki67$^+$ IECs.

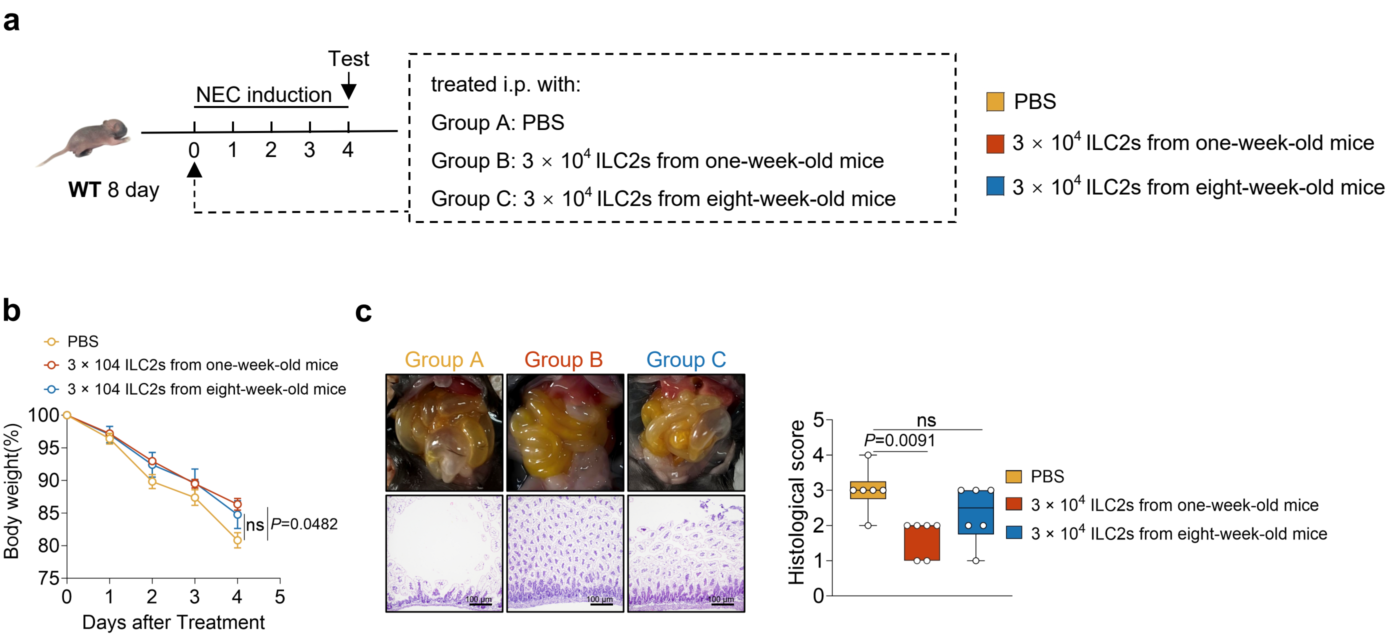

Supplementary Figure 9. Adoptive transfer of neonatal ILC2s ameliorates NEC. a Schematic diagram of adoptive transfer using ILC2s from 1-week-old or 8-week-old mice prior to NEC induction. **b** Body weight changes in the indicated groups ($n = 6$ biological replicates per group). Data are presented as mean ± SEM. **c** Macroscopic image and H&E staining of the intestines (scale bar: 100 μm), with corresponding inflammation scores ($n = 6$ biological replicates per group). All experiments were performed using C57BL/6 mice of both sexes at P8, with littermates randomly assigned to control and experimental groups. Each data point represents one biologically independent mouse, and results are representative of at least three independent experiments. Box plots show the median (centre line, 50th percentile), with the lower and upper bounds of the box representing the 25th and 75th percentiles, respectively. Whiskers extend to the absolute minimum and maximum values (0th and 100th percentiles, respectively) of the dataset. *P*-values were determined by one-way ANOVA followed by Tukey-Kramer multiple comparisons test (two-sided) (**b** and **c**). ns = not significant. Source data are provided as a Source Data file.

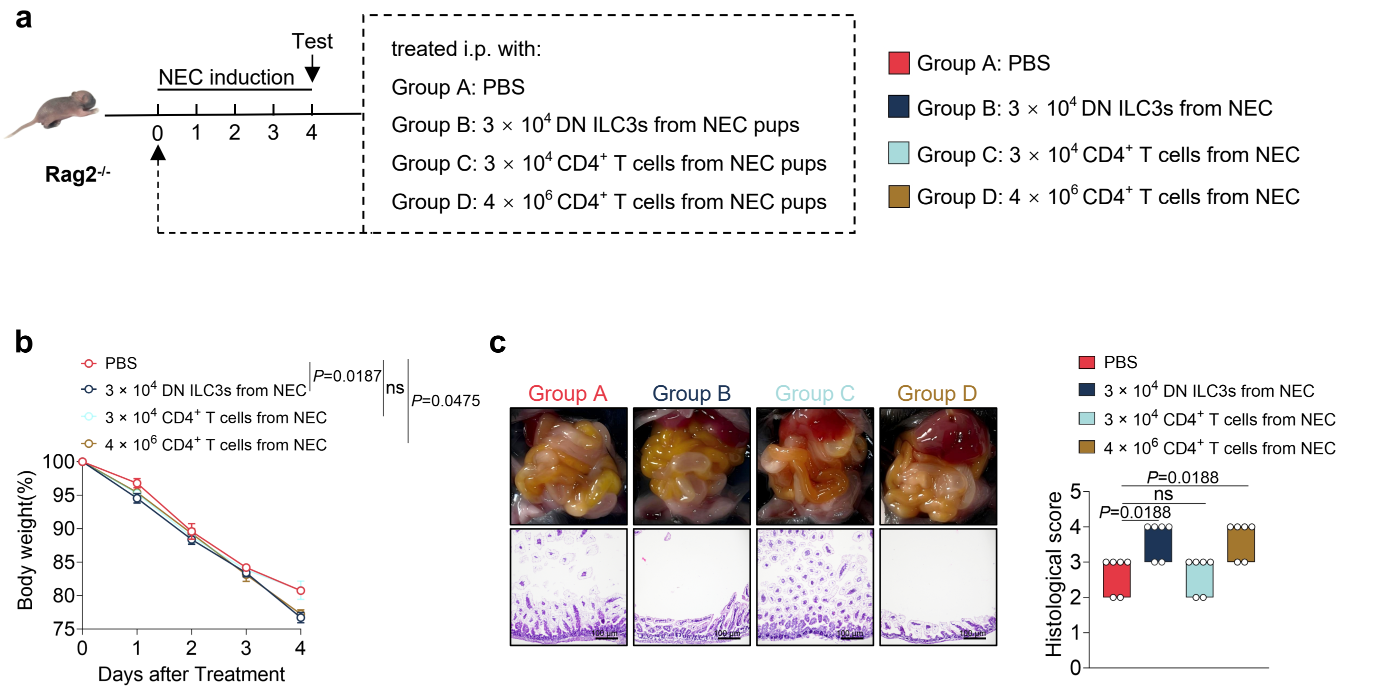

Supplementary Figure 10. Adoptive transfer of intestinal DN ILC3s or high-dose CD4$^+$ T cells exacerbate intestinal inflammation in Rag2$^{-/-}$ mice. a Schematic diagram of adoptive transfer using intestinal CD4$^+$ T cells and DN ILC3s prior to NEC induction. **b** Body weight changes in the indicated groups (*n* = 6 biological replicates per group). Data are presented as mean ± SEM. **c** Macroscopic image and H&E staining of the intestines (scale bar: 100 μm), with corresponding inflammation scores (*n* = 6 biological replicates per group). All experiments were performed using Rag2$^{-/-}$ mice of both sexes at P8, with littermates randomly assigned to control and experimental groups. Each data point represents one biologically independent mouse, and results are representative of at least three independent experiments. Box plots show the median (centre line, 50th percentile), with the lower and upper bounds of the box representing the 25th and 75th percentiles, respectively. Whiskers extend to the absolute minimum and maximum values (0th and 100th percentiles, respectively) of the dataset. *P*-values were determined by one-way ANOVA followed by Tukey-Kramer multiple comparisons test (two-sided) (**b** and **c**). ns = not significant. Source data are provided as a Source Data file.

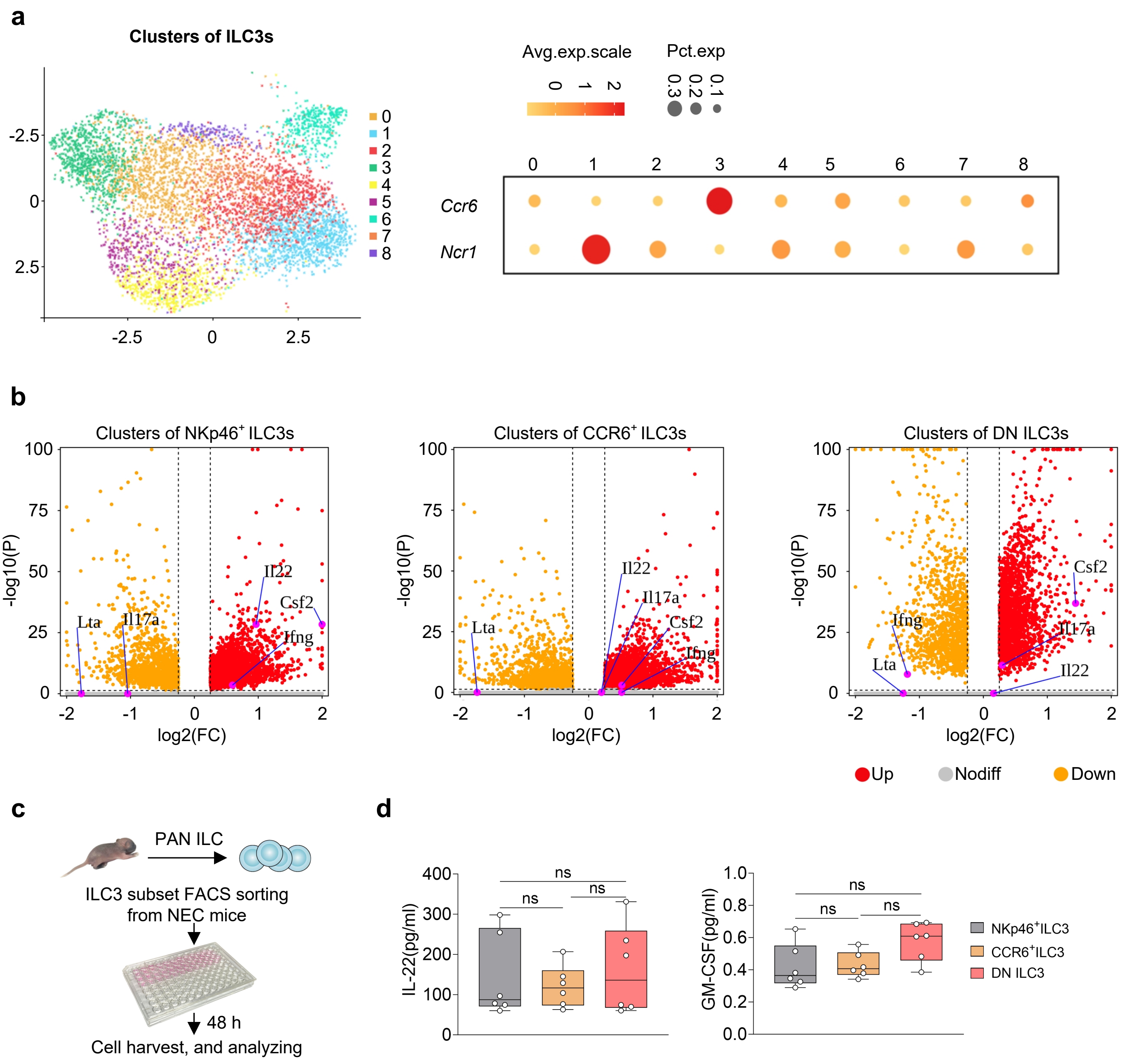

Supplementary Figure 11. Single-cell transcriptomic profiling reveals distinct functional states in DN ILC3s. a Uniform manifold approximation and projection (UMAP) visualization of ILC3 clusters. The right panel showing the expression levels of *Ccr6* and *Ncr1* across these clusters. **b** Volcano plots showing differentially expressed genes in NKp46+ ILC3s, CCR6+ ILC3s, and DN ILC3s. **c** Schematic diagram of in vitro culture system for three ILC3 subsets. **d** Levels of IL-22 and GM-CSF in NKp46+ ILC3, CCR6+ ILC3, and DN ILC3 subsets (n = 6 biological replicates per group). All experiments were performed using C57BL/6 mice of both sexes at P8, with littermates randomly assigned to experimental groups. Each data point represents one biologically independent mouse, and results are representative of at least three independent experiments. Box plots show the median (centre line, 50th percentile), with the lower and upper bounds of the box representing the 25th and 75th percentiles, respectively. Whiskers extend to the absolute minimum and maximum values (0th and 100th percentiles, respectively) of the dataset. *P*-values were determined by one-way ANOVA followed by Tukey-Kramer multiple comparisons test (two-sided) (**d**). ns = not significant. Source data are provided as a Source Data file.

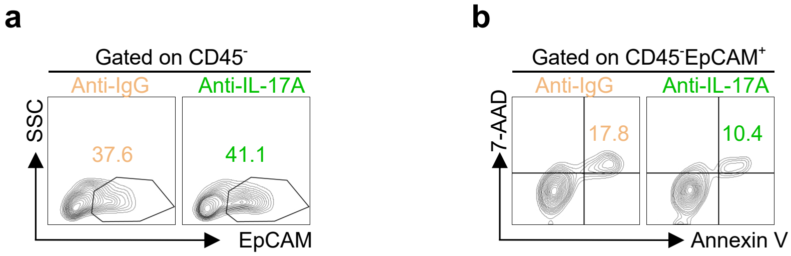

Supplementary Figure 12. Treatment with neutralizing anti-IL-17A antibody increases IEC abundance and inhibits cell death. a Representative flow cytometry profiles of IECs. **b** Representative flow cytometry profiles of Annexin V$^+$7-AAD$^+$ IECs. **c** Representative flow cytometry profiles of Ki67$^+$ IECs.

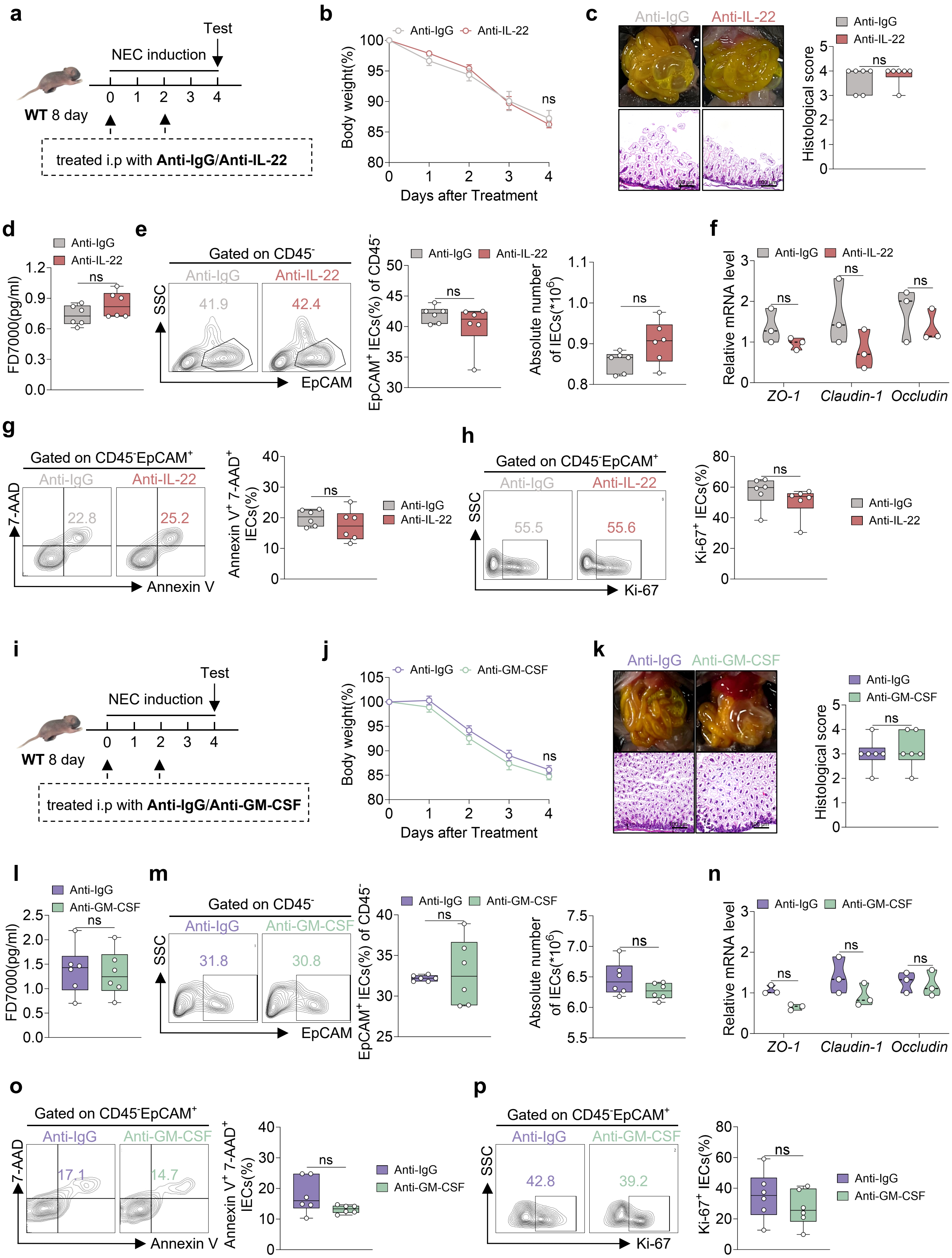
a
Test
NEC induction
WT 8 day
treated i.p with Anti-IgG/Anti-IL-22
b
Anti-IgG
Anti-IL-22
Body weight(%)
Days after Treatment
ns
c
Anti-IgG
Anti-IL-22
Histological score
ns
d
FD7000(pg/ml)
ns
e
Gated on CD45-
Anti-IgG
Anti-IL-22
41.9
42.4
SSC
EpCAM
EpCAM+ IECs(%) of CD45-
Absolute number of IECs(*10^6)
ns
f
Relative mRNA level
ZO-1
Claudin-1
Occludin
ns
g
Gated on CD45-EpCAM+
22.8
25.2
7-AAD
Annexin V
Annexin V+ 7-AAD+ IECs(%)
ns
h
Gated on CD45-EpCAM+
55.5
55.6
SSC
Ki-67
Ki-67+ IECs(%)
ns
i
Test
NEC induction
WT 8 day
treated i.p with Anti-IgG/Anti-GM-CSF
j
Anti-IgG
Anti-GM-CSF
Body weight(%)
Days after Treatment
ns
k
Anti-IgG
Anti-GM-CSF
Histological score
ns
l
FD7000(pg/ml)
ns
m
Gated on CD45-
31.8
30.8
SSC
EpCAM
EpCAM+ IECs(%) of CD45-
Absolute number of IECs(*10^6)
ns
n
Relative mRNA level
ZO-1
Claudin-1
Occludin
ns
o
Gated on CD45-EpCAM+
17.1
14.7
7-AAD
Annexin V
Annexin V+ 7-AAD+ IECs(%)
ns
p
Gated on CD45-EpCAM+
42.8
39.2
SSC
Ki-67
Ki-67+ IECs(%)
ns

Supplementary Figure 13. Treatment with neutralizing anti-IL-22 or anti-GM-CSF antibody has no effect on intestinal barrier function and NEC progression. a Schematic diagram of neutralizing anti-IL-22 antibody (anti-IL-22) treatment during NEC induction. **b** Body weight changes in the indicated groups (n = 6 biological replicates per group). Data are presented as mean ± SEM. **c** Macroscopic image and H&E staining of the intestines (scale bar: 100 μm), with corresponding inflammation scores (n = 6 biological replicates per group). **d** Intestinal permeability was assessed by plasma FD7000 concentrations (n = 6 biological replicates per group). **e** Representative flow cytometry profiles and percentage and absolute number of IECs (n = 6 biological replicates per group). **f** Relative mRNA levels of *ZO-1*, *Claudin-1*, and *Occludin* in the intestines (n = 3 biological replicates per group). **g** Representative flow cytometry profiles and statistical analysis of Annexin V^+7-AAD^+ IECs (n = 6 biological replicates per group). **h** Representative flow cytometry profiles and statistical analysis of $Ki67^+$ IECs (n = 6 biological replicates per group). **i** Schematic diagram of neutralizing anti-GM-CSF antibody (anti-GM-CSF) treatment during NEC induction. **j** Body weight changes in the indicated groups (n = 6 biological replicates per group). Data are presented as mean ± SEM. **k** Macroscopic image and H&E staining of the intestines (scale bar: 100 μm), with corresponding inflammation scores (n = 6 biological replicates per group). **l** Intestinal permeability was assessed by plasma FD7000 concentrations (n = 6 biological replicates per group). **m** Representative flow cytometry profiles and percentage and absolute number of IECs (n = 6 biological replicates per group). **n** Relative mRNA levels of *ZO-1*, *Claudin-1*, and *Occludin* in the intestines (n = 3 biological replicates per group). **o** Representative flow cytometry profiles and statistical analysis of Annexin V^+7-AAD^+ IECs (n = 6 biological replicates per group). **p** Representative flow cytometry profiles and statistical analysis of $Ki67^+$ IECs (n = 6 biological replicates per group). All experiments were performed using C57BL/6 mice of both sexes at P8, with littermates randomly assigned to control and experimental groups. Each data point represents one biologically independent mouse, and results are representative of at least three independent experiments. Box plots show the median (centre line, 50th percentile), with the lower and upper bounds of the box representing the 25th and 75th percentiles, respectively. Whiskers extend to the absolute minimum and maximum values (0th and 100th percentiles, respectively) of the dataset. P-values were determined by unpaired two tailed Student's t tests (**b**–**h** and **j**–**p**). ns = not significant. Source data are provided as a Source Data file.

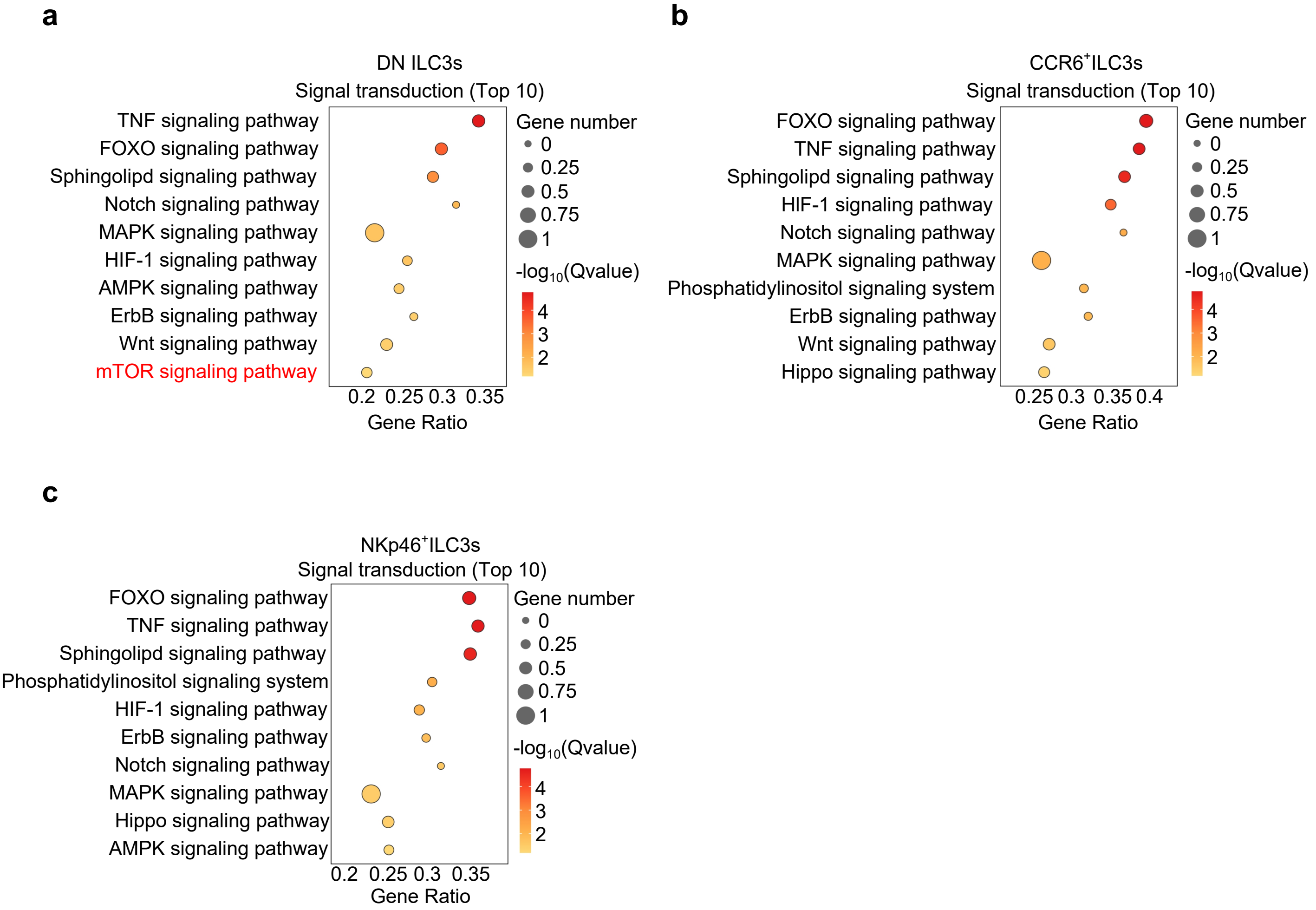

Supplementary Figure 14. Signal transduction pathway enrichment in ILC3 subsets. a–c Dot plots displaying the top 10 significantly enriched signal transduction pathways in NEC DN ILC3s **(a)**; CCR6⁺ ILC3s **(b)**; and NKp46⁺ ILC3s **(c)**.

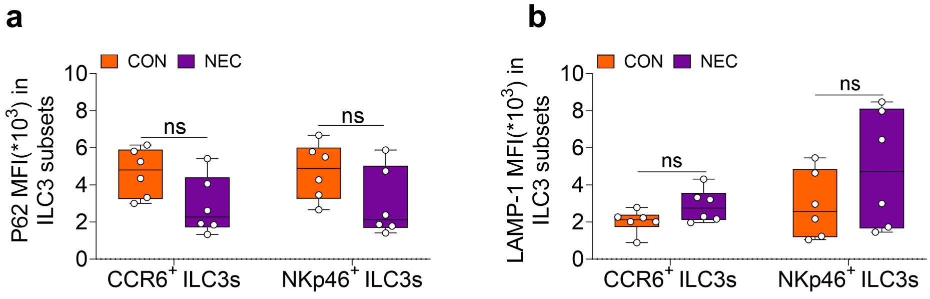

Supplementary Figure 15. Autophagy in CCR6⁺ or NKp46⁺ ILC3s is comparable between control and NEC mice. **a**, **b** Protein levels of P62 **(a)** and LAMP-1 **(b)** in CCR6⁺ ILC3s and NKp46⁺ ILC3s (*n* = 6 biological replicates per group). All experiments were performed using C57BL/6 mice of both sexes at P8, with littermates randomly assigned to control and experimental groups. Each data point represents one biologically independent mouse, and results are representative of at least three independent experiments. Box plots show the median (centre line, 50th percentile), with the lower and upper bounds of the box representing the 25th and 75th percentiles, respectively. Whiskers extend to the absolute minimum and maximum values (0th and 100th percentiles, respectively) of the dataset. *P*-values were determined by unpaired two tailed Student's t tests (**a** and **b**). ns = not significant. Source data are provided as a Source Data file.

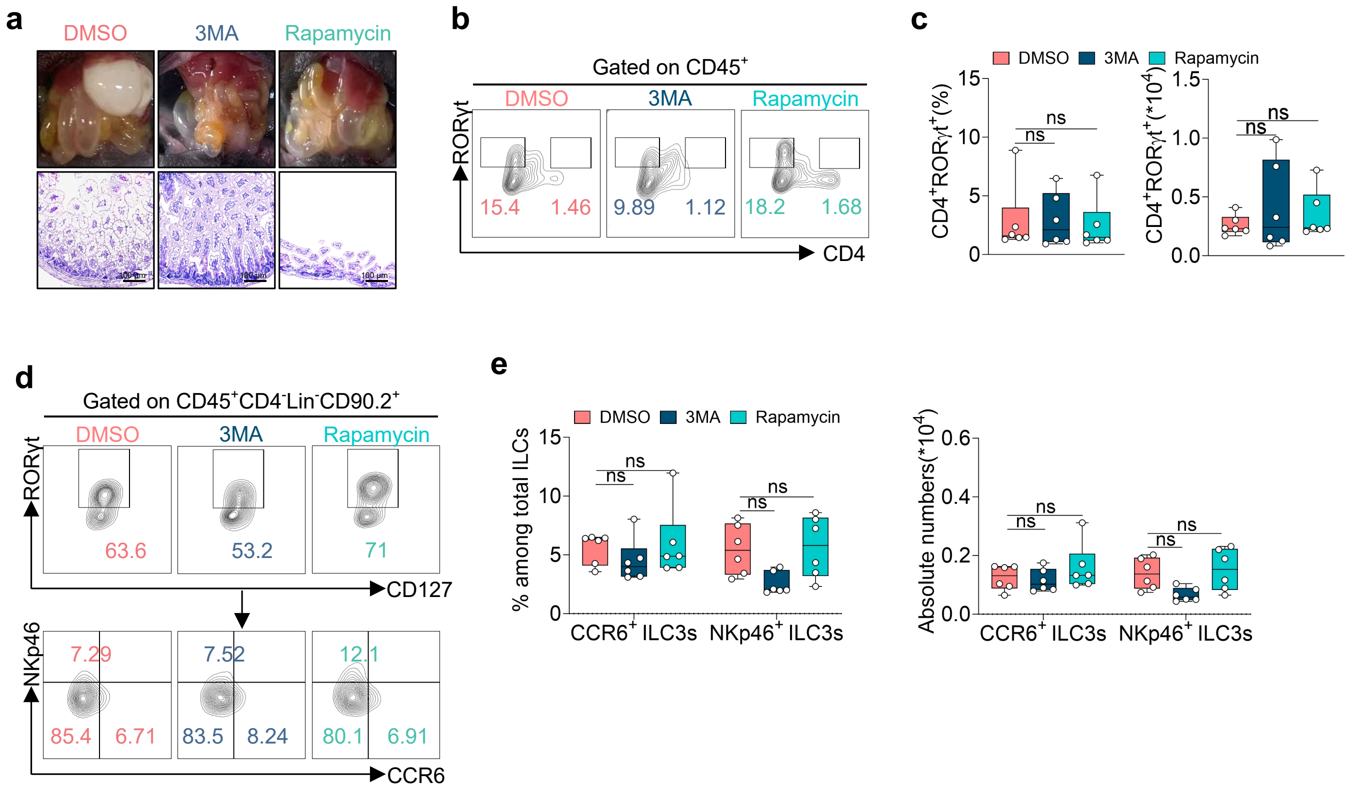

Supplementary Figure 16. Autophagy interventions do not affect the levels of intestinal NKp46⁺ ILC3s, CCR6⁺ ILC3s, and CD4⁺RORγt⁺ cells. **a** Macroscopic image and H&E staining of the intestines (scale bar: 100 μm). **b**, **c** Representative flow cytometry profiles (**b**) and percentage and absolute number (**c**) of intestinal CD4⁺RORγt⁺ cells (*n* = 6 biological replicates per group). **d**, **e** Representative flow cytometry profiles (**d**) and percentage and absolute number (**e**) of total ILC3s and ILC3 subsets (*n* = 6 biological replicates per group). All experiments were performed using C57BL/6 mice of both sexes at P8, with littermates randomly assigned to control and experimental groups. Each data point represents one biologically independent mouse, and results are representative of at least three independent experiments. Box plots show the median (centre line, 50th percentile), with the lower and upper bounds of the box representing the 25th and 75th percentiles, respectively. Whiskers extend to the absolute minimum and maximum values (0th and 100th percentiles, respectively) of the dataset. *P*-values were determined by one-way ANOVA followed by Tukey-Kramer multiple comparisons test (two-sided) (**c, e**). ns = not significant. Source data are provided as a Source Data file.

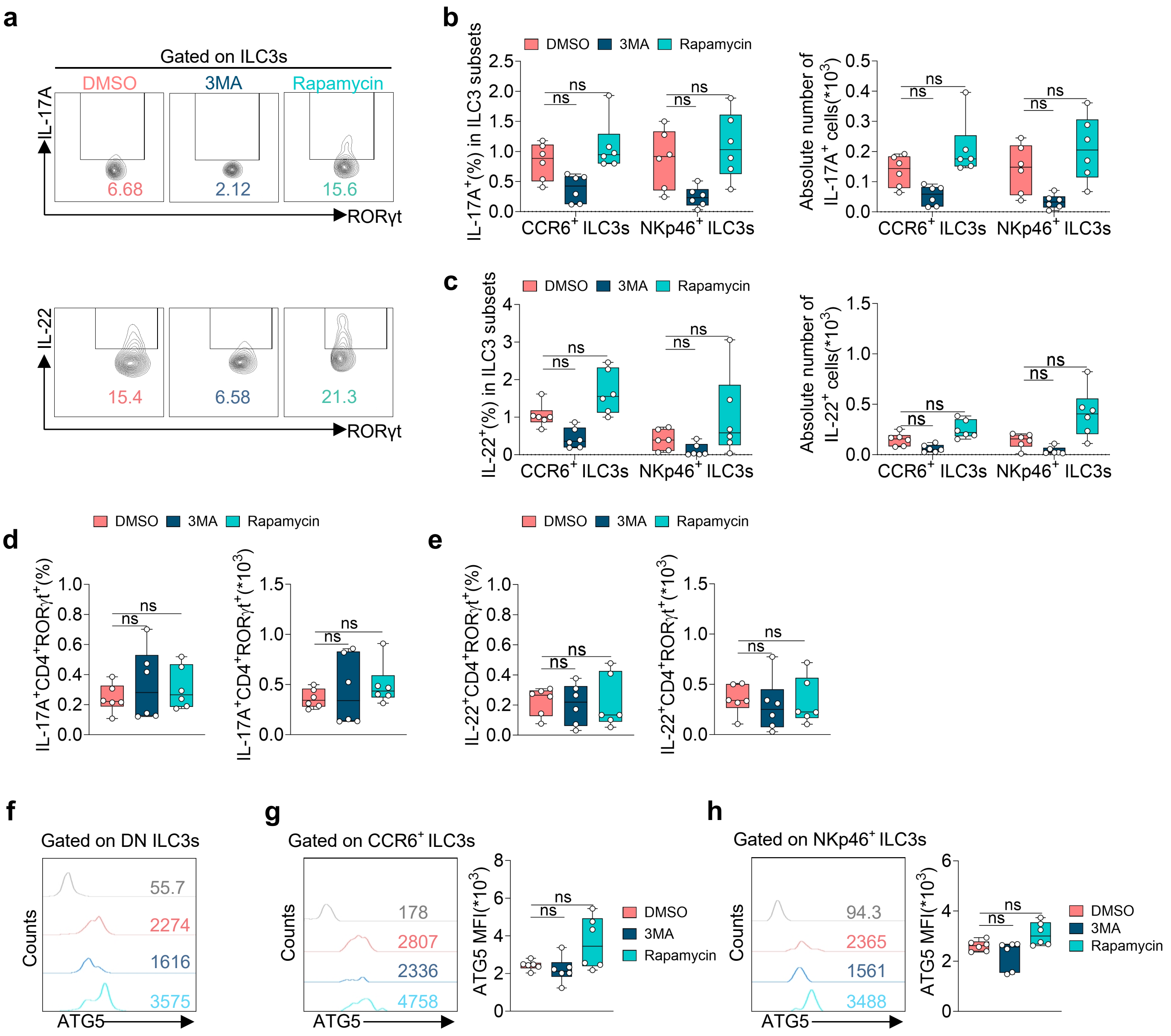

Supplementary Figure 17. Autophagy interventions do not affect the function of intestinal NKp46$^+$ ILC3s, CCR6$^+$ ILC3s, and CD4$^+$RORγt$^+$ cells. a Representative flow cytometry profiles of intestinal IL-17A$^+$ and IL-22$^+$ ILC3s. **b** Percentage and absolute number of IL-17A$^+$CCR6$^+$ and IL-17A$^+$NKp46$^+$ ILC3s (*n* = 6 biological replicates per group). **c** Percentage and absolute number of IL-22$^+$CCR6$^+$ and IL-22$^+$NKp46$^+$ ILC3s (*n* = 6 biological replicates per group). **d** Percentage and absolute number of IL-17A$^+$CD4$^+$RORγt$^+$ cells (*n* = 6 biological replicates per group). **e** Percentage and absolute number of IL-22$^+$CD4$^+$RORγt$^+$ cells (*n* = 6 biological replicates per group). **f** Flow cytometry histogram of ATG5 expression in DN ILC3s. **g** Flow cytometry histogram and quantification of ATG5 expression in CCR6$^+$ ILC3s (*n* = 6 biological replicates per group). **h** Flow cytometry histogram and quantification of ATG5 expression in NKp46$^+$ ILC3s (*n* = 6 biological replicates per group). All experiments were performed using C57BL/6 mice of both sexes at P8, with littermates randomly assigned to control and experimental groups. Each data point represents one biologically independent mouse, and results are representative of at least three independent experiments. Box plots show the median (centre line, 50th percentile), with the lower and upper bounds of the box representing the 25th and 75th percentiles, respectively. Whiskers extend to the absolute minimum and maximum values (0th and 100th percentiles, respectively) of the dataset. *P*-values were determined by one-way ANOVA followed by Tukey-Kramer multiple comparisons test (two-sided) (**b**–**e**, **g**, and **h**). ns = not significant. Source data are provided as a Source Data file.

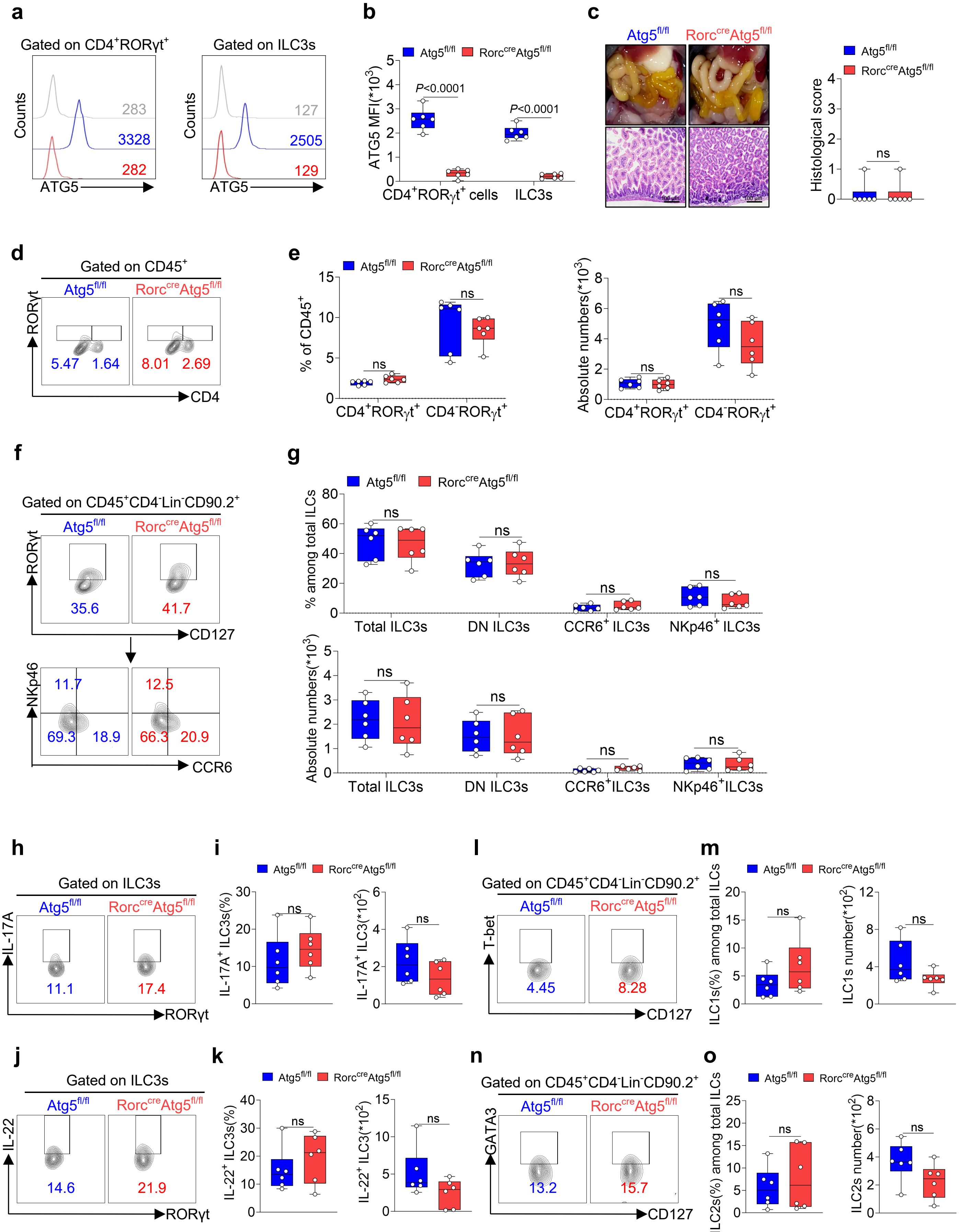

a
Gated on CD4+RORγt+
Gated on ILC3s
Counts
283
3328
282
127
2505
129
ATG5
b
Atg5fl/fl
RorccreAtg5fl/fl
ATG5 MFI(*10^3)
P<0.0001
P<0.0001
CD4+RORγt+ cells
ILC3s
c
Atg5fl/fl
RorccreAtg5fl/fl
Histological score
ns
d
Gated on CD45+
Atg5fl/fl
RorccreAtg5fl/fl
RORγt
5.47
1.64
8.01
2.69
CD4
e
Atg5fl/fl
RorccreAtg5fl/fl
% of CD45+
Absolute numbers(*10^3)
ns
CD4+RORγt+
CD4-RORγt+
f
Gated on CD45+CD4-Lin-CD90.2+
Atg5fl/fl
RorccreAtg5fl/fl
RORγt
35.6
41.7
CD127
NKp46
11.7
69.3
18.9
12.5
66.3
20.9
CCR6
g
Atg5fl/fl
RorccreAtg5fl/fl
% among total ILCs
Absolute numbers(*10^3)
ns
Total ILC3s
DN ILC3s
CCR6+ ILC3s
NKp46+ ILC3s
h
Gated on ILC3s
Atg5fl/fl
RorccreAtg5fl/fl
IL-17A
11.1
17.4
RORγt
i
Atg5fl/fl
RorccreAtg5fl/fl
IL-17A+ ILC3s(%)
IL-17A+ ILC3(*10^2)
ns
l
Gated on CD45+CD4-Lin-CD90.2+
Atg5fl/fl
RorccreAtg5fl/fl
T-bet
4.45
8.28
CD127
m
Atg5fl/fl
RorccreAtg5fl/fl
ILC1s(%) among total ILCs
ILC1s number(*10^2)
ns
j
Gated on ILC3s
Atg5fl/fl
RorccreAtg5fl/fl
IL-22
14.6
21.9
RORγt
k
Atg5fl/fl
RorccreAtg5fl/fl
IL-22+ ILC3s(%)
IL-22+ ILC3(*10^2)
ns
n
Gated on CD45+CD4-Lin-CD90.2+
Atg5fl/fl
RorccreAtg5fl/fl
GATA3
13.2
15.7
CD127
o
Atg5fl/fl
RorccreAtg5fl/fl
ILC2s(%) among total ILCs
ILC2s number(*10^2)
ns

Supplementary Figure 18. *Atg5* deficiency does not alter the levels of intestinal ILCs and CD4$^+$RORγt$^+$ cells. a, **b** Flow cytometry histogram **(a)** and statistical analysis **(b)** of ATG5 expression in CD4$^+$RORγt$^+$ cells and ILC3s (*n* = 6 biological replicates per group). **c** Macroscopic image and H&E staining of the intestines (scale bar: 100 μm), with corresponding inflammation scores (*n* = 6 biological replicates per group). **d**, **e** Representative flow cytometry profiles **(d)** and percentage and absolute number **(e)** of intestinal CD4$^+$RORγt$^+$ and CD4$^-$RORγt$^+$ cells (*n* = 6 biological replicates per group). **f**, **g** Representative flow cytometry profiles **(f)** and percentage and absolute number **(g)** of intestinal ILC3 subsets (*n* = 6 biological replicates per group). **h**, **i** Representative flow cytometry profiles **(h)** and percentage and absolute number **(i)** of intestinal IL-17A$^+$ ILC3s (*n* = 6 biological replicates per group). **j**, **k** Representative flow cytometry profiles **(j)** and percentage and absolute number **(k)** of intestinal IL-22$^+$ ILC3s (*n* = 6 biological replicates per group). **l**, **m** Representative flow cytometry profiles **(l)** and percentage and absolute number **(m)** of intestinal ILC1s (*n* = 6 biological replicates per group). **n**, **o** Representative flow cytometry profiles **(n)** and percentage and absolute number **(o)** of intestinal ILC2s (*n* = 6 biological replicates per group). All experiments were performed using Atg5$^{fl/fl}$ and Rorc^{cre}Atg5$^{fl/fl}$ mice of both sexes at P8, with littermates randomly assigned to control and experimental groups. Each data point represents one biologically independent mouse, and results are representative of at least three independent experiments. Box plots show the median (centre line, 50th percentile), with the lower and upper bounds of the box representing the 25th and 75th percentiles, respectively. Whiskers extend to the absolute minimum and maximum values (0th and 100th percentiles, respectively) of the dataset. *P*-values were determined by unpaired two tailed Student's t tests (**b**, **c**, **e**, **g**, **i**, **k**, **m**, and **o**). ns = not significant. Source data are provided as a Source Data file.

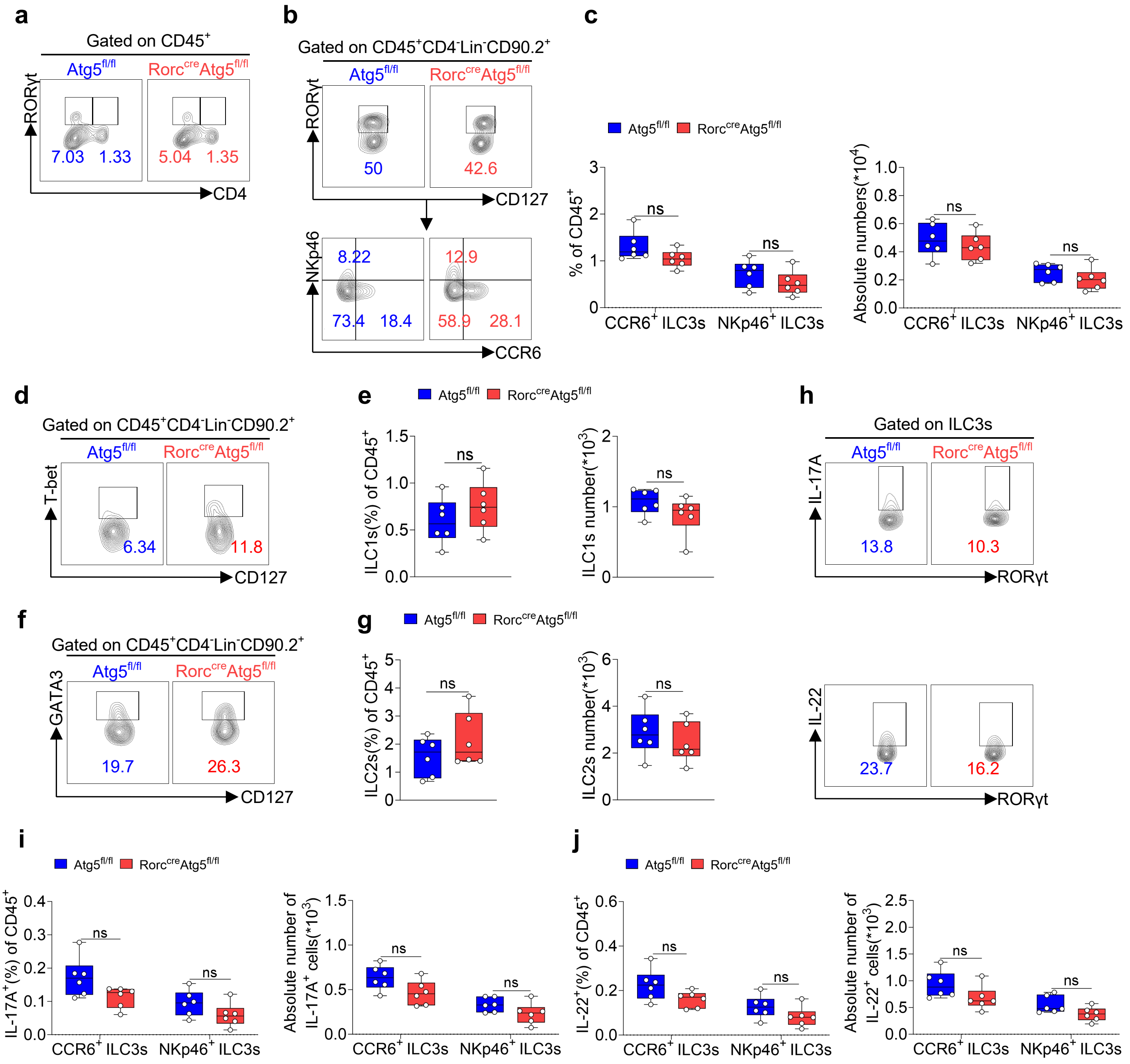
a
Gated on CD45+
Atg5fl/fl
RorccreAtg5fl/fl
RORγt
7.03 1.33
5.04 1.35
CD4
b
Gated on CD45+CD4-Lin-CD90.2+
Atg5fl/fl
RorccreAtg5fl/fl
RORγt
50
42.6
CD127
NKp46
8.22
73.4 18.4
12.9
58.9 28.1
CCR6
c
Atg5fl/fl RorccreAtg5fl/fl
% of CD45+
ns
ns
CCR6+ ILC3s NKp46+ ILC3s
Absolute numbers(*104)
ns
ns
CCR6+ ILC3s NKp46+ ILC3s
d
Gated on CD45+CD4-Lin-CD90.2+
Atg5fl/fl
RorccreAtg5fl/fl
T-bet
6.34
11.8
CD127
e
Atg5fl/fl RorccreAtg5fl/fl
ILC1s(%) of CD45+
ns
ILC1s number(*103)
ns
h
Gated on ILC3s
Atg5fl/fl
RorccreAtg5fl/fl
IL-17A
13.8
10.3
RORγt
f
Gated on CD45+CD4-Lin-CD90.2+
Atg5fl/fl
RorccreAtg5fl/fl
GATA3
19.7
26.3
CD127
g
Atg5fl/fl RorccreAtg5fl/fl
ILC2s(%) of CD45+
ns
ILC2s number(*103)
ns
IL-22
23.7
16.2
RORγt
i
Atg5fl/fl RorccreAtg5fl/fl
IL-17A+(%) of CD45+
ns
ns
CCR6+ ILC3s NKp46+ ILC3s
Absolute number of IL-17A+ cells(*103)
ns
ns
CCR6+ ILC3s NKp46+ ILC3s
j
Atg5fl/fl RorccreAtg5fl/fl
IL-22+(%) of CD45+
ns
ns
CCR6+ ILC3s NKp46+ ILC3s
Absolute number of IL-22+ cells(*103)
ns
ns
CCR6+ ILC3s NKp46+ ILC3s

Supplementary Figure 19. ***Atg5*** **deficiency does not alter the levels of intestinal NKp46⁺ ILC3s, CCR6⁺ ILC3s, ILC1s, and ILC2s. a** Representative flow cytometry profiles of intestinal $CD4^{+}ROR\gamma t^{+}$ and $CD4^{-}ROR\gamma t^{+}$ cells. **b**, **c** Representative flow cytometry profiles **(b)** and percentage and absolute number **(c)** of intestinal $CCR6^{+}$ and $NKp46^{+}$ ILC3s (*n* = 6 biological replicates per group). **d**, **e** Representative flow cytometry **(d)** and percentage and absolute number **(e)** of intestinal ILC1s (*n* = 6 biological replicates per group). **f**, **g** Representative flow cytometry profiles **(f)** and percentage and absolute number **(g)** of intestinal ILC2s (*n* = 6 biological replicates per group). **h** Representative flow cytometry profiles of intestinal IL-$17A^{+}$ and IL-22^{+} ILC3s. **i** Percentage and absolute number of IL-$17A^{+}CCR6^{+}$ and IL-$17A^{+}$ $NKp46^{+}$ ILC3s (*n* = 6 biological replicates per group). **j** Percentage and absolute number of IL-$22^{+}CCR6^{+}$ and IL-$22^{+}NKp46^{+}$ ILC3s (*n* = 6 biological replicates per group). All experiments were performed using $Atg5^{fl/fl}$ and $Rorc^{cre}Atg5^{fl/fl}$ mice of both sexes at P8, with littermates randomly assigned to control and experimental groups. Each data point represents one biologically independent mouse, and results are representative of at least three independent experiments. Box plots show the median (centre line, 50th percentile), with the lower and upper bounds of the box representing the 25th and 75th percentiles, respectively. Whiskers extend to the absolute minimum and maximum values (0th and 100th percentiles, respectively) of the dataset. *P*-values were determined by unpaired two tailed Student's t tests (**c**, **e**, **g**, **i**, and **j**). ns = not significant. Source data are provided as a Source Data file.

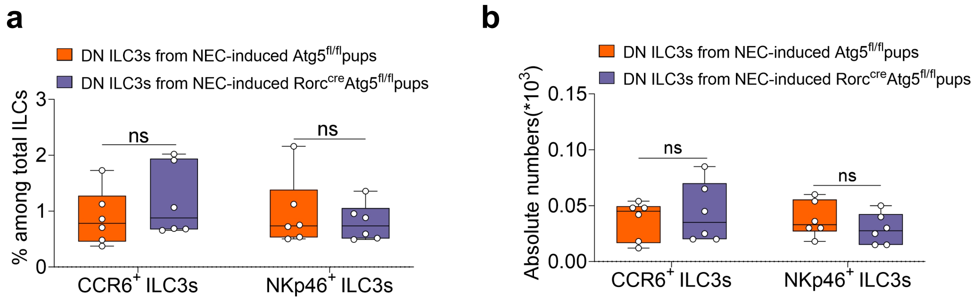

Supplementary Figure 20. Adoptive transfer of *Atg5*-deficient DN ILC3s does not alter the abundances of CCR6⁺ and NKp46⁺ ILC3s in *Nfil3*-deficient mice. a, **b** Percentage **(a)** and absolute number **(b)** of intestinal CCR6⁺ ILC3s and NKp46⁺ ILC3s (n = 6 biological replicates per group). All experiments were performed using Nfil3$^{-/-}$ mice of both sexes at P8, with littermates randomly assigned to control and experimental groups. Each data point represents one biologically independent mouse, and results are representative of at least three independent experiments. Box plots show the median (centre line, 50th percentile), with the lower and upper bounds of the box representing the 25th and 75th percentiles, respectively. Whiskers extend to the absolute minimum and maximum values (0th and 100th percentiles, respectively) of the dataset. *P*-values were determined by unpaired two tailed Student's t tests (**a** and **b**). ns = not significant. Source data are provided as a Source Data file.

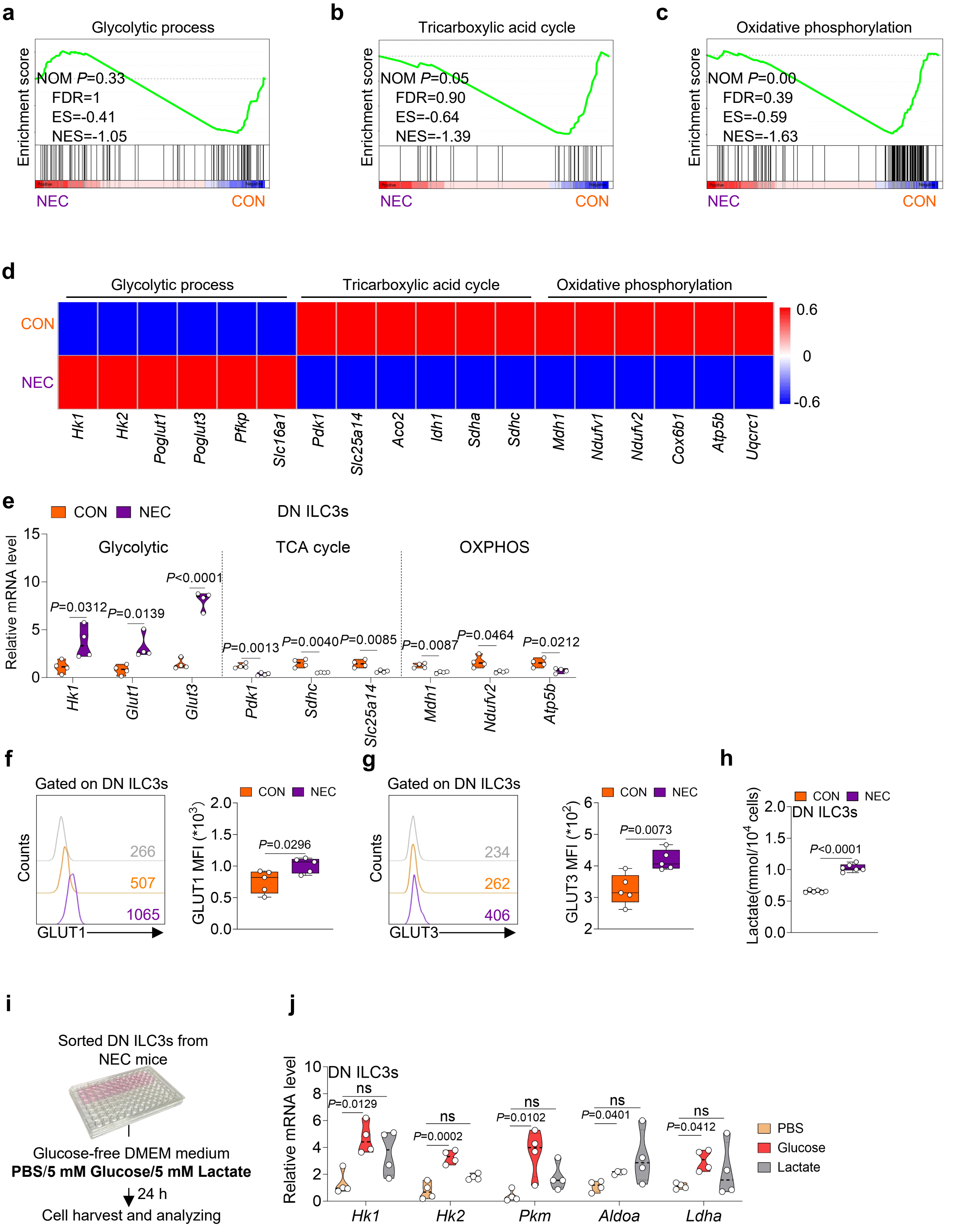

a
Glycolytic process
Enrichment score
NOM P=0.33
FDR=1
ES=-0.41
NES=-1.05
NEC
CON
b
Tricarboxylic acid cycle
Enrichment score
NOM P=0.05
FDR=0.90
ES=-0.64
NES=-1.39
NEC
CON
c
Oxidative phosphorylation
Enrichment score
NOM P=0.00
FDR=0.39
ES=-0.59
NES=-1.63
NEC
CON
d
Glycolytic process
Tricarboxylic acid cycle
Oxidative phosphorylation
CON
NEC
0.6
0
-0.6
Hk1 Hk2 Poglut1 Poglut3 Pfkp Slc16a1 Pdk1 Slc25a14 Aco2 Idh1 Sdha Sdhc Mdh1 Ndufv1 Ndufv2 Cox6b1 Atp5b Uqcrc1
e
CON NEC
DN ILC3s
Relative mRNA level
Glycolytic
TCA cycle
OXPHOS
P=0.0312
P=0.0139
P<0.0001
P=0.0013
P=0.0040
P=0.0085
P=0.0087
P=0.0464
P=0.0212
Hk1 Glut1 Glut3 Pdk1 Sdhc Slc25a14 Mdh1 Ndufv2 Atp5b
f
Gated on DN ILC3s
Counts
266
507
1065
GLUT1
CON NEC
GLUT1 MFI (*10³)
P=0.0296
g
Gated on DN ILC3s
Counts
234
262
406
GLUT3
CON NEC
GLUT3 MFI (*10²)
P=0.0073
h
CON NEC
DN ILC3s
Lactate(mmol/10⁴ cells)
P<0.0001
i
Sorted DN ILC3s from NEC mice
Glucose-free DMEM medium
PBS/5 mM Glucose/5 mM Lactate
24 h
Cell harvest and analyzing
j
DN ILC3s
Relative mRNA level
ns
P=0.0129
P=0.0002
P=0.0102
P=0.0401
P=0.0412
Hk1 Hk2 Pkm Aldoa Ldha
PBS
Glucose
Lactate

Supplementary Figure 21. Glycolysis-related genes are upregulated in DN ILC3s during NEC. **a−c** Gene Set Enrichment Analysis (GSEA) demonstrating downregulation in the glycolytic process **(a)**; tricarboxylic acid (TCA) cycle **(b)**; and oxidative phosphorylation (OXPHOS) **(c)** of NEC DN ILC3s. **d** Heatmap showing the expression profiles of the genes related to glycolytic process, TCA cycle, and OXPHOS in DN ILC3s. **e** Relative mRNA levels of the indicated genes in DN ILC3s (n = 4 biological replicates per group). **f** Protein level of GLUT1 in DN ILC3s (n = 5 biological replicates per group). **g** Protein level of GLUT3 in DN ILC3s (n = 5 biological replicates per group). **h** Statistical analysis of lactate concentrations (n = 6 biological replicates per group). **i** DN ILC3s were treated with PBS, 5 mM glucose, or 5 mM lactate for 24 h. **j** Relative mRNA levels of *Hk1*, *Hk2*, *Pkm*, *Aldoa*, and *Ldha* in DN ILC3s under the culture system (n = 4 biological replicates per group). All experiments were performed using C57BL/6 mice of both sexes at P8, with littermates randomly assigned to control and experimental groups. Each data point represents one biologically independent mouse, and results are representative of at least three independent experiments. Box plots show the median (centre line, 50th percentile), with the lower and upper bounds of the box representing the 25th and 75th percentiles, respectively. Whiskers extend to the absolute minimum and maximum values (0th and 100th percentiles, respectively) of the dataset. *P*-values were determined by unpaired two tailed Student’s t tests (**e−h**) or one-way ANOVA followed by Tukey-Kramer multiple comparisons test (two-sided) (**j**). ns = not significant. Source data are provided as a Source Data file.

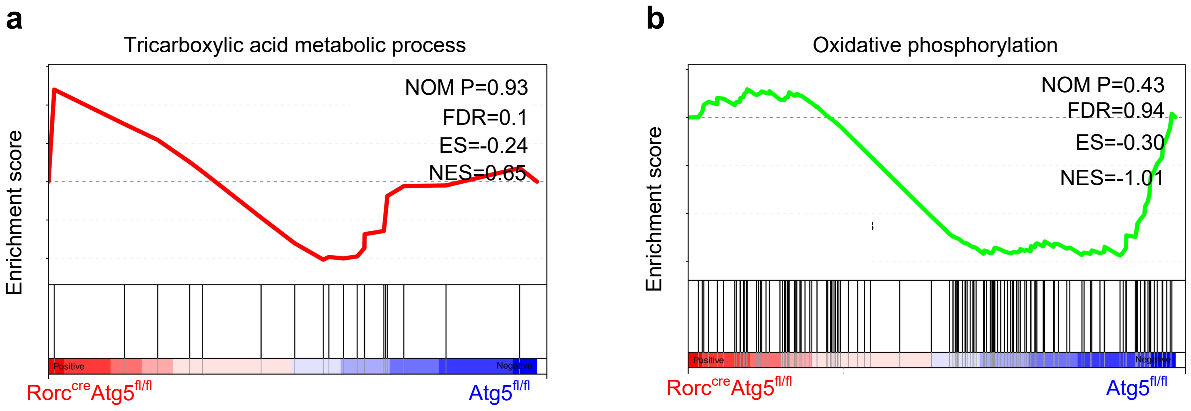

Supplementary Figure 22. *Atg5* deficiency does not alter the activity of OXPHOS and TCA cycle in DN ILC3s. a, b GSEA plots demonstrating the enrichment of TCA cycle **(a)** and OXPHOS **(b)** in DN ILC3s from $Rorc^{cre}Atg5^{fl/fl}$ versus $Atg5^{fl/fl}$ mice. GSEA was performed on bulk RNA-seq data using 1,000 one-sided phenotype-based permutation tests. Genes were ranked by signal-to-noise ratio, and multiple comparisons were adjusted via Benjamini-Hochberg FDR calculation. Significance was defined as nominal $P < 0.05$ and FDR < 0.25.

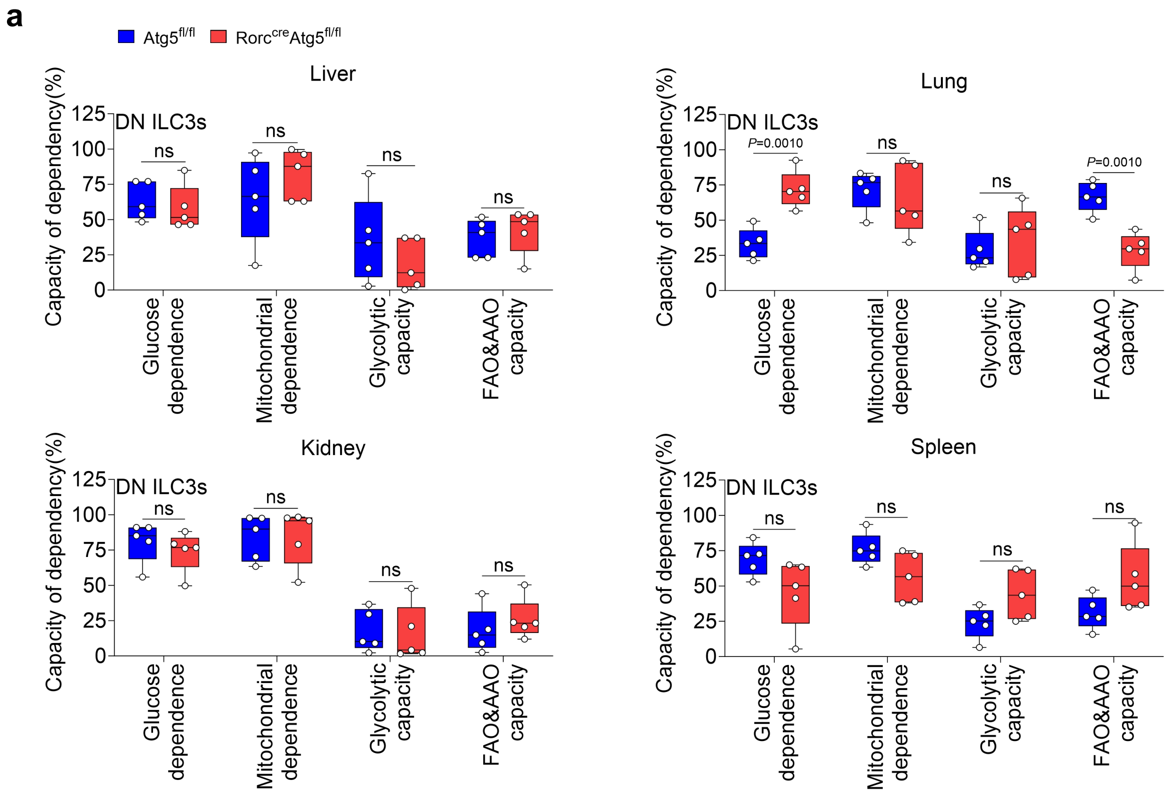

Supplementary Figure 23. The metabolic dependency profiles of DN ILC3s from different tissues. a Glucose dependency, mitochondrial dependency, glycolysis activity, and FAO and AAO in liver, lung, kidney, and splenic DN ILC3s (*n* = 5 biological replicates per group). All experiments were performed using $Atg5^{fl/fl}$ and $Rorc^{cre}Atg5^{fl/fl}$ mice of both sexes at P8, with littermates randomly assigned to control and experimental groups. Each data point represents one biologically independent mouse, and results are representative of at least three independent experiments. Box plots show the median (centre line, 50th percentile), with the lower and upper bounds of the box representing the 25th and 75th percentiles, respectively. Whiskers extend to the absolute minimum and maximum values (0th and 100th percentiles, respectively) of the dataset. *P*-values were determined by unpaired two tailed Student's t tests (**a**). ns = not significant. Source data are provided as a Source Data file.

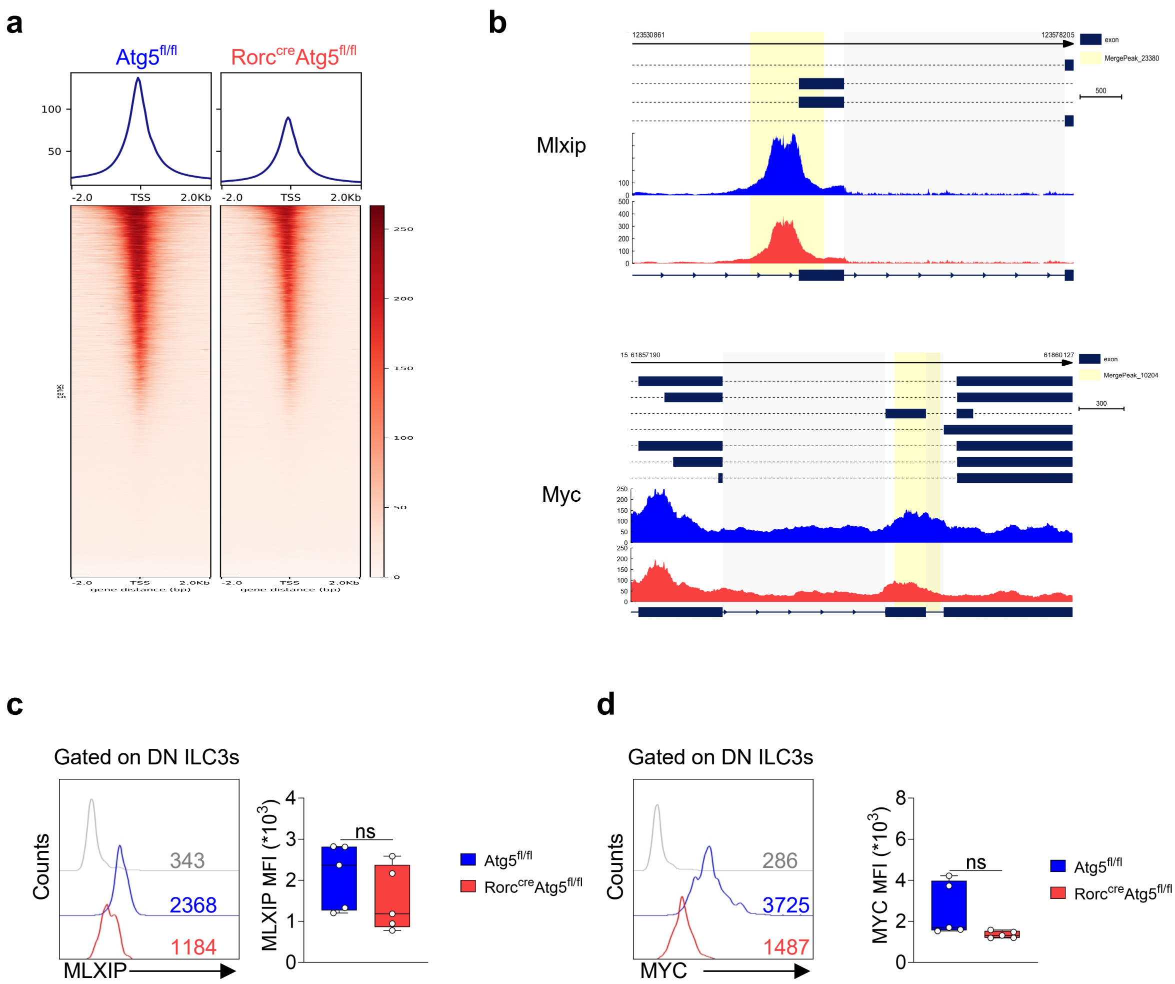

Supplementary Figure 24. *Atg5* deficiency in DN ILC3s does not affect Mlxip and Myc expression. a Aggregate plots and heatmap showing chromatin accessibility around transcription start sites (TSSs) in DN ILC3s isolated from Atg5$^{fl/fl}$ and Rorc^{cre}Atg5$^{fl/fl}$ mice. The aggregate plots displaying the mean ATAC-seq signal intensity across all TSS regions, while the heatmap showing the individual signal distribution for each gene. **b** Genome browser tracks showing chromatin accessibility at the Mlxip and Myc loci. Exons are depicted as black rectangles, and the highlighted yellow regions indicate differential accessible peaks. **c**, **d** Protein levels of MLXIP **(c)** and MYC **(d)** in DN ILC3s (n = 5 biological replicates per group). All experiments were performed using Atg5$^{fl/fl}$ and Rorc^{cre}Atg5$^{fl/fl}$ mice of both sexes at P8, with littermates randomly assigned to control and experimental groups. Each data point represents one biologically independent mouse, and results are representative of at least three independent experiments. Box plots show the median (centre line, 50th percentile), with the lower and upper bounds of the box representing the 25th and 75th percentiles, respectively. Whiskers extend to the absolute minimum and maximum values (0th and 100th percentiles, respectively) of the dataset. *P*-values were determined by unpaired two tailed Student's t tests (**c** and **d**). ns = not significant. Source data are provided as a Source Data file.

a

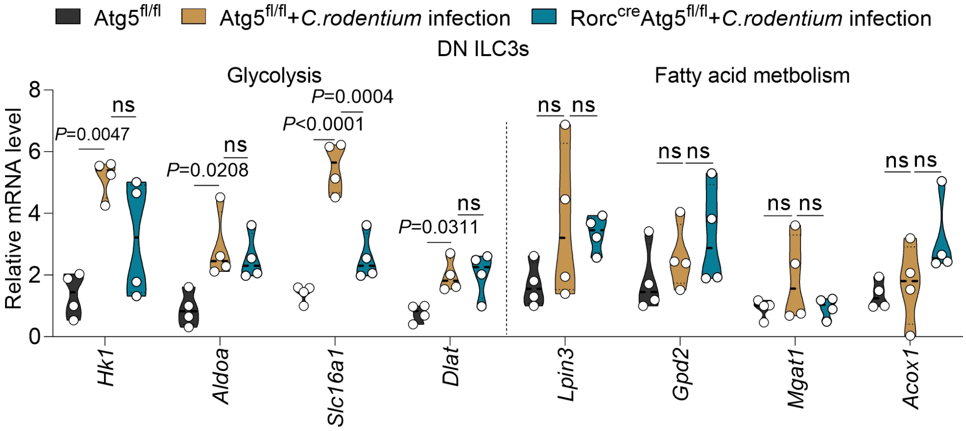

Supplementary Figure 25. *Atg5* deficiency in DN ILC3s impairs the expression of glycolysis-related rather than fatty acid metabolism-related genes in *Citrobacter rodentium* infection models. a Relative mRNA levels of glycolysis- and fatty acid metabolism-related genes in DN ILC3s (n = 4 biological replicates per group). Experiments were performed using Atg5$^{fl/fl}$ and Rorc^{cre}Atg5$^{fl/fl}$ mice of both sexes at 8 weeks, with randomly assigned to control and experimental groups. Each data point represents three biologically independent mouse, and results are representative of at least three independent experiments. Data are presented as violin plots (showing the kernel density distribution of the dataset) overlaid with box-and-whisker plots and all individual data points. For box-and-whisker plots: the center line indicates the median (50th percentile); box limits represent the 25th and 75th percentiles (upper and lower quartiles); whiskers extend to 1.5 × the interquartile range (IQR). *P*-values were determined by one-way ANOVA followed by Tukey-Kramer multiple comparisons test (two-sided) (**a**). ns = not significant. Source data are provided as a Source Data file.

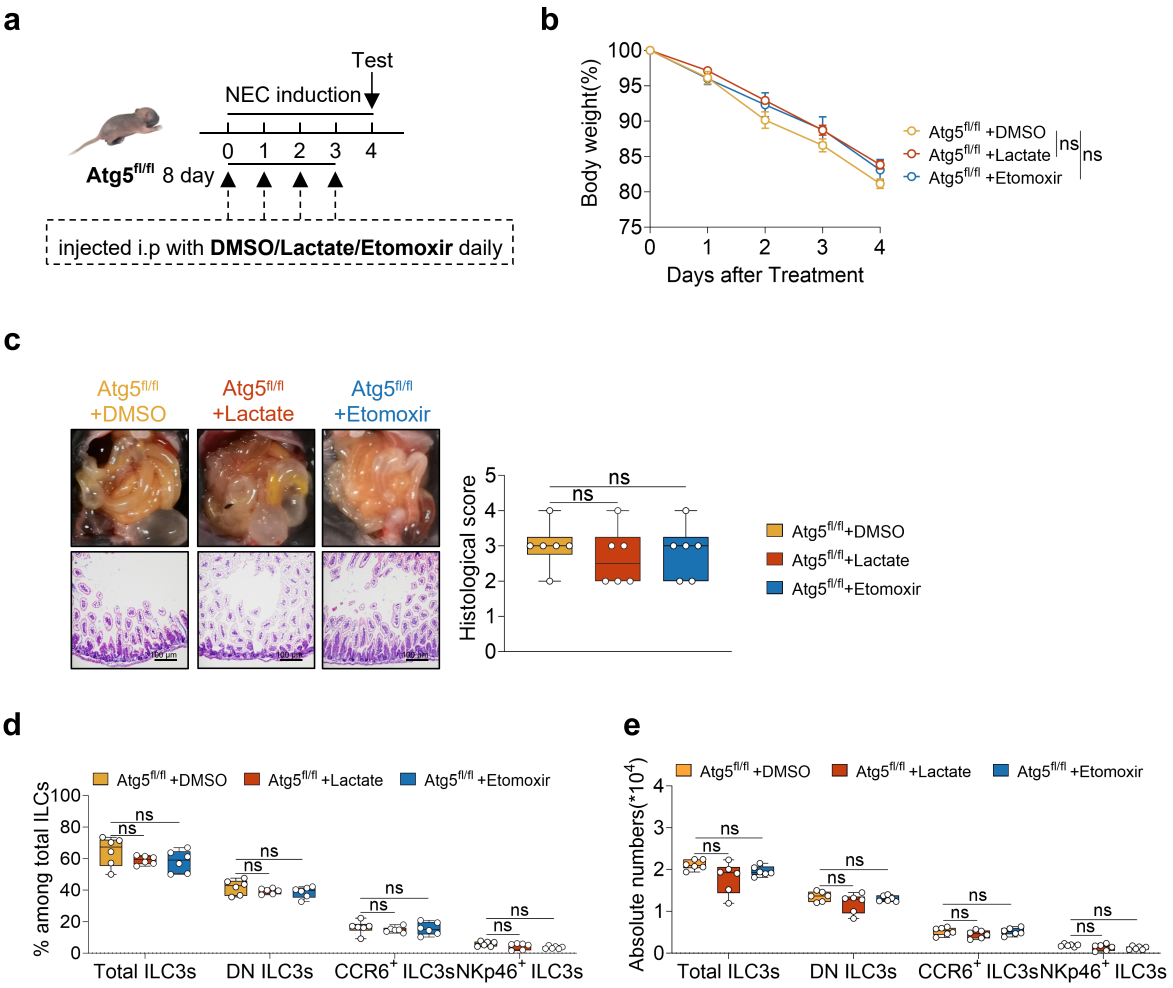

Supplementary Figure 26. Metabolic interventions have no effects on intestinal inflammation and the levels of ILC3 subsets in Atg5$^{fl/fl}$ mice. a Schematic diagram of metabolic interventions in Atg5$^{fl/fl}$ mice during NEC. **b** Body weight changes in the indicated groups (*n* = 6 biological replicates per group). Data are presented as mean ± SEM. **c** Macroscopic image and H&E staining of the intestines (scale bar: 100 μm), with corresponding inflammation scores (*n* = 6 biological replicates per group). **d**, **e** Percentage **(d)** and absolute number **(e)** of total ILC3s and ILC3 subset (*n* = 6 biological replicates per group). All experiments were performed using Atg5$^{fl/fl}$ mice of both sexes at P8, with littermates randomly assigned to control and experimental groups. Each data point represents one biologically independent mouse, and results are representative of at least three independent experiments. Box plots show the median (centre line, 50th percentile), with the lower and upper bounds of the box representing the 25th and 75th percentiles, respectively. Whiskers extend to the absolute minimum and maximum values (0th and 100th percentiles, respectively) of the dataset. *P*-values were determined by one-way ANOVA followed by Tukey-Kramer multiple comparisons test (two-sided) (**b-e**). ns = not significant. Source Data are provided as a Source Data file.

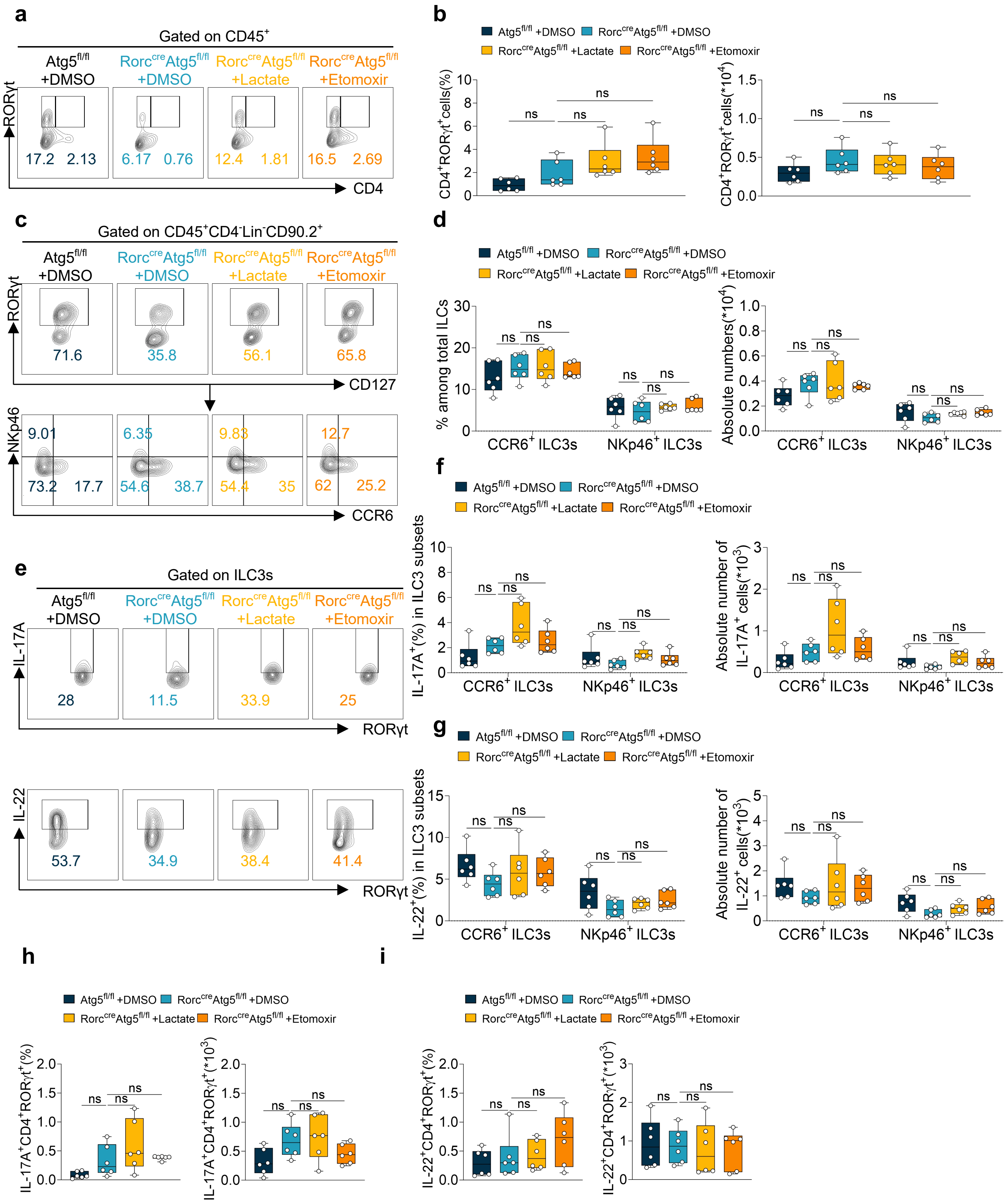
a
Gated on CD45+
Atg5fl/fl +DMSO
RorccreAtg5fl/fl +DMSO
RorccreAtg5fl/fl +Lactate
RorccreAtg5fl/fl +Etomoxir
RORγt
CD4
17.2 2.13
6.17 0.76
12.4 1.81
16.5 2.69
b
CD4+RORγt+cells(%)
CD4+RORγt+cells(*10^4)
ns
c
Gated on CD45+CD4-Lin-CD90.2+
RORγt
CD127
71.6
35.8
56.1
65.8
NKp46
CCR6
9.01 73.2 17.7
6.35 54.6 38.7
9.83 54.4 35
12.7 62 25.2
d
% among total ILCs
Absolute numbers(*10^4)
CCR6+ ILC3s
NKp46+ ILC3s
e
Gated on ILC3s
IL-17A
RORγt
28
11.5
33.9
25
IL-22
53.7
34.9
38.4
41.4
f
IL-17A+(%) in ILC3 subsets
Absolute number of IL-17A+ cells(*10^3)
g
IL-22+(%) in ILC3 subsets
Absolute number of IL-22+ cells(*10^3)
h
IL-17A+CD4+RORγt+(%)
IL-17A+CD4+RORγt+(*10^3)
i
IL-22+CD4+RORγt+(%)
IL-22+CD4+RORγt+(*10^3)

Supplementary Figure 27. Metabolic interventions have no effects on the levels of NKp46$^+$ ILC3s, CCR6$^+$ ILC3s, and CD4$^+$RORγt$^+$ cells in *Atg5*-deficient mice. a, **b** Representative flow cytometry profiles **(a)** and percentage and absolute number **(b)** of intestinal CD4$^+$RORγt$^+$ cells (*n* = 6 biological replicates per group). **c** Representative flow cytometry profiles of intestinal total ILC3s, DN ILC3s, CCR6$^+$ ILC3s, and NKp46$^+$ ILC3s. **d** Percentage and absolute number of intestinal CCR6$^+$ and NKp46$^+$ ILC3s (*n* = 6 biological replicates per group). **e** Representative flow cytometry profiles of intestinal IL-17A$^+$ and IL-22$^+$ ILC3s. **f** Percentage and absolute number of intestinal IL-17A$^+$CCR6$^+$ and IL-17A$^+$ NKp46$^+$ ILC3s (*n* = 6 biological replicates per group). **g** Percentage and absolute number of intestinal IL-22$^+$CCR6$^+$ and IL-22$^+$NKp46$^+$ ILC3s (*n* = 6 biological replicates per group). **h** Percentage and absolute number of intestinal IL-17A$^+$CD4$^+$RORγt$^+$ cells (*n* = 6 biological replicates per group). **i** Percentage and absolute number of intestinal IL-22$^+$CD4$^+$RORγt$^+$ cells (*n* = 6 biological replicates per group). All experiments were performed using Atg5$^{fl/fl}$ and Rorc^{cre}Atg5$^{fl/fl}$ mice of both sexes at P8, with littermates randomly assigned to control and experimental groups. Each data point represents one biologically independent mouse, and results are representative of at least three independent experiments. Box plots show the median (centre line, 50th percentile), with the lower and upper bounds of the box representing the 25th and 75th percentiles, respectively. Whiskers extend to the absolute minimum and maximum values (0th and 100th percentiles, respectively) of the dataset. *P*-values were determined by one-way ANOVA followed by Tukey-Kramer multiple comparisons test (two-sided) (**b**, **d**, and **f–i**). ns = not significant. Source data are provided as a Source Data file.

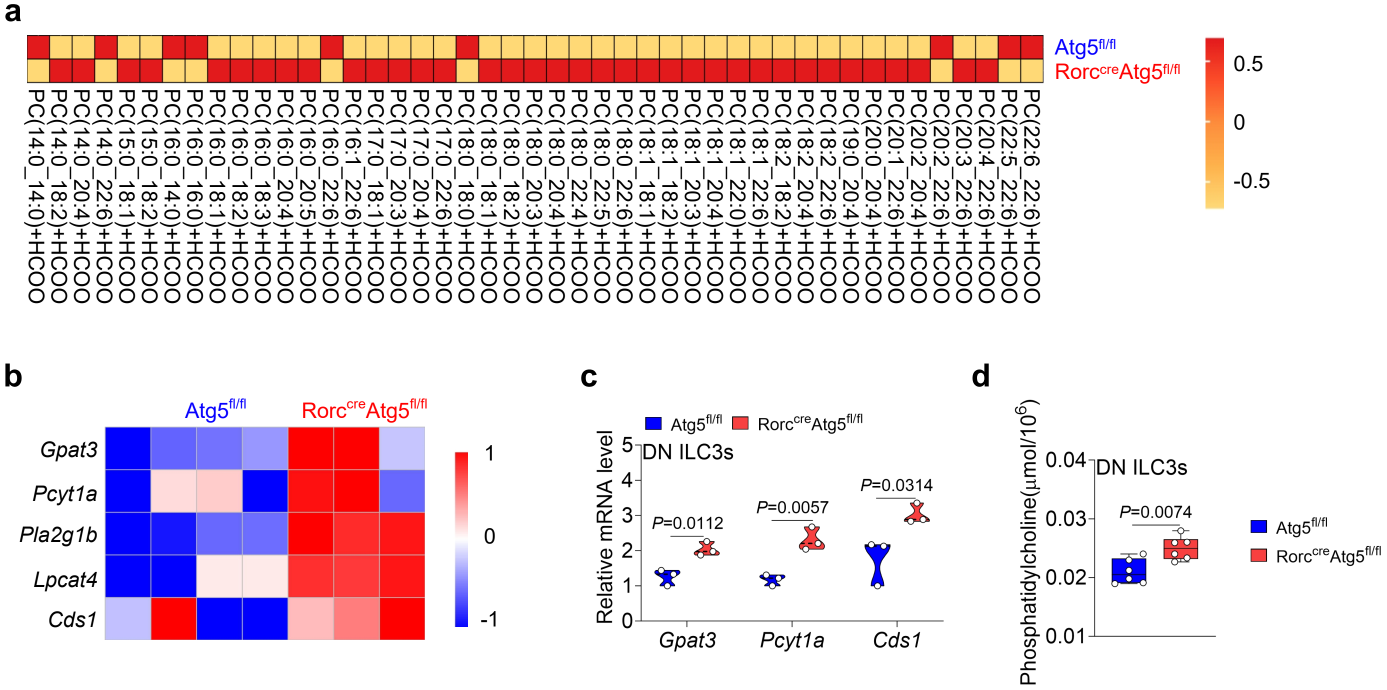

Supplementary Figure 28. *Atg5* deficiency rewires lipid metabolism and phosphatidylcholine synthesis in DN ILC3s. a Heatmap depicting the changes in lipid metabolites between Rorc^{cre}Atg5$^{fl/fl}$ and Atg5$^{fl/fl}$ mice (isolated from 14 neonates per group). **b** Heatmap showing the expression profiles of glycerophospholipid metabolism in DN ILC3s of Atg5$^{fl/fl}$ mice versus Rorc^{cre}Atg5$^{fl/fl}$ mice. **c** Relative mRNA levels of *Gpat3*, *Pcyt1a*, and *Cds1* in intestinal DN ILC3s (n = 3 biological replicates per group). **d** Statistical analysis of phosphatidylcholine concentrations in DN ILC3s (n = 6 biological replicates per group). All experiments were performed using Atg5$^{fl/fl}$ and Rorc^{cre}Atg5$^{fl/fl}$ mice of both sexes at P8, with littermates randomly assigned to control and experimental groups. Each data point represents one biologically independent mouse, and results are representative of at least three independent experiments. Box plots show the median (centre line, 50th percentile), with the lower and upper bounds of the box representing the 25th and 75th percentiles, respectively. Whiskers extend to the absolute minimum and maximum values (0th and 100th percentiles, respectively) of the dataset. *P*-values were determined by unpaired two tailed Student's t tests (**c** and **d**). ns = not significant. Source data are provided as a Source Data file.

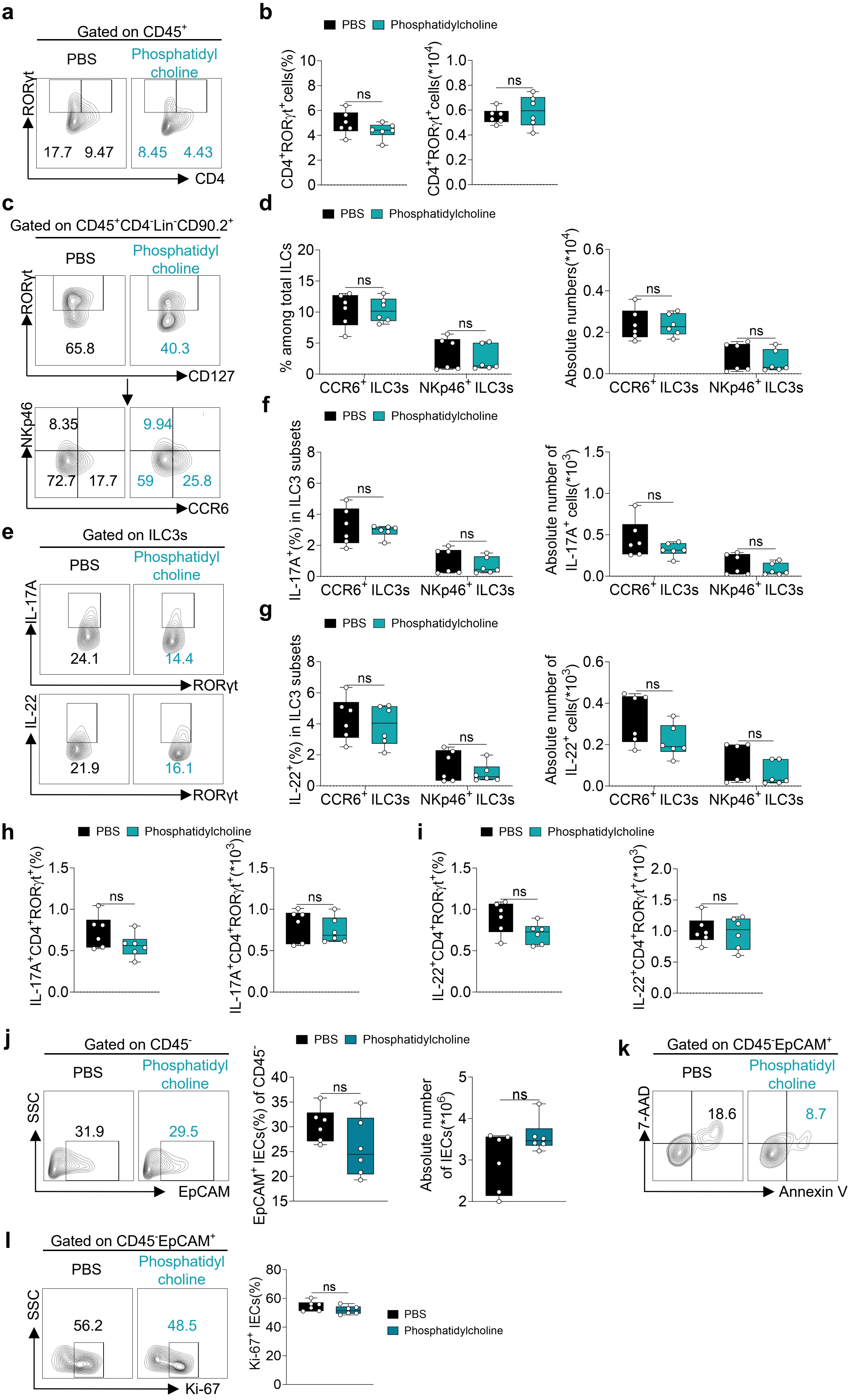
a
Gated on CD45^{+}
PBS
Phosphatidyl choline
RORγt
CD4
17.7 9.47
8.45 4.43
b
PBS Phosphatidylcholine
CD4^{+}RORγt^{+}cells(%)
CD4^{+}RORγt^{+}cells(*10^{4})
ns
c
Gated on CD45^{+}CD4^{-}Lin^{-}CD90.2^{+}
PBS
Phosphatidyl choline
RORγt
CD127
65.8
40.3
NKp46
CCR6
8.35
72.7 17.7
9.94
59 25.8
d
PBS Phosphatidylcholine
% among total ILCs
Absolute numbers(*10^{4})
CCR6^{+} ILC3s NKp46^{+} ILC3s
ns
e
Gated on ILC3s
PBS
Phosphatidyl choline
IL-17A
RORγt
24.1
14.4
IL-22
21.9
16.1
f
PBS Phosphatidylcholine
IL-17A^{+}(%) in ILC3 subsets
Absolute number of IL-17A^{+} cells(*10^{3})
CCR6^{+} ILC3s NKp46^{+} ILC3s
ns
g
PBS Phosphatidylcholine
IL-22^{+}(%) in ILC3 subsets
Absolute number of IL-22^{+} cells(*10^{3})
CCR6^{+} ILC3s NKp46^{+} ILC3s
ns
h
PBS Phosphatidylcholine
IL-17A^{+}CD4^{+}RORγt^{+}(%)
IL-17A^{+}CD4^{+}RORγt^{+}(*10^{3})
ns
i
PBS Phosphatidylcholine
IL-22^{+}CD4^{+}RORγt^{+}(%)
IL-22^{+}CD4^{+}RORγt^{+}(*10^{3})
ns
j
Gated on CD45^{-}
PBS
Phosphatidyl choline
SSC
EpCAM
31.9
29.5
PBS Phosphatidylcholine
EpCAM^{+} IECs(%) of CD45^{-}
Absolute number of IECs(*10^{6})
ns
k
Gated on CD45^{-}EpCAM^{+}
PBS
Phosphatidyl choline
7-AAD
Annexin V
18.6
8.7
l
Gated on CD45^{-}EpCAM^{+}
PBS
Phosphatidyl choline
SSC
Ki-67
56.2
48.5
Ki-67^{+} IECs(%)
ns
PBS
Phosphatidylcholine

Supplementary Figure 29. Phosphatidylcholine supplementation has no effects on the levels of intestinal NKp46$^+$ ILC3s, CCR6$^+$ ILC3s, CD4$^+$RORγt$^+$ cells, and IECs. **a**, **b** Representative flow cytometry profiles **(a)** and percentage and absolute number **(b)** of intestinal CD4$^+$RORγt$^+$ cells (*n* = 6 biological replicates per group). **c** Representative flow cytometry profiles of intestinal total ILC3s, DN ILC3s, CCR6$^+$ ILC3s, and NKp46$^+$ ILC3s. **d** Percentage and absolute number of intestinal CCR6$^+$ and NKp46$^+$ ILC3s (*n* = 6 biological replicates per group). **e** Representative flow cytometry profiles of intestinal IL-17A$^+$ and IL-22$^+$ ILC3s. **f** Percentage and absolute number of intestinal IL-17A$^+$CCR6$^+$ and IL-17A$^+$NKp46$^+$ ILC3s (*n* = 6 biological replicates per group). **g** Percentage and absolute number of intestinal IL-22$^+$CCR6$^+$ and IL-22$^+$NKp46$^+$ ILC3s (*n* = 6 biological replicates per group). **h**, **i** Percentage and absolute number of intestinal IL-17A$^+$CD4$^+$RORγt$^+$ **(h)** and IL-22$^+$CD4$^+$RORγt$^+$ cells **(i)** (*n* = 6 biological replicates per group). **j** Representative flow cytometry profiles and proportion and absolute number of IECs (*n* = 6 biological replicates per group). **k** Representative flow cytometry profiles of Annexin V and 7-AAD staining in IECs (*n* = 6 biological replicates per group). **l** Representative flow cytometry profiles of Ki67$^+$ IECs (*n* = 6 biological replicates per group). All experiments were performed using C57BL/6 mice of both sexes at P8, with littermates randomly assigned to control and experimental groups. Each data point represents one biologically independent mouse, and results are representative of at least three independent experiments. Box plots show the median (centre line, 50th percentile), with the lower and upper bounds of the box representing the 25th and 75th percentiles, respectively. Whiskers extend to the absolute minimum and maximum values (0th and 100th percentiles, respectively) of the dataset. *P*-values were determined by unpaired two tailed Student's t tests (**b**, **d**, **f-j**, and **l**). ns = not significant. Source data are provided as a Source Data file.

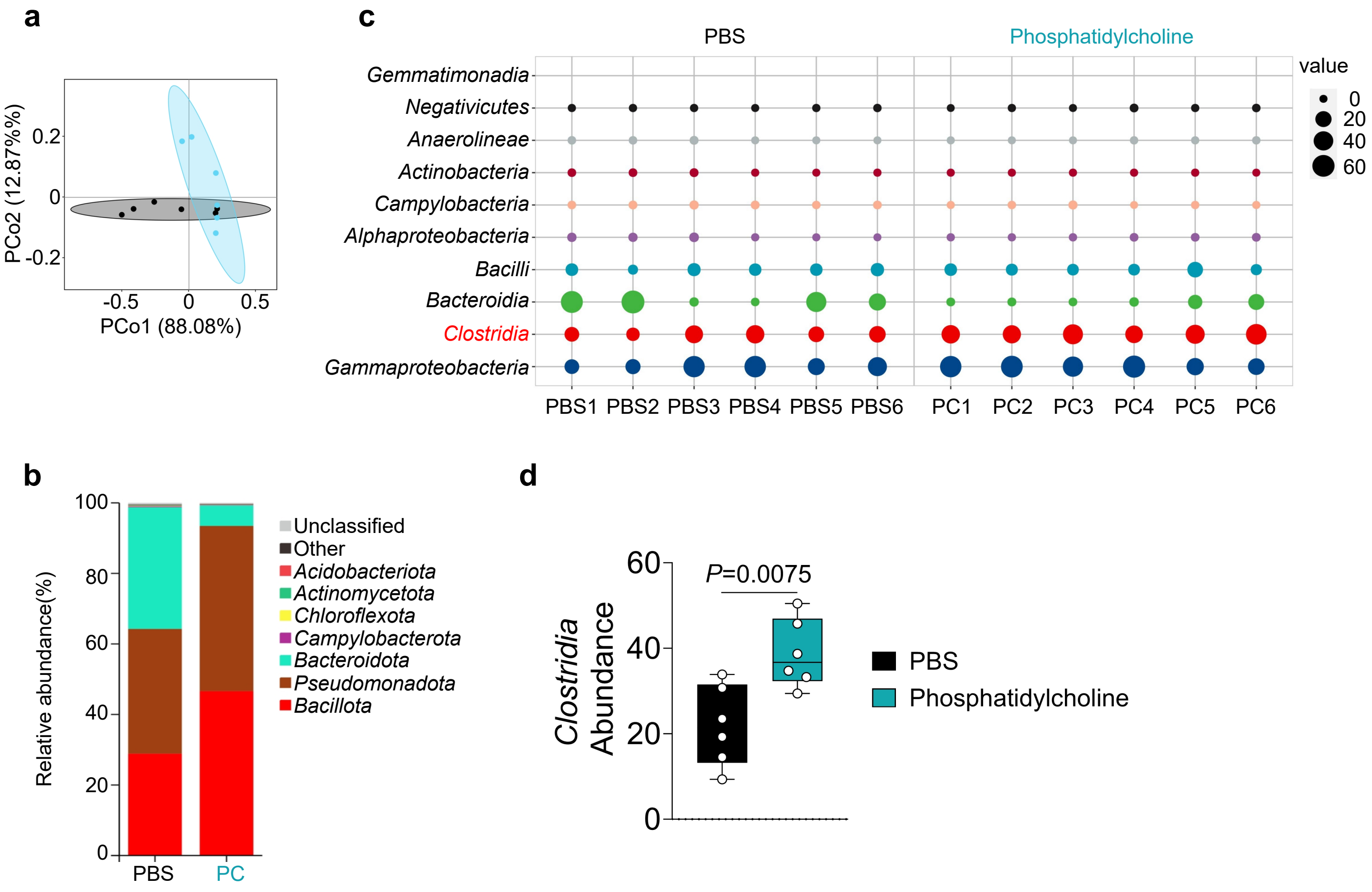

Supplementary Figure 30. Phosphatidylcholine supplementation alters the gut microbiota composition in NEC, with a significant enrichment in *Clostridium* species. a Principal component analysis (PCA) of microbial community profiles between the PBS- and PC-treated groups. **b** Stacked bar chart illustrating the relative abundance of the top bacterial phyla. **c** Dot plot showing the relative abundance of the top 10 bacterial taxa at the order/class levels. **d** *Clostridia* abundance in NEC mice treated with PBS or phosphatidylcholine PC (n = 6 biological replicates per group). Experiments were performed using C57BL/6 mice of both sexes at P8, with littermates randomly assigned to control and experimental groups. Each data point represents one biologically independent mouse, and results are representative of at least three independent experiments. Box plots show the median (centre line, 50th percentile), with the lower and upper bounds of the box representing the 25th and 75th percentiles, respectively. Whiskers extend to the absolute minimum and maximum values (0th and 100th percentiles, respectively) of the dataset. *P*-values were determined by unpaired two tailed Student's t tests (**d**). Source data are provided as a Source Data file.

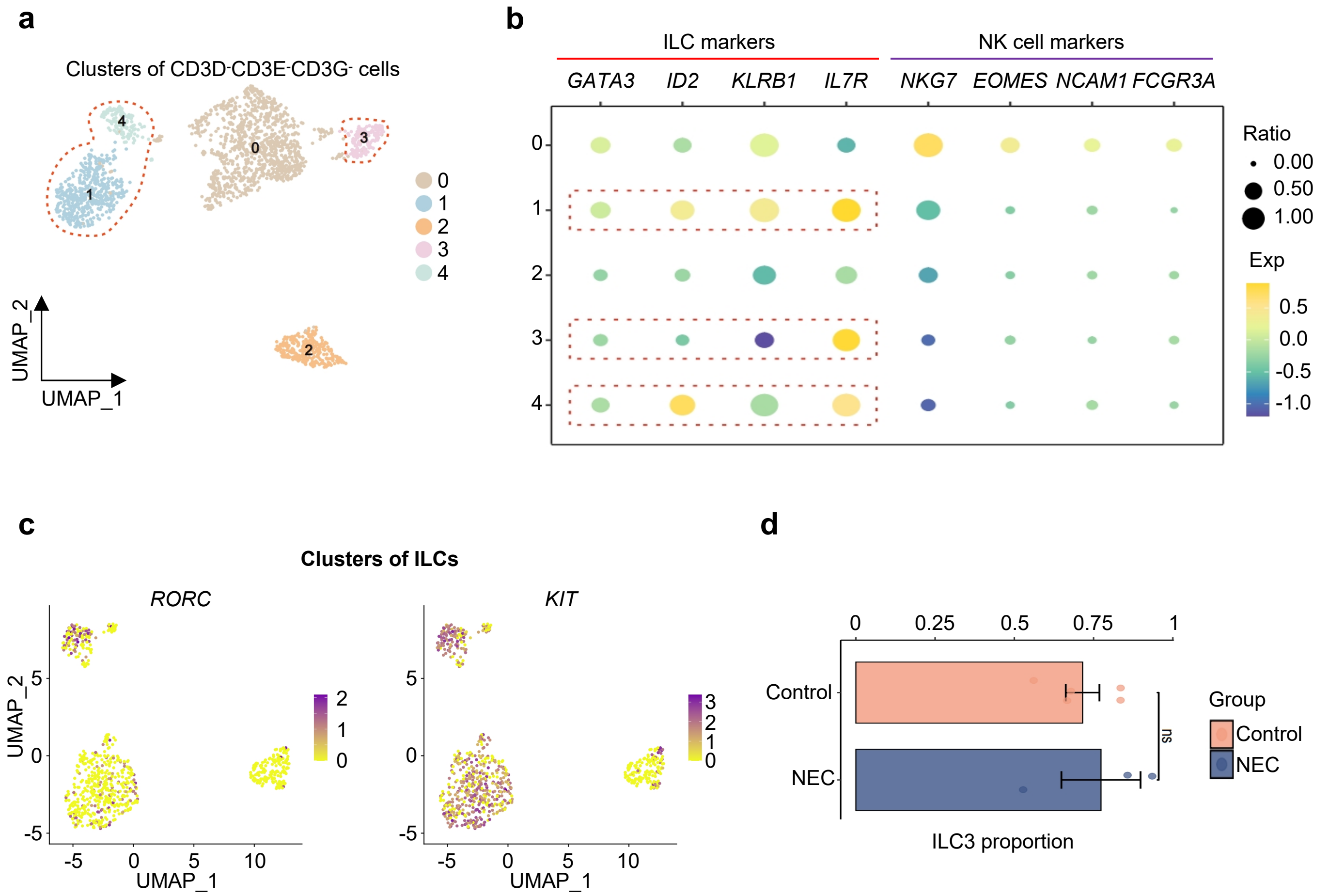

Supplementary Figure 31. Single-cell transcriptomic profiling identifies ILC3s as a predominant ILC subset in neonatal patients with NEC. a Uniform Manifold Approximation and Projection (UMAP) visualization of $CD3^-$ $CD3E^-$ $CD3G^-$ ILCs. **b** Bubble plot showing the expression of canonical ILC and NK cell markers across the identified clusters. **c** UMAP plots showing the expression of *RORC* and *KIT* within the ILC clusters. **d** Box-and-whisker plot comparing the proportion of ILC3s in control and NEC patients. Data are presented as the mean ± SEM. *P*-values were determined by unpaired two tailed Student's t tests (**d**). ns = not significant.

a

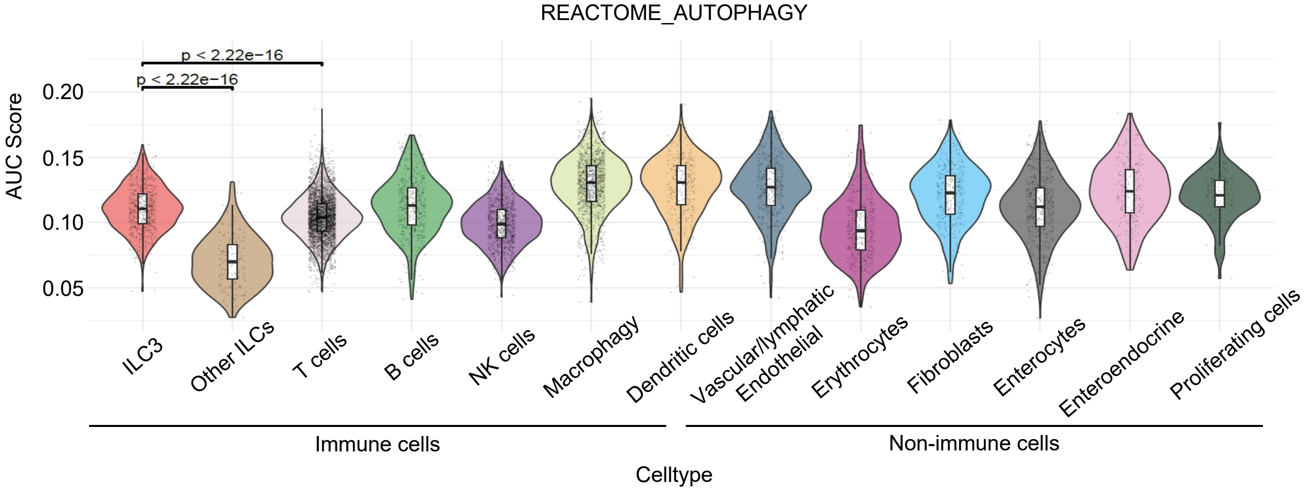

Supplementary Figure 32. Single-cell transcriptomic analysis reveals autophagy activation in district intestinal cells. a Violin plot showing the activity of the REACTOME_AUTOPHAGY pathway (measured by AUC score) across distinct intestinal cell types, including immune and non-immune cells. Statistical significance between ILC3s and other indicated cell populations was assessed by Wilcox test.

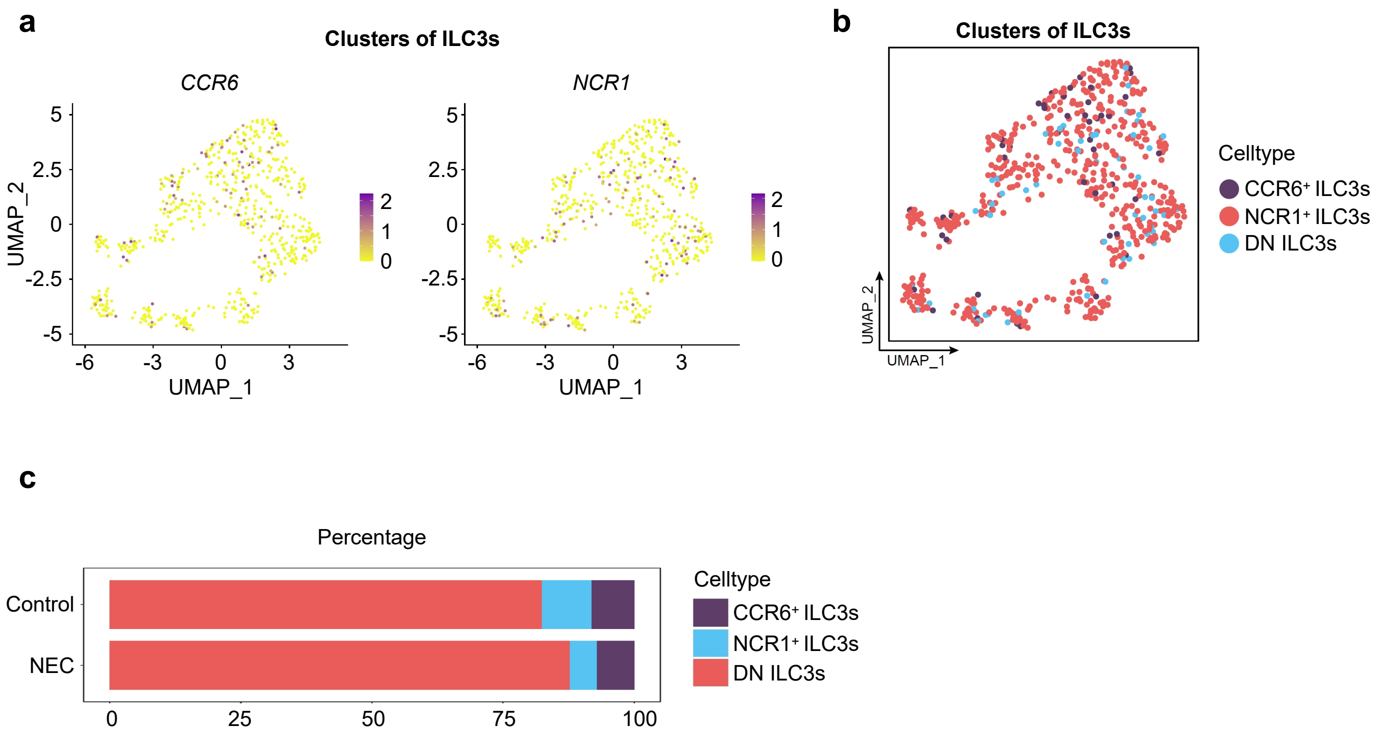

Supplementary Figure 33. Single-cell transcriptomic analysis reveals increased DN ILC3s in patient with NEC. **a** UMAP plots showing the expression levels of *CCR6* and *NCR1* in the ILC3 clusters. **b** UMAP visualization of CCR6^{+} ILC3s (dark purple), NCR1^{+} ILC3s (light blue), and DN ILC3s (red). **c** Stacked bar chart of ILC3 subset composition in Control and NEC infants.

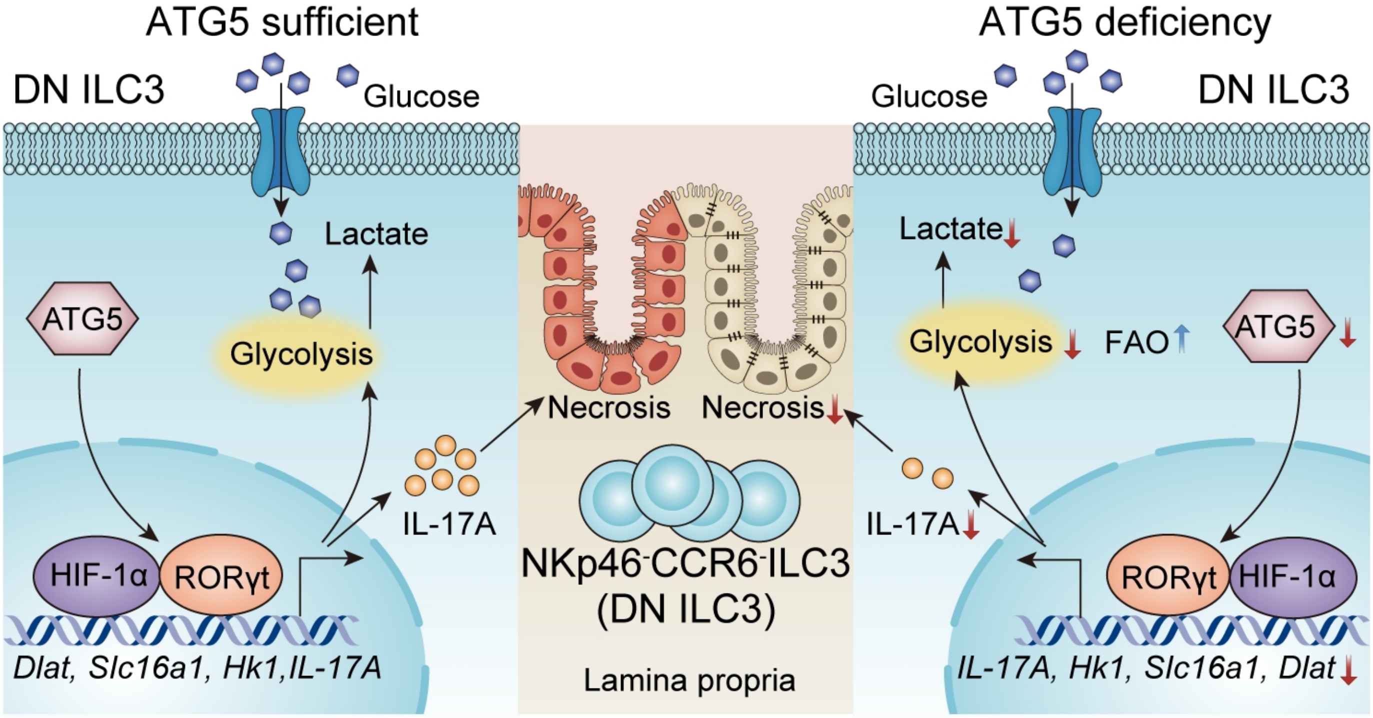

Supplementary Figure 34. Schematic model of the autophagy-energy-DN ILC3 pathogenicity axis during NEC development.

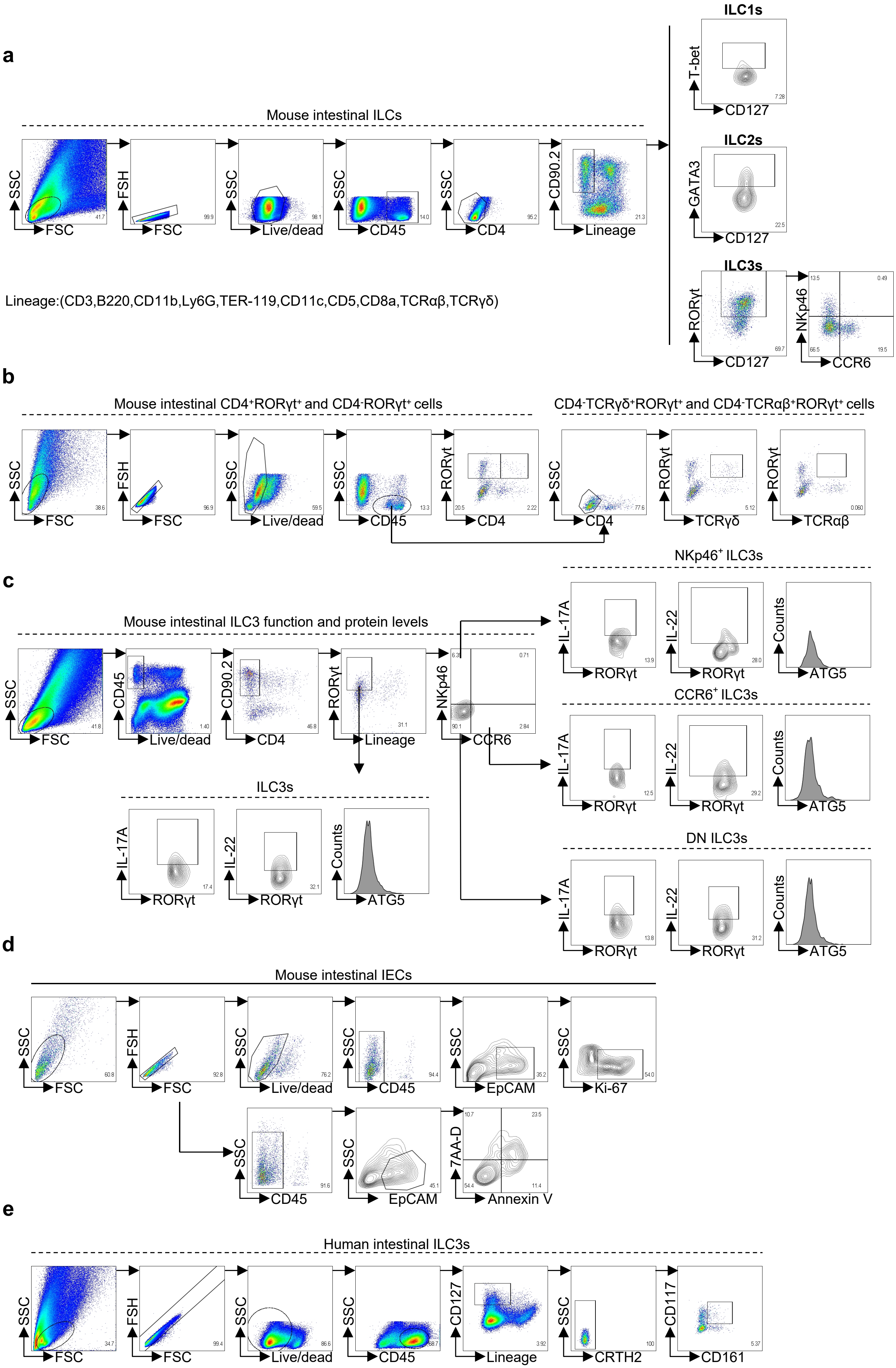

a
Mouse intestinal ILCs
SSC
FSC
FSH
Live/dead
CD45
CD4
CD90.2
Lineage
41.7
99.9
98.1
14.0
95.2
21.3
Lineage:(CD3,B220,CD11b,Ly6G,TER-119,CD11c,CD5,CD8a,TCRαβ,TCRγδ)
ILC1s
T-bet
CD127
7.28
ILC2s
GATA3
22.5
ILC3s
RORγt
69.7
NKp46
CCR6
13.5
0.49
66.5
19.5
b
Mouse intestinal CD4+RORγt+ and CD4-RORγt+ cells
CD4-TCRγδ+RORγt+ and CD4-TCRαβ+RORγt+ cells
38.6
96.9
59.5
13.3
20.5
2.22
77.6
5.12
0.060
TCRγδ
TCRαβ
c
Mouse intestinal ILC3 function and protein levels
41.8
1.40
46.8
31.1
6.35
0.71
90.1
2.84
NKp46+ ILC3s
IL-17A
IL-22
Counts
ATG5
13.9
28.0
CCR6+ ILC3s
12.5
29.2
ILC3s
17.4
32.1
DN ILC3s
13.8
31.2
d
Mouse intestinal IECs
60.8
92.8
76.2
94.4
EpCAM
35.2
Ki-67
54.0
91.6
45.1
7AA-D
Annexin V
10.7
23.5
54.4
11.4
e
Human intestinal ILC3s
34.7
99.4
86.6
68.7
CD127
3.92
CRTH2
100
CD117
CD161
5.37

Supplementary Figure 35. Flow cytometry gating strategies for the identification of intestinal innate lymphoid cell (ILC) subsets, RORγt+ T cells, and epithelial cells in mice and humans. **a** The gating strategy for ILC subsets (applied in Fig. 1c, 1h, 3l, 4d, 4n, 6d, 7d, Suppl. Fig. 3a–d, 5d, 6b, 16e, 18g, 18l–o, 19c, 19e, 19g, 26d, 27b, 28d). **b** Gating strategy for the identification of RORγt^{+} T cells in the mouse intestinal lamina propria (applied in Fig. 1d, Suppl. Fig. 4b,c, 16c, 18e, 19c, 27b, 29b). **c** The gating strategy for functional and protein-level analyses in intestinal ILC3 subsets (applied in Fig. 1k–n, 3f-h, 3m,n, 3p, 4f–i, 5c–f, 5i, 5l, 6e,f, 7e–h, Suppl. Fig. 5e,f, 15a,b, 17a–h, 18h–k, 19i,j, 21f,g, 23a, 24c,d, 27f−i, 29f−i). **d** The gating strategy for IECs (applied in Fig. 2d−g, 2m−p, 7j, Suppl. Fig. 1h−j, 13e,g,h,m,o,p, 29j–l). **e** The gating strategy for human ILC3s (applied in Fig. 8d).

a

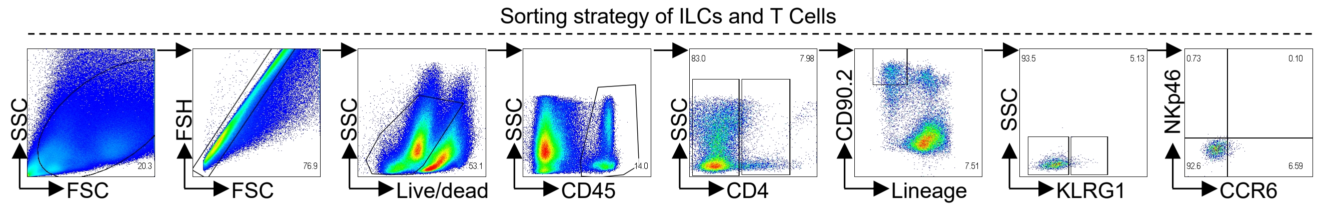

CD4$^+$ T cells: CD45$^+$CD4$^+$

ILC2s: CD45$^+$CD4$^-$CD90.2$^+$Lin$^-$KLRG1$^+$

NKp46$^+$ ILC3s: CD45$^+$CD4$^-$CD90.2^{hi}Lin$^-$KLRG1$^-$CCR6$^-$NKp46$^+$

CCR6$^+$ ILC3s: CD45$^+$CD4$^-$CD90.2^{hi}Lin$^-$KLRG1$^-$CCR6$^+$NKp46$^-$

DN ILC3s: CD45$^+$CD4$^-$CD90.2^{hi}Lin$^-$KLRG1$^-$CCR6$^-$NKp46$^-$

b

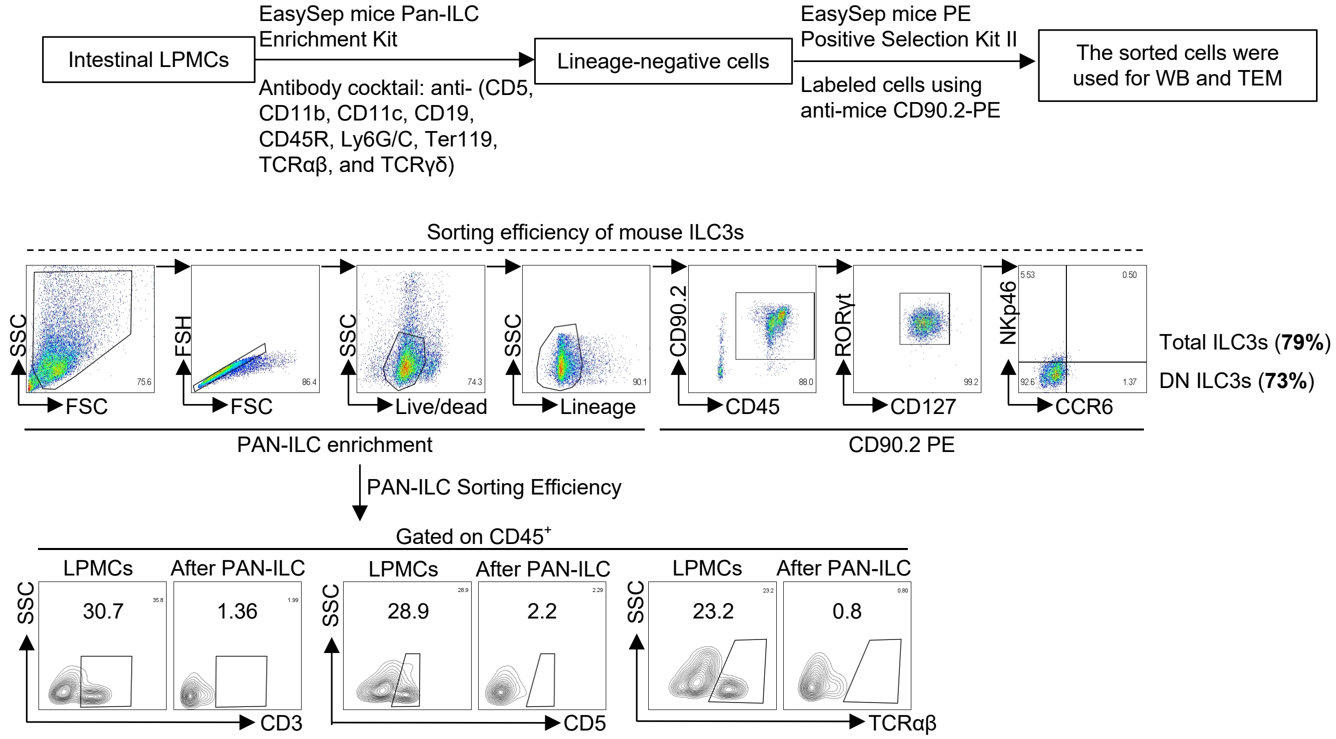

c

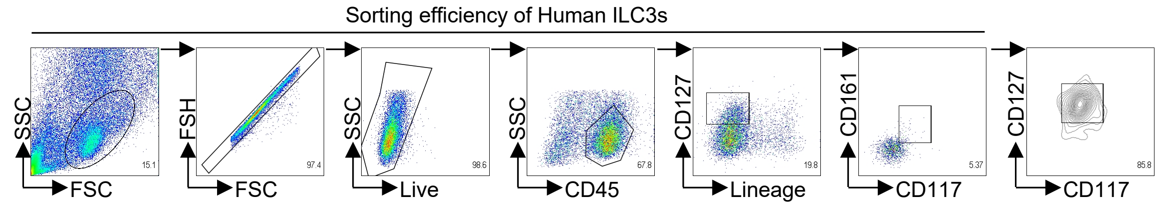

Supplementary Figure 36. Isolation and gating strategies for mouse and human intestinal ILCs. a The flow cytometry-based sorting for ILC subsets and CD4$^+$ T cells (applied in Fig. 2i, 3d,q, 5h,j,m, 6h, 7i,o, Suppl. Fig. 7a–f, 9a–c, 10a–c, 13d, 21e,h–j, 25a, 28c,d). **b** Schematic workflow for intestinal ILC purification from mouse intestinal lamina propria mononuclear cells (LPMCs). ILCs were enriched using the EasySep Mouse Pan-ILC Enrichment Kit (depleting Lineage$^+$ cells) and positively selected with anti-CD90.2-PE (EasySep Mouse PE Positive Selection Kit II). This purification workflow was applied in Figure 3c, 3e and 3o. **c** The gating strategy for sorting human ILC3s (applied in Fig. 3e–g).

	Gestational age at birth (weeks+days)	Weight at birth (g)	Age at surgery (days)	Sex	Diagnosis
Control1	37+1	2200	17	Female	Hirschsprung disease
Control2	37+1	3000	17	Female	Hirschsprung disease
Control3	39	3200	87	Female	Hirschsprung disease
Control4	37	3300	343	Female	Hirschsprung disease
Control5	40	3300	14	Male	Intestinal Atresia
Control6	27	1150	159	Female	Ileostomy Closure
Control7	39	3170	117	Male	Hirschsprung disease
Control8	39	2750	207	Male	Congenital Anorectal Atresia
Control9	39	3580	60	Male	Hirschsprung disease
NEC1	26+4	800	27	Female	IIIB
NEC2	31	850	1	Female	IIIB
NEC3	27	1200	37	Female	IIB
NEC4	30+4	990	18	Male	IIIB
NEC5	37+5	3100	3	Female	IIB
NEC6	38+6	3200	1	Female	IIIB
NEC7	37+1	2740	45	Female	IIIB

Supplementary Table 1. Clinical characteristics of human patient cohorts. Clinical and demographic characteristics of the study subjects, including control patients with non-inflammatory intestinal conditions and patients with NEC. Key parameters include gestational age at birth (weeks+days), birth weight (g), age at surgery (days), sex, and primary diagnosis.

Supplementary Table 2. Reagents used in this study

Name	Cat#	Vendor	Country
Fix & Perm Solution	554722	BD Biosciences	USA
DMSO	V900090	Sigma	USA
RPMI-1640	01-100-1ACS	BI	Israel
FBS	04-001-1ACS	BI	Israel
Penicillin-Streptomycin Solution	03-031-1B	BI	Israel
DMEM	06-1055-57-1A	BI	Israel
DMEM(No Glucose)	BL1124A	Biosharp	China
PEG300	S6704	Selleck Chem	USA
Percoll	17-0891-09	GE	Sweden
DTT	P001008	Amresco	USA
Collagenase I	17104019	Gibco	USA
Hyaluronidase	H3506-1G	SIGMA	USA
Dispase II	04942078001	Roche	Switzerland
DNase I	B002138-0025	Sangon Biotech	China
Bicinchoninic Acid Protein Assay Kit	P0012S	Beyotime	China
TRIzol reagent	15596-026	Invitrogen	USA
StarScript II RT Kit	A214-10	Genstar	China
2 × RealStar Universal SYBR qPCR Mix	A308-10	Genstar	China
3-MA (3-Methyladenine)	S2767	Selleck Chem	USA
Rapamycin	S1039	Selleck Chem	USA
Etomoxir	E4787	Selleck Chem	USA
2-Deoxy-D-glucose (2-DG)	S4701	Selleck Chem	USA
Oligomycin A	S1478	Selleck Chem	USA
Sodium lactate	S817836	Macklin	China
PMA	P-800-1mg	Sigma-Aldrich	USA
Ionomycin	I-700-1mg	Sigma-Aldrich	USA
Brefeldin A	B-275-1mg	Sigma-Aldrich	USA
Phosphatidylcholine(PC)Colorimetric Assay kit	E-BC-K796-M	Elabscience	China
Lactic Acid(LA) Colorimetric Assay Kit	E-BC-K002-M	Elabscience	China
Matrigel	M8371	Solarbio	China
8.0 μm pore size PET track-etched membrane	353097	Falcon	USA
Phosphatidylcholine	8002-43-5	Aladdin	China
Mouse IL-22 Uncoated ELISA Kit with Plates	88-7422-22	Invitrogen	USA
Mouse IL-17A (homodimer) Uncoated ELISA Kit with Plates	88-7371-22	Invitrogen	USA
Mouse GM-CSF ELISA Kit	DG30864M	Dogesce	China
Human IL-22 ELISA Kit	DG10322H	Dogesce	China
Human IL-17A ELISA Kit	DG10431H	Dogesce	China
Foxp3 / Transcription Factor Staining Buffer kit	TNB-0607	TONBO biosciences	USA
C11-BODIPY	D3861	Invitrogen	USA
HCS LipidTOX Deep Red neutral lipid stain	H34477	Invitrogen	USA
DCFH-DA (cytosolic ROS)	C369	Invitrogen	USA
Puromycin dihydrochloride	HY-B1743A	MedChemExpress	USA
EasySep Mouse Pan-ILC Enrichment Kit	19875	StemCell	Canada
EasySep Release Mouse PE Positive Selection Kit	17656	StemCell	Canada
DAPI staining solution	C1006	Beyotime Biotechnology	China
FITC-labeled dextran	-	Sigma-Aldrich	USA

Supplementary Table 3 . Antibodies used in this study

Name (Dilution)	Fluorochrome	Cat#	Vendor	Country
**Flow cytometry**				
Biotin-anti-mouse CD4 (1:100)	-	100508	BioLegend	USA
Biotin-anti-mouse CD8a (1:100)	-	100704	BioLegend	USA
Biotin-anti- mouse TER-119 (1:100)	-	116204	BioLegend	USA
Biotin-anti-mouse CD11c (1:100)	-	117304	BioLegend	USA
Biotin-anti-mouse NK-1.1 (1:100)	-	108704	BioLegend	USA
Biotin-anti-mouse CD5 (1:100)	-	13-0051-85	eBioscience	USA
Biotin-anti-mouse TCR β chain (1:100)	-	13-5961-85	eBioscience	USA
Biotin-anti-mouse TCR γ/δ (1:100)	-	13-5711-85	eBioscience	USA
Biotin-anti-mouse CD3e (1:100)	-	13-0861-86	eBioscience	USA
Biotin-anti-mouse Ly-6G (1:100)	-	13-5931-86	eBioscience	USA
Biotin-anti-mouse CD11b (1:100)	-	11-0112-86	eBioscience	USA
Biotin-anti-human/mouse CD45R/B220 (1:100)	-	103204	BioLegend	USA
Biotin-anti-human CD56 (1:100)	-	13-0567-82	eBioscience	USA
Biotin-anti-human CD11b (1:100)	-	301304	BioLegend	USA
Biotin-anti-human TCRγδ (1:100)	-	331206	BioLegend	USA
Biotin-anti-human TCRαβ (1:100)	-	13-9986-82	eBioscience	USA
Biotin-anti-human CD14 (1:100)	-	367106	BioLegend	USA
Biotin-anti-human CD34 (1:100)	-	343524	BioLegend	USA
Biotin-anti-human CD19 (1:100)	-	302204	BioLegend	USA
Biotin-anti-human CD3 (1:100)	-	300404	BioLegend	USA
Biotin-anti-human CD123 (1:100)	-	13-1239-82	eBioscience	USA
Biotin-anti-human CD8 (1:100)	-	301004	BioLegend	USA
Biotin-anti-human CD11c (1:100)	-	13-0116-82	eBioscience	USA
Biotin-anti-human FceR1 alpha Monoclonal Antibody (1:100)	-	13-5899-82	eBioscience	USA
STREPTAVIDIN (1:100)	FITC	11-4317-87	eBioscience	USA
STREPTAVIDIN (1:100)	APC-eFluor 780	47-4317-82	eBioscience	USA
anti- mouse-CD90.2 (1:100)	PE-Cyanine7	25-0902-82	eBioscience	USA
anti-mouse CD127 (1:100)	PB	48-1271-82	eBioscience	USA
anti-mouse RORγt (1:100)	PerCP-eFluor710	46-6981-80	eBioscience	USA
anti -mouse CD196 (1:100)	APC	129814	BioLegend	USA
anti-mouse CD335 (1:100)	PE	12-3351-82	eBioscience	USA
anti-mouse CD335 (1:100)	FITC	11-3351-82	eBioscience	USA
anti-mouse CD4 (1:100)	ef506	69-0042-82	eBioscience	USA
anti-mouse CD4 (1:100)	ef450	48-0042-82	eBioscience	USA
anti-mouse CD45 (1:100)	APC-ef780	47-0451-82	eBioscience	USA
anti-mouse CD45 (1:50)	ef506	69-0451-82	eBioscience	USA
anti-mouse CD45 (1:100)	PE-eFluor 610	61-0451-82	eBioscience	USA
anti-mouse/human Gata-3 (1:100)	PE	12-9966-42	eBioscience	USA
anti-mouse/human T-bet (1:100)	eFluor660	50-5825-82	eBioscience	USA
anti-mouse IL-22 (1:50)	PE	12-7221-82	eBioscience	USA
anti-mouse-KLRG1 (1:100)	PerCP-eFluor 710	46-5893-80	eBioscience	USA
anti-mouse/rat IL-17A (1:50)	PE	12-7177-81	eBioscience	USA
anti-mouse CD326 (1:100)	AF488	118210	BioLegend	USA
APC Annexin V (1:100)	APC	640920	BioLegend	USA
anti-mouse/rat-Ki-67 (1:100)	APC	17-5698-82	eBioscience	USA
anti-human CD45 (1:100)	PerCP5.5	45-0459-42	eBioscience	USA
anti-human CD45 (1:100)	APC-H7	560274	BD Bioscience	USA
anti-human CD127 (1:100)	FITC	11-1278-42	eBioscience	USA
anti-human CD3 (1:100)	BV421	563797	BD Bioscience	USA

Supplementary Table 3 . Antibodies used in this study

Name (Dilution)	Fluorochrome	Cat#	Vendor	Country
**Flow cytometry**				
anti-human CD117 (1:100)	EF450	48-1178-42	eBioscience	USA
anti-human CD161 (1:100)	PE-Cyanine7	25-1619-42	eBioscience	USA
anti-human CD161 (1:100)	BV605	748284	BD Bioscience	USA
anti-human CD294 (1:100)	PE	12-2949-42	eBioscience	USA
ATG5 Antibody (1:50)	PE	sc-133158 PE	Santa	USA
anti-puromycin (1:100)	AF488	MABE343-AF488	Sigma-Aldrich	USA
c-Myc Rabbit mAb (1:50)	-	R380784	Zenbio	China
SQSTM1/p62 Rabbit pAb (1:50)	-	380612	Zenbio	China
GLUT1 Rabbit mAb (1:50)	-	R380464	Zenbio	China
HIF1 alpha Rabbit pAb (1:50)	-	340462	Zenbio	China
GLUT3 Polyclonal antibody (1:50)	-	20403-1-AP	proteintech	USA
LAMP-1 Polyclonal antibody (1:50)	-	33243-1-AP	proteintech	USA
MLXIP Polyclonal antibody (1:50)	-	13614-1-AP	proteintech	USA
Donkey anti-rabbit IgG(min,x-reactivity) (1:100)	PE	406421	BioLegend	USA
**Western blot**				
APG5L Rabbit mAb (1:500)	-	R381320	Zenbio	China
ULK1 Rabbit mAb (1:500)	-	R381887	Zenbio	China
LC3B Rabbit mAb (1:500)	-	R381544	Zenbio	China
GAPDH Rabbit mAb (1:500)	-	R380626	Zenbio	China
Goat Anti-Rabbit IgG H&L(HRP) (1:200)	-	511203	Zenbio	China
Goat Anti-Mouse IgG H&L(HRP) (1:200)	-	511103	Zenbio	China
**Cytokine neutralization antibody**				
Anti-IL-17A	-	BE0173	Bio X Cell	USA
Mouse IgG1 isotype control antibody	-	BE0083	Bio X Cell	USA
Anti-IL-22	-	16-7222-85	eBioscience	USA
Rat IgG2a isotype control antibody	-	16-4321-85	eBioscience	USA
Anti-GM-CSF	-	BE0259	Bio X Cell	USA
Rat IgG2a isotype control antibody	-	BE0089	Bio X Cell	USA
**Immunofluorescence histology**				
APG5L Rabbit mAb (1:100)	-	R381320	Zenbio	China
Anti-human-CD3-BV421 (1:50)	BV421	563797	BD Bioscience	USA
Anti-RORγt (1:100)	-	sc-293150	Santa	USA
Goat Anti-Rabbit IgG H&L (1:100)	AF488	ab150077	Abcam	UK
Goat Anti-Mouse IgG H&L (1:100)	AF647	ab150115	Abcam	UK
**Chip-PCR**				
HIF-1alpha (D1S7W) XP Rabbit mAb (1:100)	-	36169T	CST	USA
Normal Rabbit IgG (1:100)	-	2729S	CST	USA

Supplementary Table 4. Sequences of primers used in this study

Primers name	Sequence	Primers name	Sequence
Ms-β-actin-for	5’-CGTGCGTGACATCAAAGAGAAG-3’	Ms-β-actin-rev	5’-CGTTGCCAATAGTGATGACCTG-3’
Ms-Atg5-for	5’-TGTGCTTCGAGATGTGTGGTT-3’	Ms-Atg5-rev	5’-GTCAAATAGCTGACTCTTGGCAA-3’
Ms-Ulk1-for	5’-ACATCCGAGTCAAGATTGCTG-3’	Ms-Ulk1-rev	5’-GCTGGGACATAATGACCTCAGG-3’
Ms-Pcyt1a-for	5’-GATGCACAGAGTTCAGCTAAAGT-3’	Ms-Pcyt1a-rev	5’-TGGCTGCCGTAAACCAACTG-3’
Ms-Cds1-for	5’-GGTGACCACGAAACCGAGAG-3’	Ms-Cds1-rev	5’-CCGCGAATCCACCAGTTCT-3’
Ms-Gpat3-for	5’-GTGCTGGGTGTCCTAGTGC-3’	Ms-Gpat3-rev	5’-AAGCTGATCCCAATGAAAGC-3’
Ms-Lpcat4-for	5’-AAACCCGTTCGTGCATGAGTT-3’	Ms-Lpcat4-rev	5’-CGTTGTGGCATACAGTCTTCCT-3’
Ms-Hk1-for	5’-CGGAATGGGGAGCCTTTGG-3’	Ms-Hk1-rev	5’-GCCTTCCTTATCCGTTTCAATGG-3’
Ms-Ldhb-for	5’-CATTGCGTCCGTTGCAGATG-3’	Ms-Ldhb-rev	5’-GGAGGAACAAGCTCCCGTG-3’
Ms-Aldoa-for	5’-CGTGTGAATCCCTGCATTGG-3’	Ms-Aldoa-rev	5’-CAGCCCCTGGGTAGTTGTC-3’
Ms-Dlat-for	5’-TCCCTCCGCATCAGAAGGTT-3’	Ms-Dlat-rev	5’-CCAACTGGAACATCTCTGGTC-3’
Ms-Lpin3-for	5’-TCGGGCTTTCCCTCAGACT-3’	Ms-Lpin3-rev	5’-CCTGTAATGACTAGGCCCTCAG-3’
Ms-Gpd2-for	5’-GAAGGGGACTATTCTTGTGGGT-3’	Ms-Gpd2-rev	5’-GGATGTCAAATTCGGGTGTGT-3’
Ms-Mgat1-for	5’-TTGTGCTTTGGGGTGCTATCA-3’	Ms-Mgat1-rev	5’-CCACAGTGGGAACTCTCCA-3’
Ms-Pnliprp2-for	5’-ATGCCTATGGATGTCCGTGGA-3’	Ms-Pnliprp2-rev	5’-TGCCCAGGGCTTGTCATTG-3’
Ms-Acox1-for	5’-TAACTTCCTCACTCGAAGCCA-3’	Ms-Acox1-rev	5’-AGTTCCATGACCCATCTCTGTC-3’
Ms-Slc16a1-for	5’-TGTTAGTCGGAGCCTTCATTTC-3’	Ms-Slc16a1-rev	5’-CACTGGTCGTTGCACTGAATA-3’
Ms-ZO-1-for	5'-AACCCGAAACTGATGCTGTG-3'	Ms-ZO-1-rev	5'-CCCTTGGAATGTATGTGGAGAG-3'
Ms-Claudin-1-for	5'-GGAGTCAGTGTTTCAGCCTATGGT-3'	Ms-Claudin-1-rev	5'-GAAGGGTTCATGCCTCTCATCT-3'
Ms-Occludin-for	5'-CCCAGATTAGAGTCCAAAGTCAGT-3'	Ms-Occludin-rev	5'-CGGAAACCTTAGAGAGATGCC-3'
Ms-Hk2-for	5′-TGATCGCCTGCTTATTCACGG-3′	Ms-Hk2-rev	5′-AACCGCCTAGAAATCTCCAGA-3′
Ms-Glut1-for	5'-CTCTGTCGGCCTCTTTGTTAAT-3'	Ms-Glut1-rev	5'-CCAGTTTGGAGAAGCCCATAAG-3'
Ms-Glut3-for	5'-CTTTGGCAGACGCAACTCTAT-3'	Ms-Glut3-rev	5'-ACCAGAATCCCAACAACGATG-3'
Ms-Pdk1-for	5’-GGACTTCGGGTCAGTGAATGC-3’	Ms-Pdk1-rev	5’-TCCTGAGAAGATTGTCGGGGA-3’
Ms-Slc25a14-for	5’-CCACTTTAAGCCATGAGATGTCT-3’	Ms-Slc25a14-rev	5’-CAAGGCCGCCATACACAAA-3’
Ms-Sdhc-for	5’-GCTGCGTTCTTGCTGAGACA-3’	Ms-Sdhc-rev	5’-ATCTCCTCCTTAGCTGTGGTT-3’
Ms-Mdh1-for	5’-TTCTGGACGGTGTCCTGATG-3’	Ms-Mdh1-rev	5’-TTTCACATTGGCTTTCAGTAGGT-3’
Ms-Ndufv1-for	5’-TTTCTCGGCGGGTTGGTTC-3’	Ms-Ndufv1-rev	5’-GGTTGGTAAAGATCCGGTCTTC-3’
Ms-Ndufv2-for	5’-GCAAGGAATTTGCATAAGACAGC-3’	Ms-Ndufv2-rev	5’-TAGCCATCCATTCTGCCTTTG-3’
Ms-Atp5b-for	5’-GGTTCATCCTGCCAGAGACTA-3’	Ms-Atp5b-rev	5’-AATCCCTCATCGAACTGGACG-3’
Ms-Ldha-for	5’-TGTCTCCAGCAAAGACTACTGT-3’	Ms-Ldha-rev	5’-GACTGTACTTGACAATGTTGGA-3’
Ms-Pkm-for	5’-GTACCCCCATCCCAGTTCCA-3’	Ms-Pkm-rev	5’-TCTGAGGTCTCACACAGGAAA-3’
Ms-Map1lc3b-for	5’-TTATAGAGCGATACAAGGGGGAG-3’	Ms-Map1lc3b-rev	5’-CGCCGTCTGATTATCTTGATGAG3’
Hu-ATG5-for	5’-AAAGATGTGCTTCGAGATGTGT-3’	Hu-ATG5-rev	5’-CACTTTGTCAGTTACCAACGTCA-3’
Hu-ATG7-for	5’-ATGATCCCTGTAACTTAGCCCA-3’	Hu-ATG7-rev	5’-CACGGAAGCAAACAACTTCAAC-3’
Hu-ATG12-for	5’-TAGAGCGAACACGAACCATCC-3’	Hu-ATG12-rev	5’-CACTGCCAAAACACTCATAGAGA-3’
Hu-IL-17A-for	5’-CGATCCACCTCACCTTGGA-3’	Hu-IL-17A-rev	5’-TCCCAGATCACAGAGGGATATCTCTC-3’
Hu-IL-22-for	5’-TGCTGTTCCCTCAATCTG-3’	Hu-IL-22-rev	5’-TGTGCTTAGCCTGTTGCTG-3’
Ms-Dlat-ChIP-site1-for	GTGCGTGGTAGAAATAAAGAGC	Ms-Dlat-ChIP-site1-rev	CAGTTCCCAGCACCCACA
Ms-Dlat-ChIP-site1-for	AGCAGAAGAGCCTGGGAGA	Ms-Dlat-ChIP-site1-rev	CAGTTCCCAGCACCCACA
Ms-Dlat-ChIP-site1-for	GAAATAAAGAGCCGAAGC	Ms-Dlat-ChIP-site1-rev	GTCAGGTAATACAAGGTCAT
Ms-Slc16a1-ChIP-site1-for	TTCCCCTTTTCTCCTCCAG	Ms-Slc16a1-ChIP-site1-rev	ATAAGGATGTGCGGGGTCT
Ms-Slc16a1-ChIP-site1-for	TTTTCCCCTTTTCTCCTCCA	Ms-Slc16a1-ChIP-site1-rev	ATAAGGATGTGCGGGGTCT
Ms-Slc16a1-ChIP-site1-for	CGCCAAAAGAAGCTACCCC	Ms-Slc16a1-ChIP-site1-rev	TGATAAGGATGTGCGGGG
Ms-Hk1-ChIP-site1-for	5’-GCCAGAGCAGGAGAATGACG-3	Ms-Hk1-ChIP-site1-rev	5’-GGGAACGTGCAGGGTGAT-3
Ms-Hk1-ChIP-site2-for	5’-CAGCCAGAGCAGGAGAATGA-3	Ms-Hk1-ChIP-site2-rev	5’-GGGAACGTGCAGGGTGAT-3’
Ms-Hk1-ChIP-site3-for	5’-ATCACCCTGCACGTTCCC-3’	Ms-Hk1-ChIP-site3-rev	5’-CCTCCAGATCGTGTCCCAC-3’
Clostrdium-for	5’-AAAGGAAGATTAATACCGCATAA-3’	Clostrdium-rev	5’-ATCTTGCGACCGTACTCCCC-3’
