# Supplementary material for: Blockade of NKp46⁻ CCR6⁻ ILC3 autophagy protects against necrotizing enterocolitis by restoring energy metabolism balance in mice
Source: Nat Commun. 2026 May 19;17:6579. doi: 10.1038/s41467-026-73356-x (PMC13381918; doi:10.1038/s41467-026-73356-x)
Supplement: Supplementary file 2 — Reporting Summary [file 41467_2026_73356_MOESM2_ESM.pdf]

nature portfolio

Corresponding author(s): Yumei He

Last updated by author(s): Apr 27, 2026

Reporting Summary

Nature Portfolio wishes to improve the reproducibility of the work that we publish. This form provides structure for consistency and transparency in reporting. For further information on Nature Portfolio policies, see our Editorial Policies and the Editorial Policy Checklist.

Statistics

For all statistical analyses, confirm that the following items are present in the figure legend, table legend, main text, or Methods section.

n/a	Confirmed	
☐	☒	The exact sample size (*n*) for each experimental group/condition, given as a discrete number and unit of measurement
☐	☒	A statement on whether measurements were taken from distinct samples or whether the same sample was measured repeatedly
☐	☒	The statistical test(s) used AND whether they are one- or two-sided<br>*Only common tests should be described solely by name; describe more complex techniques in the Methods section.*
☒	☐	A description of all covariates tested
☐	☒	A description of any assumptions or corrections, such as tests of normality and adjustment for multiple comparisons
☐	☒	A full description of the statistical parameters including central tendency (e.g. means) or other basic estimates (e.g. regression coefficient) AND variation (e.g. standard deviation) or associated estimates of uncertainty (e.g. confidence intervals)
☐	☒	For null hypothesis testing, the test statistic (e.g. *F*, *t*, *r*) with confidence intervals, effect sizes, degrees of freedom and *P* value noted<br>*Give P values as exact values whenever suitable.*
☒	☐	For Bayesian analysis, information on the choice of priors and Markov chain Monte Carlo settings
☒	☐	For hierarchical and complex designs, identification of the appropriate level for tests and full reporting of outcomes
☒	☐	Estimates of effect sizes (e.g. Cohen's *d*, Pearson's *r*), indicating how they were calculated

Our web collection on statistics for biologists contains articles on many of the points above.

Software and code

Policy information about availability of computer code

Data collection	No custom code or software was used for data collection.
Data analysis	All software used for data analysis are commercially available.FlowJo V10 (Treestar) - flow cytometry data analysis; Microsoft Excel (version 3.11.0.36530); Graphpad Prism 8 - graph plotting and statistics; NIS-Elements Viewer 4.50 - viewing of histology and immunostained slides;

For manuscripts utilizing custom algorithms or software that are central to the research but not yet described in published literature, software must be made available to editors and reviewers. We strongly encourage code deposition in a community repository (e.g. GitHub). See the Nature Portfolio guidelines for submitting code & software for further information.

Data

Policy information about availability of data

All manuscripts must include a data availability statement. This statement should provide the following information, where applicable:

- Accession codes, unique identifiers, or web links for publicly available datasets
- A description of any restrictions on data availability
- For clinical datasets or third party data, please ensure that the statement adheres to our policy

The single-cell RNA-seq (scRNA-seq) data generated in this study have been deposited in the NCBI Gene Expression Omnibus (GEO) database under accession code GSE306522 [https://www.ncbi.nlm.nih.gov/geo/query/acc.cgi?acc=GSE306522]. The ATAC-seq data generated in this study have been deposited in the NCBI Gene Expression Omnibus (GEO) database under accession code GSE319269 [https://www.ncbi.nlm.nih.gov/geo/query/acc.cgi?acc=GSE319269]. The bulk RNA-seq data

generated in this study have been deposited in the NCBI Gene Expression Omnibus (GEO) database under accession code GSE306398 [https://www.ncbi.nlm.nih.gov/geo/query/acc.cgi?acc=GSE306398]. The 16S rRNA gene sequencing data generated in this study have been deposited in the NCBI Sequence Read Archive (SRA) database under BioProject accession code PRJNA1432033 [https://www.ncbi.nlm.nih.gov/sra/PRJNA1432033]. The metabolomic data generated in this study have been deposited in the MetaboLights database under accession code MTBLS12891 [https://www.ebi.ac.uk/metabolights/editor/MTBLS12891/overview]. The raw flow cytometry data for the core figures in this study have been deposited in Figshare with the following DOIs: 32025558 [https://doi.org/10.6084/m9.figshare.32025558, 32020050 [https://doi.org/10.6084/m9.figshare.32020050], 32020017 [https://doi.org/10.6084/m9.figshare.32020017], 32019897 [https://doi.org/10.6084/m9.figshare.32019897], 32013036 [https://doi.org/10.6084/m9.figshare.32013036], 32011416 [https://doi.org/10.6084/m9.figshare.32011416], 32010489 [https://doi.org/10.6084/m9.figshare.32010489], 32009283 [https://doi.org/10.6084/m9.figshare.32009283], 32008974 [https://doi.org/10.6084/m9.figshare.32008974], 32008173 [https://doi.org/10.6084/m9.figshare.32008173], 32008092 [https://doi.org/10.6084/m9.figshare.32008092], 32006430 [https://doi.org/10.6084/m9.figshare.32006430]. All other raw flow cytometry data are available from the corresponding author upon reasonable request. All data are included in the Supplementary Information or available from the authors. The raw numbers for charts and graphs are available in the Source Data file whenever possible.

Research involving human participants, their data, or biological material

Policy information about studies with human participants or human data. See also policy information about sex, gender (identity/presentation), and sexual orientation and race, ethnicity and racism.

Reporting on sex and gender	Both male and female infants were included in our study. NEC: 7 cases (1 male and 6 female). Controls: 9 cases (4 male and 5 female). The sex and gender of the infants did not influence the recruitment. Due to the small sample size, sex was included in analyses as a possible covariate but did not reveal any statistically significant or noteworthy differences between groups.
Reporting on race, ethnicity, or other socially relevant groupings	There were no relevant groupings in this study.
Population characteristics	In the NEC group, patients were clinically diagnosed with necrotising enterocolitis. The control group consisted of patients with non-NEC intestinal conditions undergoing ostomy reanastomosis procedures, without clinical or histological evidence of intestinal disease. Their primary diagnoses included meconium plug/intestinal obstruction, spontaneous intestinal perforation, Hirschsprung's disease (utilising unaffected tissue), duodenal atresia, and intestinal pseudo-obstruction.
Recruitment	Fresh small intestinal tissues were obtained from surgical resections in neonatal patients at the Department of Pediatric Surgery, Zhujiang Hospital, Southern Medical University (Guangzhou, China). Human intestinal samples were collected from neonates undergoing resection for NEC (at the time of stoma closure) and from non-NEC surgical controls. Control tissues were obtained during stoma closure surgery in the absence of active intestinal disease or history of NEC at the of sampling, whose primary diagnoses including meconium plug/ileus, spontaneous intestinal perforation, Hirschsprung disease (unaffected tissue used), duodenal atresia, and intestinal pseudo-obstruction. All subjects underwent screening for hepatitis B surface antigen, hepatitis C virus, classical swine fever virus, and human immunodeficiency virus antibodies. Samples were collected in de-identified form under informed consent exemption, thereby eliminating self-selection bias.
Ethics oversight	This research was approved by the Southern Medical University Zhujiang Hospital Medical Ethics Committee (Approval ID: 2023-KY-035-01). Written informed consent was obtained from the legal guardians of all participants, including explicit consent for the publication of de-identified clinical information that may potentially contain indirect identifiers of individual participants.

Note that full information on the approval of the study protocol must also be provided in the manuscript.

Field-specific reporting

Please select the one below that is the best fit for your research. If you are not sure, read the appropriate sections before making your selection.

☒ Life sciences ☐ Behavioural & social sciences ☐ Ecological, evolutionary & environmental sciences

For a reference copy of the document with all sections, see nature.com/documents/nr-reporting-summary-flat.pdf

Life sciences study design

All studies must disclose on these points even when the disclosure is negative.

Sample size	No statistical method was used to predetermine sample size. We employed the standard experimental protocol recognised within the field, namely three independent biological replicates. Each replicate group comprised four to six mice, with mice in the same group were from different litters.
Data exclusions	No sample were excluded from analysis.
Replication	All attempts at replication were successful. All experiments were independently performed at least three times to ensure reproducibility.
Randomization	Mouse data: Animals were randomized into experimental groups. Genotype-specific mice were grouped according to genotype, with all experiments utilising sex-matched littermates.<br>Human data: All experiments were randomly assigned into experimental groups.
Blinding	Animal studies were not blinded. Mice were identified by ear tags and genotyped within 1 week of birth. Group allocation was based on

Blinding	genotype, so no randomization was applicable. Histological analysis was performed by two independent investigators, who were blinded to group allocation of mice and patients.

Reporting for specific materials, systems and methods

We require information from authors about some types of materials, experimental systems and methods used in many studies. Here, indicate whether each material, system or method listed is relevant to your study. If you are not sure if a list item applies to your research, read the appropriate section before selecting a response.

Materials & experimental systems

n/a	Involved in the study
☐	☒ Antibodies
☒	☐ Eukaryotic cell lines
☒	☐ Palaeontology and archaeology
☐	☒ Animals and other organisms
☒	☐ Clinical data
☒	☐ Dual use research of concern
☒	☐ Plants

Methods

n/a	Involved in the study
☒	☐ ChIP-seq
☐	☒ Flow cytometry
☒	☐ MRI-based neuroimaging

Antibodies

Antibodies used

Flow cytometry (mouse panel):
Biotin-anti-mouse CD4; Company: BioLegend; Clone: RM4-5; Cat#: 100508; 1:100
Biotin-anti-mouse CD8a; Company: BioLegend; Clone: 53-6.7; Cat#: 100704; 1:100
Biotin-anti- mouse TER-119; Company: BioLegend; Clone: TER-119; Cat#:116204; 1:100
Biotin-anti-mouse CD11c; Company: BioLegend; Clone: N418; Cat#: 117304; 1:100
Biotin-anti-mouse NK-1.1; Company: BioLegend; Clone: PK136; Cat#: 108704; 1:100
Biotin-anti-human/mouse CD45R/B220; Company: BioLegend; Clone: RA3-6B2; Cat#: 103204; 1:100
Biotin-anti-mouse TCR β chain; Company: eBioscience; Clone: H57-597; Cat#: 13-5961-85; 1:100
Biotin-anti-mouse TCR γ/δ; Company: eBioscience; Clone: GL3; Cat#: 13-5711-85; 1:100
Biotin-anti-mouse CD3e; Company: eBioscience; Clone: 145-2C11; Cat#: 13-0031-86; 1:100
Biotin-anti-mouse Ly-6G; Company: eBioscience; Clone: RB6-8C5; Cat#: 13-5931-86; 1:100
Biotin-anti-mouse CD11b; Company: eBioscience; Clone: M1/70; Cat#: 13-0112-86; 1:100
STREPTAVIDIN-FITC; Company: eBioscience; Clone: N/A; Cat#: 11-4317-87; 1:100
STREPTAVIDIN-APC-eFluor 780; Company: eBioscience; Clone: N/A; Cat#: 47-4317-82; 1:100
Anti-mouse-CD90.2(Thy1.2)-PE-Cyanine7; Company: eBioscience; Clone: 53-2.1; Cat#: 25-0902-82; 1:100
Anti-mouse-CD90.2(Thy1.2)-PE; Company: eBioscience; Clone: 53-2.1; Cat#: 12-0902-82; 1:100
Anti-mouse-CD127-eFluor 450; Company: eBioscience; Clone: A7R34; Cat#: 48-1271-82; 1:100
Anti-mouse-RORγt-PerCP-eFluor710; Company: eBioscience; Clone: B2D; Cat#: 46-6981-80; 1:100
Anti-mouse-CD196(CCR6)-APC; Company: BioLegend; Clone: 29-2L17; Cat#: 129814; 1:100
Anti-mouse-CD335(NKp46)-PE; Company: eBioscience; Clone: 29A1.4; Cat#: 12-3351-82; 1:100
Anti-mouse-CD335(NKp46)-FITC; Company: eBioscience; Clone: 29A1.4; Cat#: 11-3351-82; 1:100
Anti-mouse-CD4-eFluor 506; Company: eBioscience; Clone: RM4-5; Cat#: 69-0042-82; 1:100
Anti-mouse-CD4-eFluor 450; Company: eBioscience; Clone: RM4-5; Cat#: 48-0042-82; 1:100
Anti-mouse-CD45-APC-eFluor 780; Company: eBioscience; Clone: 30-F11; Cat#: 47-0451-82; 1:100
Anti-mouse-CD45-eFluor 506; Company: eBioscience; Clone: 30-F11; Cat#: 69-0451-82; 1:50
Anti-mouse-CD45-PE-eFluor 610; Company: eBioscience; Clone: 30-F11; Cat#: 61-0451-82; 1:100
Anti-human/mouse-Gata-3-PE; Company: eBioscience; Clone: TWAJ; Cat#: 12-9966-42; 1:100
Anti-human/mouse-T-bet-eFluor660; Company: eBioscience; Clone: eBio4B10 (4B10); Cat#: 50-5825-82; 1:100
Anti-mouse-IL-22-PE; Company: eBioscience; Clone: 1H8PWSR; Cat#: 12-7221-82; 1:50
Anti-mouse/rat -IL-17A-PE; Company: eBioscience; Clone: eBio17B7; Cat#: 12-7177-81; 1:50
Anti-mouse-ATG5-PE; Company: Santa; Clone: C-1; Cat#: sc-133158 PE; 1:50
Biotin-anti-mouse CD5;Company: eBioscience; Clone: 53-7.3; Cat#: 13-0051-85; 1:100
Anti-mouse-CD326 (Ep-CAM)-AF488;Company:BioLegend; Clone:G8.8; Cat#: 118210; 1:100
Anti-mouse/rat-Ki-67-APC;Company:eBioscience; Clone:SolA15; Cat#: 17-5698-82; 1:100
APC Annexin V;Company:BioLegend;Clone: N/A; Cat#: 640920; 1:100
7-AAD Viability Staining Solution;Company:BioLegend; Clone: N/A;Cat#: 420404; 1:100
Anti-mouse-KLRG1-PerCP-eFluor 710; Company: eBioscience; Clone: (2F1); Cat#: 46-5893-80; 1:100
c-Myc Rabbit mAb; Company: Zenbio; Clone: R03-1I8; Cat#: R380784; 1:50
SQSTM1/p62 Rabbit pAb; Company: Zenbio;Clone: N/A; Cat#: 380612; 1:50
GLUT1 Rabbit mAb; Company: Zenbio; Clone: R09-7B8; Cat#: R380464; 1:50
HIF1 alpha Rabbit pAb; Company: Zenbio; Cat#: 340462; 1:50
GLUT3 Polyclonal antibody; Company: proteintech; Clone: N/A; Cat#: 20403-1-AP; 1:50
LAMP-1 Polyclonal antibody; Company: proteintech; Clone: N/A; Cat#: 33243-1-AP; 1:50
MLXIP Polyclonal antibody; Company: proteintech; Clone: N/A; Cat#: 13614-1-AP; 1:50
Donkey anti-rabbit IgG(min,x-reactivity)-PE; Company: BioLegend; Cat#: 406421; 1:100
Anti-Puromycin-FITC; Company: Sigma-Aldrich; Clone: 12D10; Cat#: MABE343-AF488; 1:100

Flow cytometry (human panel):

Biotin-anti-human/mouse CD45R/B220; Company: BioLegend; Clone: RA3-6B2; Cat#: 103204; 1:100
Biotin-anti-humanCD56 (NCAM); Company: eBioscience; Clone: CMSSB; Cat#: 13-0567-82; 1:100
Biotin-anti-human CD11b; Company: BioLegend; Clone: ICRF44; Cat#: 301304; 1:100
Biotin-anti-human TCRγδ; Company: BioLegend; Clone: B1; Cat#: 331206; 1:100
Biotin-anti-human ab TCR; Company: eBioscience; Clone: IP26; Cat#: 13-9986-82; 1:100
Biotin-anti-human CD14; Company: BioLegend; Clone: 63D3; Cat#: 367106; 1:100
Biotin-anti-human CD34; Company: BioLegend; Clone: 581; Cat#: 343524; 1:100
Biotin-anti-human CD19; Company: BioLegend; Clone: HIB19; Cat#: 302204; 1:100
Biotin-anti-human CD3; Company: BioLegend; Clone: UCHT1; Cat#: 300404; 1:100
Biotin-anti-human CD123; Company: eBioscience; Clone: 6H6; Cat#: 13-1239-82; 1:100
Biotin-anti-human CD8; Company: BioLegend; Clone: RPA-T8; Cat#: 301004; 1:100
Biotin-anti-human CD11c; Company: eBioscience; Clone: 3.9; Cat#: 13-0116-82; 1:100
Biotin-anti-human FceR1 alpha Company: eBioscience; Clone: CRA1; Cat#: 13-5899-82; 1:100
STREPTAVIDIN-FITC; Company: eBioscience; Clone: N/A; Cat#: 11-4317-87; 1:100
STREPTAVIDIN-APC-eFluor 780; Company: eBioscience; Clone: N/A; Cat#: 47-4317-82; 1:100
Anti-human-CD45-PerCP5.5; Company: eBioscience; Clone: HI30; Cat#: 45-0459-42; 1:100
Anti-human-CD127-FITC; Company: eBioscience; Clone: eBioRDR5; Cat#: 11-1278-42; 1:100
Anti-human-CD3-BV421; Company: BD Bioscience; Clone: SK7; Cat#: 563797; 1:100
Anti-human-CD117(c-Kit)-EF450; Company: eBioscience; Clone: 104D2; Cat#: 48-1178-42; 1:100
Anti-human-CD161-PE-Cyanine7; Company: eBioscience; Clone: HP-3G10; Cat#: 25-1619-42; 1:100
Anti-human-CD161-BV605; Company: BD Bioscience; Clone: HP-3G10; Cat#: 748284; 1:100
Anti-human-CD294 (CRTH2)-PE; Company: eBioscience; Clone: BM16; Cat#: 12-2949-42; 1:100
Anti-human CD45-APC-H7;Company: BD Bioscience; Clone:2D1; Cat#: 560274; 1:100

Cytokine neutralization antibody:
Anti-IL-17A; Bio X Cell; Clone 17F3; Cat#: BE0173
Mouse IgG1 isotype control antibody; Bio X Cell; Clone MOPC-21; Cat#: BE0083
Anti-IL-22; eBioscience; Clone IL22JOP; Cat#: 16-7222-85;
Rat IgG2a isotype control antibody; eBioscience; Clone eBR2a; Cat#: 16-4321-85;
Anti-GM-CSF; Bio X cell; Clone MP1-22E9; Cat#: BE0259
Rat IgG2a isotype control antibody; Bio X cell; Clone 3A2; Cat#:BE0089

Western blot:
APG5L Rabbit mAb; Company: Zenbio; Clone: R06-1E5; Cat#: R381320; 1:500
ULK1 Rabbit mAb; Company: Zenbio; Clone: R02-8E7; Cat#: R381887; 1:500
LC3B Rabbit mAb; Company: Zenbio; Clone: R06-4K9; Cat#: R381544; 1:500
GAPDH Rabbit mAb; Company: Zenbio; Clone: R09-4E-1; Cat#: R380626; 1:500
Goat Anti-Rabbit IgG H&L(HRP); Company: Zenbio; Clone: N/A; Cat#: 511203; 1:200
Goat Anti-Mouse IgG H&L(HRP); Company: Zenbio; Clone: N/A; Cat#: 511103; 1:200

Immunofluorescence histology:
APG5L Rabbit mAb; Company: Zenbio; Clone: R06-1E5; Cat#: R381320; 1:100
Anti-human-CD3-BV421; Company: BD Bioscience; Clone: SK7; Cat#: 563797; 1:50
Anti-RORγ; Company: Santa; Clone: 27.92; Cat#: sc-293150; 1:100
Goat Anti-Rabbit IgG H&L(AF488); Company: Abcam; Clone: N/A; Cat#: ab150077; 1:100
Goat Anti-Mouse IgG H&L(AF647); Company: Abcam; Clone: N/A; Cat#: ab150115; 1:100

Chip-PCR:
HIF-1alpha (D1S7W) XP Rabbit mAb; Company: CST; Clone: D1S7W; Cat#: 36169T; 1:100
Normal Rabbit IgG; Company: CST; Cat#: 2729S; 1:100

Validation

Flow cytometry: All antibodies are validated by manufacturer for use in flow cytometry and in their specified species as listed in the "Antibodies used" section above. Prior to data acquisition, we carefully titrated each of these antibodies individually, and then performed optimization of the full panels.

Animals and other research organisms

Policy information about studies involving animals; ARRIVE guidelines recommended for reporting animal research, and Sex and Gender in Research

Laboratory animals

C57BL/6 mice (eight-day-old) were obtained from the Experimental Animal Center of Southern Medical University (Guangzhou, China). Atg5fl/fl mice were provided by Professor Zhexiong Lian (Guangdong Academy of Medical Sciences, Guangzhou, China), with genotyping confirmation. Rorccre mice were provided by Professor Chen Dong (Tsinghua University). Rag2–/– mice (stock no.C000115) with a C.B6(Cg)-Ragtm1.1Cgn/J background and Nfil3–/– mice (stock no. NM-KO-190125) with a C57BL/6JCya-Nfil3em1/Cya background were purchased from Changzhou Cavens Laboratory Animal Co., Ltd. (Changzhou, China) and Shanghai Model Organisms Center, Inc. (Shanghai, China), respectively. All mice were housed in a specific pathogen-free (SPF) barrier animal facility under a controlled 12-h light/12-h dark cycle, constant temperature (22 ± 2°C) and relative humidity (50 ± 10%), with ad libitum access to sterile commercial chow and autoclaved drinking water. Age- and sex-matched littermates were used for all experiments. For experiments comparing healthy breastfed control mice and NEC model experimental mice, the two groups were housed separately to avoid interference from breastfeeding dams with the NEC modeling procedure, while maintaining identical environmental parameters between groups. For experiments comparing different intervention groups within the NEC model (control and experimental groups both subjected to NEC induction), mice were randomly assigned to groups after modeling and co-housed to eliminate cage effect. All animal experimental protocols were approved by the Institutional Animal Care and Use Committee of Southern Medical University (Approval Number: L2023083).

Wild animals

This study did not involve wild animals.

Reporting on sex

This study did not focus on mice of a single gender; all newborn mice from the same litter were randomly assigned to the experiment.

Field-collected samples

This Study did not involve samples collected from the field.

Ethics oversight

All animal experimental protocols were approved by the Institutional Animal Care and Use Committee of Southern Medical University Experimental Animal Ethics Committee (Approval Number: L2023083).

Note that full information on the approval of the study protocol must also be provided in the manuscript.

Plants

Seed stocks

Not applicable.

Novel plant genotypes

Not applicable.

Authentication

Not applicable.

Flow Cytometry

Plots

Confirm that:

☒ The axis labels state the marker and fluorochrome used (e.g. CD4-FITC).

☒ The axis scales are clearly visible. Include numbers along axes only for bottom left plot of group (a 'group' is an analysis of identical markers).

☒ All plots are contour plots with outliers or pseudocolor plots.

☒ A numerical value for number of cells or percentage (with statistics) is provided.

Methodology

Sample preparation

Mouse and human intestinal samples were washed with ice-cold PBS, cut longitudinally, cleaned thoroughly, and mechanically minced into 1-cm pieces with scissors. Intestinal fragments were transferred into Hank's Balanced Salt Solution (HBSS) buffer containing 10 mM EDTA (XPBiomed, Cat#: C3530-0100) and 1 mM dithiothreitol (DTT, Amresco; Cat#: MS5511) and incubated for 30 min at 37 °C on an orbital shaker to remove epithelial cells and mucus. After vortexing and washing twice with PBS, the epithelial fraction was discarded. The remaining tissues were minced into 1-mm pieces and digested in Roswell Park Memorial Institute (RPMI)-1640 medium (BI, Cat#: 01-100-1ACS) supplemented with 5% fetal bovine serum (FBS; BI, Cat#: 04-001-1ACS), 1 mg/mL collagenase I (Gibco, Cat#: 17104019), 100 µg/mL DNase I (Sangon Biotech, Cat#: B002138-0025), 1 mg/mL dispase (Roche, Cat#: 04942078001), and 10 mM HEPES (Beyotime, Cat#: ST092), with incubation for 45 min at 37 °C on an orbital shaker. Digested tissues were filtered through a 70-µm cell strainer, and the cell suspension was resuspended in 40% Percoll (GE Healthcare, Cat#:17-0891-09). LPMCs were enriched by 40%/80% Percoll gradient centrifugation. After centrifugation at 400 × g for 25 min at room temperature, the white intermediate layer was collected and washed with PBS to obtain LPMCs, which were then used for flow cytometry analysis or cell sorting.

Instrument

CytoFLEX S flow cytometer (Beckman Coulter, Brea, CA, USA)
CytoFLEX SRT Cell Sorter (Beckman Coulter, Brea, CA, USA)
BD FACSAria III (BD Biosciences, USA)

Software

FlowJo V10 (Treestar)

Cell population abundance

An EasySep Mouse Pan-ILC Enrichment Kit (StemCell, Canada) was used to sort ILC3s according to the manufacturer's instructions. Briefly, lineage-negative cells were first enriched using the kit, which includes antibodies against mouse lineage markers (CD3e, CD11b, CD11c, CD19, Gr-1, TER119, TCRβ, and TCRγδ). The enriched lineage-negative cells were then labeled with anti-mouse CD90.2-PE (eBioscience, USA) and subjected to positive selection using an EasySep Mouse PE Positive Selection Kit II (StemCell, Canada) to isolate ILC3s. Following this strategy, ILC3s with a purity exceeding 79% were obtained. Approximately 8,000–10,000 intestinal ILC3s can be sorted from each mouse using this method.

Gating strategy

Mouse intestinal DN ILC3s were identified as CD45+CD4−Lin−CD90.2+CD127+RORγt+CCR6−NKp46− cells; NKp46+ ILC3s as CD45+CD4−Lin−CD90.2+CD127+RORγt+CCR6−NKp46+ cells; CCR6+ ILC3s as CD45+CD4−Lin−CD90.2+CD127+RORγt+CCR6 +NKp46− cells; ILC1s as CD45+CD4−Lin−CD90.2+CD127+T-bet+ cells; ILC2s as CD45+CD4−Lin−CD90.2+CD127+GATA3+ cells. The mouse lineage markers included CD3, B220, CD11b, Ly6G, Ter119, CD11c, CD5, CD8a, TCRαβ and TCRγδ. Mouse

intestinal TCRγδ T cells were identified as CD45+TCRγδ+ cells, and TCRαβ T cells as CD45+TCRαβ+ cells. Mouse intestinal IECs were identified as CD45−EpCAM+ cells. Human intestine mucosal ILC3s were identified as CD45+Lin−CD127+CD161+CRTH2 −CD117+ cells. The human lineage markers included CD11b, TCRγδ, TCRαβ, CD14, CD34, CD19, CD3, CD123, CD8, CD5, CD4, CD11c and FceR1. FACS data were acquired using a CytoFLEX S flow cytometer (Beckman Coulter, Brea, CA, USA) and analyzed using FlowJo V10.0.8. Flow cytometry analysis was performed at the Department of Immunology and the Department of Developmental Biology, School of Basic Medical Sciences, Southern Medical University.

☒ Tick this box to confirm that a figure exemplifying the gating strategy is provided in the Supplementary Information.
