# Supplementary material for: Blockade of NKp46⁻ CCR6⁻ ILC3 autophagy protects against necrotizing enterocolitis by restoring energy metabolism balance in mice
Source: Nat Commun. 2026 May 19;17:6579. doi: 10.1038/s41467-026-73356-x (PMC13381918; doi:10.1038/s41467-026-73356-x)
Supplement: Supplementary file 3 — Transparent Peer Review file [file 41467_2026_73356_MOESM3_ESM.pdf]

nature portfolio

Peer Review File

Blockade of NKp46⁻ CCR6⁻ ILC3 autophagy protects against necrotizing enterocolitis by restoring energy metabolism balance in mice

Corresponding Author: Professor Yumei He

This file contains all reviewer reports in order by version, followed by all author rebuttals in order by version.

Version 0:

Reviewer comments:

Reviewer #1

(Remarks to the Author)
The study investigates the role of $NKp46^{-}CCR6^{-}$ (double-negative, DN) ILC3 autophagy in NEC. Using single-cell transcriptomics, T cell–deficient mice, and targeted interventions, the authors show that ATG5-dependent autophagy regulates DN ILC3 metabolism, impacting NEC severity. ATG5 deficiency or autophagy inhibition reduced ILC3 abundance and function, alleviating NEC, while modulation of fatty acid oxidation or lactate restored DN ILC3 function and worsened disease. Phosphatidylcholine supplementation similarly reduced autophagy and inflammation. Clinically, NEC patients exhibited elevated ILC3s and autophagy gene expression. These findings suggest DN ILC3 autophagy as a key metabolic regulator and potential therapeutic target in NEC.
Overall the paper is novel and interesting. However, there are number of issues that need to be addressed.
Major comments:
1). The authors report an increase in ILC3 numbers and subsets, accompanied by elevated type 3 cytokine secretion in NEC. However, previous studies have reported conflicting findings, including decreased IL-22 and unchanged IL-17 (PMID: 33188181) and decreased IL-22 (PMID: 40023163). The authors should clarify how their results reconcile these discrepancies, considering ILC3 subset differences, cytokine-specific functions, and model-dependent effects. Additionally, Figure 1b shows a substantial decrease in ILC2s, suggesting that broader shifts in ILC populations may contribute to NEC pathogenesis. The authors should also address whether the observed increase in ILC3s could be influenced by the gavage procedure, given that control mice were breastfed while NEC mice received oral gavage.
2). The manuscript does not clearly explain how overactivation of IL-17 and IL-22 disrupts intestinal homeostasis. For instance, it is unclear whether this leads to increased intestinal epithelial cell death, barrier dysfunction, or other features of a "leaky gut." Have the authors attempted to rescue the phenotype using neutralizing antibodies against these cytokines? Inclusion of such experiments would strengthen the mechanistic conclusions.
3). Lines 145–148: The authors assert that T cells play a minimal role in NEC development and that DN ILC3s are the primary drivers. However, in the discussion, several cited studies indicate that T cells can significantly contribute to NEC pathogenesis. The authors should address this apparent discrepancy and consider softening their claims regarding the exclusivity of ILC3 involvement.
4). In the discussion, the authors cite "purity" as an experimental limitation, but it is unclear what is meant by this. The manuscript would benefit from a clear presentation of the gating strategies used to quantify ILCs. While the statement in line 319 that CD4—cells do not exclusively represent ILC3s is accurate, additional strategies to exclude T cells- such as gating out TCRα/β or CD3/CD5 positive cells—should be considered and discussed.
5). The single-cell data presented in Figure 1 could be further leveraged to explore ILC3 biology. Analysis of the metabolic profiles of these cells would strengthen the authors' claims and provide additional mechanistic insight.
6). In figure 2, the authors report that transfer of DN ILC3s from control mice induces significant weight loss in recipient mice compared to PBS-treated controls. The manuscript does not provide a clear explanation for this phenotype. The authors should discuss potential mechanisms underlying this effect and, if possible, provide supporting data to clarify how DN ILC3 transfer contributes to weight loss.
7). In Figure 8, the manuscript would benefit from a deeper characterization of the ILC3 compartment. Examining ILC3 subclusters and determining how the double-negative population changes in NEC patients could provide valuable context. Additionally, presenting the expression of effector cytokine transcripts such as IL17 and IL22 in patient samples would help bridge the murine findings with the human setting and strengthen the overall translational impact of the study

Reviewer #2

(Remarks to the Author)
In this manuscript, He and colleagues sought to define how ILC3s drive immune inflammation that drives necrotizing enterocolitis (NEC), a serious ailment in preterm neonates. In summary, the authors found that DN ILC3s are the crucial subset driving this disease, through an increase in autophagy that drives ILC3 pro-inflammatory responses. More specifically, autophagy in DN ILC3s was found to induce glycolysis at the expense of lipid metabolism, and ATG5 or autophagy-deficient ILC3s revert this phenotype. To reach these conclusions, the authors used a combination of single cell transcriptomics analysis, in vivo models of NEC in T cell-deficient mice and specific interventions on distinct molecular pathways.

This is an interesting work that clearly indicates an important role for ILC3 autophagy in the promotion of NEC. However, there are important experiments and issues that need to be addressed in order to unequivocally prove some of the conclusions stated, especially the confirmation that DN ILC3s are, indeed, the major contributor for NEC, as well as additional exploration about how DN ILC3 autophagy leads to glycolysis-mediated cytokine production, which seems counterintuitive compared to the canonical function of autophagy. Specific points are listed below:

Major:
- The findings that autophagy induces glycolytic function in DN ILC3s are counterintuitive. In many cases, an inhibition of autophagy would be associated with an increase in aerobic glycolysis as a compensatory mechanism. Is the phenotype observed in Figure 5 uniquely found on DN ILC3 cells from neonates? How about DN ILC3 cells in other tissues or from adults?

- Mechanistically, how would autophagy inhibition mediate lipid absorption and inhibition of glycolysis? It would be important to further analyze these non-canonical connections. One example would be to investigate, in NEC DN ILC3 cells, whether transcription factors associated with glycolysis (e.g., Myc) are being preferentially degraded (in comparison to other cells). Another important assessment could be the tracking of the epigenetic state (using either ATACseq or CHIPseq associated with glycolysis-initiating TFs) of the genes associated with glycolysis versus fatty acid uptake.

- The DN ILC3 transfer experiments into WT or Rag2KO mice are important to establish some causal relation, but there are some potential confounding effects due to the presence of ILCs in host mice – that may explain why the differences observed are somewhat small. It would be important to perform experiments where this transfer is done into ILC- or ILC3-deficient mice, such as Nfil3-KO or even into Rag2-KO x Rorc-KO.

- Autophagy (or at least Atg5) seems to be increased not only on DN ILC3s, but also on other ILC3 subsets. Are there any pathways specifically enriched in DN ILC3s? That would be worth checking, since the authors claim that DN ILC3s play an apparent dominant role in NEC.

- Alternatively, is the autophagy pathway differentially regulating DN ILC3s, if compared to other ILC3 subsets? It would be interesting to evaluate if canonical downstream effects of autophagy are triggered differently between ILC3 subsets.

- Is Glut1 expression (or other Glucose transporters) altered in NEC DN ILC3s? What is the main fuel for glycolysis in NEC DN ILC3s? Ideally, experiments using carbon-labeled glucose or lactate in vitro/in vivo would showcase which carbon source is being used. I, of course, understand that such experiments may be challenging. Instead, in vitro experiments using exogenous lactate or glucose and an evaluation of glycolysis in NEC DN ILC3s should provide enough evidence for the identification of the carbon source used by these cells.

Minor:
- A revision of text for clarity and minor typos will enhance the quality of the manuscript.

- The experiments with lactate and etomoxir should also include Atg5fl controls.

Reviewer #3

(Remarks to the Author)
Blocking NKp46-CCR6-ILC3 autophagy alleviates necrotizing enterocolitis by restoring energy metabolism balance
Reviewer Comments

Summary:
The manuscript by He et al. investigates the role of ILC3s in necrotizing enterocolitis (NEC) through the NKp46–CCR6 autophagy pathway. Using single-cell transcriptomics, murine models, and targeted interventions, the authors show that attenuation of NEC can be achieved by reducing ILC3 abundance via ATG5 knockdown or autophagy inhibition. They further link enhanced fatty acid metabolism with reduced glycolysis, while inhibition of fatty acid oxidation restored functionality of ATG5-deficient NKp46–CCR6– ILC3s, exacerbating disease. Phosphatidylcholine (PC) supplementation improved intestinal inflammation by inhibiting NKp46–CCR6– autophagy. The clinical relevance of this work is supported by patient data demonstrating elevated ILC3 levels and enrichment of autophagy-related genes in NEC intestines. Given the

complex and poorly understood pathophysiology of NEC, and the ongoing search for novel therapeutic strategies, this study will be of interest to the readership of Nature Communications, though several substantive issues, particularly relating to ILC3 subsets and the autophagy-metabolism-ILC3-NEC axis, require additional data clarification before publication.

Major Comments:

Novelty
1. The manuscript overstates novelty. Prior work has already implicated ILC3 dysregulation in NEC (e.g., Cho et al., Nat Commun 2020; Zhao et al., Cell Biosci 2022). While focusing on DN ILC3s is of interest, the functional distinction between DN, CCR6+, and NKp46+ subsets is incompletely addressed. More work is needed to convincingly establish DN ILC3s as the causal drivers of NEC pathology.
2. The role of autophagy in immune cell metabolic regulation is well-established. The incremental advance here is its application to DN ILC3s in NEC, which is narrower in scope than framed.
Introduction
3. The introduction states that NEC affects the terminal ileum and proximal colon. While colonic involvement can occur, it is less common; NEC primarily impacts the small intestine, particularly the distal ileum. This statement should be revised to avoid overemphasizing colonic disease and to more accurately reflect the typical distribution of NEC.
4. The authors describe an exaggerated neutrophil response as a contributor in the context of IL-17/IL-22 signaling. For clarity and relevance, this section would be stronger if discussed in the context of small bowel pathology, given that NEC predominantly affects the small intestine.
5. The last paragraph identifies the novel hypothesis (autophagy–energy metabolism axis in DN ILC3s during NEC) but comes after a large amount of mechanistic background. The novelty could be signposted earlier in the introduction (e.g., after briefly describing known ILC3 subsets, stating that DN ILC3s remain poorly understood in NEC). This would make the study's rationale more obvious to readers upfront.
6. It would be helpful if the introduction framed not only the mechanistic gap but also the translational implications—how studying DN ILC3 autophagy could ultimately inform NEC prevention, earlier diagnosis, or therapy in preterm infants. This would better justify the clinical importance of the work.
Results
7. The gating strategy used to define CD4−RORγt+ cells as ILC3s requires stronger validation. Although lineage-negative gating was applied, additional confirmation that these cells are TCR− (e.g., explicit staining for TCRβ and TCRγδ within the RORγt+ population) is necessary to fully exclude unconventional T cells. Because the main conclusion of the manuscript is that DN ILC3s, and not Th17 cells, drive NEC, rigorous exclusion of T cell contaminants is critical to support the authors' claim.
8. Both NKp46+ and CCR6+ ILC3s expand numerically following NEC induction, yet the manuscript highlights DN ILC3s as the primary pathogenic subset. While the abundance of DN ILC3s is clear, relative numbers alone do not establish causality. Functional comparisons across subsets—for example, assessing per-cell cytokine output of purified NKp46+, CCR6+, and DN ILC3s—would more convincingly demonstrate that DN ILC3s are uniquely pathogenic in NEC.
9. Adoptive transfer experiments show NEC-derived DN ILC3s exacerbate disease, but equivalent testing with NKp46+ and CCR6+ ILC3s is missing. Without side-by-side comparisons, it is premature to single out DN ILC3s as uniquely pathogenic.
10. The pharmacological interventions (3-MA and rapamycin) have pleiotropic effects across multiple cell types. While Atg5 conditional knockout experiments support the findings, the authors should explicitly address the limitations of systemic inhibition and how this might affect interpretation.
11. The data show that Atg5 deficiency in DN ILC3s correlates with lipid accumulation, reduced glycolysis, and altered cytokine output. However, it remains unclear whether the metabolic reprogramming observed is the mechanistic driver of functional changes or a secondary effect of Atg5 loss. Rescue experiments that restore glycolytic flux (or alternatively suppress FAO) in Atg5-deficient DN ILC3s would provide stronger evidence of causality between metabolic state and pathogenic function.
12. Phosphatidylcholine supplementation is presented as therapeutic, but the mechanism remains speculative. Is PC acting directly on ILC3 metabolism, altering epithelial responses, modulating the microbiome, or acting through some other mechanism? No evidence distinguishes these possibilities.
13. While human NEC tissue data are valuable, further detail is needed on patient sample size, gestational age, and NEC severity for clinical relevance. It is also unclear whether autophagy upregulation is unique to ILC3s versus other immune subsets in the human NEC tissue. Expanding or clarifying this would strengthen the translational impact.
Discussion
14. Consider using schematic figures to illustrate the proposed autophagy-metabolism-DN ILC3-NEC axis.
15. While Rag2 KO adoptive transfer experiments are informative, differences from previous studies (e.g., CD4+ T cell contribution) are attributed to cell number and model differences. It may strengthen the discussion to include quantitative data or additional controls showing that DN ILC3-mediated inflammation is not influenced by other adaptive immune cells.
16. The discussion overall downplays the inconsistencies with prior literature regarding T cell contributions. Simply attributing discrepancies to "differences in model construction" is insufficient. Direct comparative data or stronger rationale is required.
17. The discussion highlights alterations in glycolysis, fatty acid oxidation, and glycerophospholipid metabolism. While intriguing, the mechanisms connecting PC supplementation to ILC3 functional changes are not fully defined. A more detailed mechanistic hypothesis, or acknowledgment of knowledge gaps, would be valuable for readers.
18. The study proposes targeting the autophagy-energy metabolism axis as a therapeutic strategy for NEC. Consider discussing whether this axis is truly specific to NEC pathophysiology.
19. The potential translational challenges, including specificity, timing, and safety of manipulating autophagy or metabolism to "regulate intestinal homeostasis", as the authors suggest, needs to be addressed, especially in physiologically vulnerable neonates. The translational leap from murine NEC to neonatal therapy is considerable. The discussion should grapple more

seriously with challenges of manipulating autophagy or metabolism.

Minor Comments:
20. There is an extra comma in line 76 of the introduction: “CCR6+ILC3s are enriched in adult mice guts,, providing an innate source of IL-17A, IL-17F, IL-22, and GM-CSF resulting from pathogen infection and IL-23 stimulation.”
21. The manuscript would benefit from careful language editing for readability (e.g., occasional run-on sentences).

Overall Assessment:
This is a well-designed study with a potential to advance our understanding of NEC pathophysiology, adding on the current literature of ILC3s in NEC. The integration of scRNA-seq, genetic models, metabolomics, and human validation is sound. However, the current data fall short of Nature Communications standards due to concerns about novelty, experimental rigor, mechanistic clarity, and translational depth. Substantial revisions—particularly more rigorous cell validation, functional comparisons across ILC3 subsets, mechanistic rescue experiments, and clearer human clinical data—would be necessary before the work is suitable for publication. Addressing these points may require additional analyses, particularly to substantiate the mechanistic findings. Additionally, including a schematic figure to illustrate the proposed mechanism would greatly enhance clarity and accessibility of the work.

Recommendation: Major Revision
While the study is fundamentally strong, additional experimentation should be considered before publication.

Reviewer #4

(Remarks to the Author)
I co-reviewed this manuscript with one of the reviewers who provided the listed reports. This is part of the Nature Communications initiative to facilitate training in peer review and to provide appropriate recognition for Early Career Researchers who co-review manuscripts.

Reviewer #5

(Remarks to the Author)
In this study, He and colleagues propose that ILC3s play a pathogenic role in necrotizing enterocolitis (NEC) by secreting cytokines that exacerbate disease severity. Mechanistically, the authors demonstrate that this detrimental effect is linked to increased autophagy, which in turn alters the metabolic programming of ILC3s. Deletion of Atg5 in RORγt$^+$ cells, reduces disease pathology during NEC, shifts ILC3 metabolism toward fatty acid utilization, and leads to the accumulation of phosphatidylcholine (PC). Finally, supplementation with PC alleviates NEC symptoms, suggesting that PC administration may represent a potential therapeutic intervention for neonatal NEC.
Overall, the study presents several interesting findings. However, a number of important points remain unclear:
1. ILC2 involvement: In the scRNA-seq data shown in Fig. 1, there appears to be not only an increase in ILC3s but also a notable decrease in ILC2s. Do ILC2s play any role in NEC? Would adoptive transfer of ILC2s from adult mice in the NEC setting improve or worsen disease outcomes?
2. ILC3 subset interpretation: The authors focus on double-negative (DN) ILC3s and emphasize their specific role in NEC. However, prior studies have shown that DN ILC3s are precursors of NKp46$^+$, T-bet–dependent ILC3s. Because NKp46$^+$ ILC3s begin to expand around day 15 of life, it is possible that at day 8 the ILC3 compartment consists predominantly of DN ILC3s, and the observed phenomena may reflect ILC3 biology in general rather than a DN-specific effect. This possibility should be carefully considered and discussed.
3. Gating strategy in Fig. 1c: It is unclear why the authors gated on CD45$^+$Lin$^-$CD4$^-$CD90.2$^+$ cells. If anti-CD3 is included in the lineage cocktail, excluding CD4$^+$ cells would inadvertently remove CCR6$^+$ LTi-like ILC3s, which could substantially affect downstream analyses and interpretation.
4. Concerns about ILC gating (Supplementary Fig. 1d): The ILC gating strategy raises concerns, as more than 50% of the CD45$^+$ cells appear to be CD90.2$^+$—a surprisingly high proportion. This would only be plausible if the samples were pre-enriched using the Stemcell Pan-ILC kit, which should be clearly stated in the figure legend.
5. Contradictory findings regarding IL-22: A recent study (Zhang et al., Immunity, 2025) demonstrated that ILC3-derived IL-22 is protective in NEC. In contrast, the present work suggests a detrimental role. Cytokine-blocking experiments targeting IL-22, IL-17, and/or GM-CSF should be performed to distinguish which ILC3-derived cytokines are pathogenic versus beneficial.
6. Evidence for autophagic vesicles: The authors should provide a higher-resolution image demonstrating the presence of autophagic vesicles in NEC ILC3s. In Fig. 3c, it is not clear whether the observed structures possess double or single membranes.

Version 1:

Reviewer comments:

Reviewer #1

(Remarks to the Author)

The authors have adequately addressed all of the concerns raised during the review process, and I have no further comments at this time.

Reviewer #2

(Remarks to the Author)
I thank the authors for thoroughly addressing all my comments. The revised manuscript is significantly improved in quality and I believe it is suitable for publication at Nature Communications.

Reviewer #3

(Remarks to the Author)
General Comments: The authors have provided a comprehensive response that significantly strengthens the manuscript's scientific rigor. The technical improvements in gating resolve previous ambiguities. Furthermore, by moving from correlative observations to functional validation, specifically through adoptive transfers and metabolic rescue experiments, the authors have strengthened the mechanistic link between Atg5-dependent autophagy, HIF-1α stability, and ILC3 pathogenicity. The work now meets the mechanistic depth expected for Nature Communications.

Evaluation of Revisions

1. Functional Validation of the Pathogenic ILC3 Subset
The authors have addressed the previous ambiguity regarding which ILC3 subset drives the NEC phenotype. The newly included adoptive transfer experiments comparing NKp46+, CCR6+, and NKp46-CCR6- (Double Negative) ILC3 subsets side-by-side, provide the functional granularity required to support that DN ILC3s are uniquely pathogenic in this model. By demonstrating that only the DN subset significantly exacerbates clinical distress and pro-inflammatory cytokine profiles (IL-17A), the authors have strengthened the evidence for this specific population. Additionally, the inclusion of a gavage-only control group effectively rules out the stress of the procedure as a confounding variable.

2. Technical Validation and Gating Strategy
The technical quality of the flow cytometry data has been improved. The refined gating strategy, specifically the explicit exclusion of TCRαβ+ and TCRγδ+ cells, provides the necessary confidence that the observed effects are intrinsic to ILC3s and not driven by contaminating T-cell populations.

3. Mechanistic Depth: The Autophagy-Metabolism Axis
This revision provides a more robust mechanistic framework for how autophagy dictates ILC3 pathogenicity. The authors utilized ATAC-seq and ChIP-qPCR to demonstrate that Atg5-dependent autophagy is required for the stabilization of HIF-1α and its subsequent binding to the promoters of glycolytic genes (e.g., Ldha, Pkm2). This provides a molecular explanation of how autophagy-deficient cells undergo metabolic exhaustion. The bioenergetic data from the Seahorse XF assays further support this, showing that inhibiting autophagy blunts the glycolytic shift associated with the pro-inflammatory function of these cells in the neonatal gut.

4. Establishing Causality in Metabolic Reprogramming
In response to concerns regarding whether metabolic reprogramming was a driver or a secondary effect of Atg5 loss, the authors provided supplementary mechanistic data. By demonstrating that restoring glycolytic flux or stabilizing HIF-1α in Atg5-deficient DN ILC3s partially rescues IL-17A production and disease pathogenicity, they have established a stronger link, showing that the autophagy-mediated metabolic state is a functional driver of the cell's pathogenic behavior.

5. Pharmacological vs. Genetic Precision
The authors have addressed the pleiotropic risks of systemic 3-MA and Rapamycin treatment. By emphasizing their Atg5 conditional knockout (RorccreAtg5fl/fl) data as the primary evidence, and adding an explicit discussion regarding the limitations of systemic pharmacological inhibition, the authors have provided a more balanced interpretation of their intervention data.

6. Clarification of Therapeutic Mechanisms (Phosphatidylcholine)
While the role of Phosphatidylcholine (PC) remains multifaceted, the authors have provided data to better distinguish its effects. Their revisions suggest that PC supports intestinal barrier integrity and epithelial metabolic fitness rather than acting as a direct signaling ligand for ILC3s. The addition of these caveats and the preliminary epithelial data provide a more grounded therapeutic rationale.

7. Clinical Relevance and Translational Insight
The authors have expanded their analysis of human infant NEC samples, providing better clinical context. The confirmation that the expansion of DN ILC3s and their associated autophagy markers correlates with human disease strengthens the translational narrative. The finding that neutralizing IL-17A (but not IL-22) rescues the phenotype in their model offers a more evidence-based therapeutic lead.

8. Integration of the Visual Mechanistic Model
The updated summary figure is an important addition in this revision. It effectively synthesizes the experimental findings into a cohesive signaling axis: Autophagy HIF-1α stability Glycolytic Flux IL-17A Secretion Intestinal Damage. This model is now better supported by the new data provided in the revision and serves as a useful framework for the study's conclusions.

Final Recommendation: The authors have been responsive to the previous round of reviews, performing a substantial volume of new experiments that have significantly improved the scientific rigor of the manuscript. The work is now technically sound and the mechanistic claims are better supported. I recommend the manuscript for publication in Nature Communications.

Reviewer #4

(Remarks to the Author)
I co-reviewed this manuscript with one of the reviewers who provided the listed reports. This is part of the Nature Communications initiative to facilitate training in peer review and to provide appropriate recognition for Early Career Researchers who co-review manuscripts.

Reviewer #5

(Remarks to the Author)
The authors have satisfactorily addressed my concerns by providing substantial additional experimental data that reinforce and support their original conclusions.

Open Access This Peer Review File is licensed under a Creative Commons Attribution 4.0 International License, which permits use, sharing, adaptation, distribution and reproduction in any medium or format, as long as you give appropriate credit to the original author(s) and the source, provide a link to the Creative Commons license, and indicate if changes were made.
In cases where reviewers are anonymous, credit should be given to 'Anonymous Referee' and the source.
The images or other third party material in this Peer Review File are included in the article's Creative Commons license, unless indicated otherwise in a credit line to the material. If material is not included in the article's Creative Commons license and your intended use is not permitted by statutory regulation or exceeds the permitted use, you will need to obtain permission directly from the copyright holder.
To view a copy of this license, visit https://creativecommons.org/licenses/by/4.0/

Point-by-point response

On behalf of all contributing authors, I would like to express our sincere appreciation for the valuable commentsfrom the reviewers on our manuscript titled "Blockade of NKp46$^-$CCR6$^-$ILC3autophagy protects against necrotizing enterocolitis via restoring energy metabolismbalance" (NCOMMS-25-63020-T). We are grateful for the time and effort you and thereviewers dedicated to providing feedback. The insightful comments have improvedthe clarity and scientific rigor of our manuscript.

In response to the reviewers' feedback, we have extensively revised our manuscript and added new data to strengthen our findings. We have addressed all questions point by point, referencing corresponding page and line numbers. Below is a summary of the main revisions.

(1) We have established a gavage control group to exclude the confounding effects of the procedure. Oral gavage did not induce intestinal inflammation or alter the proportions of innate lymphoid cell (ILC) subsets in neonatal mice.

(2) We have provided detailed gating strategies and validation data. TCRαβ and TCRγδ staining confirmed minimal T-cell contamination in the CD4$^-$RORγt$^+$ population, and its frequency remained unchanged after NEC induction.

(3) Adoptive transfer of NKp46$^-$CCR6$^-$ double negative (DN) ILC3s—not NKp46$^+$ or CCR6$^+$ ILC3s—exacerbated the severity of necrotizing enterocolitis (NEC) in neonatal models. Single-cell analyses further revealed that the pathogenic effects of DN ILC3s were specifically driven by IL-17A.

(5) We have confirmed the specific role of IL-17A in intestinal barrier function during NEC, showing that neutralizing IL-17A, not IL-22 or GM-CSF, alleviated weight loss and intestinal inflammation in neonatal mice.

(6) We have conducted adoptive transfer of DN ILC3s from *Atg5*-deficient or control littermates into *Nfil3*-deficient recipients. These experiments excluded potential confounding by endogenous ILCs and confirmed the pathogenicity of DN ILC3s during NEC development.

(7) Analysis of ATAC-seq highlighted a unique autophagy–metabolism axis in DN ILC3s. Mechanistically, ATG5-dependent HIF-1α binding to glycolytic genes drove the pathogenicity of DN ILC3s during NEC.

(8) We have elucidated the multi-target mechanism of phosphatidylcholine (PC) in NEC. PC directly inhibited IL-17A/IL-22 secretion, reduced ATG5 expression, and induced metabolic reprogramming in DN ILC3s. Moreover, PC protected intestinal barrier integrity and reshaped the gut microbiome by enhancing *Clostridia* abundance.

(9) We have validated the clinical relevance by showing that DN ILC3s, a subset predominant in the infant intestine, were expanded in NEC patients and associated with elevated IL-17A/IL-22 levels.

(10) Adoptive transfer of neonatal—but not adult—ILC2s significantly ameliorated NEC. In addition, comparing the effects of DN ILC3 and T cell transfer further revealed distinct roles for these immune subsets in disease progression.

(11) We have thoroughly revised the Introduction and Discussion. Specifically, we reframed our study's innovation to avoid overstatement; clarified that NEC predominantly affects the terminal ileum; and refined our central hypothesis and its translational relevance. We also clarified discrepancies with prior findings by comparing our data to the literature. To improve conceptual clarity, we added a mechanistic model of the autophagy–metabolism–DN ILC3 axis in NEC pathogenesis. Additionally, the manuscript has been edited by Editage (www.editage.cn) to improve language quality, and all figures have been replaced with high-resolution versions.

For convenience, all changes in the revised manuscript are **indicated in blue text**. We hope you find our revised version convincing to meet your standards for publication.

Response to Reviewer #1:

We are grateful for your through and insightful feedback of our manuscript. Your constructive comments have significantly strengthened our conclusions and enhanced the overall quality of this work. To address all points raised, we have conducted supplementary experiments and revised the manuscript accordingly, with all changes **indicated in blue text**. Pleases refer to the detailed, point-by-point responses provided below. We sincerely hope that this revised version now meets your expectations and earns your approval.

Reviewer #1 (Comments to the Author):

The study investigates the role of NKp46^{-}CCR6^{-} (double-negative, DN) ILC3 autophagy in NEC. Using single-cell transcriptomics, T cell–deficient mice, and targeted interventions, the authors show that ATG5-dependent autophagy regulates DN ILC3 metabolism, impacting NEC severity. ATG5 deficiency or autophagy inhibition reduced ILC3 abundance and function, alleviating NEC, while modulation of fatty acid oxidation or lactate restored DN ILC3 function and worsened disease. Phosphatidylcholine supplementation similarly reduced autophagy and inflammation. Clinically, NEC patients exhibited elevated ILC3s and autophagy gene expression. These findings suggest DN ILC3 autophagy as a key metabolic regulator and potential therapeutic target in NEC.
Overall the paper is novel and interesting. However, there are number of issues that need to be addressed.

Reply: We sincerely appreciate your constructive and insightful comments.

1. The authors report an increase in ILC3 numbers and subsets, accompanied by elevated type 3 cytokine secretion in NEC. However, previous studies have reported conflicting findings, including decreased IL-22 and unchanged IL-17 (PMID: 33188181) and decreased IL-22 (PMID: 40023163). The authors should clarify how their results reconcile these discrepancies, considering ILC3 subset differences, cytokine-specific functions, and model-dependent effects. Additionally, Figure 1b shows a substantial decrease in ILC2s, suggesting that broader shifts in ILC populations may contribute to NEC pathogenesis. The authors should also address whether the observed increase in ILC3s could be influenced by the gavage procedure, given that control mice were breastfed while NEC mice received oral gavage.

Reply: We sincerely apologize for the insufficient clarity in our original manuscript. We greatly appreciate this critical and constructive comment, which has guided us to systematically compare our work with the previous reports (PMID: 33188181, PMID: 40023163). Therefore, we have clarified the consistencies and discrepancies between our findings and prior studies as follows.

- Our study shares core consensus with Cho et al. (*Nat. Commun.*, 2020, 10.1038/s41467-020-19400-w, PMID: 33188181): both studies demonstrate that ILC3 subset imbalance and type 3 immune polarization are critical immunopathological features of NEC. While previous work broadly categorized ILC3s into $NKp46^+$ and $NKp46^-$ populations, we subdivided them into three functionally distinct subsets using scRNA-seq and flow cytometry, and identified DN ILC3s as the key pathogenic subset via adoptive transfer experiments. Regarding cytokine source, prior studies measured bulk tissue, whereas we validated ILC3-subset-specific cytokines; we confirmed that increased DN ILC3-derived IL-17A drives NEC pathogenesis, and reduced total IL-22 levels in prior work resulted from the ablation of protective $NKp46^+$ ILC3s, without essential contradiction between these findings. Cho et al. also reported elevated IL-17 and IL-22 levels in mouse NEC tissue, corroborating our findings of dysregulated type 3 cytokines. Notably, we noted potential model-dependent effects: previous studies used postnatal 3-day neonatal mice (immature immune system), whereas our P8 model more closely aligns with the clinical high-incidence period of human NEC.
- We wish to clarify that there are no conflicting conclusions between our work and the study by Zhang et al. (*Immunity*, 2025, 10.1016/j.immuni.2025.02.003, PMID: 40023163). Instead of directly quantifying IL-22 levels in NEC model mice or patients, Zhang et al. used epithelial-specific conditional knockout mice to infer potential reduction in IL-22 levels during NEC based on the protective role of epithelial-derived signaling. In contrast, we directly assessed IL-17A and IL-22 production by flow cytometry-sorted ILC3s from human NEC infants, demonstrating elevated levels of these cytokines specifically in ILC3s—consistent with our human scRNA-seq data. Thus, while Zhang et al. highlighted a potential reduction in overall IL-22 signaling through epithelial mechanisms, our study reveals that ILC3s themselves are a source of increased IL-22 during NEC. These findings are not contradictory but rather reflect different biological compartments and methodological approaches.

In the revised manuscript, we added ILC3 subset comparative data, supplemented relevant detailed discussion and revised overgeneralized statements to improve conclusion precision.

Previous studies have reported elevated IL-17 and IL-22 expression in mouse NEC intestinal tissues, while loss of NKp46^{+} ILC3s has been shown to reduce IL-22 expression (*Nat. Commun.*, 2020, 10.1038/s41467-020-19400-w, PMID: 33188181). Additionally, intestinal epithelial cell-specific gene knockout has also been implicated in regulating IL-22 expression (*Immunity*, 2025, 10.1016/j.immuni.2025.02.003, PMID: 40023163). To identify cytokines mediating the pathogenic role of DN ILC3s, we re-analyzed scRNA-seq data and validated findings by quantifying cytokine production *in vitro*. Integrated analysis consistently showed that IL-17A—not IL-22 or GM-CSF—was significantly upregulated in DN ILC3s after NEC induction. Moreover, only IL-17A neutralization—not IL-22 or GM-CSF neutralization—significantly reduced disease severity in murine NEC models, confirming the central role of IL-17A in NEC progression. These observations align with Mihi et al., who found no increase in intestinal IL-22 after NEC induction and no increased NEC susceptibility in *Il-22*-deficient mice (*Cell Rep. Med.*, 2021, 10.1016/j.xcrm.2021.100320, PMID: 34195684). However, our IL-22 neutralization results differ from Zhang et al., who reported worsened inflammation (*Immunity*, 2025, 10.1016/j.immuni.2025.02.003, PMID: 40023163). The discrepancy is likely due to the differing frequency of antibody application: they administered anti-IL-22 antibody continuously over four days, whereas we used two injections at the same dose, in line with our published protocol (*Nat. Med.,* 2018, 10.1038/nm.4467, PMID: 29334374)—suggesting sustained neutralization may be required for effective IL-22 suppression, while transient blockade may be insufficient during active inflammation. These findings highlight dosing strategy as a key variable in interpreting cytokine neutralization data. Our results further implicate IL-17A as a promising therapeutic target for NEC.

The corresponding description have been incorporated into the revised version (Page 24, Lines 529–545).

(2) We sincerely thank our reviewer for the insightful comment on the shifts in intestinal ILC populations, especially decreased ILC2 levels shown in Figure 1b. To assess the involvement of ILC2s in NEC, we have validated their alterations using flow cytometry and clarified their contributions through adoptive transfer experiments.

- Flow cytometry analysis showed that ILC3 abundances were significantly higher than those of ILC1s and ILC2s in the neonatal intestines, and were markedly

elevated during NEC. ILC2s were reduced following NEC induction, whereas ILC1s showed no significant change (Figure A a, b).

- Given the reduction in ILC2s during NEC, we next determined the specific role of these cells. 8-day-old wild-type pups were transferred with either PBS or 3×10^4 physiological ILC2s prior to NEC induction (Figure B a). Notably, transfer of ILC2s attenuated weight loss and reduced intestinal inflammation (Figure B b–d).

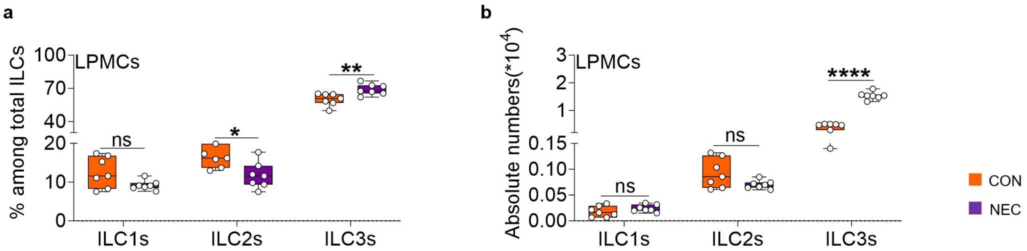

Figure A. ILC subset frequency and absolute count in CON and NEC mice. (**a**, **b**) Percentage and absolute number of intestinal ILC1s, ILC2s, and ILC3s (n = 7). Each symbol represents one pup from a litter, and data are from at least three independent experiments. Error bars represent the SEM. *p < 0.05; **p < 0.01; ***p < 0.001; ns, not significant. Statistical significance was analyzed by Student's t test (**a** and **b**).

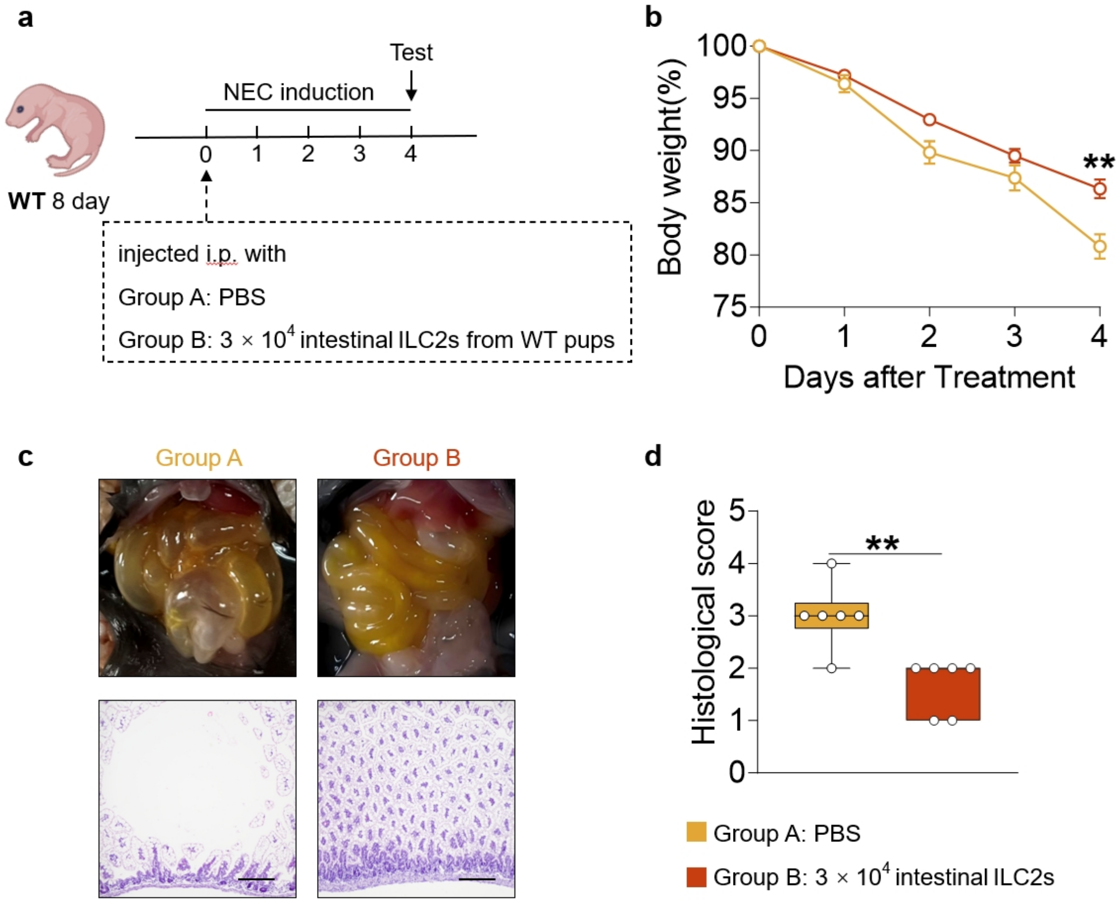

Figure B. Adoptive transfer of physiological ILC2s alleviates neonatal NEC. (**a**) Schematic diagram of adoptive transfer prior to NEC induction. (**b**) Body weight changes in the indicated groups (n = 6). (**c**) Macroscopic image and H&E staining of the intestines (scale bar: 100μm). (**d**) Statistical analysis of inflammation scores (n = 6). Each symbol represents one pup from a litter, and data are from at least three independent experiments. Error bars represent the SEM. *p < 0.05; **p < 0.01; ***p < 0.001; ns, not significant. Statistical significance was analyzed by Student's t test (**b** and **d**).

These findings demonstrate that ILC2s play a protective role in NEC pathogenesis. The precise mechanisms, and how the dynamic balance among ILC subsets influences disease progression, will be important directions for our future investigations.

(3) To determine whether the observed increase in ILC3s was influenced by the gavage procedure, littermates were divided into two groups: one group continued breastmilk feeding only, and the other received PBS by oral gavage four times daily while continuing breastfeeding (Figure C a). No significant differences were observed in weight change or intestinal inflammation between the two groups (Figure C b–d). Moreover, the oral gavage procedure did not influence the percentages and absolute numbers of intestinal CD45$^+$ cells, ILC1s, ILC2s, and ILC3s (Figure C e–l).

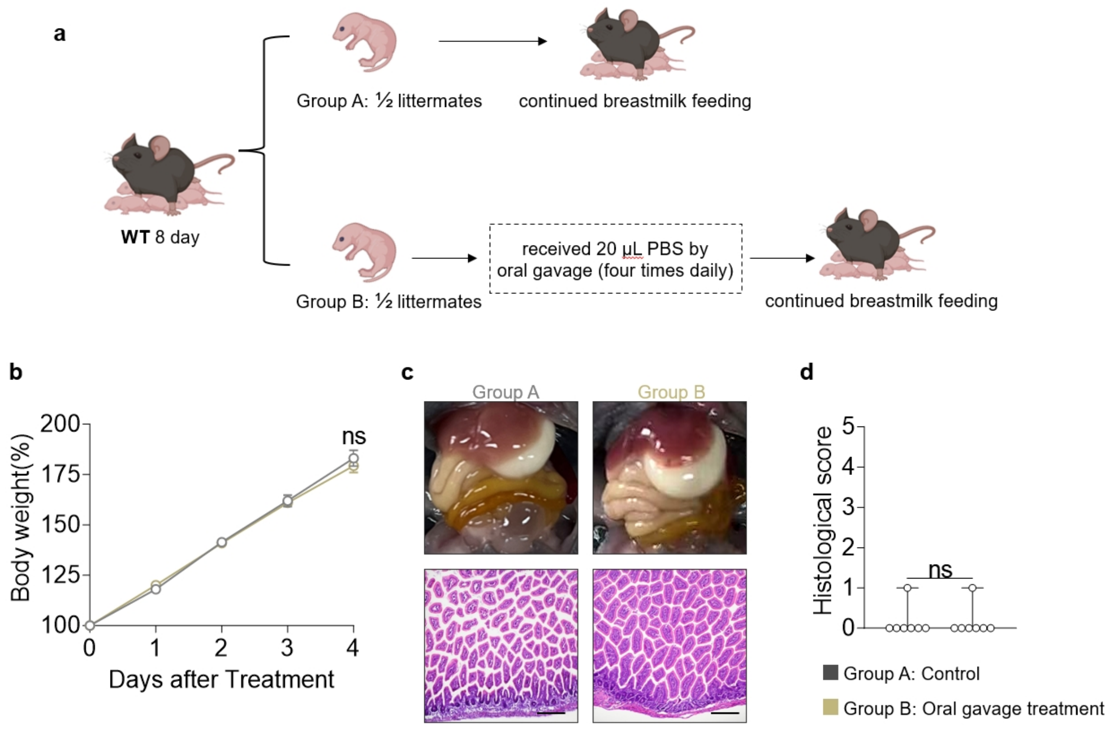

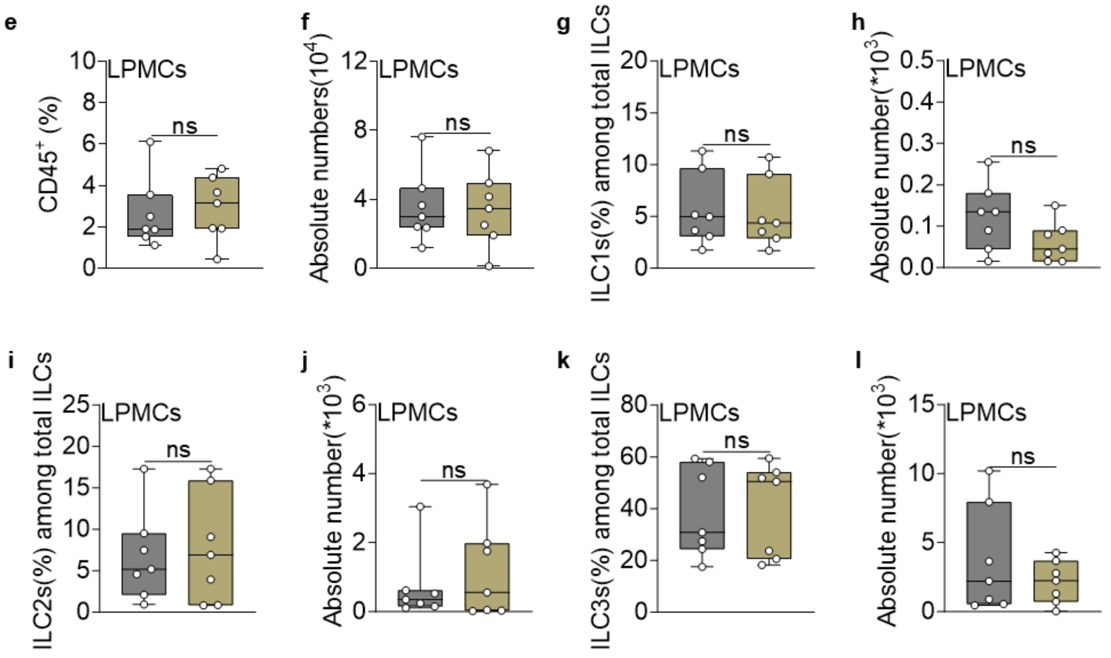

Figure C. Oral gavage procedure does not induce intestinal inflammation and response. (**a**) Experimental schema of oral gavage procedure. (**b**) Body weight changes in the indicated groups (n = 7). (**c**) Macroscopic image and H&E staining of the intestines (scale bar: 100μm). (**d**) Statistical analysis of inflammation scores (n = 7). (**e**, **f**) Percentage and absolute number of intestinal $CD45^+$ cells (n = 7). (**g**–**l**) Percentage and absolute number of intestinal ILC1s, ILC2s, and ILC3s (n = 7). Each symbol represents one pup from a litter, and data are from at least three independent experiments. Error bars represent the SEM. *$p < 0.05$; **$p < 0.01$; ***$p < 0.001$; ns, not significant. Statistical significance was analyzed by Student's t test (**b** and **d**–**l**).

These findings confirm that the gavage procedure does not induce intestinal inflammation or alter intestinal ILC subsets, suggesting that the observed changes in ILC3s are not attributable to the experimental manipulation.

The supplementary data and corresponding description have been incorporated into the revised version (Figure 1c; Supplementary Figure 1a–c; Supplementary Figure 3a–d; Supplementary Figure 9a–c), (Page 6, Lines 114–119; Page 7, Lines 136–140; Page 7, Lines 140–141; Page 9-10, Lines 200–205).

2. The manuscript does not clearly explain how overactivation of IL-17 and IL-22 disrupts intestinal homeostasis. For instance, it is unclear whether this leads to increased intestinal epithelial cell death, barrier dysfunction, or other features of a "leaky gut." Have the authors attempted to rescue the phenotype using neutralizing antibodies against these cytokines? Inclusion of such experiments would strengthen the mechanistic conclusions.

Reply: We sincerely appreciate your insightful comments and fully agree on the importance of clarifying the regulatory roles of IL-17 and IL-22 on intestinal homeostasis. As suggested, we have evaluated the alterations of intestinal barrier function under NEC conditions, and performed neutralizing antibody experiments corresponding to these two cytokines. The detailed results are presented as follows:

- Following NEC induction, intestinal permeability, shown as plasma FD7000 levels, was significantly increased (Figure D a, b). Flow cytometry analysis revealed that both the percentages and absolute numbers of intestinal epithelial cells (IECs) were reduced in NEC-induced pups, accompanied by elevated cell death and reduced proliferation (Figure D c–i). Moreover, several key tight junction proteins, including *ZO-1*, *Claudin-1*, and *Occludin*, were downregulated following NEC induction (Figure D j). These data indicate an intestinal barrier dysregulation in NEC.
- To clarify the causal link between IL-17A/IL-22 overactivation and intestinal barrier disruption, we performed neutralizing antibody experiments corresponding to these cytokines. Anti-IL-17A or IgG control were intraperitoneally injected into wild-type pups during NEC induction (Figure E a). Notably, anti-IL-17A treatment significantly improved weight retention, reduced intestinal inflammation, and decreased intestinal permeability (Figure E b–e). Flow cytometry analysis revealed increased IEC percentages and numbers, which were associated with reduced cell death and enhanced proliferation (Figure E f–i). Consistently, the expression levels of *ZO-1*, *Claudin-1*, and *Occludin* were upregulated following anti-IL-17A treatment (Figure E j). In contrast, anti-IL-22 treatment had no significant effect on weight change, intestinal inflammation, or intestinal permeability (Figure F a–e). Likewise, it did not alter IEC abundance, cell death, or proliferation, or tight junction protein expression (Figure F f–j).

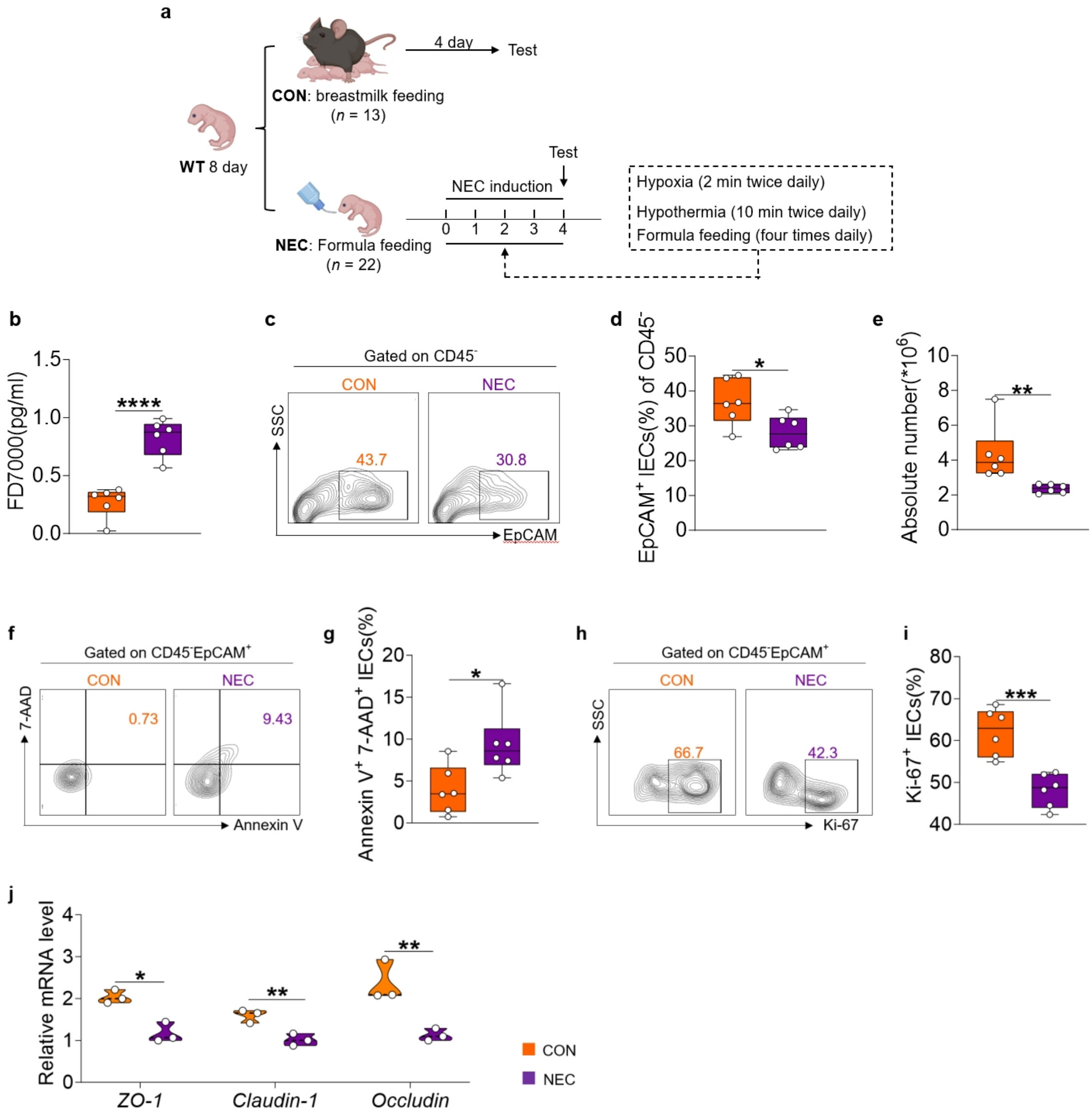

Figure D. NEC induction leads to intestinal barrier dysfunction in neonatal mice. (**a**) Experimental schema of NEC induction in wild-type pups. (**b**) Intestinal permeability was assessed by plasma FD7000 levels ($n = 6$). (**c–e**) Representative flow cytometry profiles, percentages, and absolute number of intestinal epithelial cells (IECs) ($n = 6$). (**f**, **g**) Representative flow cytometry profiles and statistical analysis of Annexin V$^+$ 7-AAD$^+$ IECs ($n = 6$). (**h**, **i**) Representative flow cytometry profiles and statistical analysis of Ki67$^+$ IECs ($n = 6$). (**j**) Relative mRNA level of tight junction proteins ($n = 3$ biological replicates). Each symbol represents one pup from a litter, and data are from at least three independent experiments. Error bars represent the SEM. *$p < 0.05$; **$p < 0.01$; ***$p < 0.001$; ns, not significant. Statistical significance was analyzed by Student's t test (**b–e**, **g**, **i**, and **j**).

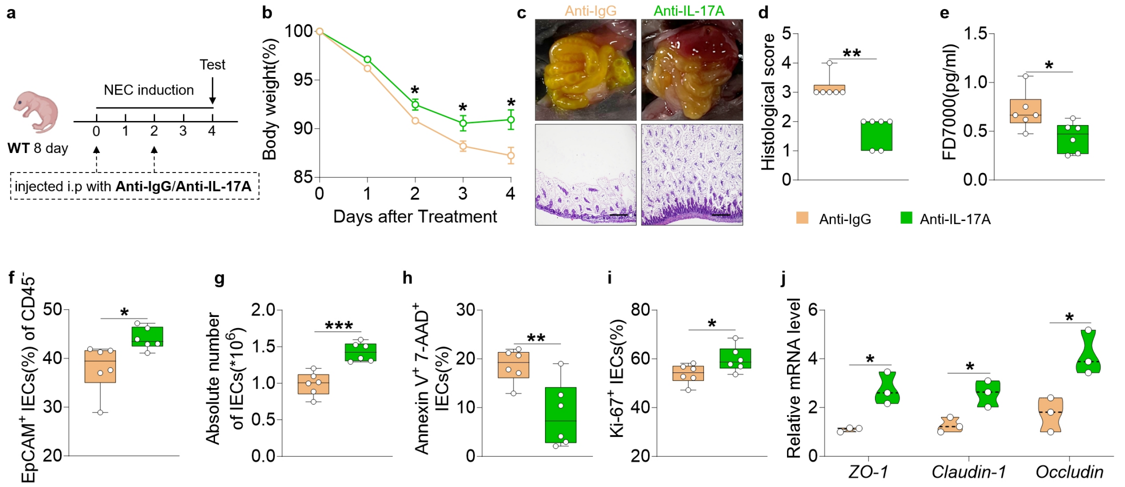

Figure E. Anti-IL-17A treatment significantly alleviates NEC severity and repairs intestinal barrier injury. (**a**) Experimental schema of neutralizing anti-IL-17A antibody during NEC induction. (**b**) Body weight changes in the indicated groups (*n* = 6). (**c**) Macroscopic image and H&E staining of the intestines (scale bar: 100 μm). (**d**) Inflammation scores were calculated (*n* = 6). (**e**) Intestinal permeability was assessed by plasma FD7000 levels (*n* = 6). (**f, g**) Percentage and absolute number of IECs (*n* = 6). (**h**) Statistical analysis of Annexin V^+ 7-AAD^+ IECs (*n* = 6). (**i**) Statistical analysis of $Ki67^+$ IECs (*n* = 6). (**j**) Relative mRNA levels of of *ZO-1*, *Claudin-1*, and *Occludin* (*n* = 3 biological replicates). Each symbol represents one pup from a litter, and data are from at least three independent experiments. Error bars represent the SEM. **p* < 0.05; ***p* < 0.01; ****p* < 0.001; ns, not significant. Statistical significance was analyzed by Student's *t* test (**b** and **d–j**).

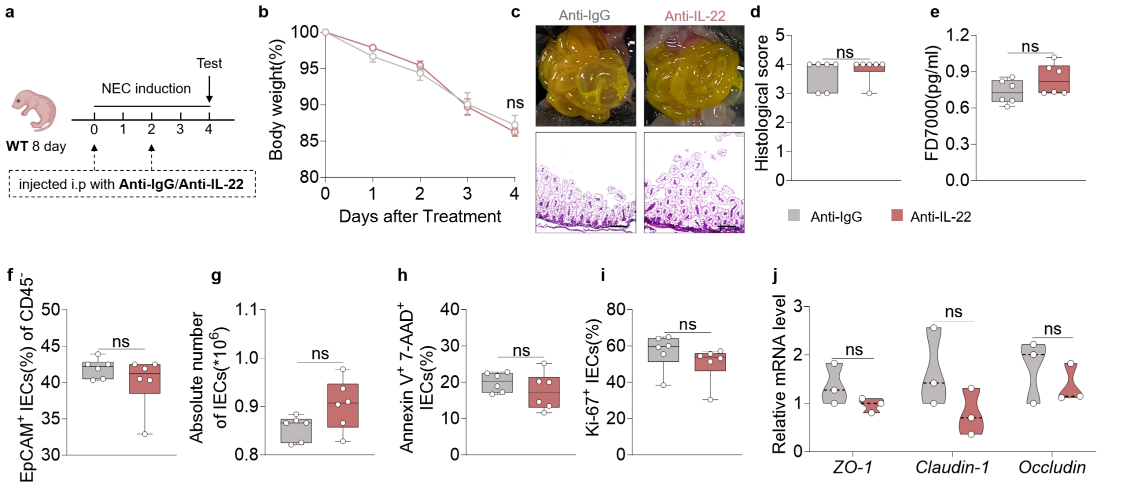

Figure F. Anti-IL-22 treatment has no effects on NEC alleviation and intestinal barrier repair. (**a**) Experimental schema of neutralizing anti-IL-22 antibody during NEC induction. (**b**) Body weight changes in the indicated groups (*n* = 6). (**c**) Macroscopic image and H&E staining of the intestines (scale bar: 100 μm). (**d**) Inflammation scores were calculated (*n* = 6). (**e**) Intestinal permeability was assessed by plasma FD7000 levels (*n* = 6). (**f, g**) Percentage and absolute number of IECs (*n* = 6). (**h**) Statistical analysis of Annexin V^+ 7-AAD^+ IECs (*n* = 6). (**i**) Statistical analysis of $Ki67^+$ IECs (*n* = 6). (**j**) Relative mRNA levels of of *ZO-1*, *Claudin-1*, and *Occludin* (*n* = 3 biological

replicates). Each symbol represents one pup from a litter, and data are from at least three independent experiments. Error bars represent the SEM. $*p < 0.05$; $**p < 0.01$; $***p < 0.001$; ns, not significant. Statistical significance was analyzed by Student's *t* test (**b** and **d**–**j**).

Collectively, these data indicate that IL-17A, rather than IL-22, plays a critical role in intestinal barrier dysfunction and NEC progression.

The supplementary data and corresponding description have been incorporated into the revised version (Figure 2j–q; Supplementary Figure 1d–k; Supplementary Figure 13a–h), (Page 6, Lines 119–128; Page 11, Lines 228–236).

3. Lines 145–148: The authors assert that T cells play a minimal role in NEC development and that DN ILC3s are the primary drivers. However, in the discussion, several cited studies indicate that T cells can significantly contribute to NEC pathogenesis. The authors should address this apparent discrepancy and consider softening their claims regarding the exclusivity of ILC3 involvement.

Reply: We sincerely appreciate your insightful comments regarding the roles of T cells in NEC pathogenesis. To address this issue, $Rag2^{-/-}$ mice were used, and adoptive transfer experiments were performed to further clarify the roles of T cells. The corresponding results are presented below.

- Firstly, NEC induction in both $Rag2^{-/-}$ and wild-type littermates resulted in comparable disease severity (Figure G a–d), with no significant differences in the frequencies or absolute numbers of total ILC3s, DN ILC3s, $CCR6^{+}$ ILC3s, $NKp46^{+}$ ILC3s, or IL-$17A^{+}$ and IL-22^{+} ILC3 subsets (Figure G e–j). This finding contrasts with a previous report showing that $Rag1^{-/-}$ mice were resistant to NEC induction, pointing to potential functional differences between the two models. Re-examination of that study (*J Clin Invest*, 2016, 10.1172/JCI83356) revealed that, while a similar induction protocol was used, the key methodological distinction was the addition of a bacterial product cocktail derived from NEC patient intestinal contents. The lack of critical details—including donor patient severity, and the composition and dosage of the bacterial products—precludes direct cross-study comparison. Consistent with prior reports, we found that $CD4^{-}ROR\gamma^{+}$ cells (predominantly DN ILC3s) were more than 8-fold more abundant than $CD4^{+}ROR\gamma^{+}$ T cells in neonatal NEC. This marked numerical dominance, together with the unabated susceptibility of $Rag2^{-/-}$ mice to NEC, supports a predominant role for DN ILC3s in driving NEC pathogenesis in our

model. The divergent outcomes between $Rag1^{-/-}$ and $Rag2^{-/-}$ models underscore the importance of model system considerations when interpreting immune contributions to this disease.

- To further clarify the functional roles of T cells and DN ILC3s, adoptive transfer experiments were performed in $Rag2^{-/-}$ neonates. Specifically, $Rag2^{-/-}$ recipients were intraperitoneally injected with PBS, 3×10^4 DN ILC3s, 3×10^4 $CD4^+$ T cells, or 3×10^6 $CD4^+$ T cells (Figure H a). Notably, transfer of DN ILC3s significantly exacerbated weight loss and intestinal inflammation, whereas $CD4^+$ T cells achieved similar effects when transferred at a 100-fold higher dose (Figure H b–d). This dose dependency may reflect opposing roles of $CD4^+$ T cell subsets in NEC pathogenesis, as previously reported (*J. Clin. Invest.*, 2016, 10.1172/JCI83356), that the NEC environment suppresses regulatory T cells while expanding pathogenic Th17 cells, suggesting a functional equilibrium. At low cell numbers, these opposing forces may counterbalance, yielding no net effect; at high numbers, however, the equilibrium may shift toward pathogenicity. These findings establish a functional hierarchy: while T cells can contribute at high numbers, DN ILC3s serve as key early drivers of NEC pathogenesis.

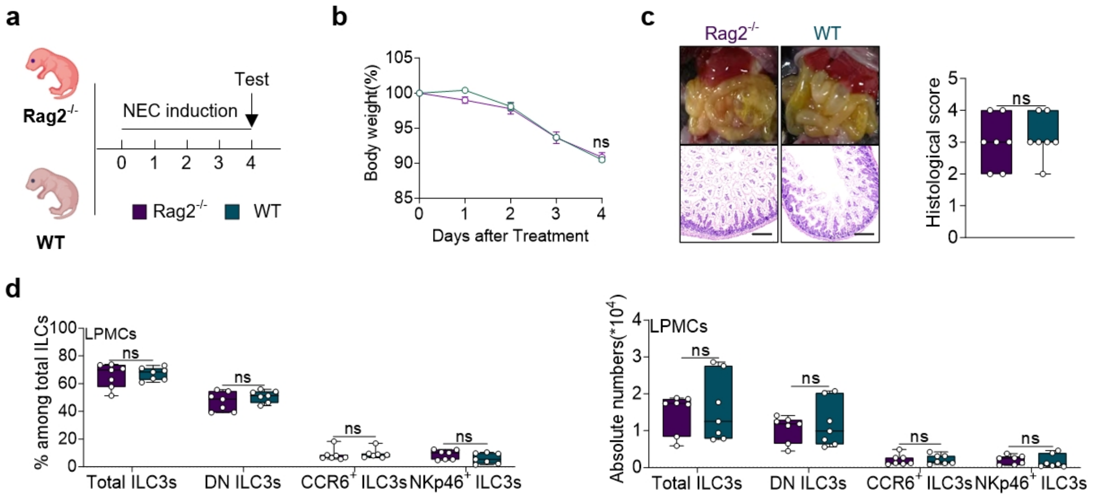

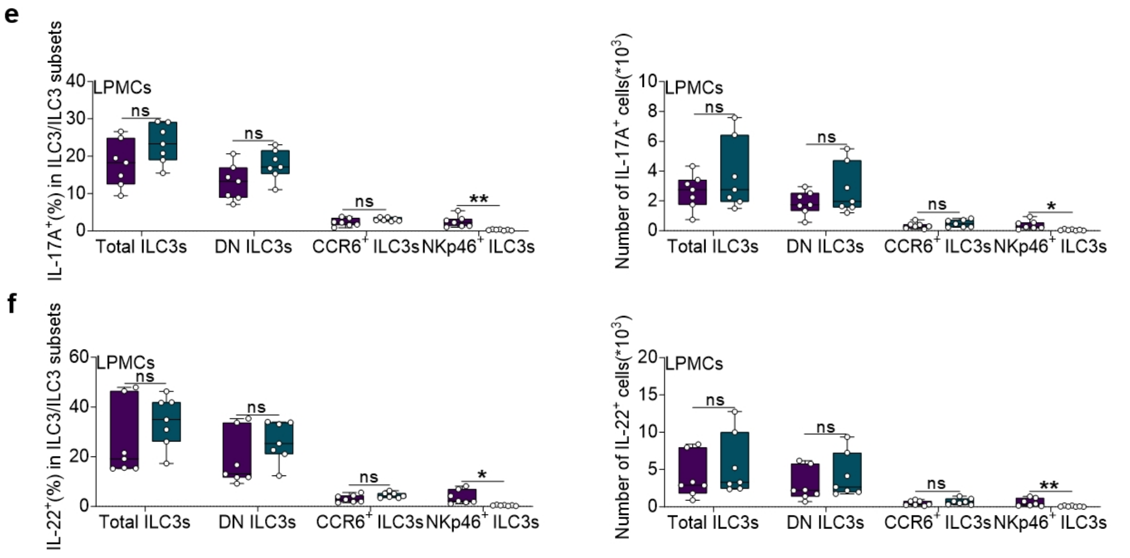

Figure G. Adaptive immune deficiency does not alter disease outcomes following NEC induction. (**a**) Experimental NEC was induced in Rag2$^{-/-}$ and wild-type neonates. (**b**) Body weight changes in the indicated groups (n = 7). (**c**) Macroscopic image and H&E staining of the intestines (scale bar: 100 μm).Inflammation scores were calculated (n = 7). (**d**) Percentage and absolute number of intestinal ILC3s and ILC3 subsets (n = 7). (**e**) Percentage and absolute number of IL17A^{+} ILC3s and ILC3 subsets (n = 7). Percentage and absolute number of IL-22^{+} ILC3s and ILC3 subsets (n = 7). Each symbol represents one pup from a litter, and data are from at least three independent experiments. Error bars represent the SEM. *$p < 0.05$; **$p < 0.01$; ***$p < 0.001$; ns, not significant. Statistical significance was analyzed by Student's t test (**b**–**f**).

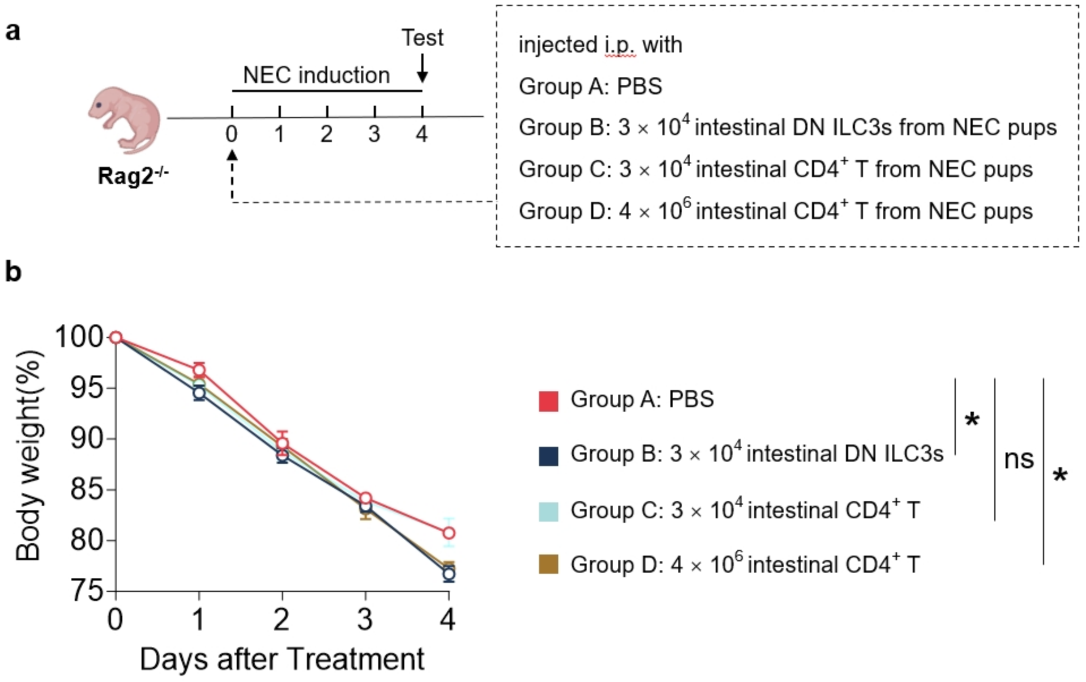

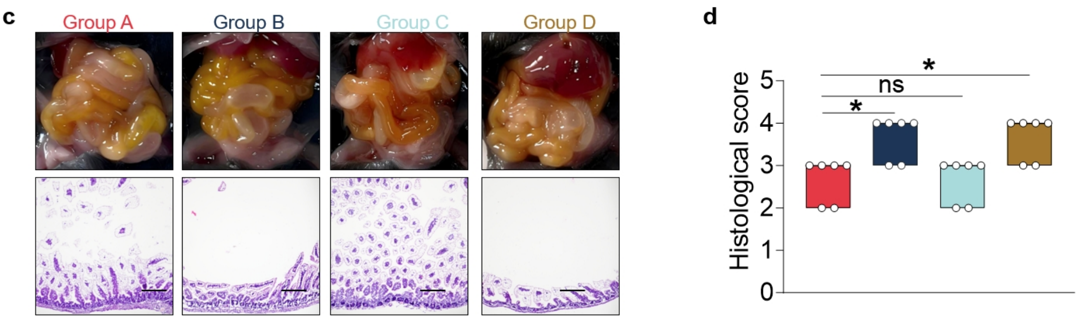

Figure H. Adoptive transfer of DN ILC3s or high number of CD4^{+} T cells exacerbates NEC in Rag2$^{-/-}$ neonates. (**a**) Schematic diagram of adoptive cell transfer experiments in Rag2$^{-/-}$ neonates. (**b**) Body weight changes in the indicated groups (n = 6). (**c**) Macroscopic image and H&E staining of the intestines (scale bar: 100 μm). (**d**) Inflammation scores were calculated (n = 6). Each symbol represents one pup from a litter, and data are from at least three independent experiments. Error bars represent the SEM. *p < 0.05; **p < 0.01; ***p < 0.001; ns, not significant. Statistical significance was analyzed by One-way ANOVA (**b** and **d**).

Collectively, these findings support our conclusion that intestinal DN ILC3s play a critical role in NEC. While T cells contribute to disease progression under certain conditions, their requirement at a markedly higher number suggests that DN ILC3s are the primary drivers in this model.

(3) Based on these findings, we have revised the Discussion correspondingly. The specific revisions are presented below:

Previous studies have shown that the NEC environment suppresses Tregs while expanding pathogenic Th17 cells, suggesting a functional equilibrium between these subsets (*J. Clin. Invest.*, 2016, 10.1172/JCI83356). At low transfer numbers, these opposing forces may counterbalance, resulting in no net effect. However, at high transfer numbers, this equilibrium may shift, allowing pathogenic subsets to predominate and promote disease progression. These findings delineate a functional hierarchy in NEC pathogenesis: while T cells can contribute when present in sufficient numbers, DN ILC3s appear to act as key early drivers of disease, consistent with their marked abundance in neonatal NEC. Elucidating the distinct contributions of these two populations is essential for developing subset-specific therapeutic interventions.

The supplementary data and corresponding description have been incorporated into the revised version (Supplementary Figure 5a–f; Supplementary Figure 10a–c), (Page 7-8 Lines 156–161; Page 10, Lines 208–213; Page 23-24, Lines 520–528).

4. In the discussion, the authors cite "purity" as an experimental limitation, but it is unclear what is meant by this. The manuscript would benefit from a clear presentation of the gating strategies used to quantify ILCs. While the statement in line 319 that CD4—cells do not exclusively represent ILC3s is accurate, additional strategies to exclude T cells- such as gating out TCRα/β or CD3/CD5 positive cells—should be considered and discussed.

Reply: We thank our reviewer for the constructive suggestion. We fully agree that a clear and detailed presentation of our gating strategies and purification efficiency would enhance the transparency and rigor of our study. In this study, ILC3s were isolated using two complementary approaches: fluorescence-activated cell sorting (FACS) and magnetic bead-based cell sorting. The purification efficiency of these methods is presented below.

- FACS strategy for distinct ILC3 subsets:

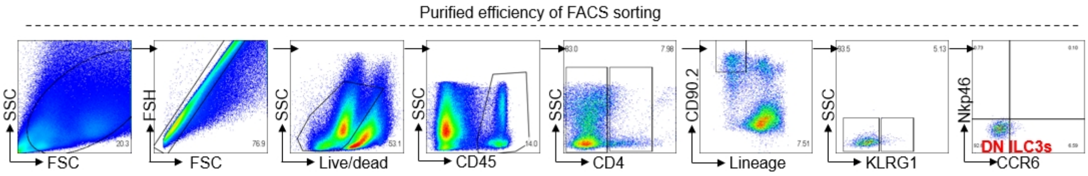

- Magnetic bead-based sorting for ILC3s. This method includes a depletion step to remove T cells ($CD3^+$, $CD5^+$, $TCR\alpha\beta^+$).

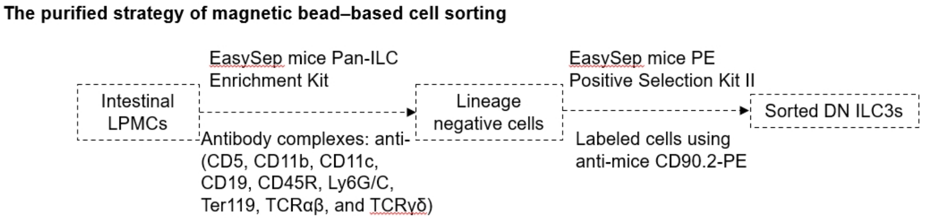

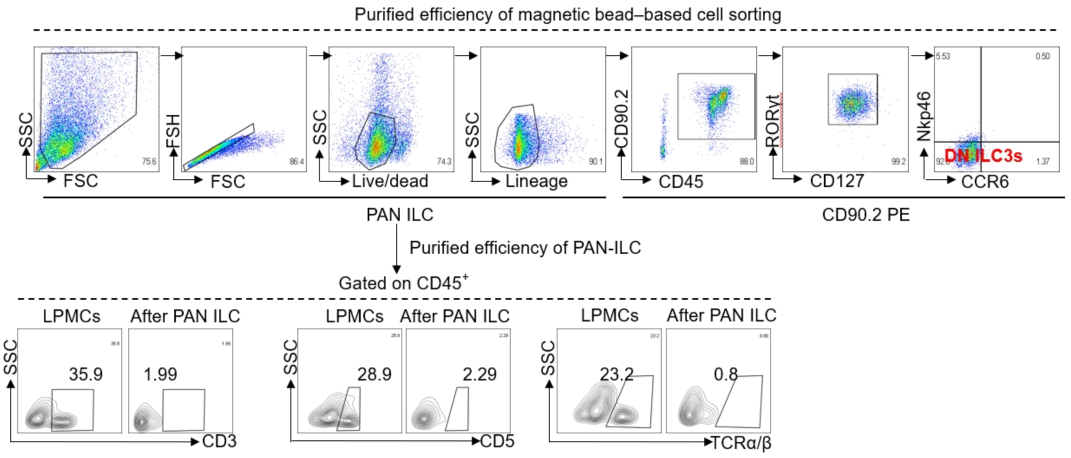

(2) Moreover, the gating strategies for ILC3s and detailed procedures for cell sorting have been revised in the Method Section. The specific revisions are presented below:

For mouse cell isolation, FACS was performed using a CytoFLEX SRT flow cytometer (Beckman Coulter, USA) to isolate target cell populations for subsequent experiments, including single-cell sequencing, RNA sequencing, ATAC-seq, RT-qPCR, *in vitro* culture, untargeted lipidomics, and adoptive transfer. Cell populations were defined as follows: DN ILC3s, $CD45^{+}CD4^{-}CD90.2^{hi}Lin^{-}KLRG1^{-}CCR6^{-}NKp46^{-}$; $NKp46^{+}$ ILC3s, $CD45^{+}CD4^{-}CD90.2^{hi}Lin^{-}KLRG1^{-}CCR6^{-}NKp46^{+}$; $CCR6^{+}$ ILC3s, $CD45^{+}CD4^{-}CD90.2^{hi}Lin^{-}KLRG1^{-}CCR6^{+}NKp46^{-}$; ILC2s, $CD45^{+}CD4^{-}CD90.2^{+}Lin^{-}$ $KLRG1^{+}$; and $CD4^{+}$ T cells, $CD45^{+}CD4^{+}$.

For immunomagnetic isolation of DN ILC3s, the EasySep Mouse Pan-ILC Enrichment Kit and EasySep Mouse PE Positive Selection Kit II (StemCell, Canada) were used according to the manufacturer's instructions. In brief, lineage-negative cells were first enriched, then labeled with anti-mouse CD90.2-PE antibody and further purified to obtain DN ILC3s. After sorting, cell purity was verified by flow cytometry. Purified cells were used for western blotting and transmission electron microscopy. The lineage markers used for mouse cell sorting included CD3, B220, CD11b, Ly6G, Ter119, CD11c, CD5, CD8a, TCRαβ, TCRγδ, and NK1.1.

The sorting strategies, the purity data, the validation are provided in the revised version (Supplementary Figure 36a, b), (Page 31, Lines 687–705).

5. The single-cell data presented in Figure 1 could be further leveraged to explore ILC3 biology. Analysis of the metabolic profiles of these cells would strengthen the authors' claims and provide additional mechanistic insight.

Reply: We deeply appreciate your insightful comments. In response, we have re-analyzed our scRNA-seq data focusing on the metabolic profiles of DN ILC3s and validated these metabolic alterations. The corresponding results are presented below.

- Gene Set Enrichment Analysis of scRNA-seq data revealed enhanced glycolysis and concomitant reduction in tricarboxylic acid (TCA) cycle and oxidative phosphorylation (OXPHOS) activity in DN ILC3s from NEC neonates (Figure I a–c). Consistently, the heatmap showed that glycolysis-related genes (e.g., *Hk1*, *Hk2*, *Pfkp*, *Slc16a1*) were upregulated, whereas genes involved in the TCA cycle

(e.g., *Pdk1*, *Slc25a4*, *Aco2*, *Idh1*, *Sdhc*) and OXPHOS (e.g., *Mdh1*, *Ndufv2*, *Cox6b1*, *Uqcrc1*) were downregulated (Figure I d).

- These transcriptional alterations were further confirmed at both the mRNA and protein levels. Compared to the controls, NEC DN ILC3s exhibited increased expression of glycolysis-related genes and decreased expression of TCA cycle- and OXPHOS-related genes (Figure I e). Moreover, flow cytometry analysis showed that GLUT1 and GLUT3 expression levels were elevated in NEC DN ILC3s, accompanied by increased intracellular lactate levels (Figure I f–h).

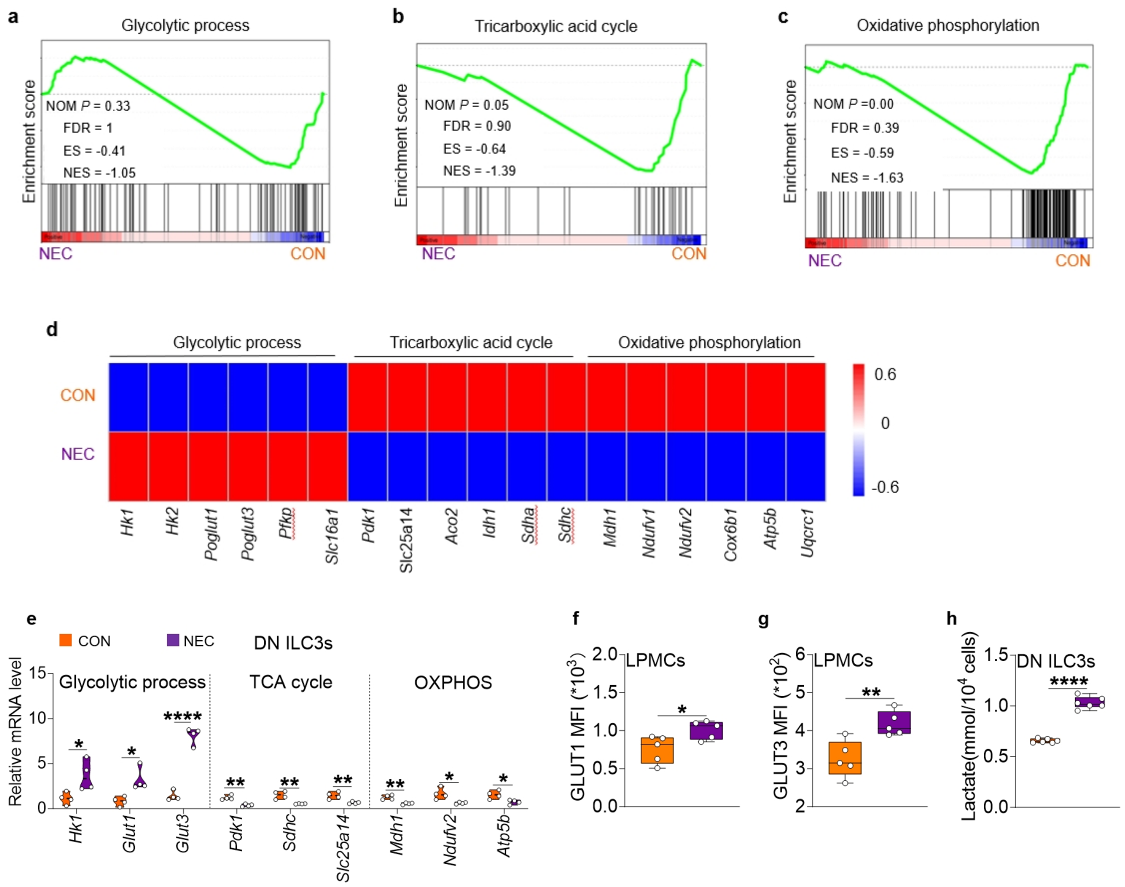

Figure I. Glycolysis is enhanced and TCA cycle/OXPHOS is inhibited in DN ILC3s during NEC induction. (**a**–**c**) Gene Set Enrichment Analysis plot demonstrating the enrichment in glycolytic process, tricarboxylic acid (TCA) cycle, and oxidative phosphorylation (OXPHOS) of DN ILC3s from CON and NEC neonatal mice. (**d**) Heatmap showing the expression profiles of glycolysis, TCA cycle, and OXPHOS. (**e**) Relative mRNA levels of the indicated genes in DN ILC3s (n = 4 biological replicates). (**f**, **g**) The protein levels of GLUT1 and GLUT3 in DN ILC3s (n = 5). (**h**) Statistical analysis of lactate levels in DN ILC3s (n = 6 biological replicates). Each symbol represents one pup from a litter, and data are from at least three independent experiments. Error bars represent the SEM. *$p < 0.05$; **p < 0.01; ***p < 0.001; ns, not significant. Statistical

significance was analyzed by Student's *t* test (**e**–**h**).

These data demonstrate that NEC DN ILC3s exhibit a distinct metabolic profile, showing enhanced glycolysis and reduced TCA cycle/OXPHOS activity.

The supplementary data and corresponding description have been incorporated into the revised version (Supplementary Figure 21a–h), (Page 15–16, Lines 334–349).

6. In figure 2, the authors report that transfer of DN ILC3s from control mice induces significant weight loss in recipient mice compared to PBS-treated controls. The manuscript does not provide a clear explanation for this phenotype. The authors should discuss potential mechanisms underlying this effect and, if possible, provide supporting data to clarify how DN ILC3 transfer contributes to weight loss.

Reply: We sincerely appreciate your insightful observations and apologize for the ambiguity in the previous submission. To address your concerns, we have reconstructed our description and performed additional experiments as follows:

- We wish to clarify the results of the adoptive transfer experiment in Figure 2. The original description stated that "Mice receiving physiologically derived DN ILC3s showed less weight loss and significantly lower intestinal inflammation scores than the PBS group". This phrasing could be misleading. In fact, adoptive transfer of physiological DN ILC3s alleviated weight loss and reduced intestinal inflammation compared to PBS controls. To further support these conclusions, we assessed intestinal barrier function in this transfer system (Figure J a), with the results presented below.
- Flow cytometry analysis revealed that the abundance and absolute number of IECs were significantly increased in neonates receiving CON DN ILC3s (Figure J b, c). Consistently, IEC viability and proliferation were elevated in this group (Figure J d, e). Moreover, the expression levels of tight junction proteins were upregulated following CON DN ILC3 transfer (Figure J f). Notably, all these disease parameters were reduced in neonates receiving NEC DN ILC3s (Figure J b–f). These data indicate that pathogenic DN ILC3s promote intestinal barrier dysfunction and IEC death.
- To explore how these DN ILC3s influence barrier integrity, we re-analyzed our scRNA-seq data. NEC DN ILC3s exhibited a distinct cytokine profile, characterized by significant upregulation of *Il17a* (Figure K a–c).
- Based on these findings, we further verified the role of IL-17A in NEC

progression. Anti-IL-17A or IgG control was administered intraperitoneally to wild-type pups during NEC induction (Figure L a). Notably, anti-IL-17A treatment significantly reduced weight loss, intestinal inflammation, and intestinal permeability (Figure L b–e). Flow cytometry analysis showed increased IEC percentages and numbers, which were associated with reduced cell death and enhanced proliferation (Figure L f–i). Additionally, the expression levels of *ZO-1*, *Claudin-1*, and *Occludin* were upregulated following anti-IL-17A treatment (Figure L j).

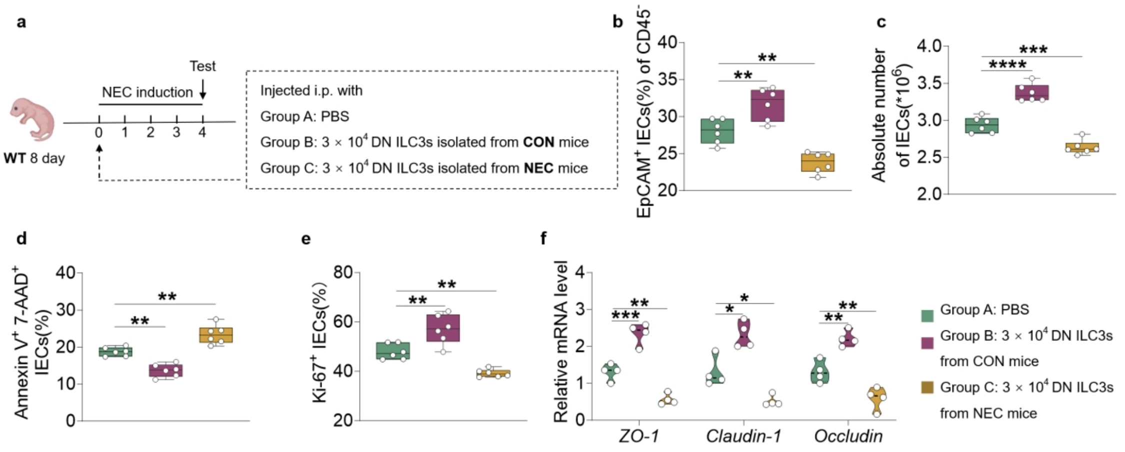

Figure J. Pathogenic DN ILC3s promote intestinal barrier dysfunction and IEC death. (**a**) Experimental schema of adoptive transfer during NEC induction. (**b**, **c**) Percentage and absolute number of IECs in $CD45^-$ cells ($n = 6$). (**d**) Statistical analysis of Annexin V^+ 7-AAD^+ IECs ($n = 6$). (**e**) Statistical analysis of $Ki67^+$ IECs ($n = 6$). (**f**) Relative mRNA levels of *ZO-1*, *Claudin-1*, and *Occludin* in the intestines ($n = 4$ biological replicates). Each symbol represents one pup from a litter, and data are from at least three independent experiments. Error bars represent the SEM. $^*p < 0.05$; $^{**}p < 0.01$; $^{***}p < 0.001$; ns, not significant. Statistical significance was analyzed by One-way ANOVA (**b**–**f**).

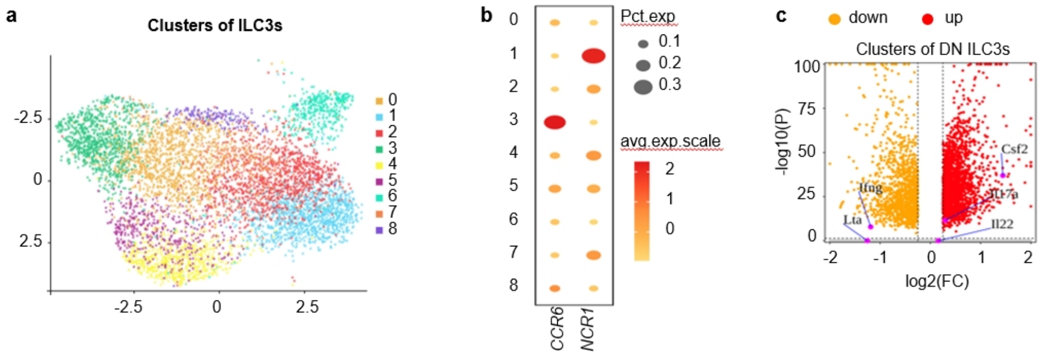

Figure K. Single-cell transcriptomic profiling reveals distinct functional states of ILC3 subsets under NEC conditions. (**a**) Uniform manifold approximation and projection (UMAP) visualization

of ILC3 clusters. (**b**) The expression levels of *CCR6* and *NCR1* across ILC3 clusters. (**c**) Volcano plots showing differentially expressed genes in DN ILC3s.

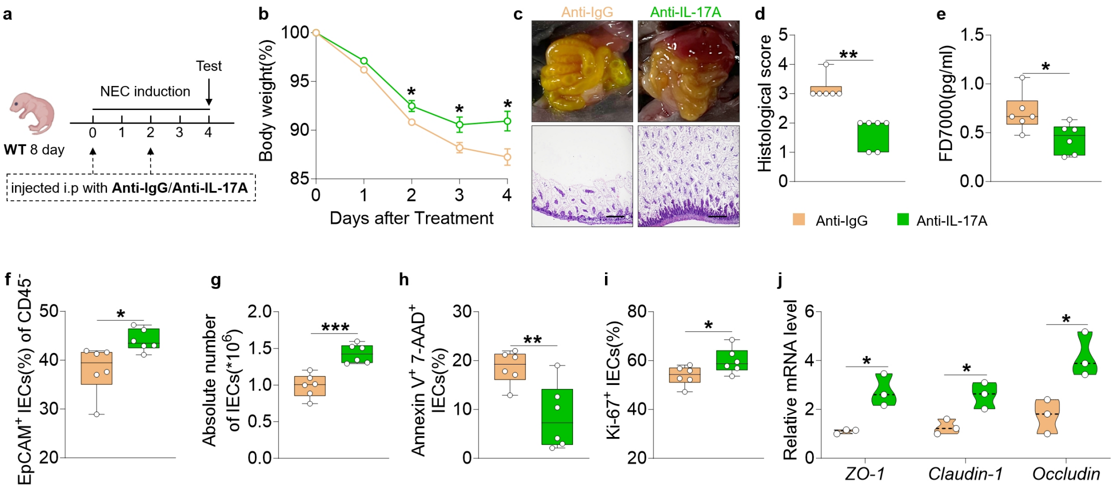

Figure L. Anti-IL-17A treatment significantly alleviates NEC severity and repairs intestinal barrier injury. (**a**) Experimental schema of neutralizing anti-IL-17A antibody during NEC induction. (**b**) Body weight changes in the indicated groups (n = 6). (**c**) Macroscopic image and H&E staining of the intestines (scale bar: 100 μm). (**d**) Inflammation scores were calculated (n = 6). (**e**) Intestinal permeability was assessed by plasma FD7000 levels (n = 6). (**f**, **g**) Percentage and absolute number of IECs (n = 6). (**h**) Statistical analysis of Annexin V^+ 7-AAD^+ IECs (n = 6). (**i**) Statistical analysis of $Ki67^+$ IECs (n = 6). (**j**) Relative mRNA levels of of *ZO-1*, *Claudin-1*, and *Occludin* (n = 3 biological replicates). Each symbol represents one pup from a litter, and data are from at least three independent experiments. Error bars represent the SEM. *$p < 0.05$; **$p < 0.01$; ***$p < 0.001$; ns, not significant. Statistical significance was analyzed by Student's t test (**b** and **d**–**j**).

Taken together, these data demonstrate that pathogenic DN ILC3s drive IL-17A production, leading to a cascade of barrier dysfunction. Transfer of these cells induces intestinal barrier disruption and IEC damage, thereby accounting for the weight loss observed in the adoptive transfer model.

The supplementary data and corresponding description have been incorporated into the revised version (Figure 2a–h, j–q; Supplementary Figure 11a, b), (Page 9, Lines 186–197; Page 10, Lines 215–221; Page 11, Lines 228–232).

7. In Figure 8, the manuscript would benefit from a deeper characterization of the ILC3 compartment. Examining ILC3 subclusters and determining how the double-negative population changes in NEC patients could provide valuable context. Additionally, presenting the expression of effector cytokine transcripts such as IL17 and IL22 in patient samples would help bridge the murine findings with the human setting and strengthen the overall translational impact of the study.

Reply: We are grateful for your valuable suggestion. As per your recommendation, we have re-clustered the ILC3 cluster and expanded our clinical analysis to determine the alterations of cytokines and autophagy activity in human samples. The corresponding results are presented below.

Based on the expression of signature gene annotations, we identified an ILC3 cluster in the intestines (Figure M a, b). The proportion of this cluster was comparable between controls and infants with NEC (Figure M c). We therefore further regrouped this cluster using the subset-specific markers and identified three subclusters: CCR6$^+$ ILC3s, NCR$^+$ ILC3s, and DN ILC3s (Figure M d, e). Notably, DN ILC3s represented the predominant ILC3 subset and were significantly increased in the NEC group (Figure M f). Further analysis showed that the expression levels of *IL-17A* and *IL-22*, as well as *ATG5*, *ATG7*, and *ATG12*, were upregulated in ILC3s from NEC infants (Figure M g). Consistent with this transcriptional profile, qPCR results further confirmed the upregulation of cytokine- and autophagy-related genes in ILC3s from NEC infants (Figure M h). Moreover, IL-17A and IL-22 production was also increased in this ILC3 subset (Figure M i, j).

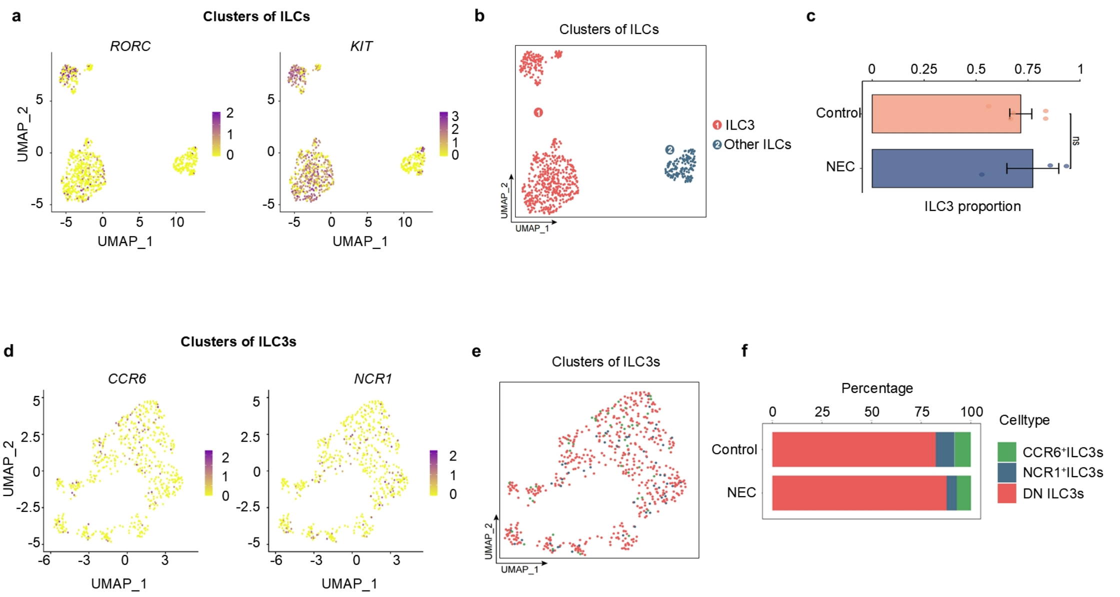

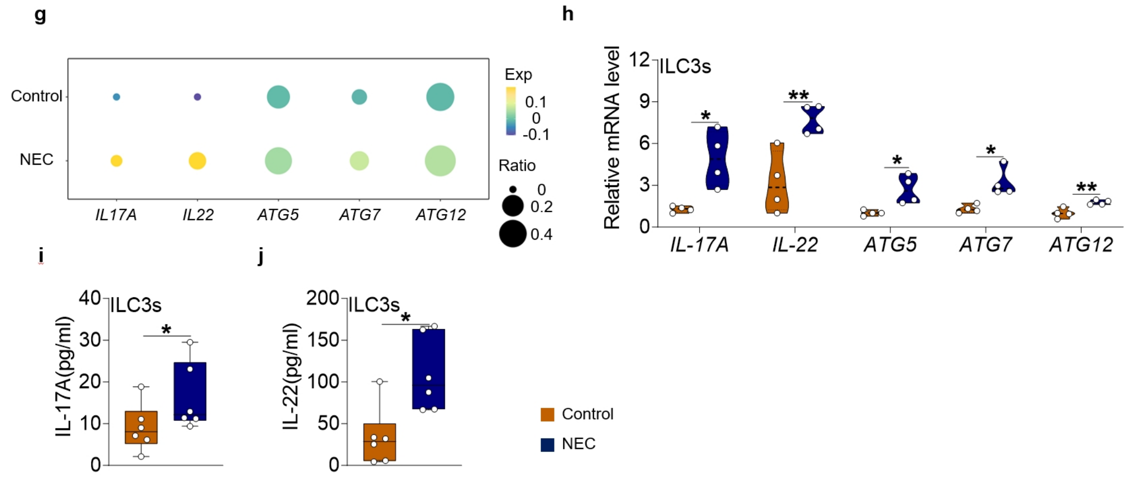

Figure M. Enhanced ILC3 function and autophagy in infants with NEC. (**a**) UMAP plots showing the expression of *RORC* an *KIT* in the ILC subsets. (**b**) UMAP visualization of the ILC clusters of controls and infants with NEC. (**c**) The proportion of ILC3s in controls and NEC infants. (**d**) UAMP plots showing the expression of *CCR6* and *NCR1* in the ILC3 subsets. (**e**) UAMP visualization of the ILC3 subclusters of controls and NEC infants. (**f**) The proportion of these subclusters in controls and NEC infants. (**g**) Average expression levels of *IL-17A*, *IL-22*, *ATG5*, *ATG7*, and *ATG12* in the ILC3 cluster. (**h**) Relative mRNA levels of *IL-17A*, *IL-22*, *ATG5*, *ATG7*, and *ATG12* in human ILC3s ($n = 4$ biological replicates). (**i**, **j**) Levels of IL-17A and IL-22 in human ILC3s ($n = 6$ biological replicates). Each symbol represents an individual neonate with NEC or healthy control. Error bars represent the SEM. *$p < 0.05$; **$p < 0.01$; ***$p < 0.001$; ns, not significant. Statistical significance was analyzed by Student's *t* test (**h–i**).

These data indicate that the function and autophagy activity are enhanced in DN ILC3s of infants with NEC, which aligns with the murine findings.

The supplementary data and corresponding description have been incorporated into the revised version (Figure 8a–g; Supplementary Figure 33a–c), (Page 20–21, Lines 452–464; Page 20, Lines 470–473).

Response to Reviewer #2:

We sincerely appreciate the time and effort you have dedicated to reviewing our manuscript. Your valuable feedback and suggestions have significantly improved the quality of our paper. In response, we have thoroughly revised the manuscript and incorporated the experimental data to strengthen the credibility of our findings. All modifications in the revised manuscript have been **indicated in blue text** for your convenience. Please refer to the detailed, point-by-point response to each comment below. We sincerely hope the revised version meets your expectations and look forward for your approval.

In this manuscript, He and colleagues sought to define how ILC3s drive immune inflammation that drives necrotizing enterocolitis (NEC), a serious ailment in preterm neonates. In summary, the authors found that DN ILC3s are the crucial subset driving this disease, through an increase in autophagy that drives ILC3 pro-inflammatory responses. More specifically, autophagy in DN ILC3s was found to induce glycolysis at the expense of lipid metabolism, and ATG5 or autophagy-deficient ILC3s revert this phenotype. To reach these conclusions, the authors used a combination of single cell transcriptomics analysis, in vivo models of NEC in T cell-deficient mice and specific interventions on distinct molecular pathways.

This is an interesting work that clearly indicates an important role for ILC3 autophagy in the promotion of NEC. However, there are important experiments and issues that need to be addressed in order to unequivocally prove some of the conclusions stated, especially the confirmation that DN ILC3s are, indeed, the major contributor for NEC, as well as additional exploration about how DN ILC3 autophagy leads to glycolysis-mediated cytokine production, which seems counterintuitive compared to the canonical function of autophagy. Specific points are listed below:

Reply: We wish to express our appreciation for your insightful comments.

Major:

1.The findings that autophagy induces glycolytic function in DN ILC3s are counterintuitive. In many cases, an inhibition of autophagy would be associated with an increase in aerobic glycolysis as a compensatory mechanism. Is the phenotype observed in Figure 5 uniquely found on DN ILC3 cells from neonates? How about DN ILC3 cells in other tissues or from adults?

Reply: We sincerely appreciate your insightful comment, which allows us to further clarify the regulatory role of autophagy in DN ILC3 glycolysis. To address your concerns, we have clarified the potential mechanisms and performed additional experiments to support these conclusions. The corresponding results are presented below.

(1) We fully acknowledge that autophagy inhibition is often coupled with a compensatory increase in aerobic glycolysis in multiple cell types (e.g., tumor cells, fibroblasts, terminally differentiated immune cells). However, emerging evidence has demonstrated that the regulation of autophagy on glycolysis is cell-type specific, context-dependent, and not a universal unidirectional mode.

- Hu et al. (*Adv. Sci.*, 2025, 10.1002/advs.202414673) revealed that in mice with hyperuricemic nephropathy, tubule-specific *Atg5* knockout leads to autophagy inhibition and glycolytic hyperactivation, thereby alleviating kidney fibrosis and improving renal function.
- Gong et al. (*Autophagy*, 2022, 10.1080/15548627.2021.1990515) showed that Newcastle disease virus infection induces mitochondrial fission, reduces membrane potential, and leads to abnormal respiratory chain in A549 cells; autophagy knockout blocks aberrant activation of glycolysis and reverses metabolic reprogramming.
- Khayati et al. (*Cancer Res*, 2022, 10.1158/0008-5472.CAN-22-1039) reported that in KrasG12D/+; p53–/– non-small cell lung cancer models, systemic autophagy knockout inhibits tumor glycolysis, promotes apoptosis, and suppresses tumor growth, while tumor-specific autophagy knockout has no such effect.

These studies highlight that the autophagy-glycolysis axis is not unidirectional but rather controlled by cellular and pathological context, supporting the specific regulatory axis observed in NEC DN ILC3s.

(2) To determine whether the autophagy-glycolysis axis in intestinal NEC DN ILC3s is tissue specific, we assessed DN ILC3s in the liver, lung, kidney, and spleen. Liver, kidney, and splenic NEC DN ILC3s from *Atg5*-deficient mice showed minimal changes in glucose dependence, mitochondrial dependence, glycolytic capacity, and fatty acid oxidation (FAO) and amino acid oxidation (AAO). In contrast, lung DN ILC3s exhibited reduced FAO and AAO capacity and compensatory increased glucose dependence upon *Atg5* deficiency (Figure A a–d).

(3) To assess the model dependency of our findings, we next employed a *C. rodentium* infection model using mice with conditional *Atg5* deletion. *C. rodentium* infection upregulated glycolysis-related genes in DN ILC3s, without altering genes involved in

fatty acid metabolism (Figure B a). However, this upregulation was unaffected by *Atg5* deficiency (Figure B a).

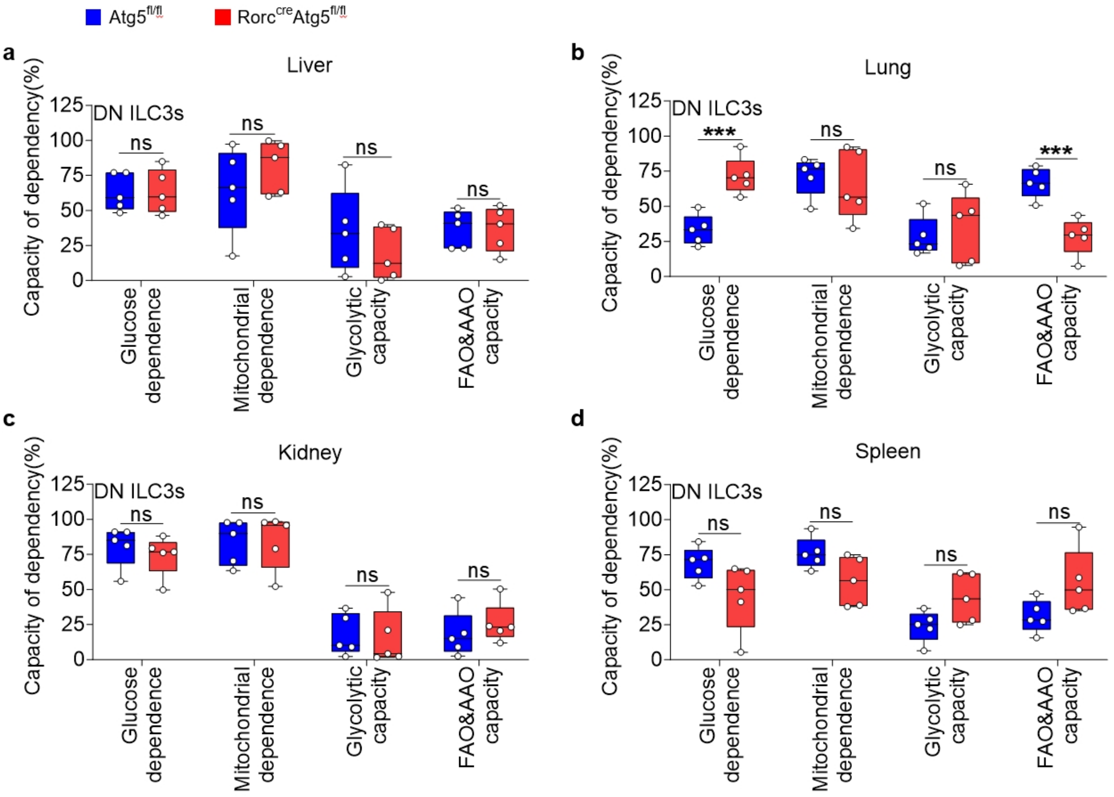

Figure A. The metabolic dependence profiles of DN ILC3s across distinct tissues. (**a**, **b**) Glucose dependence, mitochondrial dependence, glycolytic capacity, and FAO and AAO capacity in liver or lung DN ILC3s ($n = 5$). (**c**, **d**) Glucose dependence, mitochondrial dependence, glycolytic capacity, and FAO and AAO capacity in kidney or splenic DN ILC3s ($n = 5$). Each symbol represents one pup from a litter, and data are from at least three independent experiments. Error bars represent the SEM. $*p < 0.05$; $**p < 0.01$; $***p < 0.001$; ns, not significant. Statistical significance was analyzed by Student's *t* test (**a-d**).

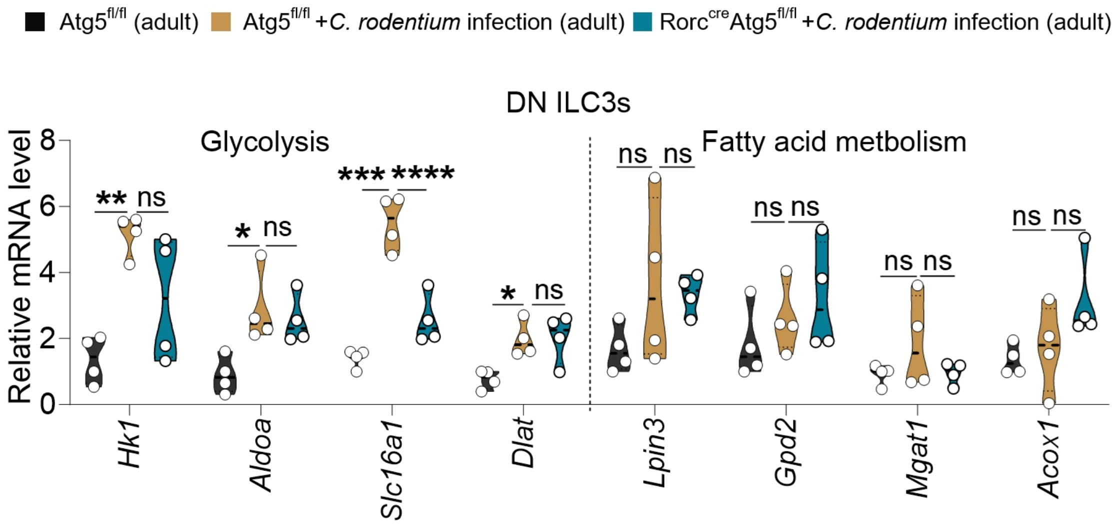

Figure B. *C. rodentium* infection impairs glycolysis in DN ILC3s. (**a**) Relative mRNA levels of glycolysis- and fatty acid metabolism-related genes in DN ILC3s ($n = 4$ biological replicates). Each symbol represents one pup from a litter, and data are from at least three independent experiments. Error bars represent the SEM. *$p < 0.05$; **$p < 0.01$; ***$p < 0.001$; ns, not significant. Statistical significance was analyzed by One-way ANOVA (**a**).

Collectively, these data demonstrate that the autophagy-glycolysis axis in pathogenic DN ILC3s is context-specific and tissue-restricted.

The supplementary data and corresponding description have been incorporated into the revised version (Supplementary Figure S23a; Supplementary Figure 25a), (Page 16–17, Lines 360–364; Page 17 Lines 374–379).

2. Mechanistically, how would autophagy inhibition mediate lipid absorption and inhibition of glycolysis? It would be important to further analyze these non-canonical connections. One example would be to investigate, in NEC DN ILC3 cells, whether transcription factors associated with glycolysis (e.g., Myc) are being preferentially degraded (in comparison to other cells). Another important assessment could be the tracking of the epigenetic state (using either ATACseq or CHIPseq associated with glycolysis-initiating TFs) of the genes associated with glycolysis versus fatty acid uptake.

Reply: We are grateful for your insightful suggestion. To directly address this issue, we have performed ATAC-seq and validated the binding activity of key transcription factors in the metabolic processes. The corresponding results are presented below.

- DN ILC3s were isolated from $Atg5^{fl/fl}$ and $Rorc^{cre}Atg5^{fl/fl}$ neonates under NEC conditions and subjected to ATAC-seq. The heatmap of chromatin accessibility around transcription start sites (TSSs) was reduced in *Atg5*-deficient DN ILC3s

(Figure C a). Analysis of key glycolysis-related TFs showed that HIF-1α, but not Mlxip and Myc, exhibited reduced chromatin accessibility in *Atg5*-deficient DN ILC3s (Figure C b–d).

- Consistent with reduced chromatin accessibility at HIF-1α-associated loci, the expression of HIF-1α was impaired in *Atg5*-deficient DN ILC3s, while MLXIP and MYC remained unaltered (Figure C e–g). Correspondingly, the binding of HIF-1α to the promoter regions of key glycolytic genes (*Hk1*, *Dlat*, and *Slc16a1*) was significantly reduced in these cells (Figure C h).

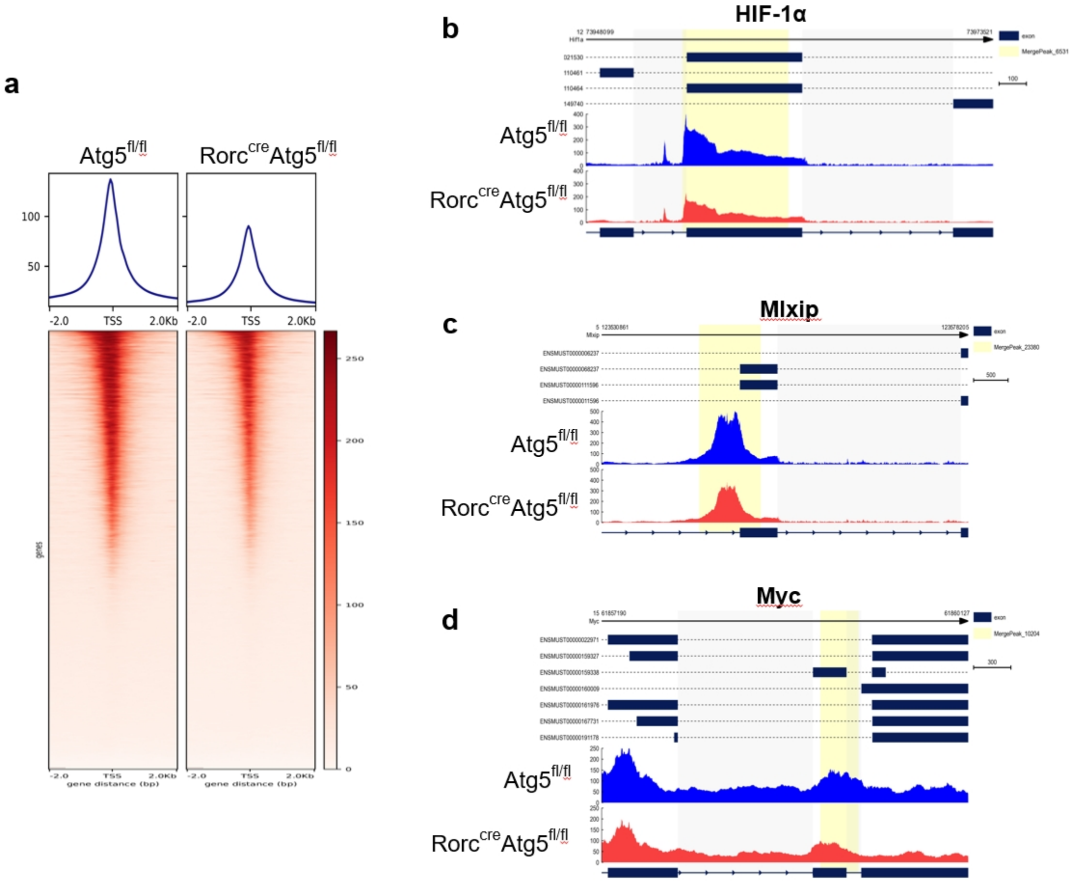

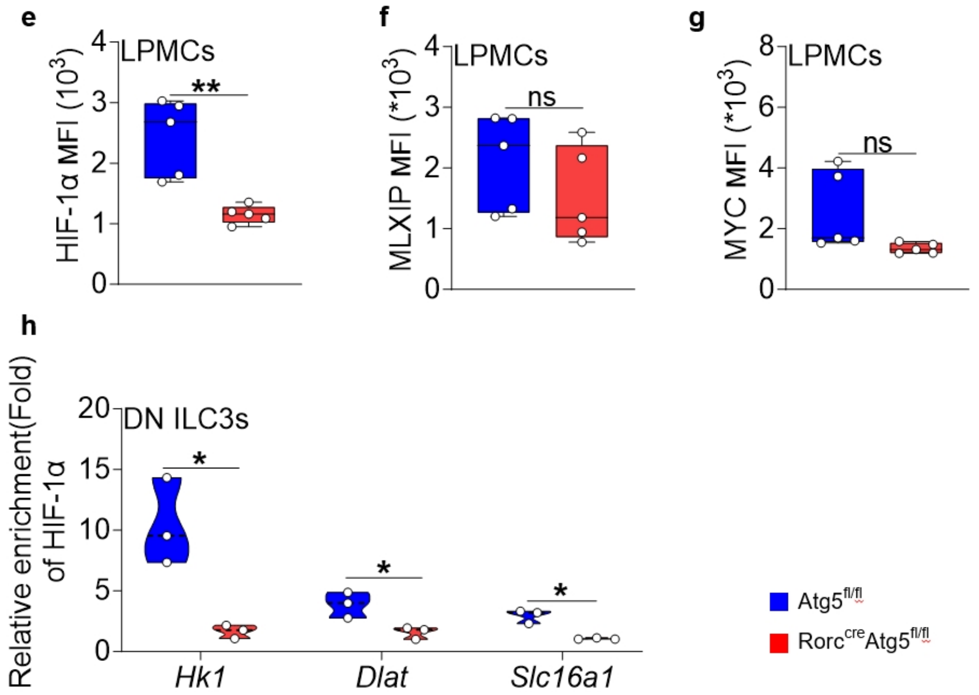

Figure C. *Atg5* deficiency reduces HIF-1α expression and transcriptional activity in DN ILC3s. (**a**) Heatmap showing chromatin accessibility around TSSs in DN ILC3s from Atg5$^{fl/fl}$ and Rorc^{cre}Atg5$^{fl/fl}$ mice. (**b–d**) Genome browser tracks showing chromatin accessibility at the HIF-1α, MLXIP, and MYC loci in DN ILC3s from Atg5$^{fl/fl}$ and Rorc^{cre}Atg5$^{fl/fl}$ mice. (**e–g**) The protein levels of HIF-1α, MLXIP, and MYC in DN ILC3s ($n = 5$). (**h**) Relative enrichment of HIF-1α binding to *Hk1*, *Dlat*, and *Slc16a1* genes ($n = 3$ biological replicates). Each symbol represents one pup from a litter, and data are from at least three independent experiments. Error bars represent the SEM. *$p < 0.05$; **$p < 0.01$; ***$p < 0.001$; ns, not significant. Statistical significance was analyzed by Student's *t* test (**e–h**).

These data indicate that *Atg5* deficiency in DN ILC3s leads to reduced chromatin accessibility and impaired HIF-1α binding at glycolysis-related gene loci, revealing that autophagy controls glycolysis in DN ILC3s during NEC.

The supplementary data and corresponding description have been incorporated into the revised version (Figure 5k–m; Supplementary Figure 24a–d), (Page 17, Lines 366–372).

3. The DN ILC3 transfer experiments into WT or Rag2KO mice are important to establish some causal relation, but there are some potential confounding effects due to the presence of ILCs in host mice – that may explain why the differences observed are somewhat small. It would be important to perform experiments where this transfer is done into ILC- or ILC3-deficient mice, such as Nfil3-KO or even into Rag2-KO x Rorc-KO.

Reply: We sincerely appreciate your insightful comment and fully agree with the potential confounding of endogenous ILCs in wild-type or Rag2$^{-/-}$ recipients. To address this concern, we have performed adoptive transfer experiments using *Atg5*-deficient DN ILC3s into Nfil3$^{-/-}$ mice. The corresponding results are presented below.

DN ILC3s isolated from Atg5$^{fl/fl}$ and Rorc^{cre}Atg5$^{fl/fl}$ littermates were intraperitoneally transferred into Nfil3$^{-/-}$ recipients prior to NEC induction (Figure D a). Mice receiving *Atg5*-deficient DN ILC3s exhibited reduced weight loss and intestinal inflammation compared to those receiving control DN ILC3s (Figure D b–d). Consistently, the proportion and absolute number of DN ILC3s were significantly reduced. However, no significant changes were observed in NKp46^{+} or CCR6^{+} ILC3s (Figure D e, f).

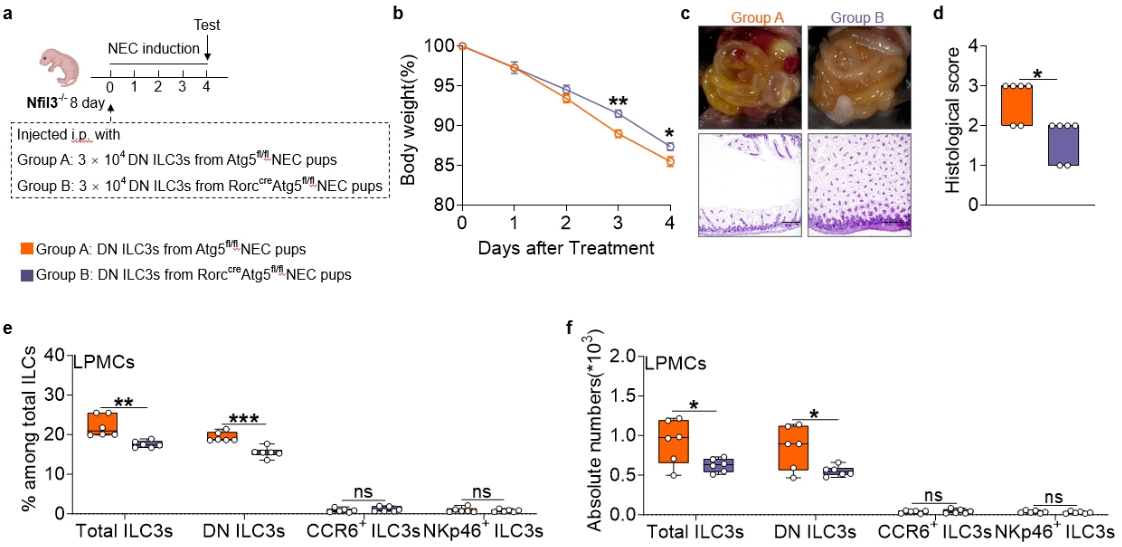

Figure D. Atg5 expression is required for DN ILC3 pathogenicity in NEC. (**a**) Experimental schema of adoptive transfer prior to NEC induction. (**b**) Body weight changes in the indicated groups ($n = 6$). (**c**) Macroscopic image and H&E staining of the intestines (scale bar: 100 μm). (**d**) Statistical analysis of inflammation scores ($n = 6$). (**e**, **f**) Proportion and absolute number of ILC3s, DN ILC3s, CCR6^{+} ILC3s, and NKp46^{+} ILC3s ($n = 6$). Each symbol represents one pup from a litter, and data are from at least three independent experiments. Error bars represent the SEM. *$p < 0.05$; **$p < 0.01$; ***$p < 0.001$; ns, not significant. Statistical significance was analyzed by Student's *t* test (**b** and **d–f**).

Through ILC-deficient Nfil3$^{-/-}$ mice, we confirm that ATG5 is a critical driver of DN ILC3 pathogenicity in NEC.

The supplementary data and corresponding description have been incorporated into the revised version (Figure 4j–n), (Page 14, Lines 309–313).

4. Autophagy (or at least Atg5) seems to be increased not only on DN ILC3s, but also on other ILC3 subsets. Are there any pathways specifically enriched in DN ILC3s? That would be worth checking, since the authors claim that DN ILC3s play an apparent dominant role in NEC.

Alternatively, is the autophagy pathway differentially regulating DN ILC3s, if compared to other ILC3 subsets? It would be interesting to evaluate if canonical downstream effects of autophagy are triggered differently between ILC3 subsets.

Reply: We sincerely appreciate your insightful comment. To address this issue, we have re-analyzed our scRNA-seq data, assessed autophagy-related signatures in the ILC3 subsets, and validated the specific regulation of autophagy in DN ILC3s. The corresponding results are presented below.

- Signal transduction pathway enrichment across three ILC3 subsets was analyzed, showing a distinct pattern in DN ILC3s. The mTOR signaling pathway—a central node that coordinates metabolic and autophagic processes—was specifically enriched in DN ILC3s (Figure E a-c). These observations were further confirmed by increased ATG5 and LAMP-1 expression, alongside reduced P62 levels, specifically in total ILC3s and DN ILC3s, but not in other subsets (Figure F a-c). These data demonstrate a specific regulation of autophagy in DN ILC3s.
- While ATG5 expression was elevated across all three ILC3 subsets during NEC, function-related autophagy regulation was restricted to DN ILC3s. 3-MA or rapamycin treatment reduced and enhanced the frequency and absolute number of total ILC3s and DN ILC3s in vivo, respectively (Figure G a, b). Consistently, IL-17A$^+$ and IL-22$^+$ DN ILC3s were significantly decreased following 3-MA treatment but increased after rapamycin treatment (Figure G c-f). Flow cytometry analysis confirmed a significant change in ATG5 expression within DN ILC3s (Figure G g). However, these interventions did not alter the proportion, function, or ATG5 expression in NKp46$^+$ or CCR6$^+$ ILC3s (Figure G a-g).
- To further validate this specificity, we employed Rorc^{cre}Atg5$^{fl/fl}$ mice (deleted Atg5 from RORγt-positive cells). Consistent with our pharmacological findings, *Atg5* deficiency significantly reduced proportions and numbers of total ILC3s and DN ILC3s (Figure H a, b). Additionally, *Atg5* deficiency impaired IL-17A and IL-22 expression in total ILC3s and DN ILC3s (Figure H c, d). However, no

significant changes were observed in the proportions or functions of NKp46$^+$ or CCR6$^+$ ILC3s (Figure H a-d).

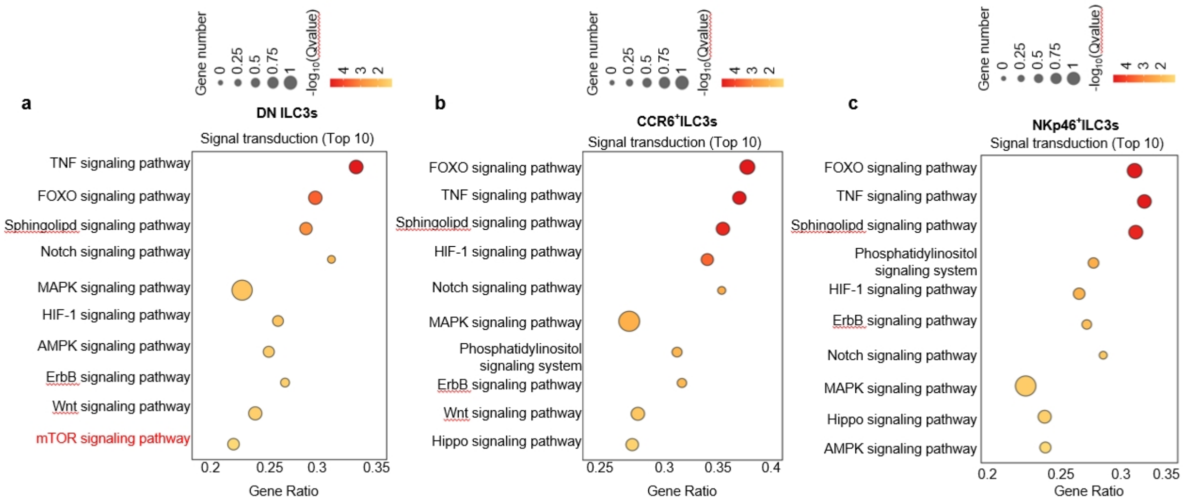

Figure E. Signal transduction enrichment in ILC3 subsets. (**a-c**) Dot plot displaying the top 10 enriched KEGG signal transduction pathways in DN ILC3s, CCR6$^+$ ILC3s, and NKp46$^+$ ILC3s.

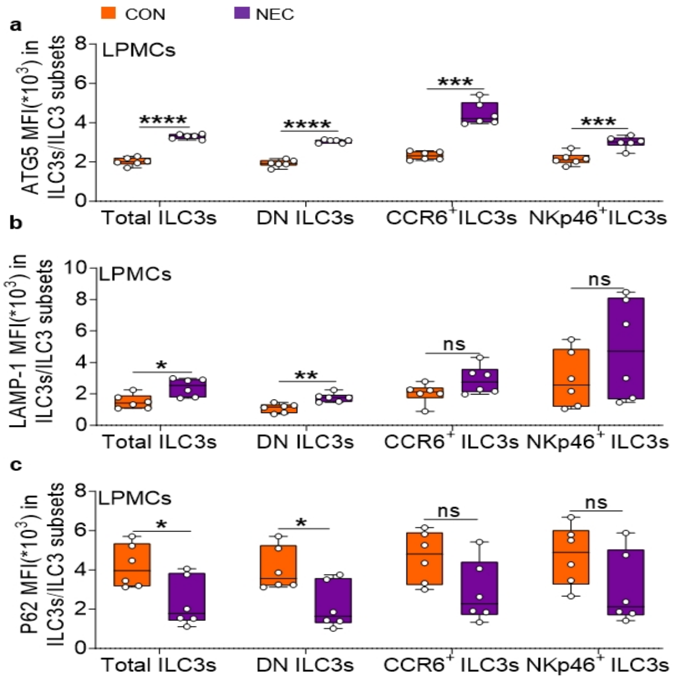

Figure F. Selective activation in DN ILC3 autophagy during NEC, shown by ATG5/LAMP-1 upregulation and P62 downregulation. (**a-c**) The protein levels of ATG5, LAMP-1, and P62 in intestinal ILC3s and ILC3 subsets ($n = 6$). Each symbol represents one pup from a litter, and data are from at least three independent experiments. Error bars represent the SEM. *$p < 0.05$; **$p < 0.01$; ***$p < 0.001$; ns, not significant. Statistical significance was analyzed by Student's t test (**a-c**).

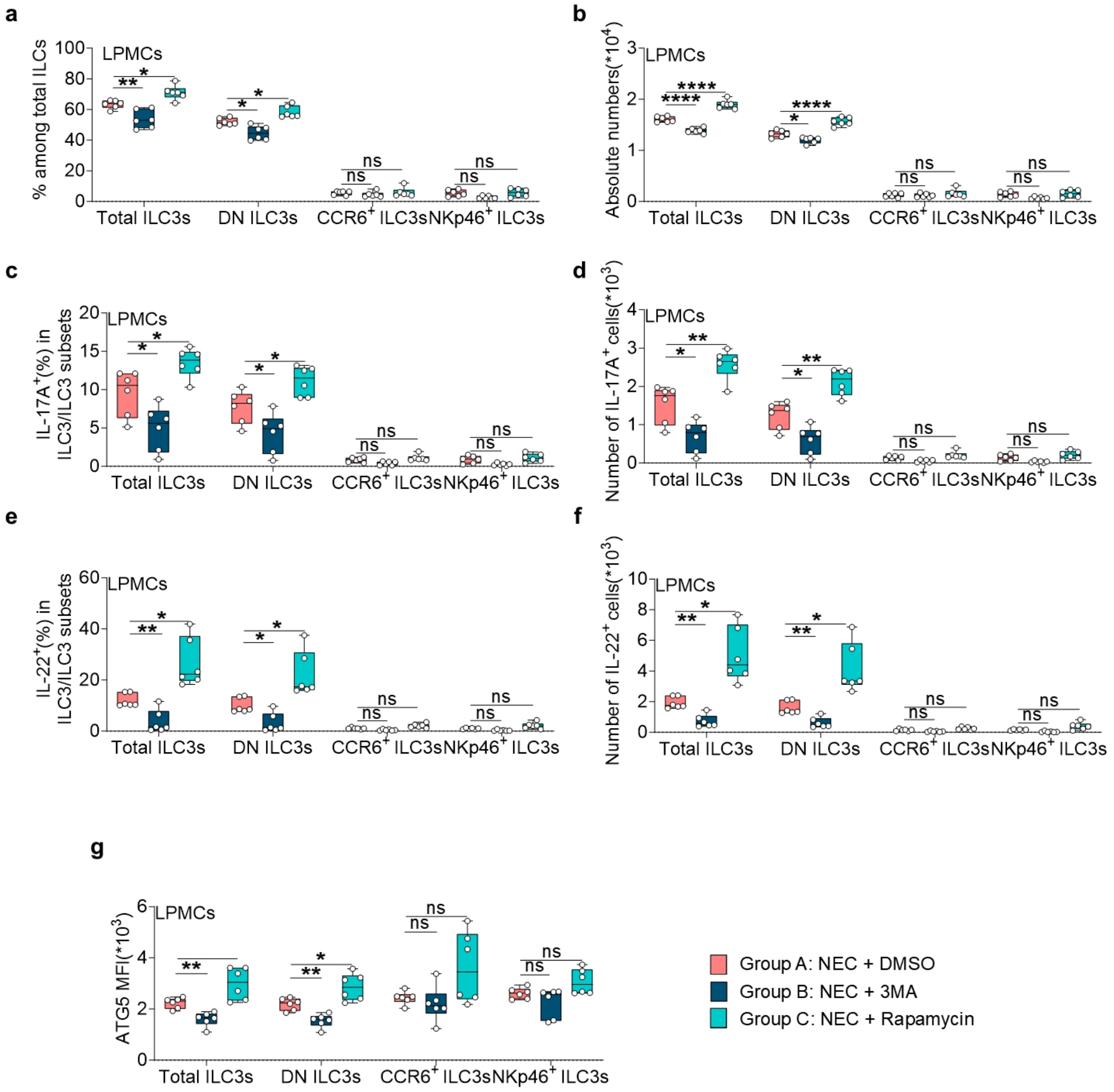

Figure G. DN ILC3 autophagy plays a crucial role in NEC development. **(a, b)** Percentage and absolute number of total ILC3s, DN ILC3s, CCR6+ ILC3s and NKp46+ ILC3s ($n = 6$). **(c, d)** Percentage and number of IL-17A+ ILC3s and IL-17A+ ILC3 subsets ($n = 6$). **(e, f)** Percentage and number of IL-22+ ILC3s and IL-22+ ILC3 subsets ($n = 6$). **(g)** The protein levels of ATG5 in ILC3s and ILC3 subsets ($n = 6$). Error bars represent the SEM. *$p < 0.05$; **$p < 0.01$; ***$p < 0.001$; ns, not significant. Statistical significance was analyzed by One-way ANOVA (**a-g**).

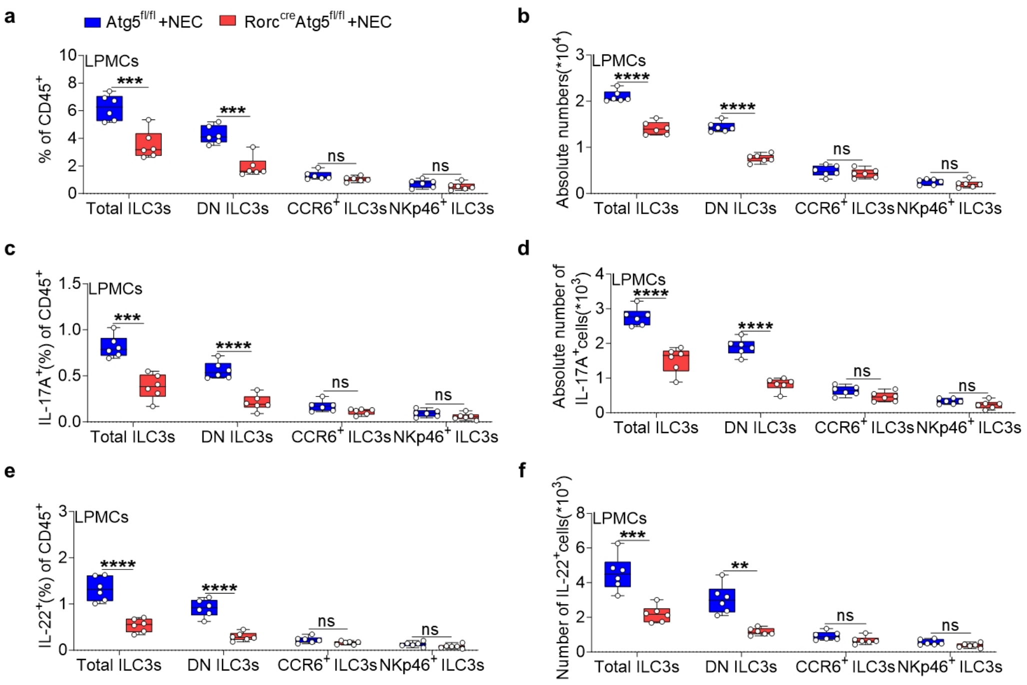

Figure H. ATG5 is key to DN ILC3s in driving NEC progression. **(a, b)** Percentage and absolute number of total ILC3s, DN ILC3s, CCR6$^+$ ILC3s, and NKp46$^+$ ILC3s ($n = 6$). **(c, d)** Percentage and number of IL-17A$^+$ ILC3s and IL-17A$^+$ ILC3 subsets ($n = 6$). **(e, f)** Percentage and number of IL-22$^+$ ILC3s and IL-22$^+$ ILC3 subsets ($n = 6$). Each symbol represents one pup from a litter, and data are from at least three independent experiments. Error bars represent the SEM. $^*p < 0.05$; $^{**}p < 0.01$; $^{***}p < 0.001$; ns, not significant. Statistical significance was analyzed by Student's t test **(a-f)**.

Collectively, these findings demonstrate that ATG5-mediated autophagy selectively drives the expansion and pathogenic function of DN ILC3s during NEC. Although ATG5 is upregulated in all ILC3 subsets, its functional effects—on cell numbers and IL-17A/IL-22 production—are restricted to DN ILC3s, highlighting this axis as key to their pathogenic role.

The supplementary data and corresponding description have been incorporated into the revised version (Figure 3f–h, l–n; Figure 4d–i; Supplementary Figure 14a–c; Supplementary Figure 15a, b; Supplementary Figure 16d, e; Supplementary Figure 17a–c; Supplementary Figure 19b, c, i, j) (Page 12, Lines 252–256; Page 12, Lines 262–271; Page 14, Lines 297–305).

5. Is Glut1 expression (or other Glucose transporters) altered in NEC DN ILC3s? What is the main fuel for glycolysis in NEC DN ILC3s? Ideally, experiments using carbon-labeled glucose or lactate in vitro/in vivo would showcase which carbon source

is being used. I, of course, understand that such experiments may be challenging. Instead, in vitro experiments using exogenous lactate or glucose and an evaluation of glycolysis in NEC DN ILC3s should provide enough evidence for the identification of the carbon source used by these cells.

Reply: We thank our reviewer for the insightful comment regarding the glucose transporter expression and the carbon source driving glycolysis in NEC DN ILC3s. To address these points, we have re-analyzed our RNA-seq data and established an in vitro system. The corresponding results are presented below.

- The glycolytic activity in DN ILC3s from the bread-fed group (CON) and the NEC model group (NEC) was analyzed. Heatmap revealed significant upregulation of glycolysis-related genes in NEC DN ILC3s (Figure I a). Consistently, quantitative real-time PCR and flow cytometry confirmed that the expression of glucose transporters *Glut1* and *Glut3* was markedly increased in these cells (Figure I b–d). This alteration was accompanied by elevated lactate production, confirming enhanced glycolytic flux in pathogenic DN ILC3s (Figure I e).
- To determine the main carbon source driving glycolysis, we established an *in vitro* system using glucose or lactate (Figure J a). Glucose stimulation significantly upregulated the expression of glycolysis-related genes—*Hk1*, *Pkm*, *Aldoa*, and *Ldha*—compared to PBS controls. In contrast, lactate had no significant effects on these genes (Figure J b).

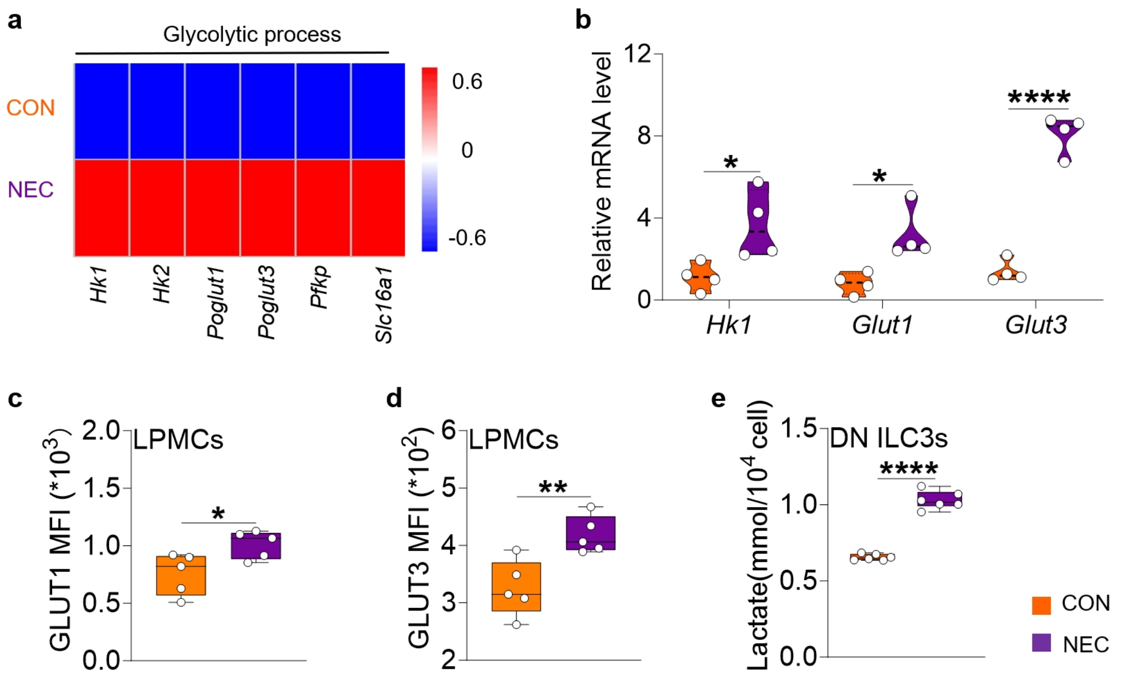

Figure I. Glycolysis are enhanced in NEC DN ILC3s. (**a**) Heatmap showing the expression of glycolysis-related genes in DN ILC3s. (**b**) Relative mRNA levels of *Hk1*, *Glut1*, and *Glut3* in DN ILC3s (n = 4 biological replicates). (**c**, **d**) The protein levels of GLUT1 and GLUT3 in DN ILC3s ($n = 5$). (**e**) Statistical analysis of lactate levels ($n = 6$). Each symbol represents one pup from a litter, and data are from at least three independent experiments. Error bars represent the SEM. $*p < 0.05$; $**p < 0.01$; $***p < 0.001$; ns, not significant. Statistical significance was analyzed by Student's t test (**b**–**e**).

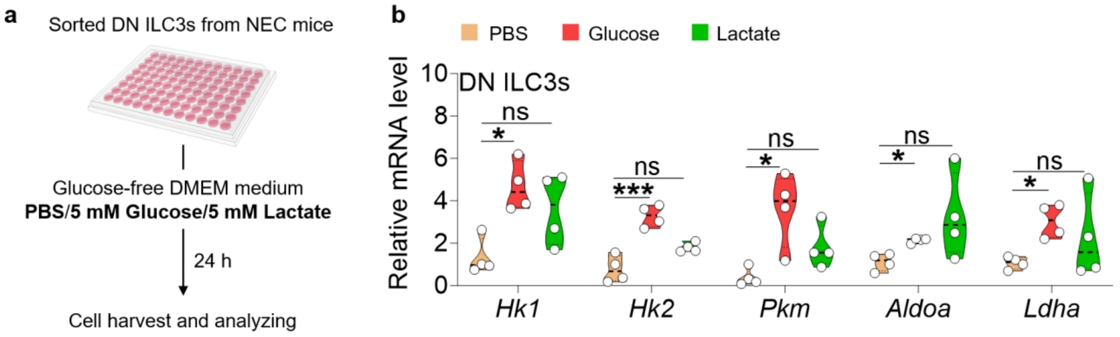

Figure J. Glucose serves as a key carbon source for glycolysis in NEC DN ILC3s. (**a**) Schematic workflow of the in vitro metabolic perturbation assay. DN ILC3s were sorted from NEC mice and cultured in glucose-free medium supplemented with PBS, 5 mM glucose, or 5 mM lactate for 24 h. (**b**) Relative mRNA levels of *Hk1*, *Hk2*, *Pkm*, *Aldoa* and *Ldha* ($n = 4$ biological replicates). Each symbol represents one pup from a litter, and data are from at least three independent experiments. Error bars represent the SEM. $*p < 0.05$; $**p < 0.01$; $***p < 0.001$; ns, not significant. Statistical significance was analyzed by Student's t test (**b**).

These findings indicate that NEC DN ILC3s rely on glucose as their core metabolic substrate, with GLUT1 and GLUT3 mediating enhanced glycolytic activity. Lactate, while elevated as a byproduct, does not serve as a major feedback regulator of glycolysis in these cells.

The supplementary data and corresponding description have been incorporated into the revised version (Supplementary Figure 21d–j), (Page 15–16, Lines 338–354)

Minor:
1. A revision of text for clarity and minor typos will enhance the quality of the manuscript.

Reply: We are grateful for your thoughtful comments and apologize for the careless typos in our previous version. We have thoroughly reviewed the manuscript to correct minor typos, grammatical errors, terminology inconsistencies, and improve clarity. We believe these revisions have strengthened the clarity and rigor of our manuscript. Additionally, the revised manuscript has been professionally edited by Editage (www.editage.cn) to enhance its readability.

2. The experiments with lactate and etomoxir should also include Atg5fl controls.

Reply: We sincerely appreciate your valuable suggestion. We apologize for omitting the $Atg5^{fl/fl}$ control data from our initial submission. These experiments were actually performed, but were not included due to negative results. We fully agree that negative controls are equally important, and have incorporated these data into the revised manuscript to improve the rigor of our study. The corresponding results are presented below.

- $Atg5^{fl/fl}$ mice were treated with vehicle control (DMSO), lactate, or etomoxir during NEC induction (Figure K a). Lactate or etomoxir alone did not significantly alter NEC severity, body weight, or ILC3 level in $Atg5^{fl/fl}$ mice (Figure K b, c).
- We therefore used DMSO-treated $Atg5^{fl/fl}$ mice as the control group for all subsequent comparisons. However, in $Rorc^{cre}Atg5^{fl/fl}$ mice, lactate or etomoxir treatment abolished the protection against NEC, exacerbating weight loss and selectively expanding DN ILC3s while increasing their IL-17A and IL-22 production (Figure L a–j).

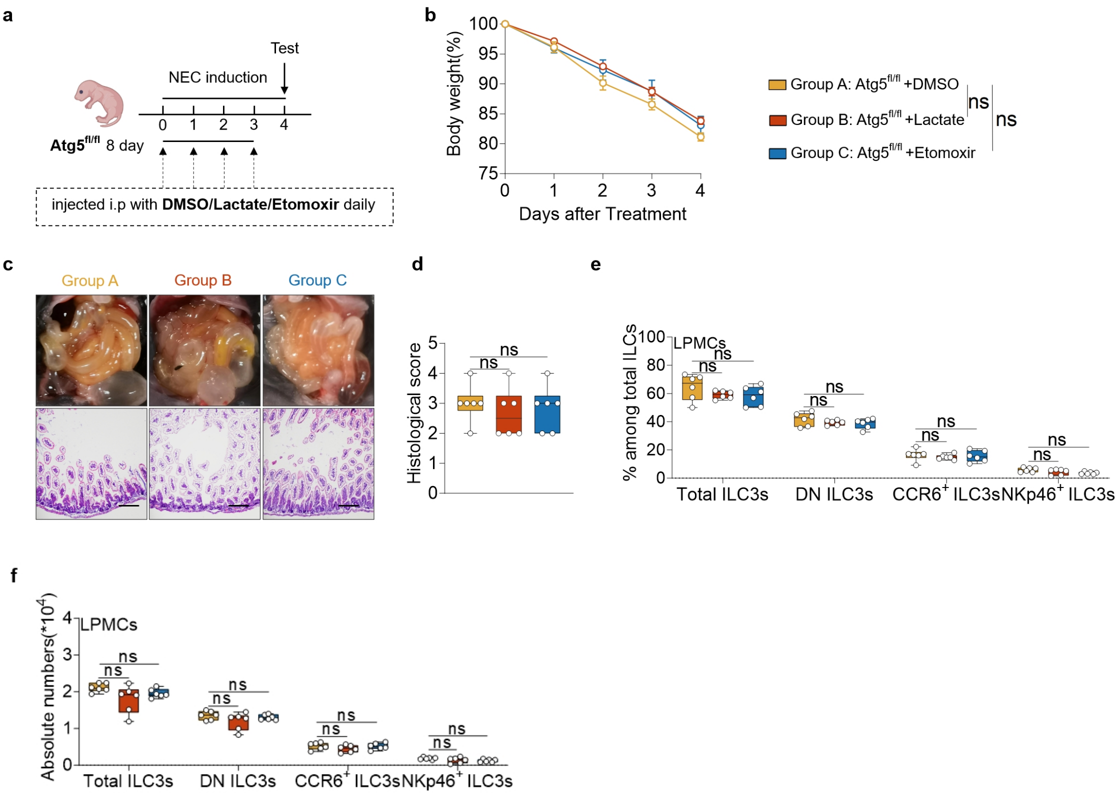

Figure K. No significant immune response in Atg5$^{fl/fl}$ mice treated with lactate or etomoxir. (**a**) Schematic representation of adoptive transfer of ILC3 subsets at physiological relevant ratios. (**b**) Body weight changes of the indicated groups ($n = 6$). (**c**) Macroscopic image and representative H&E staining of the intestines (scale bar: 100 μm). (**d**) Statistical analysis of inflammation scores ($n = 6$).(**e**, **f**) Percentage and absolute number of total ILC3s, DN ILC3s, CCR6$^+$ ILC3s, and NKp46$^+$ ILC3s ($n = 6$). Each symbol represents one pup from a litter, and data are from at least three independent experiments. Error bars represent the SEM. *$p < 0.05$; **$p < 0.01$; ***$p < 0.001$; ns, not significant. Statistical significance was analyzed by One-way ANOVA (**b,d–f**).

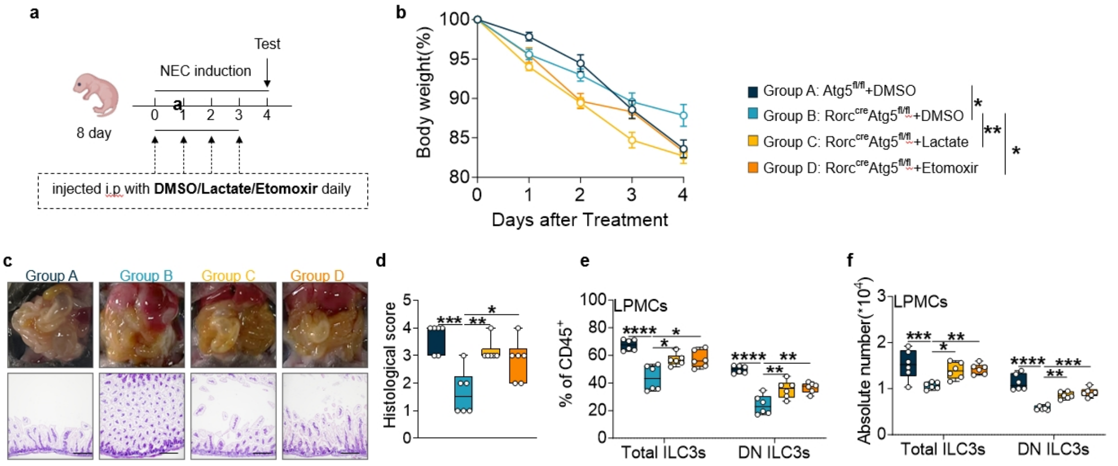

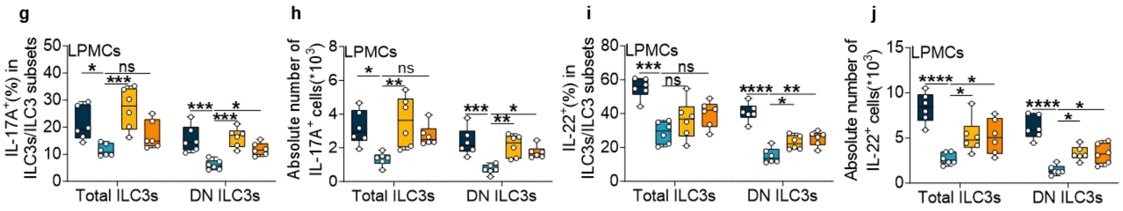

Figure L. Altered intestinal DN ILC3 glycolipid metabolic balance exacerbates NEC in *Atg5*-deficient mice. (**a**) Schematic diagram of metabolic interventions during NEC induction. (**b**) Body weight changes in the indicated groups ($n = 6$). (**c**) Macroscopic image and H&E staining of the intestines (scale bar: 100 μm). (**d**) Statistical analysis of inflammation scores ($n = 6$). (**e**, **f**) Percentage and absolute number of intestinal ILC3s and DN ILC3s ($n = 6$). (**g**, **h**) Percentage and absolute number of intestinal IL-17A$^+$ ILC3s and IL-17A$^+$ DN ILC3s ($n = 6$). (**i**, **j**) Percentage and absolute number of intestinal IL-22$^+$ ILC3s and IL-22$^+$ DN ILC3s ($n = 6$). Each symbol represents one pup from a litter, and data are from at least three independent experiments. Error bars represent the SEM. *$p < 0.05$; **p < 0.01; ***p < 0.001; ns, not significant. Statistical significance was analyzed by One-way ANOVA (**b** and **d**–**j**).

These data confirm that neither lactate nor etomoxir alters ILC3 populations in Atg5$^{fl/fl}$ control mice, providing evidence for using DMSO-treated Atg5$^{fl/fl}$ mice as the control group.

The supplementary data and corresponding description have been incorporated into the revised version (Figure 6a–h) (Supplementary Figure 26a–e), (Page 17–18, Lines 384–404).

Response to Reviewer #3

We sincerely appreciate the time and effort you have dedicated to reviewing our manuscript. Your valuable feedback and suggestions have significantly improved the quality of our paper. In response, we have thoroughly revised the manuscript and incorporated substantial new experimental data to strengthen the credibility of our findings. All modifications in the revised manuscript have been **indicated in blue text** for your convenience. Please refer to the detailed, point-by-point response to each comment below. We sincerely hope the revised version meets your expectations and look forward to your approval.

The manuscript by He et al. investigates the role of ILC3s in necrotizing enterocolitis (NEC) through the $NKp46^{-}CCR6$ autophagy pathway. Using single-cell transcriptomics, murine models, and targeted interventions, the authors show that attenuation of NEC can be achieved by reducing ILC3 abundance via ATG5 knockdown or autophagy inhibition. They further link enhanced fatty acid metabolism with reduced glycolysis, while inhibition of fatty acid oxidation restored functionality of ATG5-deficient $NKp46^{-}CCR6^{-}$ ILC3s, exacerbating disease. Phosphatidylcholine (PC) supplementation improved intestinal inflammation by inhibiting $NKp46^{-}CCR6^{-}$ autophagy. The clinical relevance of this work is supported by patient data demonstrating elevated ILC3 levels and enrichment of autophagy-related genes in NEC intestines. Given the complex and poorly understood pathophysiology of NEC, and the ongoing search for novel therapeutic strategies, this study will be of interest to the readership of Nature Communications, though several substantive issues, particularly relating to ILC3 subsets and the autophagy-metabolism-ILC3-NEC axis, require additional data clarification before publication.

Reply: We thank our reviewer for the comprehensive, fair, and insightful evaluation of our manuscript.

Major comments:

1. The manuscript overstates novelty. Prior work has already implicated ILC3 dysregulation in NEC (e.g., Cho et al., Nat Commun 2020; Zhao et al., Cell Biosci 2022). While focusing on DN ILC3s is of interest, the functional distinction between DN, $CCR6^{+}$, and $NKp46^{+}$ subsets is incompletely addressed. More work is needed to convincingly establish DN ILC3s as the causal drivers of NEC pathology.

Reply: We are grateful for your insightful comment. We sincerely apologize for the overstatement and lack of focus in our initial submission. To address these concerns, we have re-clarified the novelty of our work and provided extensive data distinguishing the role of three ILC3 subsets, as detailed below.

(1) As you pointed out, previous studies have implicated ILC3 dysregulation in NEC:

- Cho et al., (*Nat. Commun.* 2020, 10.1038/s41467-020-19400-w) reported that pro-inflammatory ILC3s are 5-fold increased during NEC.
- Zhao X.., et al (*Cell Biosci.* 2022, 10.1186/s13578-021-00739-6) showed a high percentage of RORγt^+ ILC3s during the first week, and this population was increased in both NEC mice and patients.
- Zhang Y., et al., (*Immunity*, 2025, 10.1016/j.immuni.2025.02.003) connected IL-22-producing ILC3s to NEC.

While these findings established a link between ILC3s and NEC, they did not functionally distinguish among ILC3 subsets. Our study extends these findings by identifying the specific ILC3 subset—NKp46$^-$CCR6$^-$ double negative (DN) ILC3s—as the key pathogenic driver in NEC.

(2) We performed scRNA-seq on sorted ILC3s from the breast-fed group (CON) and the NEC model group (NEC) to identify NEC-associated ILC3 subsets, finding that DN ILC3s were the predominant subset with significant expansion in NEC (Figure A a, b). Flow cytometry validated the increased frequency and absolute count of DN ILC3s in NEC-induced mice (Figure A c–e).

(3) To directly assess the specific role of DN ILC3s, we performed adoptive transfer experiments under two conditions:

- Each ILC3 subset was sorted and adoptively transferred into recipient mice at doses reflecting their respective *in vivo* frequencies (Figure B a). Notably, only transfer of DN ILC3s, but not NKp46$^+$ or CCR6$^+$ ILC3s, significantly exacerbated weight loss and intestinal inflammation (Figure B b–d).
- In addition, the experiment using equal numbers of each ILC3 subset was performed to exclude potential confounding by differences in cell number (Figure C a). Consistently, only DN ILC3s—but not NKp46$^+$ or CCR6$^+$ ILC3s—significantly exacerbated NEC pathology (Figure C b–d).

(4) To further assess the subset-specific pathogenicity of DN ILC3s in NEC, we adoptively transferred DN ILC3s isolated from control and NEC neonates into wild-type recipients (Figure D a). DN ILC3s from NEC mice induced significantly

more severe weight loss and intestinal inflammation than those from controls (Figure D b–d).

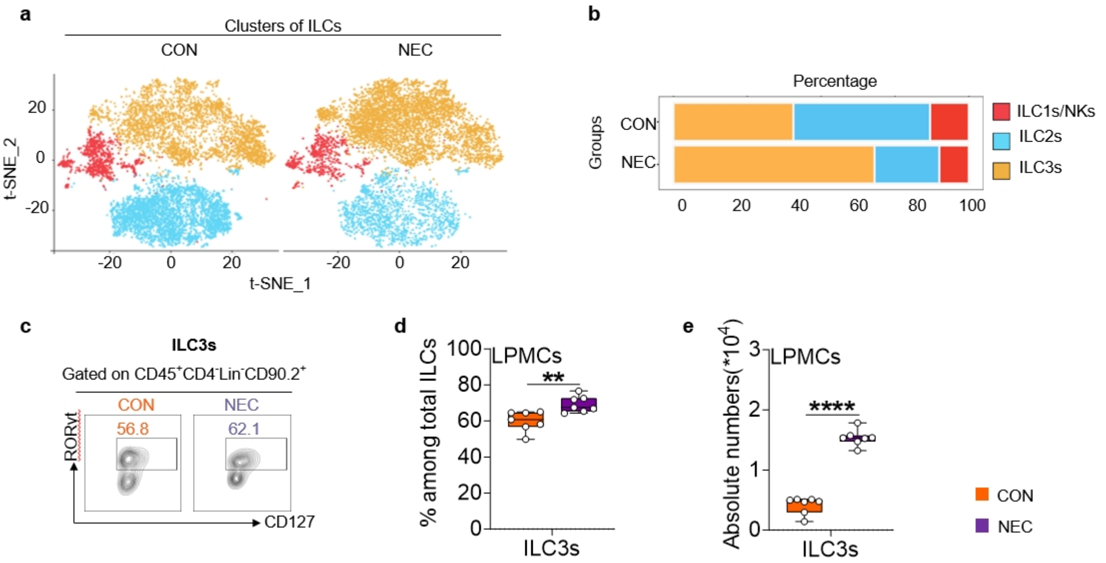

Figure A. DN ILC3s are increased during NEC. (**a**) t-Distributed Stochastic Neighbor Embedding (t-SNE) analysis comparing innate lymphoid cells (ILCs) clusters in the intestines of CON and NEC neonatal mice. (**b**) The median percentage of each ILC cluster, color-coded to match the populations in (a). (**c**) Representative flow cytometry profiles of DN ILC3s in the intestinal lamina propria. (**d**, **e**) Percentage and absolute number of intestinal ILC3s (n = 7). Each symbol represents one pup from a litter, and data are from at least three independent experiments. Error bars represent the SEM. *$p < 0.05$; **$p < 0.01$; ***$p < 0.001$; ns, not significant. Statistical significance was analyzed by Student’s t test (**d** and **e**).

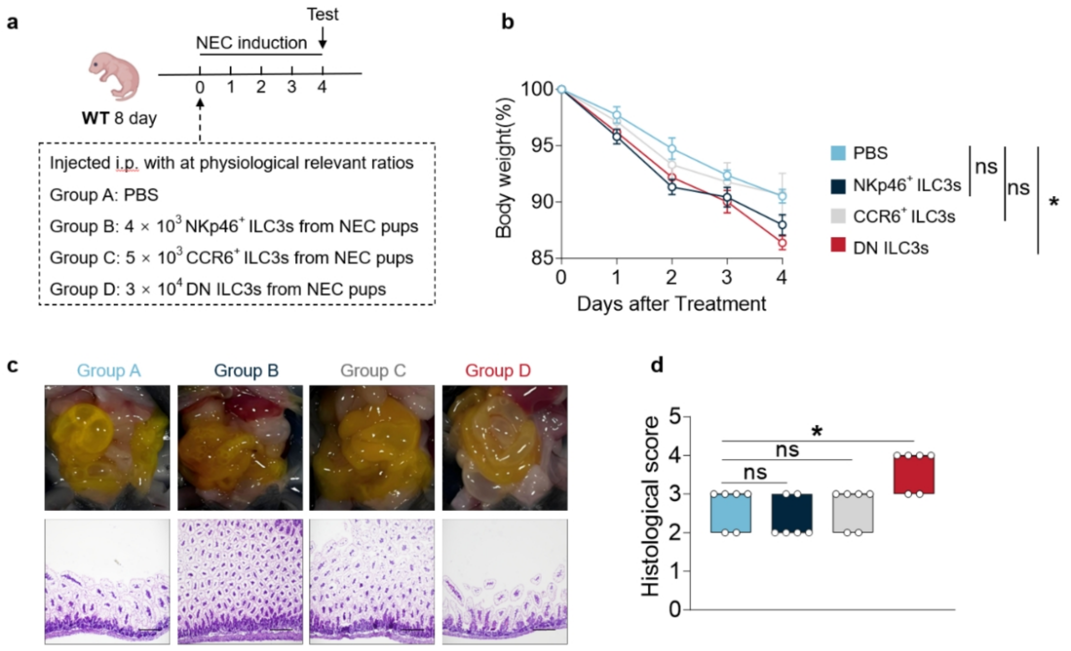

Figure B. Adoptive transfer of three ILC3 subsets at doses matching their in vivo frequencies. (**a**) Schematic representation of adoptive transfer of ILC3 subsets at physiological relevant ratios. (**b**) Body weight changes of the indicated groups ($n = 6$). (**c**) Macroscopic image and representative H&E staining of the intestines (scale bar: 100 μm). (**d**) Statistical analysis of inflammation scores ($n = 6$). Each symbol represents one pup from a litter, and data are from at least three independent experiments. Error bars represent the SEM. *$p < 0.05$; **$p < 0.01$; ***$p < 0.001$; ns, not significant. Statistical significance was analyzed by One-way ANOVA (**b** and **d**).

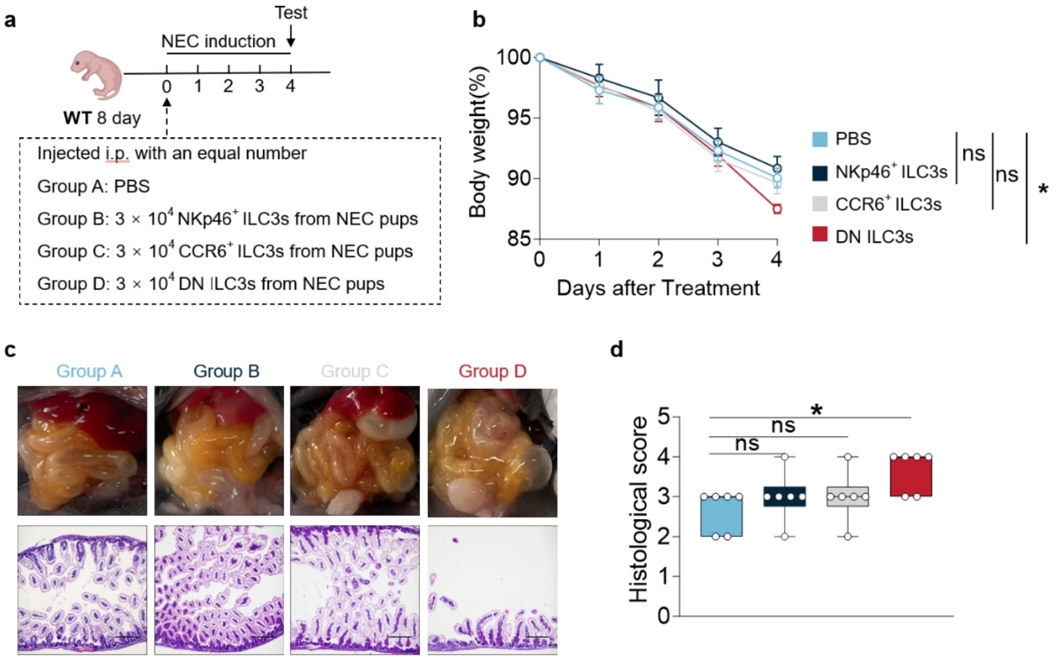

Figure C. Adoptive transfer of DN ILC3s, but not NKp46$^+$ or CCR6$^+$ ILC3s, exacerbates NEC severity. (**a**) Schematic representation of adoptive transfer of ILC3 subsets at equal numbers. (**b**) Body weight changes of the indicated groups ($n = 6$). (**c**) Macroscopic image and representative H&E staining of the intestines (scale bar: 100 μm). (**d**) Statistical analysis of inflammation scores ($n = 6$). Each symbol represents one pup from a litter, and data are from at least three independent experiments. Error bars represent the SEM. *$p < 0.05$; **$p < 0.01$; ***$p < 0.001$; ns, not significant. Statistical significance was analyzed by One-way ANOVA (**b** and **d**).

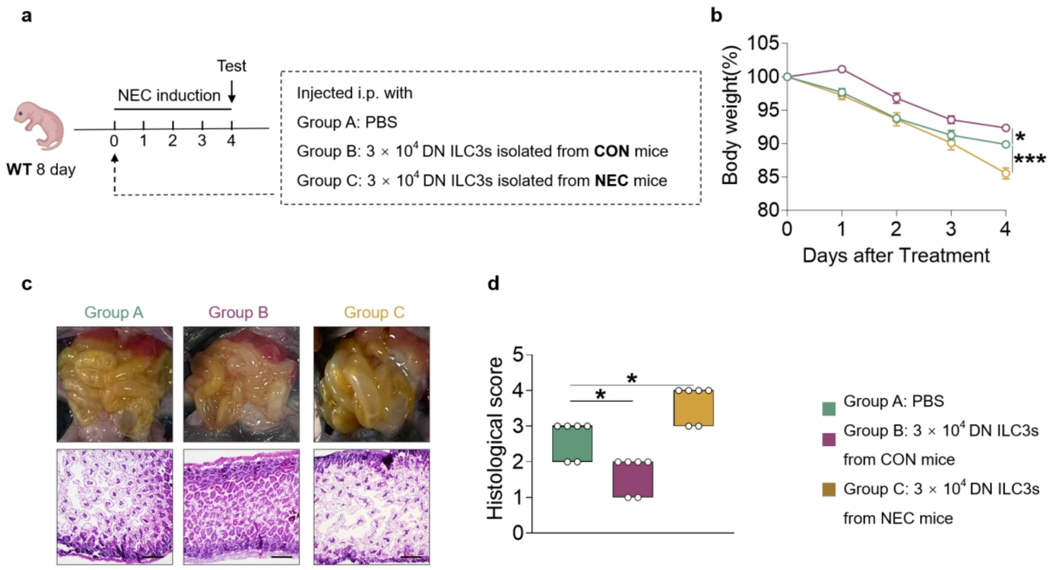

Figure D. Pathogenic DN ILC3s promote NEC severity. (**a**) Experimental schema of adoptive cell transfer during NEC induction. (**b**) Body weight changes of the indicated groups ($n = 6$). (**c**) Macroscopic image and representative H&E staining of the intestines (scale bar: 100 μm). (**d**) Statistical analysis of inflammation scores ($n = 6$). Each symbol represents one pup from a litter, and data are from at least three independent experiments. Error bars represent the SEM. $*p < 0.05$; $**p < 0.01$; $***p < 0.001$; ns, not significant. Statistical significance was analyzed by One-way ANOVA (**b** and **d**).

Collectively, these data demonstrate that DN ILC3s, not NKp46$^+$ or CCR6$^+$ ILC3s, are the key pathogenic ILC3 subset in NEC.

The supplementary data and corresponding description have been incorporated into the revised version (Figure 1a–c; Figure 2a–c; Supplementary Figure 7a–f), (Page 6–7, Lines 132–140; Page 8–9 Lines 175–185; Page 9 Lines 186–191).

In addition, we have thoroughly revised the Introduction and Discussion to remove overstated claims and appropriately contextualize our findings with the existing literature. These revisions have been incorporated into the revised version (Page 4, Lines 64–78; Page 21–22, Lines 490–495).

2. The role of autophagy in immune cell metabolic regulation is well-established. The incremental advance here is its application to DN ILC3s in NEC, which is narrower in scope than framed.

Reply: We thank our reviewer for the careful reading. We apologize that the initial submission did not adequately frame the background of ILC3 autophagy and instead presented a more general introduction of autophagy in immune cells, which may have

obscured the specific focus of our work. To address this issue, we have thoroughly revised the Introduction to directly center on ILC3 autophagy and its role in NEC pathogenesis. The specific revisions are presented below.

Although direct evidence linking ILC3 metabolism to NEC pathogenesis remains scarce, autophagy has emerged as a key contributor to disease development. In murine models of NEC, TLR4 activation induces autophagy in IECs, impairing epithelial migration and promoting disease progression (*Autophagy*, 2020, 10.1080/15548627.2019.1622216). Additionally, elevated expression of autophagy related genes in intestinal tissues from NEC patients further implicates autophagy as a critical pathogenic mechanism (*J Immunol*, 2013, 10.4049/jimmunol.1202513) (*Am J Physiol Gastrointest Liver Physiol*, 2010, 10.1152/ajpgi.00020.2010). Beyond its pathogenic effects, autophagy functions as a central regulator of cellular metabolism, shaping energy homeostasis by modulating glucose and fatty acid metabolism, as well as mitochondrial bioenergetic pathways in immune cells (*J Exp Med*, 2018, 10.1084/jem.20171375) (*Immunity*, 2017, 10.1016/j.immuni.2017.08.005). For example, autophagy provides free fatty acids through lipophagy, the selective degradation of lipid droplets, facilitating a metabolic shift from glycolysis to FAO and oxidative phosphorylation (OXPHOS) to sustain cellular energy demands (*J Allergy Clin Immunol*, 2020, 10.1016/j.jaci.2019.09.021) (*J Immunol*, 2011, 10.4049/jimmunol.1003236) (*J Exp Med*, 2011, 10.1084/jem.20110278) (*Nat Med*, 2014, 10.1038/nm.3703). While immunometabolic crosstalk in immune cell regulation is well established, the role of an autophagy–energy metabolism axis in controlling DN ILC3 activity during NEC remains unexplored. Clarifying this mechanism will deepen our understanding of how autophagy-driven DN ILC3s contribute to NEC pathogenesis, and holds promise for identifying cell-specific therapeutic targets. Such insights may guide the development of novel strategies for the prevention, early diagnosis, or treatment of NEC in preterm infants.

The corresponding description have been incorporated into the revised version (Page 5, Lines 89–104).

3. The introduction states that NEC affects the terminal ileum and proximal colon. While colonic involvement can occur, it is less common; NEC primarily impacts the small intestine, particularly the distal ileum. This statement should be revised to avoid overemphasizing colonic disease and to more accurately reflect the typical distribution

of NEC.

4. The authors describe an exaggerated neutrophil response as a contributor in the context of IL-17/IL-22 signaling. For clarity and relevance, this section would be stronger if discussed in the context of small bowel pathology, given that NEC predominantly affects the small intestine.

5. The last paragraph identifies the novel hypothesis (autophagy–energy metabolism axis in DN ILC3s during NEC) but comes after a large amount of mechanistic background. The novelty could be signposted earlier in the introduction (e.g., after briefly describing known ILC3 subsets, stating that DN ILC3s remain poorly understood in NEC). This would make the study's rationale more obvious to readers upfront.

6. It would be helpful if the introduction framed not only the mechanistic gap but also the translational implications—how studying DN ILC3 autophagy could ultimately inform NEC prevention, earlier diagnosis, or therapy in preterm infants. This would better justify the clinical importance of the work.

Reply: We thank the reviewers for their insightful feedback on our Introduction. Their comments focused on four main areas: (i) the location of NEC onset, (ii) the associated pathology, (iii) the study's innovative aspects, and (iv) its potential clinical relevance. Our specific revisions are as follows.

- Pathologically, NEC is characterized by inflammation and necrosis of the small intestine, predominantly the distal ileum (*Pediatr Res*, 2020, 10.1038/s41390-020-1074-4).
- Intestinal dysbiosis triggers Toll-like receptor 4 (TLR4) signaling in epithelial cells, compromising intestinal barrier integrity and facilitating bacterial translocation, thereby increasing susceptibility to sepsis (*Proc Natl Acad Sci U S A*, 2013, 10.1073/pnas.1219997110) (*Nat Rev Gastroenterol Hepatol*, 2016, 10.1038/nrgastro.2016.119) (*Shock*, 2020, 10.1097/shk.0000000000001487). TLR4 activation also directly drives epithelial cell death through apoptosis, autophagy, and necroptosis (*Early Hum Dev*, 2016, 10.1016/j.earlhumdev.2016.01.013) (*Nat Rev Gastroenterol Hepatol*, 2022, 10.1038/s41575-022-00594-x) (*J Biol Chem*, 2014, 10.1074/jbc.M113.534444) (*PLoS One*, 2013, 10.1371/journal.pone.0069620). This epithelial damage is worsened by impaired proliferation and mucosal repair, and amplified by neutrophil infiltration into the small intestinal mucosa, exacerbating NEC-associated inflammation (*J Biol Chem*, 2012, 10.1074/jbc.M112.378536) (*Gastroenterology*, 2005, 10.1053/j.gastro.2005.02.007) (*Gastroenterology*, 2010,

10.1053/j.gastro.2009.09.045) (*J Exp Med*, 2021, 10.1084/jem.20200344).

- In addition, the small intestine harbors a poorly characterized "double-negative" subset: CCR6−NKp46− ILC3s (DN ILC3s) (*Nature*, 2013, 10.1038/nature11813) (*Immunity*, 2010, 10.1016/j.immuni.2010.10.017). Notably, the functional role of DN ILC3s in intestinal inflammation—especially in neonatal NEC—remains completely unknown. Given their unique phenotype and tissue localization, the precise contribution of DN ILC3s to NEC pathogenesis warrants in-depth investigation.
- While immunometabolic crosstalk in immune cell regulation is well established, the role of an autophagy–energy metabolism axis in controlling DN ILC3 activity during NEC remains unexplored. Clarifying this mechanism will deepen our understanding of how autophagy-driven DN ILC3s contribute to NEC pathogenesis, and holds promise for identifying cell-specific therapeutic targets. Such insights may guide the development of novel strategies for the prevention, early diagnosis, or treatment of NEC in preterm infants.

The corresponding text has been added to the Introduction of the revised manuscript

(Page 3–4, Lines 47–48, 48–55, 74–78, 99–104).

7. The gating strategy used to define CD4−RORγt+ cells as ILC3s requires stronger validation. Although lineage-negative gating was applied, additional confirmation that these cells are TCR− (e.g., explicit staining for TCRβ and TCRγδ within the RORγt+ population) is necessary to fully exclude unconventional T cells. Because the main conclusion of the manuscript is that DN ILC3s, and not Th17 cells, drive NEC, rigorous exclusion of T cell contaminants is critical to support the authors' claim.

Reply: We sincerely appreciate your valuable comment on the gating strategy for ILC3 identification. We fully agree that excluding T-cell contaminants is essential to validate our key conclusion. Therefore, we have performed additional flow cytometry validation experiments to exclude potential T cell contaminants. The corresponding results are presented below.

TCRαβ^{+} and TCRγδ^{+} cells within the CD45^{+}CD4^{-}RORγt^{+} gate were detected at extremely low frequencies in both CON and NEC mice, with no significant intergroup differences for either subset (Figures E a–f).

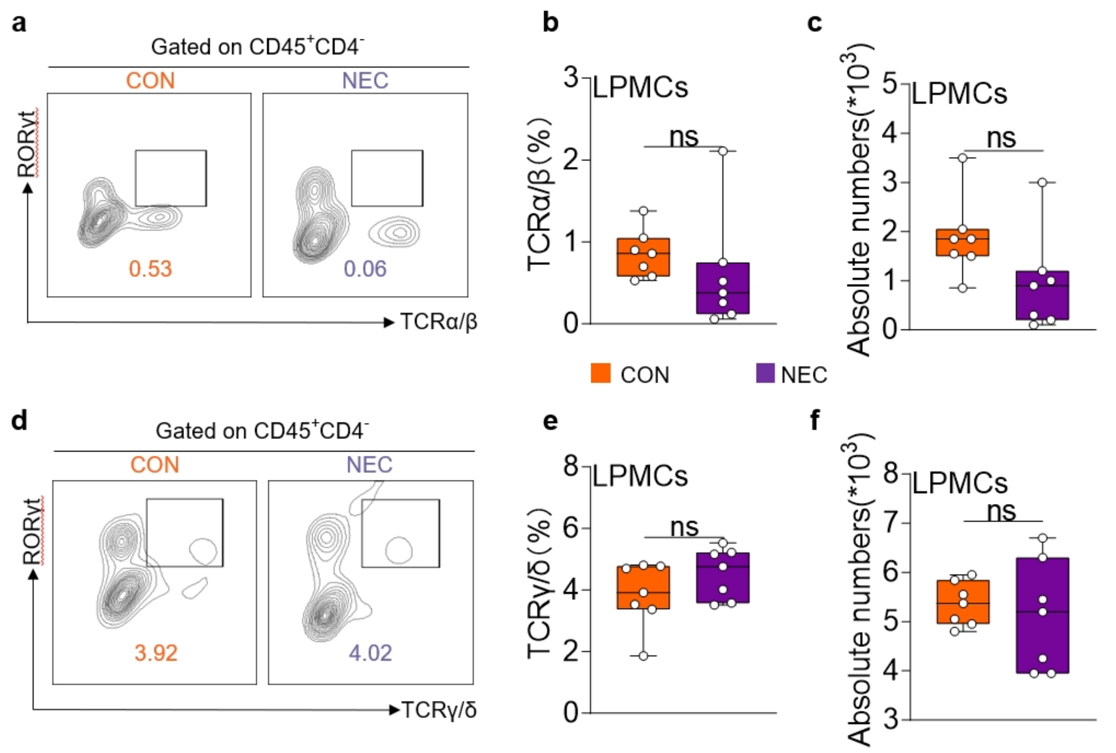

Figure E. TCRαβ and TCRγδ expression is low and unaltered in CD45$^+$CD4$^-$RORγt$^+$ cells during NEC. (**a**) Representative flow cytometry plots of RORγt and TCRαβ staining among CD45$^+$CD4$^-$ cells. (**b**, **c**) Percentage and absolute number of CD4$^-$RORγt$^+$TCRαβ$^+$ cells ($n = 7$). (**d**) Representative flow cytometry plots of RORγt and TCRγδ staining among CD45$^+$CD4$^-$ cells. (**e**, **f**) Percentage and absolute number of CD4$^-$RORγt$^+$TCRγδ$^+$ cells ($n = 7$). Each symbol represents one pup from a litter, and data are from at least three independent experiments. Error bars represent the SEM. *$p < 0.05$; **$p < 0.01$; ***$p < 0.001$; ns, not significant. Statistical significance was analyzed by Student's *t* test (**b, c, e, and f**).

These data confirm that T-cell contamination within the defined ILC3 gate is negligible and does not differ between experimental groups, supporting our conclusion that DN ILC3s, not T cells, are the key pathogenic drivers in NEC.

The supplementary data and corresponding description have been incorporated into the revised version (Supplementary Figure 4b, c), (Page 7, Lines 145–147).

8. Both NKp46$^+$ and CCR6$^+$ ILC3s expand numerically following NEC induction, yet the manuscript highlights DN ILC3s as the primary pathogenic subset. While the abundance of DN ILC3s is clear, relative numbers alone do not establish causality. Functional comparisons across subsets—for example, assessing per-cell cytokine output of purified NKp46$^+$, CCR6$^+$, and DN ILC3s—would more convincingly demonstrate that DN ILC3s are uniquely pathogenic in NEC.

9. Adoptive transfer experiments show NEC-derived DN ILC3s exacerbate disease, but

equivalent testing with NKp46$^+$ and CCR6$^+$ ILC3s is missing. Without side-by-side comparisons, it is premature to single out DN ILC3s as uniquely pathogenic.

Reply: We are grateful for your insightful suggestions and sincerely apologize for the confusion caused. The issues raised in points 8 and 9 both arise from our overly simplistic conclusion that DN ILC3s represent the sole pathogenic subgroup in NEC. Although our initial submission showed increased abundance of DN ILC3s and their pathogenicity via adoptive transfer, it lacked direct side-by-side functional and adoptive transfer comparisons with NKp46$^+$ and CCR6$^+$ ILC3 subsets. To address this, we have performed: (i) re-analysis of our scRNA-seq data; (ii) *in vitro* cytokine profiling of purified ILC3 subsets; and (iii) adoptive transfer experiments. The detailed results are presented below.

(1) Re-analysis of scRNA-seq data of sorted ILC3s from CON and NEC mice revealed that DN ILC3s exhibited the most pronounced upregulation of *Il17a* compared to NKp46$^+$ and CCR6$^+$ ILC3s, suggesting an enhanced pro-inflammatory transcriptional program driving their pathogenicity (Figures F a–c).

(2) To directly assess the functional profiles in each ILC3 subset, we sorted these cell populations and measured cytokine secretion. IL-17A production by DN ILC3s was significantly higher than that by NKp46$^+$ or CCR6$^+$ ILC3s (Figures F d). In contrast, GM-CSF and IL-22 secretion levels were comparable across all three subsets (Figures F e, f).

(3) To further assess the contributions of these ILC3 subsets in NEC, we performed two complementary sets of experiments based on *in vivo* frequencies and equal cell numbers.

- We first adoptively transferred each ILC3 subset into recipient mice at doses reflecting their respective *in vivo* frequencies (Figure G a). The results showed that mice receiving DN ILC3s exhibited significantly exacerbated disease, as evidenced by greater weight loss and higher intestinal inflammation, whereas those receiving NKp46$^+$ or CCR6$^+$ ILC3s showed no significant difference from the PBS control (Figure G b–d).
- To exclude potential confounding by differences in cell number, we repeated the experiment using equal numbers of each ILC3 subset (Figure H a). Consistently, only DN ILC3s—but not NKp46$^+$ or CCR6$^+$ ILC3s—significantly exacerbated

NEC pathology compared to controls (Figure H b–d).

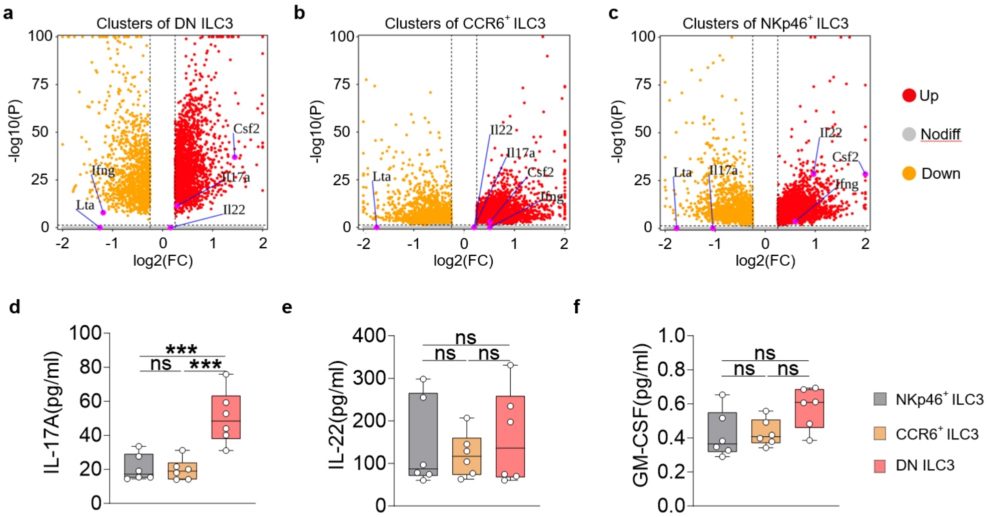

Figure F. DN ILC3s display enhanced pro-inflammatory transcriptional and functional profiles compared to NKp46⁺ and CCR6⁺ ILC3s in NEC. (**a**–**c**) Volcano plots showing differentially expressed genes in DN ILC3s, NKp46⁺ ILC3s, and CCR6⁺ ILC3s. (**d**–**f**) IL-17A, IL-22, and GM-CSF production by the indicated ILC3 subsets was measured ($n = 6$ biological replicates). Each symbol represents one pup from a litter, and data are from at least three independent experiments. Error bars represent the SEM. *$p < 0.05$; **$p < 0.01$; ***$p < 0.001$; ns, not significant. Statistical significance was analyzed by One-way ANOVA (**d**–**f**).

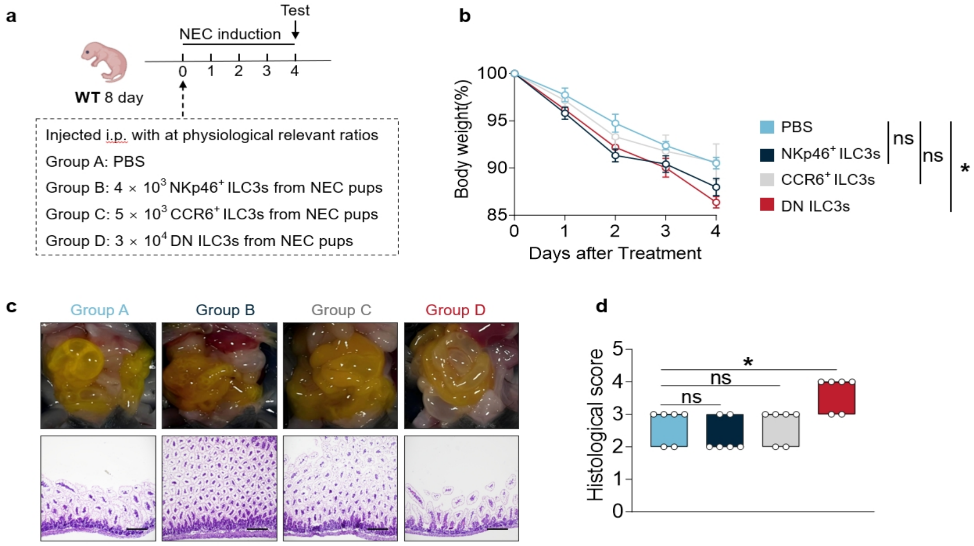

Figure G. Adoptive transfer of DN ILC3s, but not NKp46⁺ or CCR6⁺ ILC3s, exacerbates NEC severity. (**a**) Schematic representation of adoptive transfer of ILC3 subsets at physiological relevant ratios. (**b**) Body weight changes of the indicated groups ($n = 6$). (**c**) Macroscopic image and representative H&E staining of the intestines (scale bar: 100 μm). (**d**) Statistical analysis of inflammation scores ($n = 6$). Each symbol represents one pup from a litter, and data are from at

least three independent experiments. Error bars represent the SEM. *$p < 0.05$; **$p < 0.01$; ***$p < 0.001$; ns, not significant. Statistical significance was analyzed by One-way ANOVA (**b** and **d**).

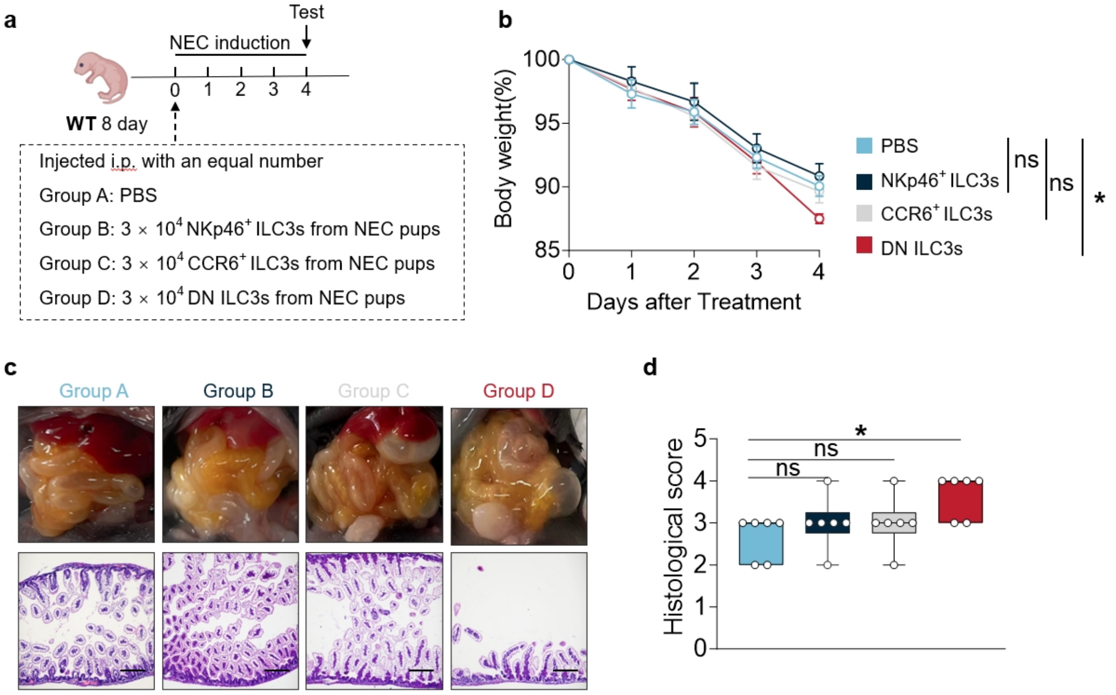

Figure H. Adoptive transfer of DN ILC3s, but not NKp46$^+$ or CCR6$^+$ ILC3s, exacerbates NEC severity. (**a**) Schematic representation of adoptive transfer of ILC3 subsets at equal numbers. (**b**) Body weight changes of the indicated groups ($n = 6$). (**c**) Macroscopic image and representative H&E staining of the intestines (scale bar: 100 μm). (**d**) Statistical analysis of inflammation scores ($n = 6$). Each symbol represents one pup from a litter, and data are from at least three independent experiments. Error bars represent the SEM. *$p < 0.05$; **$p < 0.01$; ***$p < 0.001$; ns, not significant. Statistical significance was analyzed by One-way ANOVA (**b** and **d**).

These data show that DN ILC3s produce significantly more IL-17A than the other ILC3 subsets and are the only pathogenic subset in a mice NEC model. Collectively, these findings establish a critical pathogenic role for DN ILC3s in NEC.

The supplementary data and corresponding description have been incorporated into the revised version (Figure 2i; Supplementary Figure 7a–f; Supplementary Figure 11b–d), (Page 8-9, Lines 175–185; Page 10, Lines 217–226).

10. The pharmacological interventions (3-MA and rapamycin) have pleiotropic effects across multiple cell types. While Atg5 conditional knockout experiments support the findings, the authors should explicitly address the limitations of systemic inhibition and how this might affect interpretation.

Reply: We thank our reviewer for the valuable comment regarding the

autophagy-related of our pharmacological interventions. To address this concern, we have performed *in vivo* pharmacological interventions, validated their direct effects on DN ILC3s *in vitro*, and confirmed the cell-intrinsic roles of autophagy using ILC3-specific *Atg5*-deficient mice. The corresponding results are presented below.

- Our data from the initial submission demonstrated that autophagy intervention modulates NEC disease progression. 3-MA reduced the frequency and absolute number of total ILC3s and DN ILC3s, while rapamycin increased these populations (Figure I a, b). Consistently, IL-17A and IL-22 levels in total ILC3s and DN ILC3s were decreased by 3-MA and increased by rapamycin (Figure I c–f). However, no such significant changes were observed in $CCR6^+$ or $NKp46^+$ ILC3 subsets (Figure I a–f).

- To directly assess the effect of 3-MA and rapamycin on DN ILC3s, we established an *in vitro* culture system. Consistent with *in vivo* findings, 3-MA significantly reduced IL-17A and IL-22 production by DN ILC3s, while rapamycin increased their levels (Figure J a, b). These data confirm that autophagy directly regulates DN ILC3 function.

- We validated the autophagic regulation of DN ILC3s using ILC3-specific *Atg5* conditional knockout ($Rorc^{cre}Atg5^{fl/fl}$) mice. Consistent with pharmacological results, *Atg5* ablation in ILC3s during NEC significantly reduced the frequency, absolute number, and IL-17A/IL-22 production of total ILC3s and DN ILC3s, with no significant effects on $NKp46^+$ or $CCR6^+$ ILC3s (Figure K a-f).

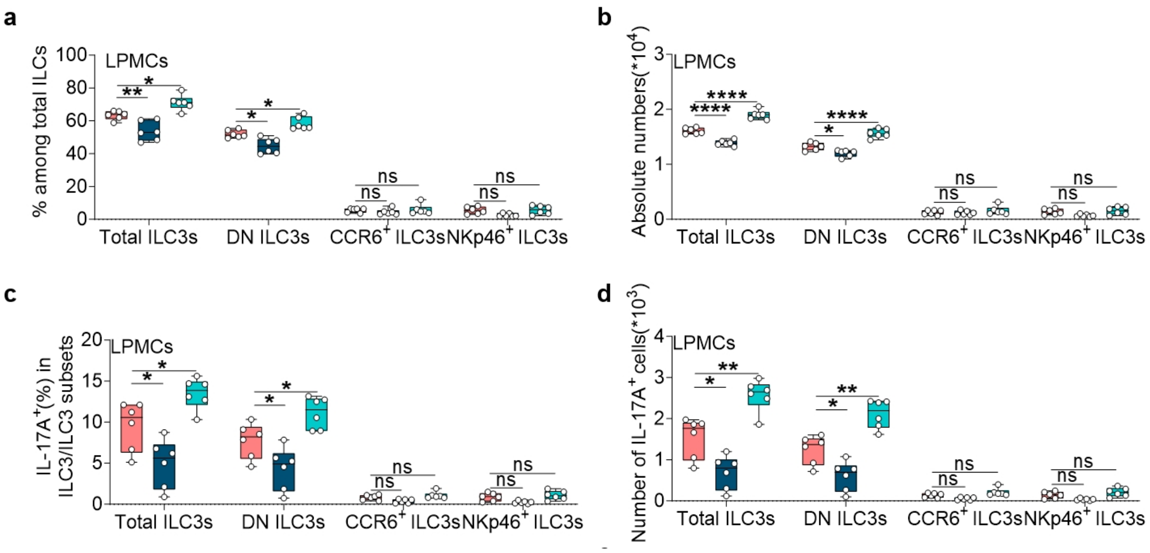

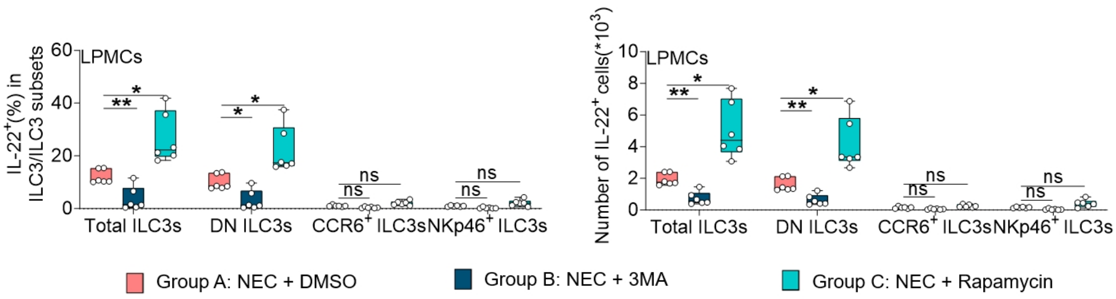

Figure I. Autophagy modulation regulates the abundance and pathogenic function of DN ILC3s in NEC mice. (**a**, **b**) Percentage and absolute number of intestinal total ILC3s, DN ILC3s, CCR6+ ILC3s and NKp46+ ILC3s ($n = 6$). (**c**, **d**) Percentage and number of intestinal IL-17A+ ILC3s and IL-17A+ ILC3 subsets. (**e**, **f**) Percentage and number of intestinal IL-22+ ILC3s and IL-22+ ILC3 subsets ($n = 6$). Each symbol represents one pup from a litter, and data are from at least three independent experiments. Error bars represent the SEM. *$p < 0.05$; **$p < 0.01$; ***$p < 0.001$; ns, not significant. Statistical significance was analyzed by Student's *t* test (**a–f**).

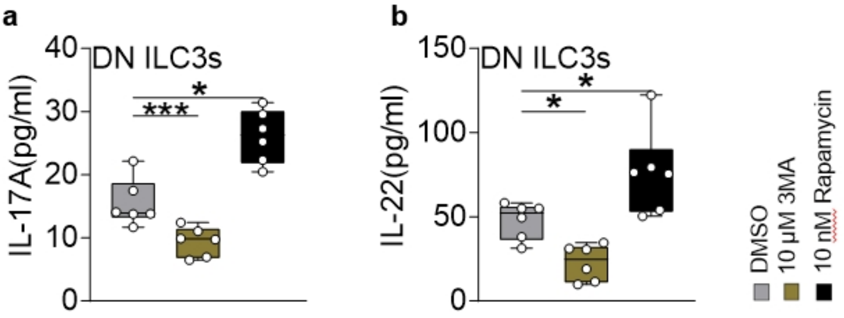

Figure J. Autophagy directly regulates DN ILC3 function. (**a**, **b**) Levels of IL-17A and IL-22 in DN ILC3s following 3-MA or rapamycin treatment in vitro ($n = 6$ biological replicates). Each symbol represents one pup from a litter, and data are from at least three independent experiments. Error bars represent the SEM. *$p < 0.05$; **$p < 0.01$; ***$p < 0.001$; ns, not significant. Statistical significance was analyzed by Student's *t* test (**a** and **b**).

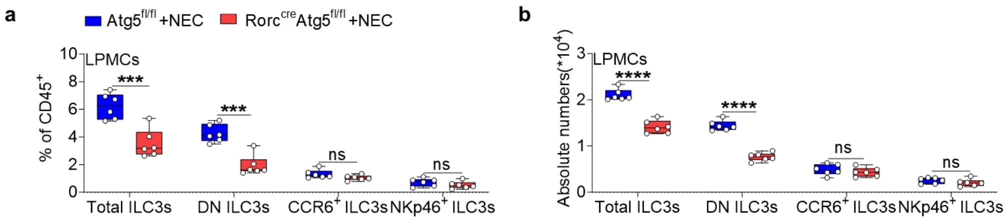

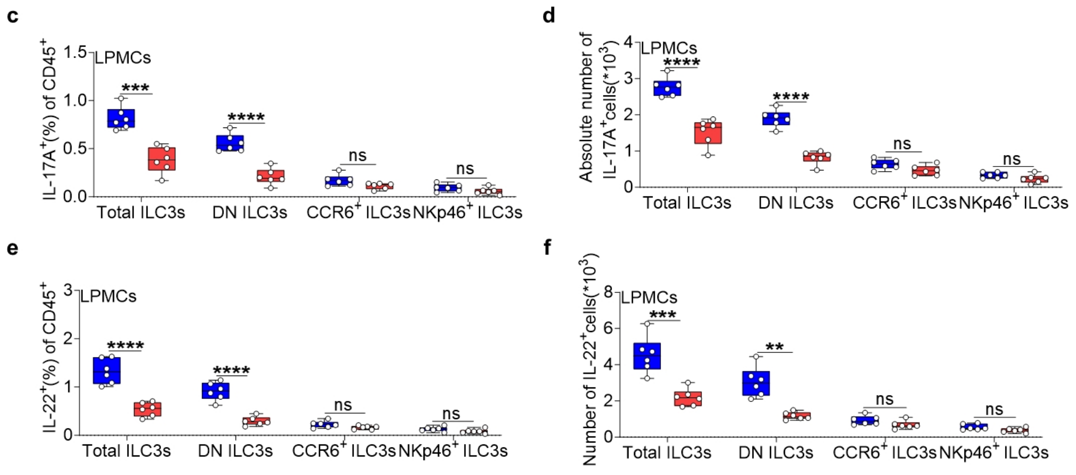

Figure K. ATG5 is essential for DN ILC3 pathogenicity in NEC. (**a**, **b**) Percentage and absolute number of intestinal total ILC3s, DN ILC3s, CCR6$^+$ ILC3s and NKp46$^+$ ILC3s ($n = 6$). (**c**, **d**) Percentage and number of intestinal IL-17A$^+$ ILC3s and IL-17A$^+$ ILC3 subsets. (**e**, **f**) Percentage and number of intestinal IL-22$^+$ ILC3s and IL-22$^+$ ILC3 subsets ($n = 6$). Each symbol represents one pup from a litter, and data are from at least three independent experiments. Error bars represent the SEM. $*p < 0.05$; $**p < 0.01$; $***p < 0.001$; ns, not significant. Statistical significance was analyzed by Student's *t* test (**a–f**).

Collectively, through pharmacological, *in vitro*, and genetic approaches, we demonstrate that autophagy in DN ILC3s is a critical driver of NEC pathogenesis.

The supplementary data and corresponding description have been incorporated into the revised version (Figure 3i–n, q; Figure 4a–i; Supplementary Figure 16e; Supplementary Figure 17a–c; Supplementary Figure 19b, c, i, j), (Page 12–13, Lines 258–271; Page 13, Lines 278–282; Page 13-14, Lines 294–308).

11. The data show that Atg5 deficiency in DN ILC3s correlates with lipid accumulation, reduced glycolysis, and altered cytokine output. However, it remains unclear whether the metabolic reprogramming observed is the mechanistic driver of functional changes or a secondary effect of Atg5 loss. Rescue experiments that restore glycolytic flux (or alternatively suppress FAO) in Atg5-deficient DN ILC3s would provide stronger evidence of causality between metabolic state and pathogenic function.

Reply: We sincerely appreciate your insightful comments regarding the causal link between Atg5-mediated metabolic reprogramming and DN ILC3s. To determine whether metabolic remodeling drives functional changes or a secondary effect of *Atg5* loss, we have re-analyzed the metabolic profiles of our RNA-seq data and performed metabolic rescue experiments. The corresponding results are presented below.

- Re-analysis of scRNA-seq data from CON and NEC DN ILC3s showed

upregulation in glycolysis-related genes following NEC induction, which was validated using quantitative real-time PCR (qPCR) (Figure L a, b). In contrast, *Atg5*-deficient DN ILC3s exhibited downregulation of glycolysis-related genes and concurrent upregulation of fatty acid metabolism genes (Figure L c, d). These data indicate that *Atg5* loss in DN ILC3s drives a shift from glycolysis toward fatty acid metabolism, establishing metabolic reprogramming upon *Atg5* deficiency.

- Following NEC induction, Rorc^{cre}Atg5$^{fl/fl}$ mice exhibited a significant protective phenotype against NEC, which was completely abrogated by lactate or etomoxir treatment. Metabolic intervention exacerbated weight loss and NEC progression in knockout mice (Figure M a–c), accompanied by selective expansion of total ILC3s (especially the DN ILC3 subset) and significantly enhanced IL-17A and IL-22 production in both total ILC3s and DN ILC3s (Figure M d–c).

- To determine whether the observed metabolic alterations drive DN ILC3 functional changes or represent a secondary consequence of *Atg5* deficiency, we sorted DN ILC3s from Atg5$^{fl/fl}$ and Rorc^{cre}Atg5$^{fl/fl}$ mice and cultured these cells with DMSO, lactate, or etomoxir for 48 h (Figure N a). Both lactate and etomoxir treatments enhanced IL-17A and IL-22 production by DN ILC3s from Rorc^{cre}Atg5$^{fl/fl}$ mice (Figure N b, c).

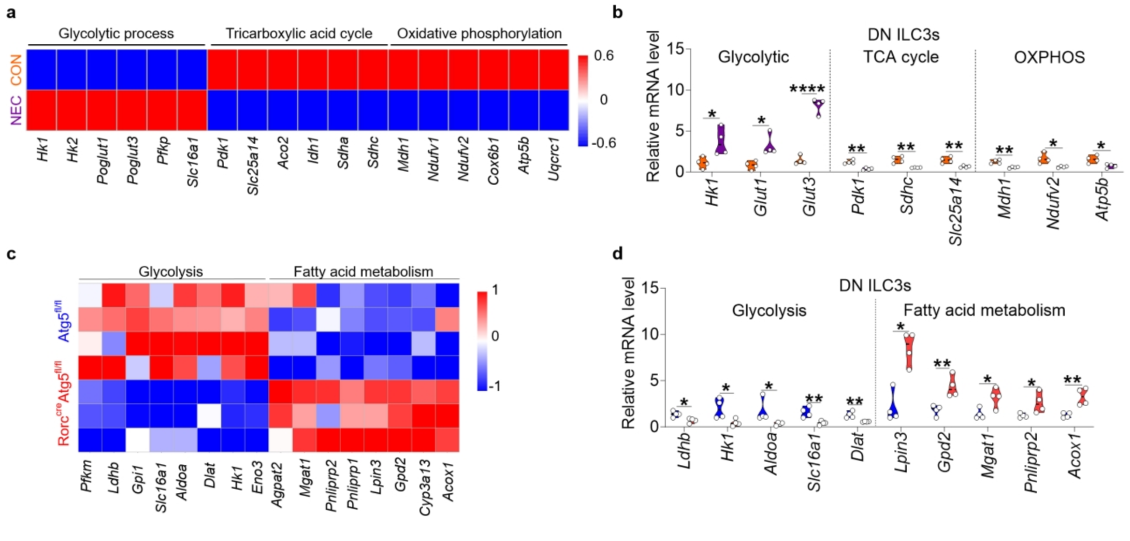

Figure L. *Atg5* deficiency in DN ILC3 induces glycolipid metabolic reprogramming. (**a**) Heatmap of glycolysis-related genes in DN ILC3s from CON and NEC mice. (**b**) Relative mRNA levels of the indicated genes in DN ILC3s ($n = 4$ biological replicates). (**c**) Heatmap of glycolysis- and fatty acid metabolism-related genes in DN ILC3s from Atg5$^{fl/fl}$ and Rorc^{cre}Atg5$^{fl/fl}$ mice. (**d**) Relative

mRNA levels of the indicated genes in DN ILC3s ($n = 4$ biological replicates). Each symbol represents one pup from a litter, and data are from at least three independent experiments. Error bars represent the SEM. *$p < 0.05$; **$p < 0.01$; ***$p < 0.001$; ns, not significant. Statistical significance was analyzed by Student's *t* test (**b** and **d**).

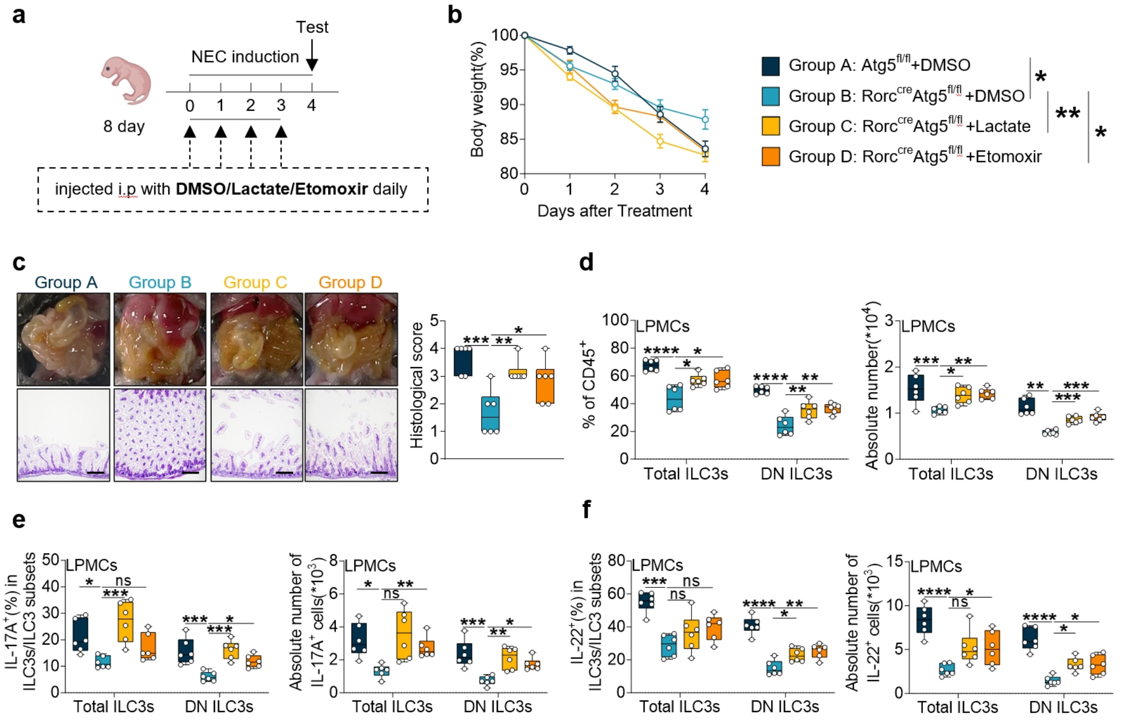

Figure M. Metabolic interventions aggravate NEC and activate ILC3 effector function in ILC3 autophagy-deficient neonatal mice. (**a**) Schematic diagram of metabolic interventions during NEC induction. (**b**) Body weight changes in the indicated groups ($n = 6$). (**c**) Macroscopic image and H&E staining of the intestines (scale bar: 100 μm), and inflammation scores were calculated ($n = 6$). (**d**) Percentage and absolute number of intestinal ILC3s and DN ILC3s ($n = 6$). (**e**) Percentage and absolute number of intestinal IL-17A^{+} ILC3s and IL-17A^{+} DN ILC3s ($n = 6$). (**f**) Percentage and absolute number of intestinal IL-22^{+} ILC3s and IL-22^{+} DN ILC3s ($n = 6$). Each symbol represents one pup from a litter, and data are from at least three independent experiments. Error bars represent the SEM. *$p < 0.05$; **$p < 0.01$; ***$p < 0.001$; ns, not significant. Statistical significance was analyzed by One-way ANOVA (**b**–**f**).

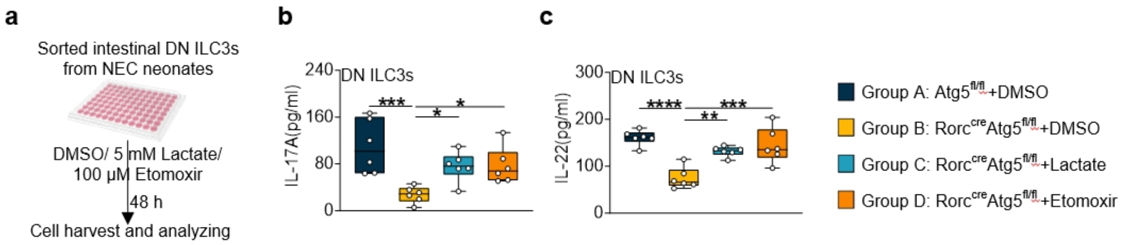

Figure N. Lactate or etomoxir treatment increases DN ILC3 function in vitro. (**a**) DN ILC3s were treated with dimethyl sulfoxide (DMSO), lactate, or etomoxir for 48 h. (**b, c**) Levels of IL-17A and

IL-22 in DN ILC3s following different treatments ($n = 6$ biological replicates). Each symbol represents one pup from a litter, and data are from at least three independent experiments. Error bars represent the SEM. *$p < 0.05$; **$p < 0.01$; ***$p < 0.001$; ns, not significant. Statistical significance was analyzed by One-way ANOVA (**b** and **c**).

These data demonstrate that *Atg5* deficiency drives metabolic reprogramming in DN ILC3s, and that this metabolic shift is a causal driver of their altered function.

The supplementary data and corresponding description have been incorporated into the revised version (Figure 5g, h; Figure 6 a–h; Supplementary Figure 21d, e), (Page 15, Lines 327–332; Page 15-16, Lines 338–346; Page 16, Lines 389–404).

12. Phosphatidylcholine supplementation is presented as therapeutic, but the mechanism remains speculative. Is PC acting directly on ILC3 metabolism, altering epithelial responses, modulating the microbiome, or acting through some other mechanism? No evidence distinguishes these possibilities.

Reply: We are grateful for your insightful comment regarding the mechanism of phosphatidylcholine (PC) supplementation. To explore the underlying effects of PC, we have performed additional experiments on DN ILC3 metabolism, IEC function, and the gut microbiome. The corresponding results are presented below.

- PC treatment significantly impaired glycolytic capacity in DN ILC3s (Figure O a), as further evidenced by reduced lactate production (Figure O b). These data indicate that PC directly regulates DN ILC3 metabolism.
- PC treatment did not alter the proportion or absolute number of IECs (Figure P a, b). However, it reduced IEC apoptosis rather than affecting proliferation (Figure P c, d). Moreover, PC treatment contributed to intestinal barrier repair, as evidenced by reduced FD7000 concentrations and upregulated tight junction protein expression (Figure P e, f). These findings demonstrate that PC protects the intestinal epithelium during NEC.
- Additionally, PC treatment induced gut microbiome remodeling (Figure Q a, b), with a significant increase in *Clostridia* abundance (Figure Q c, d). These observations were further confirmed by elevated *Clostridia* abundance in fecal samples (Figure Q e).

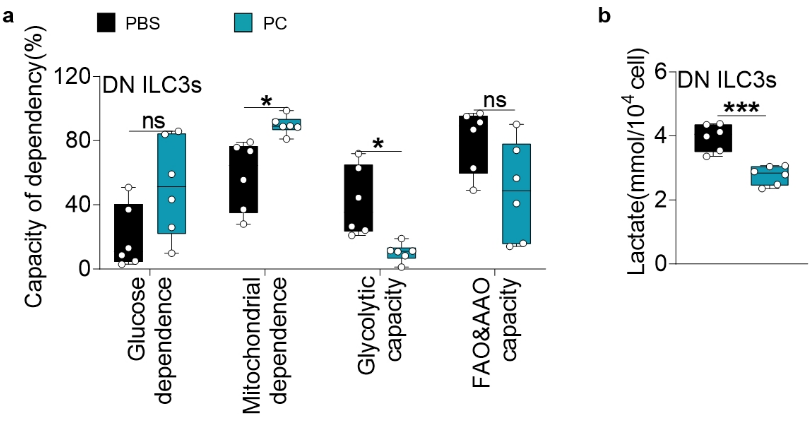

Figure O. PC directly regulates DN ILC3 metabolism. (**a**) Glucose dependence, mitochondrial dependence, glycolytic capacity, and FAO and AAO capacity in intestinal DN ILC3s ($n = 6$). (**b**) Lactate concentrations in DN ILC3s ($n = 6$). Each symbol represents one pup from a litter, and data are from at least three independent experiments. Error bars represent the SEM. $^*p < 0.05$; $^{**}p < 0.01$; $^{***}p < 0.001$; ns, not significant. Statistical significance was analyzed by Student's *t* test (**a** and **b**).

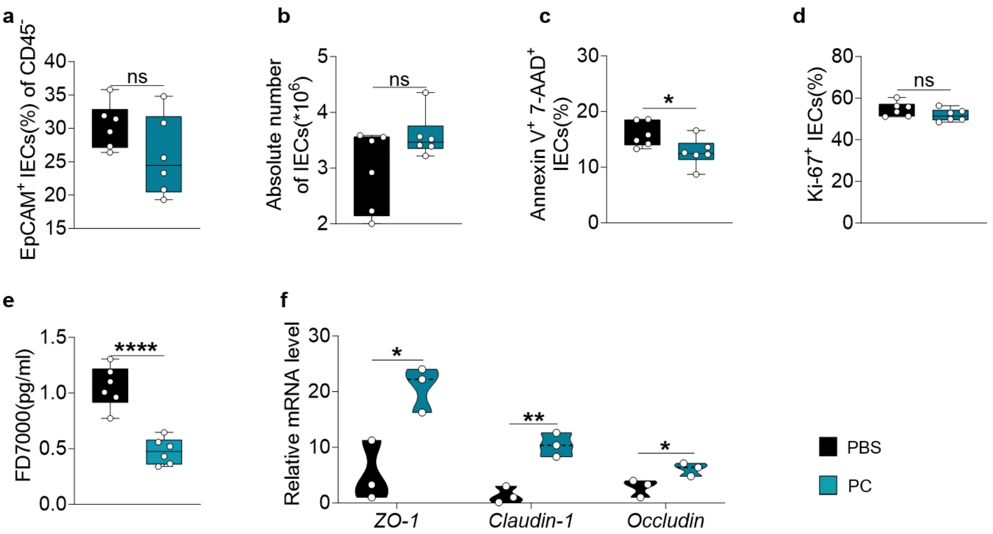

Figure P. PC directly enhances IEC function. (**a**, **b**) Percentage and absolute number of intestinal epithelial cells (IECs) ($n = 6$). (**c**, **d**) Statistical analysis of Annexin V^+ 7-AAD$^+$ and Ki67$^+$ staining in IECs ($n = 6$). (**e**) Intestinal permeability was assessed by plasma FD7000 levels ($n = 6$). (**f**) Relative mRNA levels of *ZO-1*, *Claudin-1*, and *Occludin* in the intestines ($n = 3$ biological replicates). Each symbol represents one pup from a litter, and data are from at least three independent experiments. Error bars represent the SEM. $^*p < 0.05$; $^{**}p < 0.01$; $^{***}p < 0.001$; ns, not significant. Statistical significance was analyzed by Student's *t* test **(a-f).**

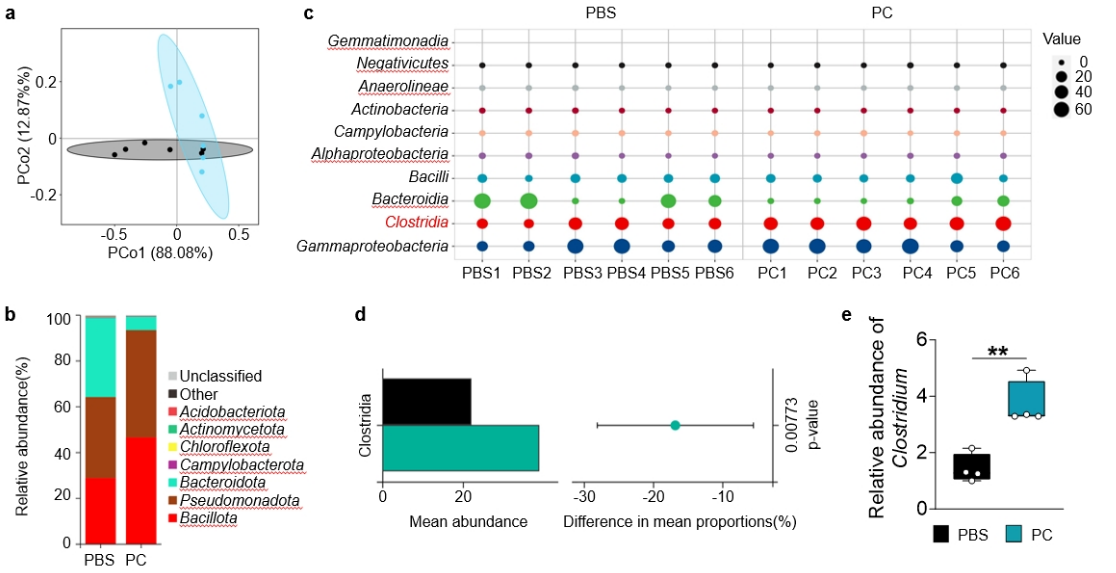

Figure Q. PC directly remodels the gut microbiome. (**a**) Principal component analysis of microbial community profiles. (**b**) Stacked bar chart illustrating the relative abundance of the top bacterial phyla. (**c**) Dot plot showing the relative abundance of the top 10 bacterial taxa at the order/class level. (**d**) Bar chart showing the mean relative abundance of *Clostridia*, with a forest plot displaying the difference in mean proportions ($n = 6$). (**e**) The relative abundance of *Clostridium* in fecal samples ($n = 4$ biological replicates). Each symbol represents one pup from a litter, and data are from at least three independent experiments. Error bars represent the SEM. *$p < 0.05$; **$p < 0.01$; ***$p < 0.001$; ns, not significant. Statistical significance was analyzed by Student's t test **(d** and **e).**

Collectively, these data demonstrate that PC acts through a multimodal mechanism, directly targeting DN ILC3 metabolism, enhancing IEC function, and promoting a protective gut microbiome.

The supplementary data and corresponding description have been incorporated into the revised version (Figure 7h–m; Supplementary Figure 29j–l; Supplementary Figure 30a–d), (Page 19, Lines 418–439).

13. While human NEC tissue data are valuable, further detail is needed on patient sample size, gestational age, and NEC severity for clinical relevance. It is also unclear whether autophagy upregulation is unique to ILC3s versus other immune subsets in the human NEC tissue. Expanding or clarifying this would strengthen the translational impact.

Reply: We sincerely appreciate your constructive recommendations. We fully agree that providing detailed clinical information and comparing autophagy activity across immune subsets would improve the clinical relevance. Accordingly, we have clarified

the patient sample details and re-analyzed the human scRNA-seq data. The corresponding results are presented below.

- Human samples were obtained from 9 controls and 7 infants with NEC, with gestational age ranging from 26 to 39 weeks and postnatal age ranging from 1 to 343 days. NEC samples were obtained during surgical resection for severe NEC (Bell stage: IIB to IIIB). Control samples were collected from age-matched infants undergoing surgery for other indications (e.g., stoma reanastomosis in the absence of active intestinal disease, with primary diagnoses including meconium plug/ileus, spontaneous intestinal perforation, Hirschsprung disease (unaffected tissue used), duodenal atresia, and intestinal pseudo-obstruction). The detailed information has been added to the Methods section and is also presented below Table 1.

- To determine autophagy activity in ILC3s and other immune subsets, we re-analyzed the REACTOME_AUTOPHAGY pathway activity (AUC score) across all major immune and non-immune cell subsets in human single-cell data. Autophagy was enriched in both immune and non-immune cells in the context of NEC. Notably, the autophagy score of ILC3s was significantly higher than that of other ILCs and T cells (Figure R a).

	Gestational age at birth (weeks+days)	Weight at birth (g)	Age at surgery (days)	Sex	Diagnosis
Control1	37+1	2200	17	Female	Hirschsprung disease
Control2	37+1	3000	17	Female	Hirschsprung disease
Control3	39	3200	87	Female	Hirschsprung disease
Control4	37	3300	343	Female	Hirschsprung disease
Control5	40	3300	14	Male	Intestinal Atresia
Control6	27	1150	159	Female	Ileostomy Closure
Control7	39	3170	117	Male	Hirschsprung disease
Control8	39	2750	207	Male	Congenital Anorectal Atresia
Control9	39	3580	60	Male	Hirschsprung disease
NEC1	26+4	800	27	Female	IIIB
NEC2	31	850	1	Female	IIIB
NEC3	27	1200	37	Female	IIB
NEC4	30+4	990	18	Male	IIIB
NEC5	37+5	3100	3	Female	IIB
NEC6	38+6	3200	1	Female	IIIB
NEC7	37+1	2740	45	Female	IIIB

Table 1. Clinical characteristics of human samples.

a

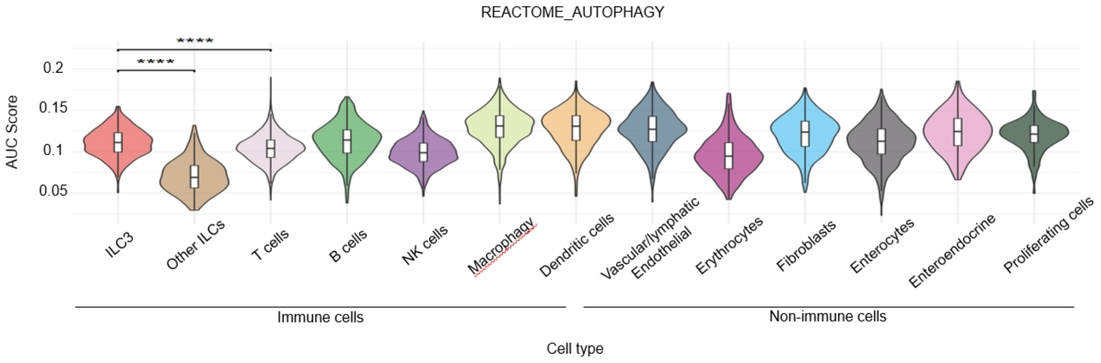

Figure R. ILC3s exhibit an enhanced autophagy activity. (**a**) Violin plot showing the activity of the REACTOME_AUTOPHAGY pathway (measured by AUC score) across distinct intestinal cell types. Statistical significance was analyzed by Tukey's multiple comparisons test (**a**). ****$p < 0.0001$.

These data demonstrate that autophagy is upregulated in human ILC3s from the NEC group.

The supplementary data and corresponding description have been incorporated into the revised version (Supplementary Figure 32a; Supplementary Table 1), (Page 21, Lines 466–470).

Discussion

14. Consider using schematic figures to illustrate the proposed autophagy-metabolism-DN ILC3-NEC axis.

Reply: We sincerely appreciate your constructive suggestion. We fully acknowledge that a schematic figure will enhance the clarity and readability of our work. Accordingly, we have added a working model illustrating the autophagy-metabolism-DN ILC3-NEC axis.

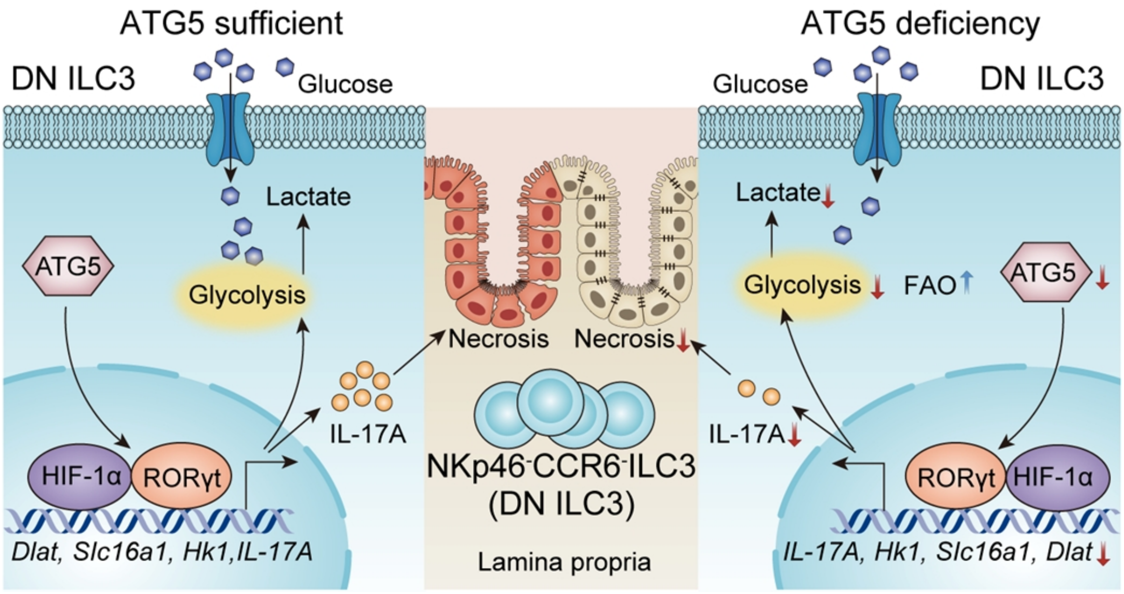

ATG5-mediated autophagy as a critical regulator of DN ILC3 pathogenicity in NEC. Mechanistically, ATG5 sustains the HIF-1α/RORγt transcriptional program, which drives glycolytic metabolism and IL-17A secretion in DN ILC3s. This metabolism promotes intestinal epithelial necrosis, exacerbating NEC severity. Conversely, *Atg5* deficiency blunts HIF-1α/RORγt activity, reducing glycolysis and enhancing FAO, thereby dampening IL-17A production and mitigating intestinal damage.

The working model and corresponding description have been incorporated into the revised version (Supplementary Figure 34), (Page 21–22, Lines 477–484).

15. While Rag2 KO adoptive transfer experiments are informative, differences from previous studies (e.g., CD4+ T cell contribution) are attributed to cell number and model differences. It may strengthen the discussion to include quantitative data or additional controls showing that DN ILC3-mediated inflammation is not influenced by other adaptive immune cells.

16. The discussion overall downplays the inconsistencies with prior literature regarding T cell contributions. Simply attributing discrepancies to “differences in model construction” is insufficient. Direct comparative data or stronger rationale is required.

Reply: We thank our reviewer for these insightful comments. We fully agree that the role of T cells in NEC is well-established. In response, we have compared our data with published reports and conducted adoptive transfer experiments to further support our conclusions. The corresponding results are presented below.

- Firstly, NEC induction in both *Rag2*$^{-/-}$ and wild-type littermates resulted in comparable disease severity (Figure S a–d), with no significant differences in the

frequencies or absolute numbers of total ILC3s, DN ILC3s, $CCR6^+$ ILC3s, $NKp46^+$ ILC3s, or IL-$17A^+$ and IL-22^+ ILC3 subsets (Figure S e–j). This finding contrasts with a previous report showing that $Rag1^{-/-}$mice were resistant to NEC induction, pointing to potential functional differences between the two models. Re-examination of that study (*J Clin Invest*, 2016, dois:10.1172/JCI83356) revealed that, while a similar induction protocol was used, the key methodological distinction was the addition of an bacterial product cocktail derived from NEC patient intestinal contents. The lack of critical details—including donor patient severity, and the composition and dosage of the bacterial products—precludes direct cross-study comparison. Consistent with prior reports, we found that $CD4^-ROR\gamma^+$ cells (predominantly DN ILC3s) were more than 8-fold more abundant than $CD4^+ROR\gamma^+$ T cells in neonatal NEC. This marked numerical dominance, together with the unabated susceptibility of $Rag2^{-/-}$ mice to NEC, supports a predominant role for DN ILC3s in driving NEC pathogenesis in our model. The divergent outcomes between $Rag1^{-/-}$ and $Rag2^{-/-}$ models underscore the importance of model system considerations when interpreting immune contributions to this disease.

- To further clarify the functional roles of T cells and DN ILC3s, we performed adoptive transfer experiments in *Rag2*$^{-/-}$neonates. Recipients were injected with PBS, 3×10^4 DN ILC3s, or 3×10^4 or 3×10^6 $CD4^+$ T cells (Figure T a). Transfer of DN ILC3s significantly exacerbated weight loss and intestinal inflammation, whereas $CD4^+$ T cells achieved comparable effects only at a 100-fold higher dose (Figure T b–d). This dose dependency may reflect opposing T cell subset functions, as previously reported (*J Clin Invest*, 2016, dois:10.1172/JCI83356) that the NEC environment suppresses regulatory T cells while expanding pathogenic Th17 cells, suggesting a functional equilibrium. At low cell numbers, these opposing forces may counterbalance, yielding no net effect; at high numbers, however, the equilibrium may shift toward pathogenicity. These findings establish a functional hierarchy: while T cells can contribute at high numbers, DN ILC3s serve as key early drivers of NEC pathogenesis.

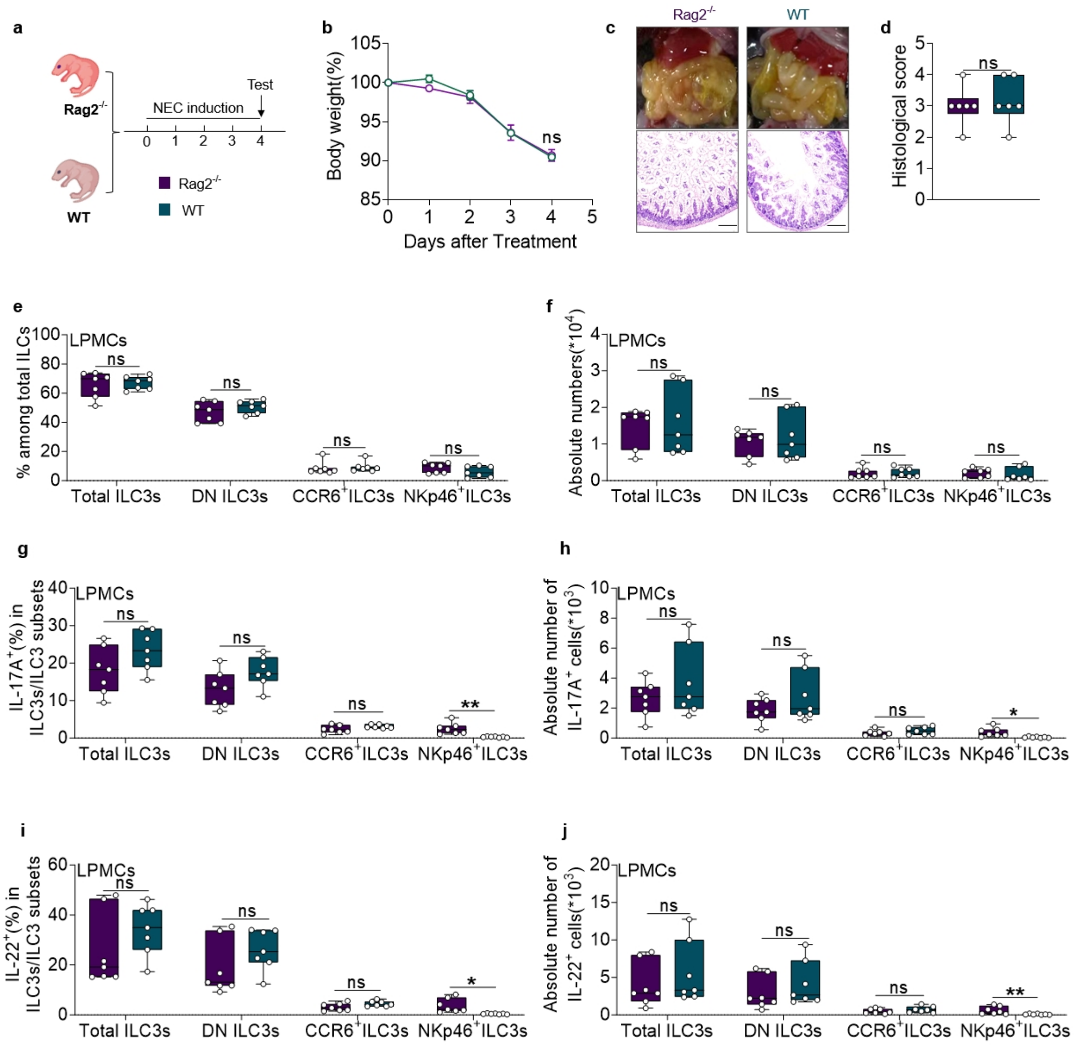

Figure S. Adaptive immune deficiency does not alter disease outcomes following NEC induction. (**a**) Experimental NEC was induced in Rag2$^{-/-}$ and wild-type neonates. (**b**) Body weight changes in the indicated groups (n = 7). (**c**) Macroscopic image and H&E staining of the intestines (scale bar: 100 μm). (**d**) Inflammation scores were calculated (n = 7). (**e, f**) Percentage and absolute number of intestinal ILC3s and ILC3 subsets (n = 7). (**g-j**) Percentage and absolute number of IL17A$^+$ or IL-22$^+$ ILC3s and ILC3 subsets (n = 7). Each symbol represents one pup from a litter, and data are from at least three independent experiments. Error bars represent the SEM. *$p < 0.05$; **$p < 0.01$; ***$p < 0.001$; ns, not significant. Statistical significance was analyzed by Student's t test (**b** and **d-j**).

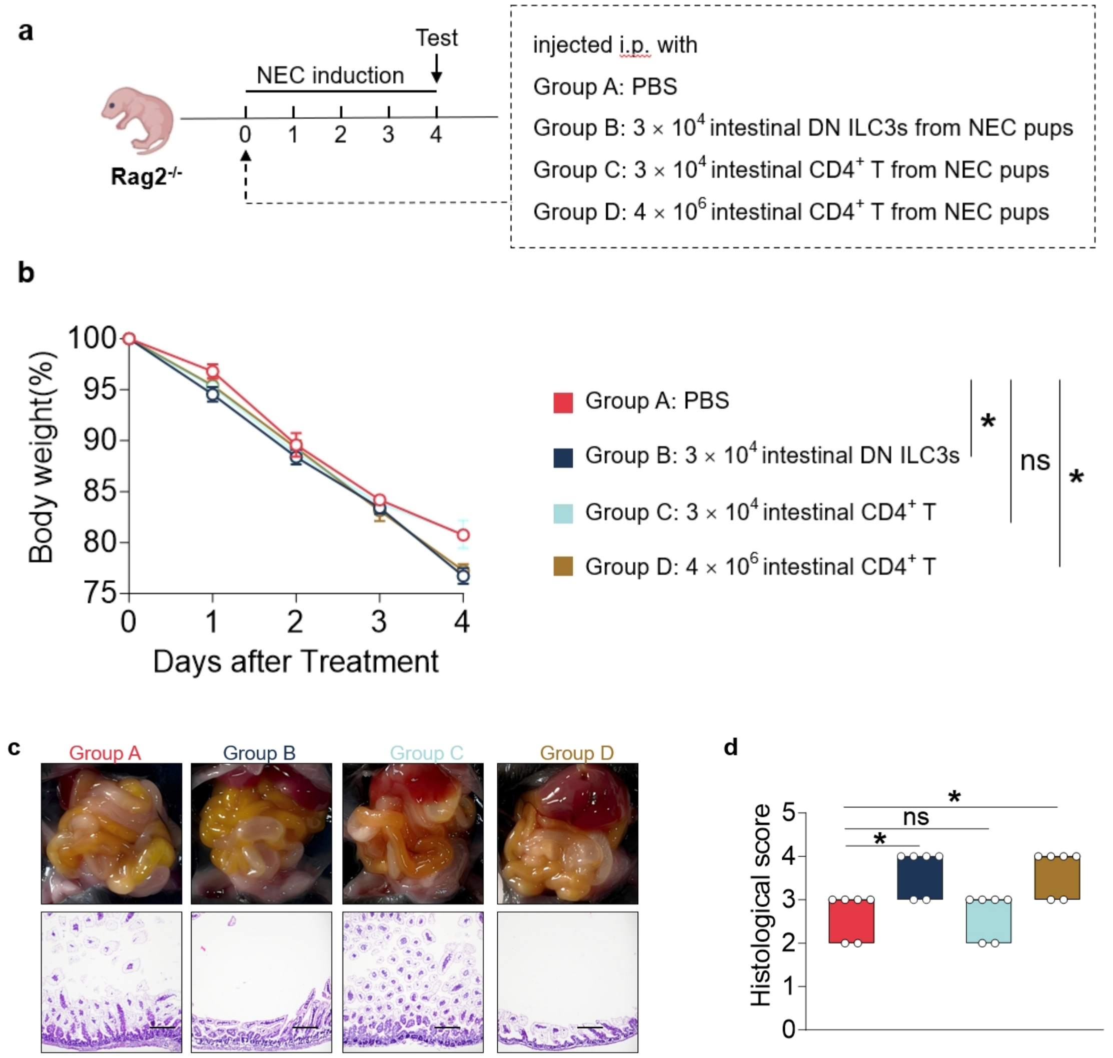

Figure T. Adoptive transfer of DN ILC3s or high number of CD4+ T cells exacerbates NEC in Rag2–/– neonates. (**a**) Schematic diagram of adoptive cell transfer experiments in Rag2–/– neonates. (**b**) Body weight changes in the indicated groups (n = 6). (**c**) Macroscopic image and H&E staining of the intestines (scale bar: 100 μm). (**d**) Inflammation scores were calculated (n = 6). Each symbol represents one pup from a litter, and data are from at least three independent experiments. Error bars represent the SEM. *$p < 0.05$; **$p < 0.01$; ***$p < 0.001$; ns, not significant. Statistical significance was analyzed by One-way ANOVA (**b** and **d**).

Collectively, these findings support our conclusion that intestinal DN ILC3s play a critical role in NEC. While T cells contribute to disease progression under certain conditions, their requirement at a markedly higher number suggests that DN ILC3s are the primary drivers in this model. Based on these findings, we have revised the Discussion section accordingly (Page 23, Lines 501–514).

The supplementary data and corresponding description have been incorporated into the revised version (Supplementary Figure 5a–f; Supplementary Figure 10a–c), (Page 8,

Lines 153–160; Page 8, 206–214).

17. The discussion highlights alterations in glycolysis, fatty acid oxidation, and glycerophospholipid metabolism. While intriguing, the mechanisms connecting PC supplementation to ILC3 functional changes are not fully defined. A more detailed mechanistic hypothesis, or acknowledgment of knowledge gaps, would be valuable for readers.

Reply: We sincerely appreciate your insightful viewpoint. We fully acknowledge that clarifying the mechanisms linking PC supplementation to ILC3 function would address this knowledge gap and benefit readers. Therefore, we have analyzed metabolic changes in DN ILC3s following PC treatment and performed *in vitro* experiments to assess its functional effects. Moreover, we have revised the Discussion section based on these new observations. The corresponding results are presented below.

- Following PC supplementation, DN ILC3s exhibited metabolic reprogramming characterized by inhibited glycolysis, as evidenced by reduced lactate production (Figure U a, b).
- To assess the direct effect of PC on DN ILC3s, we established an *in vitro* culture system (Figure V a). PC supplementation significantly reduced IL-17A and IL-22 production by DN ILC3s (Figure V b, c).These data demonstrate that PC directly inhibits glycolysis and suppresses DN ILC3s function.
- Based on above findings, we have revised the Discussion accordingly: Transcriptomic and metabolomic analyses revealed PC as a key downstream metabolite of Atg5 mediated autophagy, further indicated that PC s supplementation inhibits DN ILC3 function, restored glycolytic homeostasis, reshaped microbiota and ameliorated NEC pathology. These findings suggest PC might be a key downstream effector of the DN ILC3 autophagy–metabolism axis and a promising therapeutic avenue for NEC. Identifying the precise molecular targets and underlying mechanisms through which PC acts on DN ILC3s will be a central focus of future research.

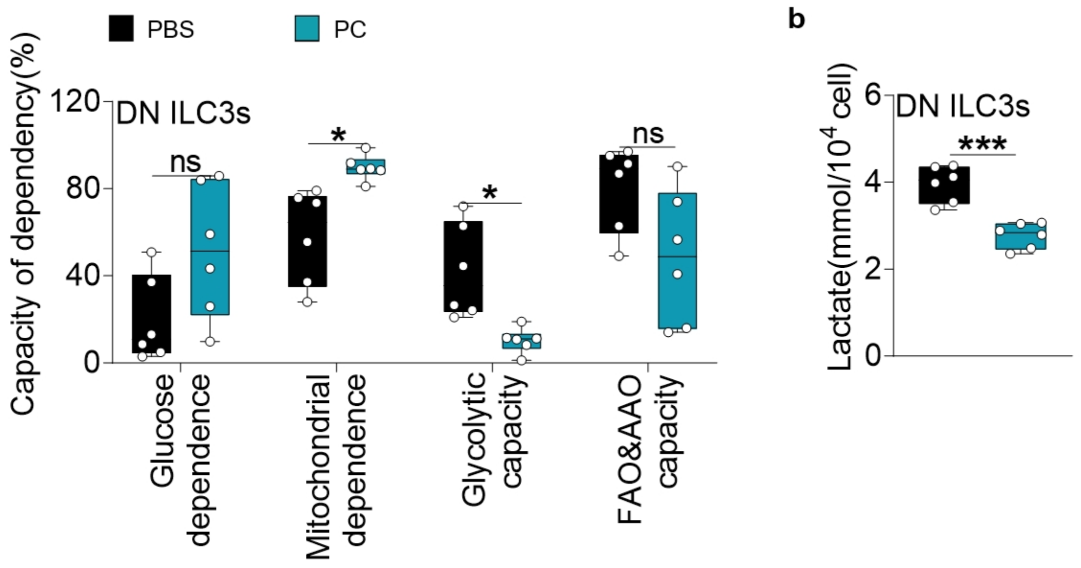

Figure U. PC treatment suppresses glycolysis and reduces lactate levels in DN ILC3s. (**a**) Glucose dependence, mitochondrial dependence, glycolytic capacity, and FAO and AAO capacity in intestinal DN ILC3s ($n = 6$) . (**b**) Lactate concentrations in intestinal DN ILC3s ($n = 6$). Each symbol represents one pup from a litter, and data are from at least three independent experiments. Error bars represent the SEM. *$p < 0.05$; **$p < 0.01$; ***$p < 0.001$; ns, not significant. Statistical significance was analyzed by Student's *t* test (**a** and **b**).

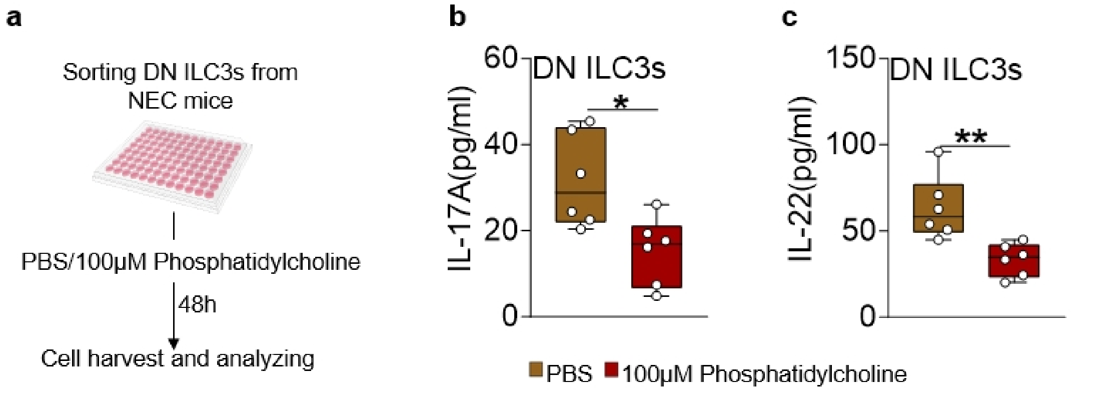

Figure V. PC treatment reduces DN ILC3 function in vitro. (**a**) DN ILC3s were treated with PBS or PC for 48 h. (**b, c**) Levels of IL-17A and IL-22 in DN ILC3s following different treatments ($n = 6$ biological replicates). Each symbol represents one pup from a litter, and data are from at least three independent experiments. Error bars represent the SEM. *$p < 0.05$; **$p < 0.01$; ***$p < 0.001$; ns, not significant. Statistical significance was analyzed by Student's *t* test (**b** and **c**).

The supplementary data and corresponding description have been incorporated into the revised version (Figure 7h, i, n, o),(Page 19, Lines 428–430; Page 20, Lines 441–443; Page 25, Lines 563–569).

18. The study proposes targeting the autophagy-energy metabolism axis as a therapeutic strategy for NEC. Consider discussing whether this axis is truly specific to NEC pathophysiology.

Reply: We sincerely thank the reviewer for this insightful question regarding the specificity of the autophagy–energy metabolism axis in NEC. To address this issue, we have examined the expression of glycolysis- and fatty acid metabolism-related genes in *Atg5*-deficient DN ILC3s using NEC and *Citrobacter rodentium* mouse models. The corresponding results are presented below.

- ILC3-specific *Atg5* ablation induced reprogramming of glycolysis and fatty acid metabolism in DN ILC3s from NEC-induced mice, as evidenced by downregulation of glycolysis-related genes and upregulation of fatty acid metabolism-related genes. In contrast, no significant differences in these genes were observed in *Atg5*-deficient DN ILC3s from C.R.-infected mice (Figure W a, b).

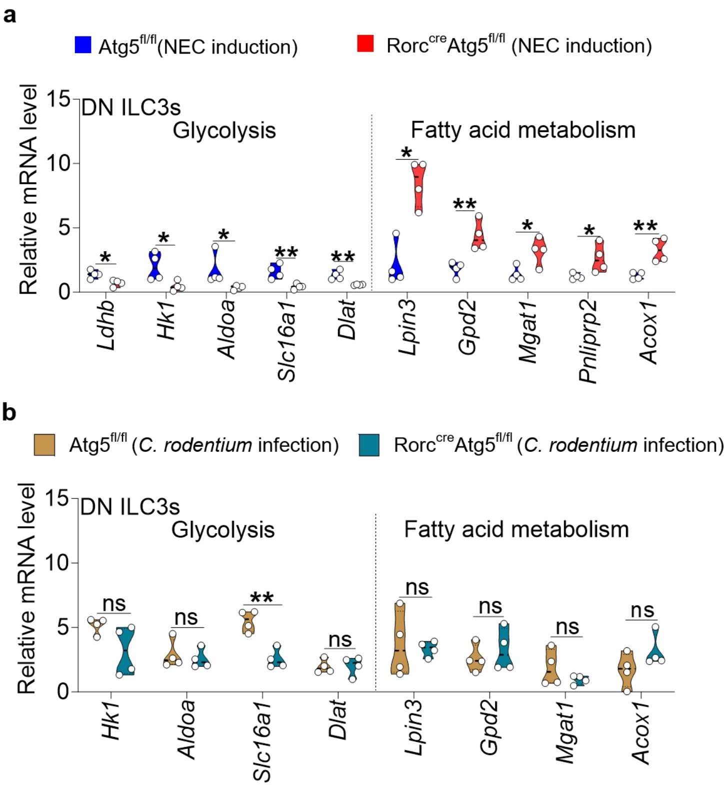

Figure W. Metabolic gene expression in DN ILC3s following NEC induction or C.R. infection. (**a**) Relative mRNA levels of glycolysis- and fatty acid metabolism-related genes in DN ILC3s following NEC induction ($n = 4$ biological replicates). (**b**) Relative mRNA levels of the indicated genes in DN ILC3s following *Citrobacter Rodentium* infection ($n = 4$ biological replicates). Each

symbol represents one pup from a litter, and data are from at least three independent experiments. Error bars represent the SEM. $*p < 0.05$; $**p < 0.01$; $***p < 0.001$; ns, not significant. Statistical significance was analyzed by Student's *t* test (**a** and **b**).

These data demonstrate that the metabolic reprogramming of DN ILC3s in NEC is disease-specific, as evidenced by distinct metabolic signatures compared to C. rodentium infection. We also discussed this point accordingly: Notably, this autophagy–metabolism axis is NEC-specific (it is absent in IBD models), highlighting a critical, context-dependent role for autophagy–energy metabolism crosstalk in regulating DN ILC3 function in the context of NEC.

The supplementary data and corresponding description have been incorporated into the revised version (Figure 5h; Supplementary Figure 25a), (Page 15, Lines 329–332; Page 17, Lines 374-379; Page 25, Lines 555–562).

19. The potential translational challenges, including specificity, timing, and safety of manipulating autophagy or metabolism to "regulate intestinal homeostasis", as the authors suggest, needs to be addressed, especially in physiologically vulnerable neonates. The translational leap from murine NEC to neonatal therapy is considerable. The discussion should grapple more seriously with challenges of manipulating autophagy or metabolism.

Reply: We sincerely appreciate your thoughtful feedback. We fully acknowledge that a more serious engagement with the translational challenges is critical to contextualizing our findings. Thus, we have revised the Discussion section to address this. The specific revisions are presented below.

Targeting the autophagy–metabolism axis—via autophagy modulation, metabolic intervention, or PC supplementation—holds promise for NEC. However, key translational challenges remain, including achieving cell-type-specific modulation, defining the optimal therapeutic window, and ensuring safety in neonates. Moreover, the large gap between murine models and human newborns demands rigorous evaluation of efficacy and toxicity in clinically relevant settings. Humanized mouse models and patient-derived intestinal organoids offer powerful platforms to bridge this gap and advance precision therapeutics for NEC.

The corresponding description have been incorporated into the revised version (Page 26, Lines 579–585).

Minor comments

20.There is an extra comma in line 76 of the introduction: "CCR6+ILC3s are enriched in adult mice guts, providing an innate source of IL-17A, IL-17F, IL-22, and GM-CSF resulting from pathogen infection and IL-23 stimulation."

Reply: We sincerely appreciate your careful reading and apologize for this error. We have corrected the extra comma in the sentence as follows:

$CCR6^+$ ILC3s, encompassing fetal lymphoid tissue inducer cells and adult lymphoid tissue inducer-like cells, contribute to lymphoid tissue formation and provide an innate source of IL-17A, IL-17F, IL-22, and GM-CSF upon stimulation.

Additionally, we have thoroughly reviewed the entire manuscript to ensure no other similar punctuation or grammatical errors remain.

21.The manuscript would benefit from careful language editing for readability (e.g., occasional run-on sentences).

Reply: We are grateful for your thoughtful comments and apologize for the language issues in our previous version, including run-on sentences. As per your recommendation, the manuscript has been professionally edited by Editage (www.editage.cn) to improve its readability and overall language quality. We believe these revisions would enhance the clarity and flow of the text.

Overall Assessment:

This is a well-designed study with a potential to advance our understanding of NEC pathophysiology, adding on the current literature of ILC3s in NEC. The integration of scRNA-seq, genetic models, metabolomics, and human validation is sound. However, the current data fall short of Nature Communications standards due to concerns about novelty, experimental rigor, mechanistic clarity, and translational depth. Substantial revisions—particularly more rigorous cell validation, functional comparisons across ILC3 subsets, mechanistic rescue experiments, and clearer human clinical data—would be necessary before the work is suitable for publication. Addressing these points may require additional analyses, particularly to substantiate the mechanistic findings. Additionally, including a schematic figure to illustrate the proposed mechanism would greatly enhance clarity and accessibility of the work.

Reply: We sincerely thank our reviewers for their comprehensive and constructive evaluation of our manuscript. We greatly appreciate the positive recognition of our study's design and its potential to advance the understanding of NEC pathogenesis, particularly regarding DN ILC3s. We have taken all concerns very seriously and have substantially addressed each point according to your recommendation. We hope our new data and additional revisions now meet your expectations and earn your approval.

Response to Reviewer #4

I co-reviewed this manuscript with one of the reviewers who provided the listed reports. This is part of the Nature Communications initiative to facilitate training in peer review and to provide appropriate recognition for Early Career Researchers who co-review manuscripts.

Reply: We greatly appreciate your thoughtful evaluation, critical feedback, and constructive suggestions, which have been highly valuable in improving the quality and rigor of our study. We have carefully addressed all of the concerns raised in your co-review and have incorporated substantial new experimental data and corresponding revisions into the revised manuscript. We hope that the additional data and revisions meet your expectations and earn your approval.

Response to Reviewer #5:

We sincerely appreciate the time and effort you have dedicated to reviewing our manuscript. Your valuable feedback and suggestions have significantly improved the quality of our paper. In response, we have thoroughly revised the manuscript and incorporated substantial new experimental data to strengthen the credibility of our findings. All modifications in the revised manuscript have been **indicated in blue text** for your convenience. Please refer to the detailed, point-by-point response to each comment below. We sincerely hope the revised version meets your expectations and look forward to your approval.

In this study, He and colleagues propose that ILC3s play a pathogenic role in necrotizing enterocolitis (NEC) by secreting cytokines that exacerbate disease severity. Mechanistically, the authors demonstrate that this detrimental effect is linked to increased autophagy, which in turn alters the metabolic programming of ILC3s. Deletion of Atg5 in RORγt^{+} cells, reduces disease pathology during NEC, shifts ILC3 metabolism toward fatty acid utilization, and leads to the accumulation of phosphatidylcholine (PC). Finally, supplementation with PC alleviates NEC symptoms, suggesting that PC administration may represent a potential therapeutic intervention for neonatal NEC.

Overall, the study presents several interesting findings. However, a number of important points remain unclear:

Reply: We are grateful for your constructive and insightful comments.

1. ILC2 involvement: In the scRNA-seq data shown in Fig. 1, there appears to be not only an increase in ILC3s but also a notable decrease in ILC2s. Do ILC2s play any role in NEC? Would adoptive transfer of ILC2s from adult mice in the NEC setting improve or worsen disease outcomes?

Reply: We sincerely appreciate your valuable comment regarding the potential involvement of ILC2s in NEC. To determine their functional role in disease development, we analyzed ILC2 alterations under NEC conditions and performed adoptive transfer experiments using both neonatal and adult ILC2s. The corresponding results are presented below.

- Our scRNA-seq data revealed reduced ILC2s in NEC-induced neonatal mice (Figure A a). Flow cytometry analysis further confirmed that the frequency of ILC2s was decreased after NEC induction, while their absolute numbers were comparable between groups (Figure A b-e).

- Adoptive transfer of ILC2s from neonatal (1-week-old) or adult (8-week-old) mice was performed to assess ILC2 function in NEC (Figure B a). Neonatal ILC2 transfer significantly reduced weight loss, intestinal inflammation and overall NEC severity, while adult ILC2s had no significant disease-modifying effect (Figure B b–d). These data demonstrate that neonatal ILC2s play a protective role in NEC.
- Based on these findings, we have revised the Discussion accordingly. The specific revisions are presented below.

Given the well-established anti-inflammatory roles of ILC2s in mucosal immunity, we further tested their function in our model. Adoptive transfer experiments demonstrated that intestinal ILC2s from neonatal mice—but not adult mice—significantly alleviated NEC. The detailed mechanism underlying this phenomenon will be the focus of future research by our group.

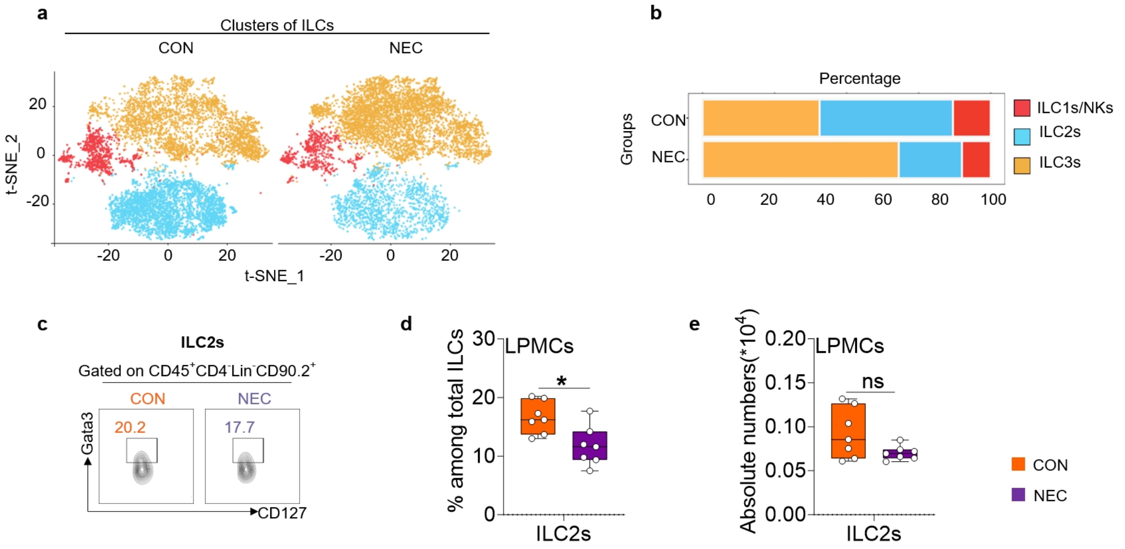

Figure A. The alterations of ILC2s following NEC induction. (**a**) t-Distributed Stochastic Neighbor Embedding (t-SNE) analysis comparing innate lymphoid cells (ILCs) clusters in the intestines of CON and NEC neonatal mice. (**b**) The median percentage of each ILC cluster, color-coded to match the populations in (a). (**c**) Representative flow cytometry profiles of intestinal ILC2s. (**d**, **e**) Percentage and absolute number of intestinal ILC2s ($n = 7$). Each symbol represents one pup from a litter, and data are from at least three independent experiments. Error bars represent the SEM. $*p < 0.05$; $**p < 0.01$; $***p < 0.001$; ns, not significant. Statistical significance was analyzed by Student's *t* test (**d** and **e**).

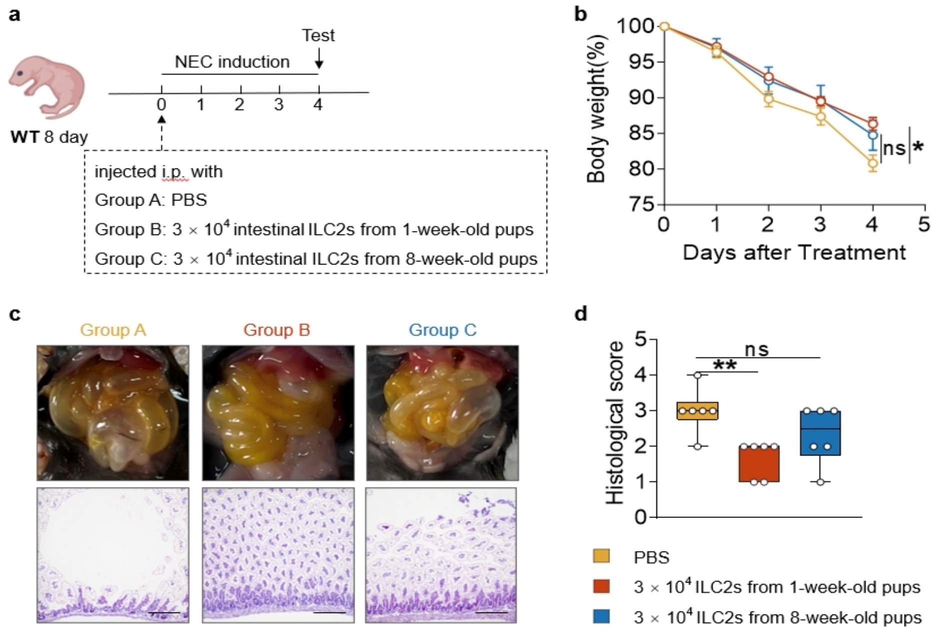

Figure B. Adoptive transfer of neonatal ILC2s under physiological conditions alleviates NEC severity. (**a**) Schematic diagram of adoptive transfer of ILC2s prior to NEC induction. (**b**) Body weight changes in the indicated groups (n = 6). (**c**) Macroscopic image and representative hematoxylin and eosin (H&E) staining of the intestines (scale bar: 100μm). (**d**) Inflammation scores were calculated based on the severity of intestinal inflammation (n = 6). Each symbol represents one pup from a litter, and data are from at least three independent experiments. Error bars represent the SEM. $*p < 0.05$; $**p < 0.01$; $***p < 0.001$; ns, not significant. Statistical significance was analyzed by One-way ANOVA (**b** and **d**).

These data demonstrate that neonatal ILC2s play a protective role in NEC.

The supplementary data and corresponding description have been incorporated into the revised version (Figure 1a–c; Supplementary Figure 9a–c), (Page 7, Lines 132–140; Page 9-10, Lines 200–205).

2. ILC3 subset interpretation: The authors focus on double-negative (DN) ILC3s and emphasize their specific role in NEC. However, prior studies have shown that DN ILC3s are precursors of NKp46$^+$, T-bet–dependent ILC3s. Because NKp46$^+$ILC3s begin to expand around day 15 of life, it is possible that at day 8 the ILC3 compartment consists predominantly of DN ILC3s, and the observed phenomena may reflect ILC3 biology in general rather than a DN-specific effect. This possibility should be carefully considered and discussed.

Reply: We sincerely appreciate your insightful comment. As previous studies have documented phenotypic conversion between ILC subsets (*Nat Commun*, 2024, 10.1038/s41467-024-52155-2), we acknowledge that our initial submission only included data from 1-week-old mice and did not assess potential conversion among the

three ILC3 populations. Therefore, we have now analyzed the proportions of these subsets at 1, 2, and 6 weeks of age as detailed below.

To determine whether the ILC3 subsets, including DN ILC3s, $CCR6^+$ ILC3s, and $NKp46^+$ ILC3s, undergo interconversion with age, we examined their frequencies at 1 week (neonatal), 2 weeks, and 6 weeks (adult). Flow cytometry analysis showed that DN ILC3s remained the predominant subset across all time points examined, with no significant reduction with age. In contrast, $NKp46^+$ ILC3s increased in adult mice and $CCR6^+$ ILC3s decreased, compared to the neonatal period (Figure C a, b).

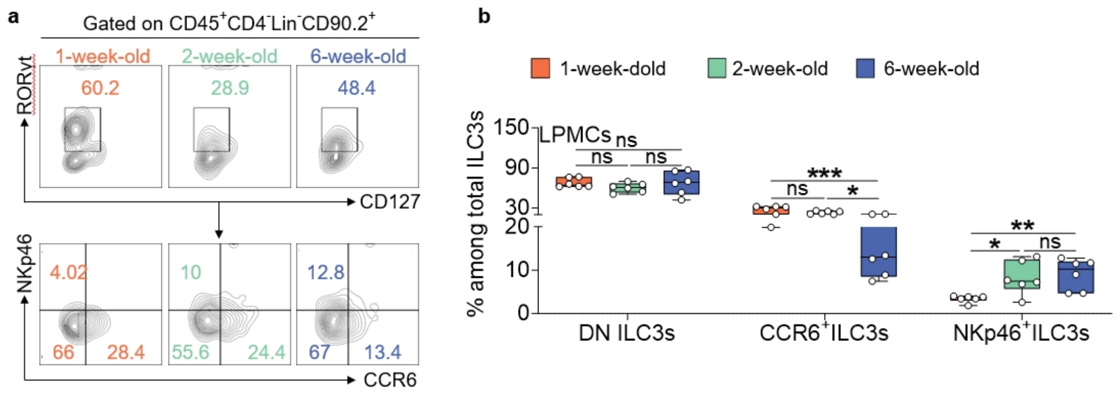

Figure C. Frequencies of ILC3 subsets at different time points. (**a**) Representative flow cytometry profiles of DN ILC3s, $CCR6^+$ ILC3s, and $NKp46^+$ ILC3s. (**b**) Percentage of DN ILC3s, $CCR6^+$ ILC3s, and $NKp46^+$ ILC3s (n = 6). Each symbol represents one pup from a litter, and data are from at least three independent experiments. Error bars represent the SEM. $^*p < 0.05$; $^{**}p < 0.01$; $^{***}p < 0.001$; ns, not significant. Statistical significance was analyzed by Student's t test (**b**).

These data indicate that the three ILC3 subsets do not undergo substantial interconversion with age.

The supplementary data and corresponding description have been incorporated into the revised version (Supplementary Figure 6a, b), (Page 8, Lines 168–171).

3. Gating strategy in Fig. 1c: It is unclear why the authors gated on $CD45^+Lin^-CD4^-CD90.2^+$ cells. If anti-CD3 is included in the lineage cocktail, excluding $CD4^+$cells would inadvertently remove $CCR6^+$LTi-like ILC3s, which could substantially affect downstream analyses and interpretation.

Reply: We thank the reviewer for this thoughtful comment. As the reviewer rightly points out, our lineage cocktail included an anti-CD3 antibody, which does exclude $CD4^+$ cells, including potential $CD4^+$ ILC3 subsets. We have thoughtfully considered this important point regarding our gating strategy. While we acknowledge this as a

limitation, several lines of evidence support that our main conclusions regarding the identified DN ILC3 subset remain valid:

- Egan et al. (*J Clin Invest*, 2016, dois:10.1172/JCI83356) report focused on the pathogenic role of $CD4^+$ T cells, and its own flow cytometric data showed that $CD4^-ROR\gamma t^+$ cells are over 8-fold more abundant than $CD4^+ROR\gamma t^+$ cells in the neonatal intestine, strongly suggesting an important, understudied role for $CD4^-ROR\gamma t^+$ cells in neonatal intestinal inflammation.
- Several studies defining ILC3s in NEC mouse models also use lineage cocktails containing anti-CD3 and anti-CD4. For example, Cho S.X., et al., (*Nat. Commun.*, 2020, 10.1038/s41467-020-19400-w) defined ILC3s as $CD4^-ROR\gamma t^+NKp46^+$ or $NKp46^-ROR\gamma t^+Tbet^-$, with lineage markers including CD3. Zhang Y., et al., a defined ILC3s as $CD45^+Lin^-CD90.2^+CD127^+KLRG1^-ROR\gamma t^+$, with lineage markers including CD3 and CD4. Thus, our gating strategy is consistent with these published approaches.
- To directly test whether excluding $CD4^+$ cells affect our conclusions, we performed adoptive transfer experiments comparing DN ILC3s and $CD4^+$ T cells in $Rag2^{-/-}$ recipients (Figure D a). Transfer of 3×10^4 DN ILC3s significantly exacerbated NEC severity, whereas the same number of $CD4^+$ T cells had no effect; a 100-fold higher dose of $CD4^+$ T cells was required to achieve comparable phenotypes (Figure D b–d).

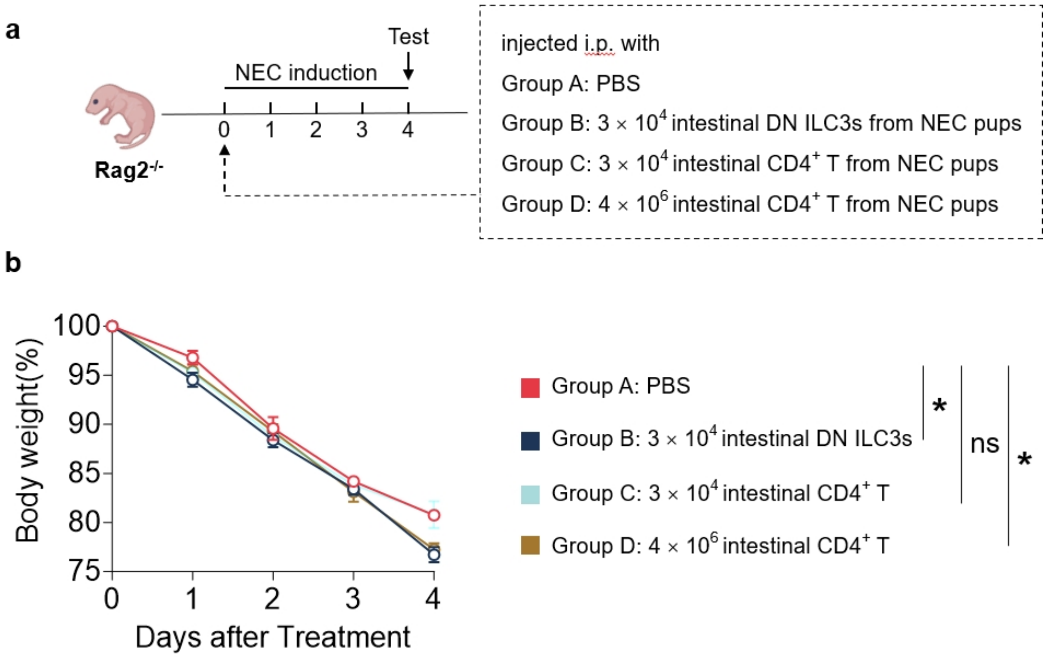

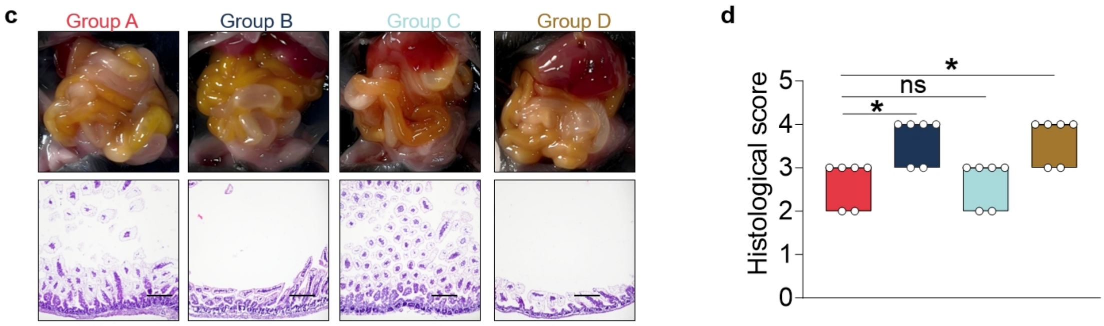

Figure D. Adoptive transfer of DN ILC3s exacerbates NEC severity. (**a**) Schematic diagram of adoptive transfer of $CD4^+$ cells and DN ILC3s prior to NEC induction. (**b**) Body weight changes in the indicated groups ($n = 6$). (**c**) Macroscopic image and representative H&E staining of the intestines (scale bar: 100 μm). (**d**) Statistical analysis of inflammation scores ($n = 6$). Each symbol represents one pup from a litter, and data are from at least three independent experiments. Error bars represent the SEM. $*p < 0.05$; $**p < 0.01$; $***p < 0.001$; ns, not significant. Statistical significance was analyzed by One-way ANOVA (**b** and **d**).

These data demonstrate that the pathogenic contribution of $CD4^+$ cells in NEC is negligible compared to DN ILC3s, further validating that excluding $CD4^+$ cells from our ILC3 gate does not impact our core conclusions.

The supplementary data and corresponding description have been incorporated into the revised version (Supplementary Figure 10a-c), (Page 10, Lines 206-213).

4. Concerns about ILC gating (Supplementary Fig. 1d): The ILC gating strategy raises concerns, as more than 50% of the $CD45^+$cells appear to be $CD90.2^+$—a surprisingly high proportion. This would only be plausible if the samples were pre-enriched using the Stemcell Pan-ILC kit, which should be clearly stated in the figure legend.

Reply 4: We sincerely appreciate your careful reading and apologize for not clearly describing the sample processing workflow in the figure legend. The samples were pre-enriched using a Pan-ILC enrichment kit (Stemcell, USA) to deplete lineage-positive hematopoietic and non-hematopoietic cells while specifically retaining $CD90.2^+$ ILCs.

We have revised the corresponding figure legend and to clearly describe this pre-enrichment procedure (Supplementary Figure 2a).

5. Contradictory findings regarding IL-22: A recent study (Zhang et al., Immunity, 2025) demonstrated that ILC3-derived IL-22 is protective in NEC. In contrast, the present work suggests a detrimental role. Cytokine-blocking experiments targeting IL-22, IL-17, and/or GM-CSF should be performed to distinguish which ILC3-derived cytokines are pathogenic versus beneficial.

Reply: We sincerely appreciate your careful review and insightful comment. We fully agree that clarifying the functional roles of IL-17A, IL-22, and GM-CSF in NEC is essential. To address this issue, we have performed *in vivo* neutralizing antibody experiments corresponding to these cytokines. The corresponding results are presented below.

- To define the specific roles of IL-17A, IL-22, and GM-CSF in NEC pathogenesis, we treated 8-day-old neonatal pups with individual neutralizing antibodies against each cytokine. First, we intraperitoneally injected anti-IL-17A or IgG isotype prior to NEC induction (Figure E a). Anti-IL-17A treatment significantly preserved body weight, and attenuated intestinal inflammation and barrier permeability (Figure E, b–e).This treatment also increased the frequency and absolute number of intestinal epithelial cells (IECs), accompanied by reduced IEC death and enhanced cell proliferation (Figure E, f–i).Consistently, anti-IL-17A treatment upregulated the expression of *ZO-1*, *Claudin-1* and *Occludin* (Figure E, j).
- Anti-IgG or anti-IL-22 was administered following the same regimen before NEC induction (Figure F a). However, anti-IL-22 treatment had no significant effect on weight change, intestinal inflammation, or intestinal permeability (Figure F b–e). Likewise, it did not alter IEC abundance, viability, proliferation, or tight junction protein expression (Figure F f–j).
- Consistent with anti-IL-22 treatment, anti-GM-CSF treatment also had no significant effect on weight change, intestinal inflammation, or intestinal permeability (Figure G a–e). Additionally, IEC levels, viability, and proliferation, as well as tight junction protein expression, were comparable between groups (Figure G f–j).

Collectively, these data demonstrate that IL-17A plays a pathogenic role in NEC, whereas IL-22 and GM-CSF have no effect.

- The role of IL-22 in our findings differs from its protective role reported by Zhang Y., et al., (*Immunity*, 2025, 10.1016/j.immuni.2025.02.003). Based on these observations, we have discussed the discrepancy in the revised Discussion, as follows:

The discrepancy is likely due to the differing frequency of antibody application: they

administered anti-IL-22 antibody continuously over four days, whereas we used two injections at the same dose, in line with our published protocol (*Nature Medicine*, 2018, 10.1038/nm.4467)—suggesting sustained neutralization may be required for effective IL-22 suppression, while transient blockade may be insufficient during active inflammation. These findings highlight dosing strategy as a key variable in interpreting cytokine neutralization data. Our results further implicate IL-17 as a promising therapeutic target for NEC.

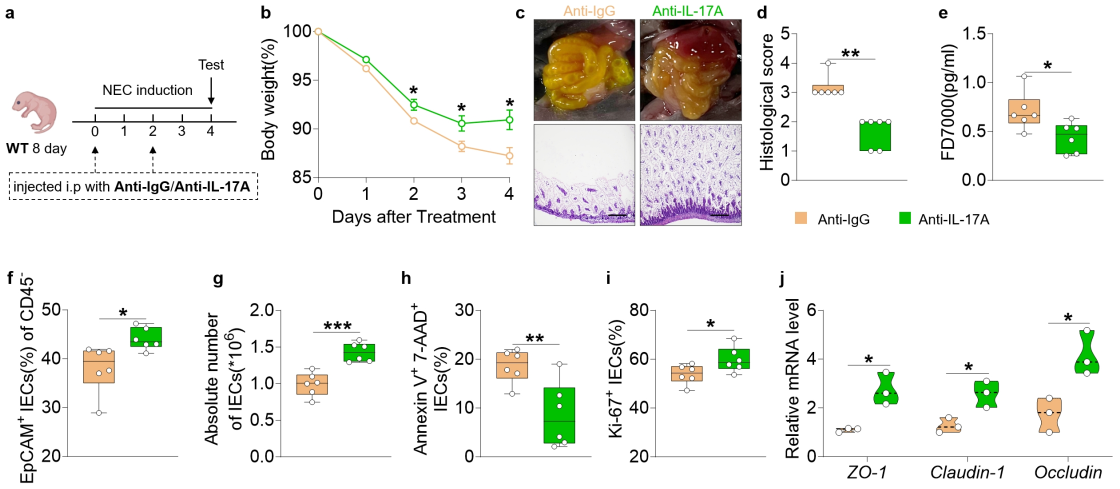

Figure E. Anti-IL-17A treatment significantly alleviates NEC severity and repairs intestinal barrier injury. (**a**) Experimental schema of neutralizing anti-IL-17A antibody (anti-IL-17A) during NEC induction. (**b**) Body weight changes in the indicated groups (n = 6). (**c**) Macroscopic image and H&E staining of the intestines (scale bar: 100 μm). (**d**) Statistical analysis of inflammation scores (n = 6). (**e**) Intestinal permeability was assessed by plasma FD7000 levels (n = 6). (**f**, **g**) Percentage and absolute number of IECs (n = 6). (**h**) Statistical analysis of Annexin V^+ 7-AAD^+ IECs (n = 6). (**i**) Statistical analysis of $Ki67^+$ IECs (n = 6). (**j**) Relative mRNA levels of of *ZO-1*, *Claudin-1*, and *Occludin* (n = 3 biological replicates). Each symbol represents one pup from a litter, and data are from at least three independent experiments. Error bars represent the SEM. *p < 0.05; **p < 0.01; ***p < 0.001; ns, not significant. Statistical significance was analyzed by Student's t test (**b** and **d-j**).

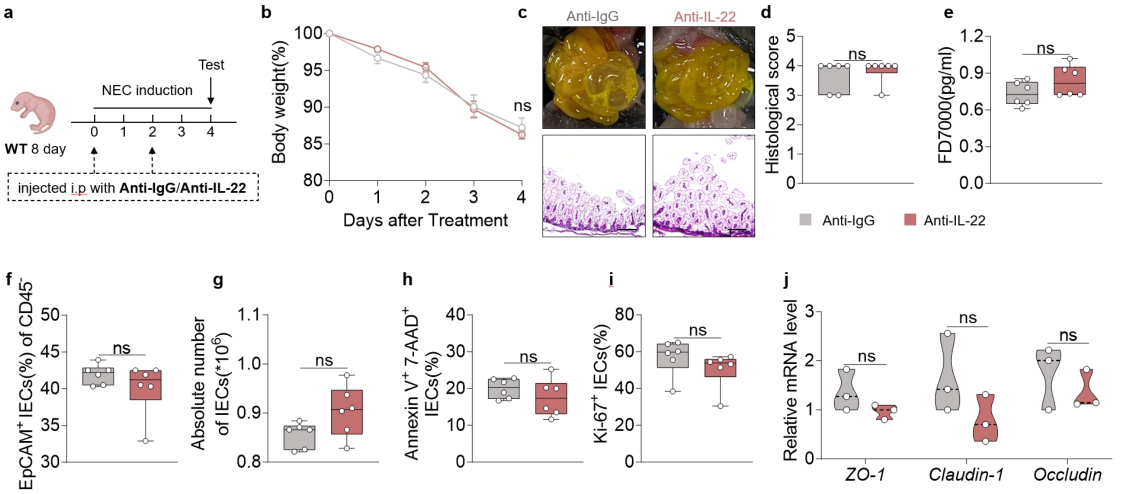

Figure F. Anti-IL-22 treatment has no effects on NEC alleviation and intestinal barrier repair. (**a**) Experimental schema of neutralizing anti-IL-22 antibody (anti-IL-22) during NEC induction. (**b**) Body weight changes in the indicated groups (n = 6). (**c**) Macroscopic image and H&E staining of the intestines (scale bar: 100 μm). (**d**) Statistical analysis of inflammation scores (n = 6). (**e**) Intestinal permeability was assessed by plasma FD7000 levels (n = 6). (**f**, **g**) Percentage and absolute number of IECs (n = 6). (**h**) Statistical analysis of Annexin V^+ 7-AAD^+ IECs (n = 6). (**i**) Statistical analysis of $Ki67^+$ IECs (n = 6). (**j**) Relative mRNA levels of of *ZO-1*, *Claudin-1*, and *Occludin* (n = 3 biological replicates). Each symbol represents one pup from a litter, and data are from at least three independent experiments. Error bars represent the SEM. *$p < 0.05$; **$p < 0.01$; ***$p < 0.001$; ns, not significant. Statistical significance was analyzed by Student's t test (**b** and **d-j**).

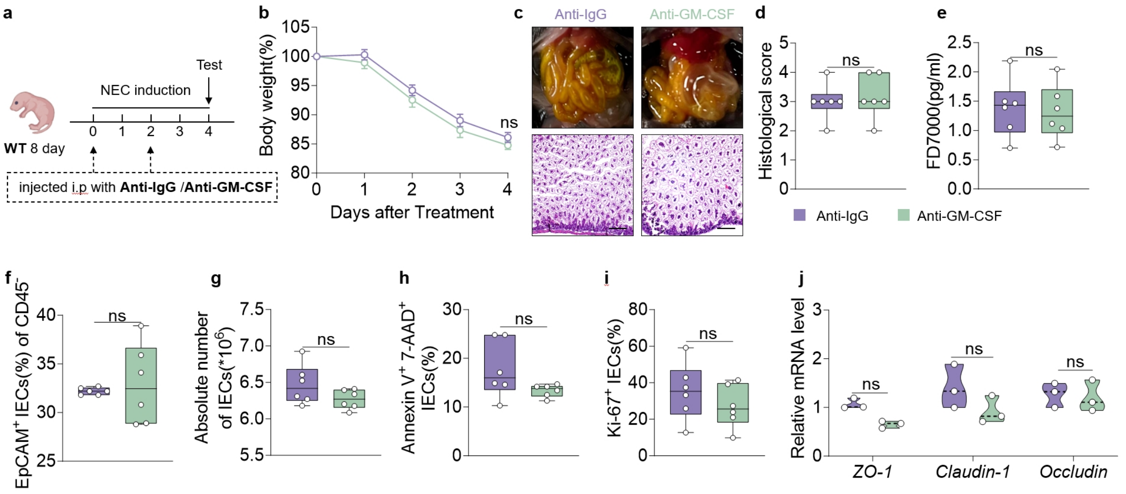

Figure G. Anti-GM-CSF treatment has no effects on NEC alleviation and intestinal barrier repair. (**a**) Experimental schema of neutralizing anti-GM-CSF antibody (anti-GM-CSF) during NEC induction. (**b**) Body weight changes in the indicated groups (n = 6). (**c**) Macroscopic image and H&E staining of the intestines (scale bar: 100 μm). (**d**) Statistical analysis of inflammation scores (n = 6). (**e**) Intestinal permeability was assessed by plasma FD7000 levels (n = 6). (**f**, **g**) Percentage

and absolute number of IECs (n = 6). (**h**) Statistical analysis of Annexin V$^+$ 7-AAD$^+$ IECs (n = 6). (**i**) Statistical analysis of Ki67$^+$ IECs (n = 6). (**j**) Relative mRNA levels of of *ZO-1*, *Claudin-1*, and *Occludin* (n = 3 biological replicates). Each symbol represents one pup from a litter, and data are from at least three independent experiments. Error bars represent the SEM. *$p < 0.05$; **$p < 0.01$; ***$p < 0.001$; ns, not significant. Statistical significance was analyzed by Student's t test (**b** and **d-j**).

The supplementary data and corresponding description have been incorporated into the revised version (Figure 2j–q; Supplementary Figure 13a–p), (Page 10–11, Lines 221–238; Page 24, Lines 540–545).

6. Evidence for autophagic vesicles: The authors should provide a higher-resolution image demonstrating the presence of autophagic vesicles in NEC ILC3s. In Fig. 3c, it is not clear whether the observed structures possess double or single membranes

Reply: We sincerely appreciate your rigorous review and apologize for not clearly presenting the transmission electron microscopy (TEM) image. To improve the clarity and accuracy of our autophagy results, we have replaced the original image with a higher-resolution version. In NEC DN ILC3s, characteristic double-membraned autophagosomes (red box) were clearly observed, whereas control DN ILC3s showed no detectable autophagic structures (Figure H a).

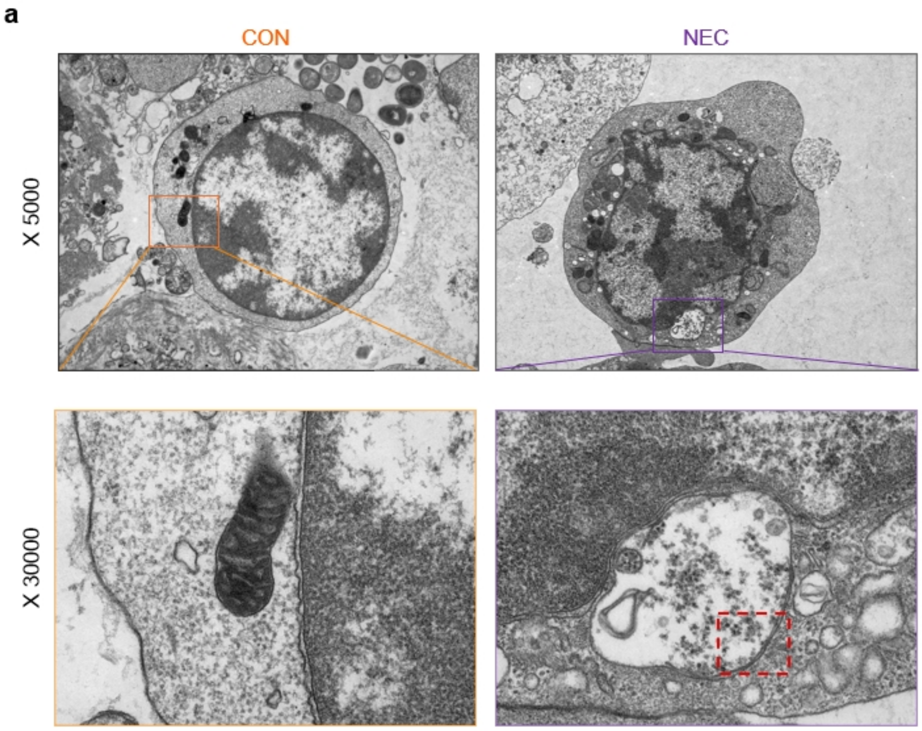

Figure H. Autophagy is activated in NEC DN ILC3s. (**a**) Representative transmission electron microscopy images of intestinal DN ILC3s sorted from CON and NEC neonates.

The supplementary high-resolution TEM images have been incorporated into the revised version (Figure 3c).
